# Elderly stroke burden: a comprehensive global study over three decades

**DOI:** 10.3389/fragi.2025.1489914

**Published:** 2025-07-15

**Authors:** Ruoyu Gou, Changjun Luo, Xudong Liang, Shuitao Qin, Hao Wu, Bing Li, Fengqi Pan, Jinwei Li, Jun-An Chen

**Affiliations:** ^1^ School of Public Health, Ningxia Medical University, Yinchuan, Ningxia, China; ^2^ Department of Cardiology, Affiliated Liutie Central Hospital of Guangxi Medical University, Liuzhou, China; ^3^ Department of Science and Education, Affiliated Liutie Central Hospital of Guangxi Medical University, Liuzhou, China; ^4^ Department of Neurosurgery, West China Hospital, Sichuan University, Chengdu, Sichuan, China; ^5^ Department of Clinical Nutrition, Affiliated Liutie Central Hospital of Guangxi Medical University, Liuzhou, China

**Keywords:** GBD, stroke, elderly patient, disability-adjusted life-years, incidence, mortality

## Abstract

**Background:**

Stroke is a serious disease that negatively affects the quality of life of patients and has become a global public health problem. This study used data from the 2021 Global Burden of Disease (GBD) study to assess the burden of stroke in the elderly population between 1990 and 2021.

**Methods:**

This cross-sectional study utilised data from the Global Burden of Disease (GBD) 2021, encompassing 204 countries and territories. The analysis included data from older patients who had experienced a stroke. The analysis includes morbidity, all-cause and cause-specific mortality, disability-adjusted life years, and corresponding estimated annual percentage changes (APCs).

**Results:**

From 1990 to 2021, the total number of stroke cases among the global elderly population rose from approximately 4.39 million to 8.19 million, with the age-standardized incidence (ASIR) decreasing from 996.06 cases per 100,000 people to 775.68 cases per 100,000 people (EAPC-0.784). The number of deaths increased from 4.08 million to 6.19 million, and the age-standardized mortality rate (ASMR) decreased from 981.87 to 600.08 deaths per 100,000 people (EAPC-1.446). The number of disability-adjusted life years (DALYs) increased from 75.96 million to approximately 111.14 million, with the age-standardized DALYs rate (ASDR) declining (EAPC-1.596). In five Socio-Demographic Index (SDI) regions, the disease burden was higher in men than in women.

**Conclusion:**

The number of stroke cases, deaths and DALYs increased in the elderly, while the global incidence, mortality and mortality rates of stroke decreased, with a higher burden on older men than women.

## 1 Introduction

There is no doubt that stroke is one of the leading causes of death and disability in the world, especially in countries with low or middle incomes (Kim et al., 2020). In the last few decades, disease patterns have shifted from communicable to non-communicable diseases in 80% of developing countries, and both population growth and aging are increasing deaths from non-communicable diseases and injuries, with stroke being one of the salients and debilitating diseases, ranking as the second death from the leading cause (Lozano et al., 2012). Several factors contribute to the risk of stroke, including air pollution, smoking, physical inactivity, and metabolic factors. If effective primary prevention strategies are not urgently implemented, there will likely be an increase in the global burden of stroke (Feigin et al., 2021). Epidemiology suggests that stroke has a similar age-specific incidence in women and men up to the age of 55 years, but men have a higher incidence at 55–75 years (Gorelick, 2019). Analyzing the Global Burden of Disease Study 2019 in a systematic manner indicates that 12.2 million individuals will experience a stroke and 6.55 million will succumb to its consequences in 2019 (Feigin et al., 2021), and Individuals aged 50 years and older are more likely to suffer from stroke than any other cause of disability-adjusted life years (DALYs) (Vos et al., 2020). It is estimated that Stroke prevalence will rise in the U.S over time, from 3.9% in 2020 to 6.4% by 2050 (Joynt Maddox et al., 2024). It is common for elderly patients who have suffered a stroke to be admitted to hospitals in the United States with a number of additional health conditions, collectively known as comorbidities (e.g., simple hypertension 55.4%, paralysis 40.1%, congestive heart failure 39.8%, and various neurological disorders 38.3%), which significantly increases the burden of care and in-hospital mortality (Chen and Li, 2023). By 2021, It is estimated that approximately 34% of the total global expenditure on healthcare will be allocated to the treatment of stroke (Rochmah et al., 2021), which has become a global public health issue as the population ages and puts a heavy burden on patients and their caregivers.

The global burden of stroke is on the rise, yet primary prevention strategies have not received adequate attention, and existing preventive measures have proven insufficient (Owolabi et al., 2022). Reducing stroke incidence in the elderly population is a critical objective to alleviate the socioeconomic burden and reduce healthcare resource consumption. Consequently, it is essential to updatinge risk estimates and the burden of disease is particularly critical toto effectively prevent the onset of stroke and its associated complications. As far as we are aware, there is lack of long-term trend analysis in the literature in the global epidemiology of stroke in the elderly population. This study utilized the GBD database to analyze the trends in stroke incidence, stroke-related mortality, and disability-adjusted life years (DALYs) among the elderly population from 1990 to 2021, along with the associated risk factors. By interpreting these data, we aim to provide a solid theoretical and data-driven foundation for future research, policy development, and practical interventions, with the goal of reducing the disease burden on this vulnerable group, improving their quality of life, and lowering disability and mortality rates.

## 2 Materials and methods

### 2.1 Data source

The GBD 2021 project conducted a comprehensive assessment of the incidence, prevalence, mortality, and DALYs associated with 371 diseases and injuries, as well as the health loss attributable to 88 risk factors, across 204 countries and territories from 1990 to 2021, using a uniform and comparable modeling approach. Data were systematically collected from various sources, including censuses, household surveys, civil registration and vital statistics, disease registries, health service use, air pollution monitoring, satellite imagery, disease notifications, and others.1 Details regarding the study design and methods of the GBD research have been extensively described in existing GBD literature.13, 14 Data on overall stroke and its pathological types were obtained from the Global Health Data Exchange (https://ghdx.healthdata.org/gbd‐result-stool) (Visited on 20 May2024). Additionally, the study employed the sociodemographic index (SDI), which quantifies a region’s sociodemographic development based on income, education, and fertility rates.

### 2.2 Case definition

According to the clinical standards of the World Health Organization, stroke is described as a symptom of brain dysfunction, which is usually focal and rapidly developing, lasting more than 24 h or leading to death. Ischemic stroke refers to neurological impairment caused by local infarction in the brain, spinal cord, or retina. Cerebral hemorrhage is defined as a stroke caused by non-traumatic intracerebral hematoma. Subarachnoid hemorrhage is defined as non-traumatic bleeding in the subarachnoid space of the brain. The Global Burden of Disease (GBD) study classifies causes into four levels, from the broadest level 1 (such as non-communicable diseases) to the most specific level 4 (such as ischemic stroke). Stroke is classified as a level 3 cause, and its subtypes are classified as level 4 causes. Although GBD 2021 provides detailed stroke data, it should be noted that the data in GBD 2021 do not clearly distinguish between first strokes and recurrent strokes. This analysis is based on comprehensive stroke data (Aho et al., 1980).

### 2.3 Overview and data collection

This cross-sectional study was approved by the Liuzhou Liutie Central Hospital. The informed consent waiver was granted by the Liuzhou Liutie Central Hospital Committee as the study involved only data analysis and no identifiable personal information. Available data, defining diseases in a standard way, and other information on stroke in the elderly population were collected using the GlobalHealth Data Exchange query tool created by GBD collaborators (Aho et al., 1980; von Elm et al., 2008).

The data are available from the GBD Results Tool of the Global Health Data Exchange (http://ghdx.healthdata.org/gbd‐results‐tool) (Visited on 20 May2024). GBD 2021 complies with the guidelines for accurate and transparent health assessment reports (GATHER).

In this study, senile patients were categorized into 8 groups: 60–64 years, 65–69 years, 70–74 years, 75–79 years, 80–84 years, 85–89 years, 90–94 years, and 95 years and older. We collected data on the amount of stroke episodes and morbidity, stroke-related deaths and mortality, and stroke-related DALYs in older populations, as well as corresponding rates at the global, regional, and national levels. We used the GBD standard population structure estimated in terms of numbers (i.e., counts) and age-standardised rates per 100,000 people. We then compared age-standardized rates between 1990 and 2021 and investigated temporal and spatial patterns based on age, sex, year, and geographic location. Data on race and ethnicity of participants are not presented in the GBD database, which does not assign race and ethnicity for data collection. To determine the average estimated yearly percentage change (EAPC), we employed linear regression. This research adhered to the STROBE guidelines for reporting observational studies (von Elm et al., 2007).

### 2.4 Socio-demographic index

An SDI is a measure of the socio-economic development of a country or region based on data on fertility, education, and income per capita. The SDI ranges from 0 to 1; higher levels indicate greater socioeconomic development. The SDI has been reported to be associated with disease morbidity and mortality. In this study, countries and geographic regions were categorized into 5 SDI regions (low, low-moderate, moderate, moderate-high, and high) to analyze the relationship between stroke burden and socioeconomic development among the elderly (aged 60 years and older) (GBD, 2019).

### 2.5 Risk factor estimation

The 2021 Global Burden of Disease study estimated the attributable burden of diseases for 88 risk factors and combinations at the global, regional, and national levels. We also gathered data on global risk factors related to childhood diabetes mortality. However, only 19 risk factors contributed to the stroke burden in the 2021 stroke analysis. Published literature provides detailed information on the risk factor definitions and specific estimation methods used in the 2021 Global Burden of Disease study. As with the causes, GBD classifies risk factors into four levels, from the broadest (level 1) to the most specific (level 4) (GBD, 2021b).

### 2.6 Fatal disease modelling

We use life registration and cause-of-death inference data as input to the Cause of Death Inference Model (CODEm) framework to estimate deaths from stroke and its subtypes. CODEm is a flexible modeling tool that uses geospatial relationships and covariate information to estimate mortality across all locations over the time series from 1990 to 2019. It reclassifies deaths in the vital statistics system, which are coded as impossible, intermediate causes of death, or unreported, using statistical methods. Detailed descriptions have been reported in previous studies (Johnson et al., 2015).

### 2.7 Non-fatal disease modelling

The DisMod-MR 2.1 (Disease Model Bayesian Meta-Regression) modeling tool was used to generate stroke incidence and prevalence estimates (GBD, 2021a). DisMod-MR is a Bayesian geospatial disease modeling software that uses data on various disease parameters, their epidemiological relationships, and geospatial relationships to generate prevalence and incidence estimates. All high-quality data on morbidity, prevalence, and mortality are used to estimate the burden of non-fatal stroke (GBD, 2019).

### 2.8 Statistical analysis

This study evaluates the global burden of stroke using various indicators, including the number of deaths, the number of cases, the number of Disability-Adjusted Life Years (DALYs), age-standardized mortality (ASMR), age-standardized disability-adjusted life years (DADRs), and age-standardized prevalence (ASIR), all within a 95% confidence interval (UI). By standardizing the global age structure, the age-standardized prevalence (ASR) is calculated, which is a crucial step in comparing different populations across geographical locations and time periods. The estimated annual percentage change (EAPC) is calculated as 100 × (exp(β) −1), with the 95% confidence interval (CI) derived from a linear regression model. The dynamic of stroke in the elderly is analyzed by calculating the EAPC to determine the temporal trend of the stroke burden. If the upper limit of the EAPC and the lower limit of its 95%CI are both negative, the corresponding ratio shows a downward trend; conversely, if the lower limit of the EAPC and the lower limit of its 95%CI are both positive, the corresponding ratio shows an upward trend.

An increased ASR trend is defined as the lower limit of the 95%CI of the EAPC estimate being greater than 0, while a decreased ASR is defined as the upper limit of the 95%CI of the EAPC estimate being less than 0. Statistical analysis was conducted using R-Studio version 4.1.2, with statistical significance set at p < 0.05. The detailed research methodology is documented in previous publications (GBD, 2019).

## 3 Results

### 3.1 Elderly population with stroke: Global trends

#### 3.1.1 Incidence

**FIGURE 1:**
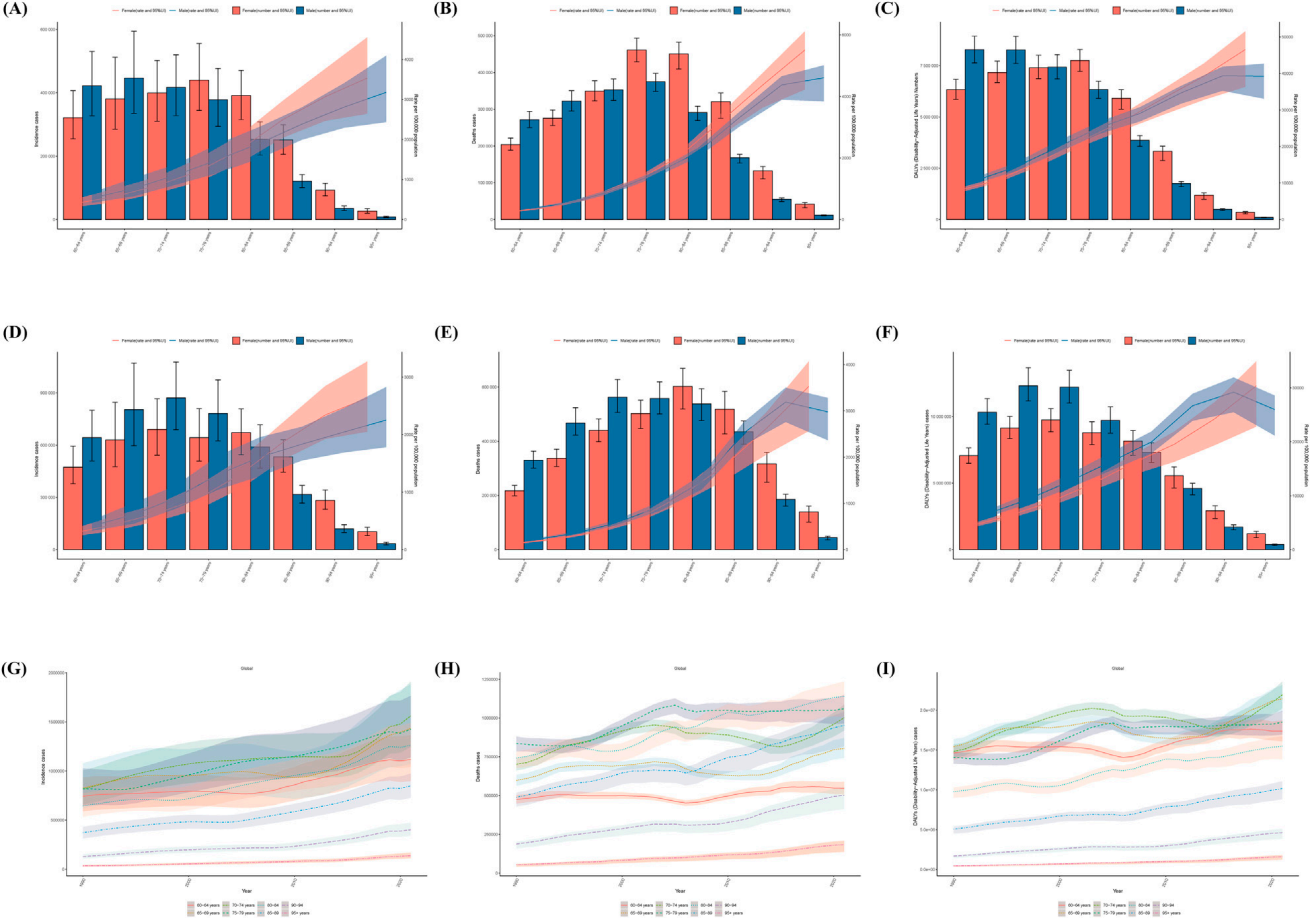
Trends in stroke Incidence, Deaths, and Disability-Adjusted Life-Years (DALYs) Among old people From 1990 to 2021. **(A–C)** are 1990 Incidence, Deaths, and Disability-Adjusted Life-Years (DALYs) by age. **(D–F)** are Incidence, Deaths, and Disability-Adjusted Life-Years (DALYs) by age in 2021. **(G–I)** are the number of Incidence, Deaths, and Disability-Adjusted Life-Years (DALYs) by age, 1990–2021.

In 1990, the highest incidence of stroke was in men aged 65–69 years, with 446,384.70 cases (95% uncertainty interval [UI], 334,954.79–594,096.31). The highest number of stroke cases occurred in women aged 75–79 years, with 439,721.36 cases (95% UI, 344,087.64–555,788.68). There were more cases of stroke in males than in females (60–74 years), and more females than males after the age of 74 years. As people aged, the incidence of stroke increased and females were more likely to suffer strokes than males. The incidence rate was 3,178.12 cases per 100,000 persons (95% UI, 2,436.12–4,093.96) for men over 95 years of age and 3,534.35 cases per 100,000 persons (95% UI, 2,642.57–4,554.63) for women (Figure 1A; Supplementary Table S1-1).

In the year 2021, the number of cases of stroke in older men and women showed an increasing and then decreasing trend. Men aged 70–74 years had the largest number of strokes, 871,144.57 (95% UI, 688,817.07–1,077,527.98). Older women had the highest number of cases of stroke at 70–74 years of age, 690,905 (95% UI, 542,727–866,864). There were more cases of stroke in males than in females in the age group of 60–74 years, and more females than males after the age of 75 years. The incidence of stroke in the elderly increased with age and was greater in females than in males, with an incidence rate of 2,252.77 (95% UI, 1782.52–2,827.18) per 100,000 persons in males over 95 years of age, compared with 2,637.40 (95% UI, 2051.70–3,273.11) (Figure 1D; Supplementary Table S1-1).

From 1990 to 2021, The incidence of stroke in the elderly population in all age groups is on the rise. In 2021, the lowest number of incidence cases was in the 95+ age group with 137,930.73 cases (95% UI, 111,259.88–168,710.38), and incidence cases were highest in the 70–74 age group with 1,562,049.46 (95% UI, 1,247,981.48–1,912,002.85) (Figure 1G; Supplementary Table S1-2).

From 1990 to 2021, the total number of stroke cases in the global elderly population increased from 4,385,489.98 (95% UI, 3,499,176.06–5,447,763.45) to 8,190,238.52 (6,578,255.45–10,033,645.12), with a downward trend in the age-standardized incidence rate (ASIR) of from 996.06 (95% UI, 798.74–1,230.66) to 775.68 (95% UI, 624.60–947.96) per 100,000 population, with an EAPC of −0.784 (−0.693 to −0.875) (Supplementary Table S3-1; Supplementary Table S4-1; Supplementary Table S6-1).

#### 3.1.2 Mortality

In 1990, the number of stroke deaths in elderly men and women showed an increasing and then decreasing trend, with the highest number of stroke deaths in elderly men and women aged 75–79 years, 374,919.32 (95% UI, 348,726.34–397,735.11) in men and 439,721.36 (95% UI, 344,087.64–555,788.68) in women. The number of stroke deaths in men before 75–79 years of age was greater than that in women, and after 80 years of age, there were more women than men. Men were more likely than women to die of strokes until they were 75–79 years old, and women after 80 years old than men. Stroke mortality increased with age, but was higher for women.

The mortality rate was 4,605.31 deaths per 100,000 persons (95% UI, 3,842.30–5,011.79) for men and 5,508.45 deaths per 100,000 persons (4,224.98–6,117.80) for women over the age of 95 years (Figure 1B; Supplementary Table S1-1). Compared to men, women died at a higher rate.

In 2021, the number of stroke deaths in older men and women, showed an increasing and then decreasing trend. It was the 70–74 year old males who died the most, 562,423.43 (95% UI, 506,562.02–627,615.74). Older females had the highest number of deaths at 80–84 years of age, 602,669.73 (95% UI, 518,909.2195–669,091.5803). There were more stroke deaths among males than females in the 60–79 years age group, and more females than males after 80 years of age. Stroke mortality in older women increased with age, with a rate of 3,530.37 deaths per 100,000 (95% UI, 2,565.77–4,069.81) in women aged 95 years and older.

In older men, stroke mortality rates increased and then decreased, with the highest mortality rate in the 90–94 years age group, which was 3,189.92 per 100,000 people (95% UI, 2,758.98–3,501.82). The stroke mortality rate for men was higher than that for women until 90–94 years of age, and higher for women than for men after 95 years of age (Figure 1E; Supplementary Tables S1-1).

From 1990 to 2021, the death rate is on the rise from stroke in all age groups of the elderly population. In 2021, the lowest number of deaths was in the 95+ age group at 184,066.65 (95% UI, 137,067.85–209,579.45), and the highest number of deaths was in the 80–84 age group at 1,140,304.34 (95% UI, 1,006,577.48–1,235,802.86) (Figure 1H; Supplementary Table S1-2).

From 1990 to 2021, the total number of stroke deaths in the global elderly population increased from 4,082,291.7 (95% UI, 3,764,144.994–4,325,839.34) to 6,194,922.11 (95% UI, 5,507,552.42–6,739,225.14), with a decreasing trend in the age-standardized mortality ratio (ASMR), which increased from 1 per 10,000 population.) showed a decreasing trend from 981.87 cases per 100,000 (95% UI, 895.79–1,042.22) to 600.08 cases per 100,000 (95% UI, 531.63–653.22), with an EAPC of −1.446 (95% UI, −1.592 to 1.3) (Supplementary Table S3-1; Supplementary Table S4-1; Supplementary Table S6-1).

#### 3.1.3 DALYs

In 1990, the number of cases of DALYs in elderly males showed a gradual decreasing trend with age, and the number of cases of DALYs in males aged 60–64 years was 8,285,744.32 (95% UI, 7,630,948.93–8,935,725.70). The number of cases of stroke DALYs in females, which showed an increasing and then decreasing trend, was highest at 75–79 years of age, with 7,751,640.45 cases (95% UI, 7,222,111.26–8,289,884.52).

STROKEDALYs were more common among elderly males than females up to the age of 70–74 years, and among females after 75 years than males.

The rate of stroke DALYs raised with age and was higher among females than among males. The rate of DALYs was 39,243.25 cases per 100,000 persons (95% UI, 33,090.15–42,658.16) for males over 95 years of age, and 46,557.14 cases (36,348.75–51,560.78) for females (Figure 1C; Supplementary Tables S1-1).

In 2021, the number of cases of STROKE DALYs in older men and women showed an increasing and then decreasing trend. Males aged 65–69 years had the largest number of cases of DALYs at 12,313,537.36 (95% UI, 11,168,359.11–13,668,696.33). Older females had the largest number of cases of DALYs among the age group 70–74 years with 9,739,641.96 cases (95% UI, 8,856,143.29–10,597,736.49). More cases of stroke DALYs were found in males than females among the age group of 60–79 years and more females than males in the age group of 80 years onwards. The rate of stroke DALYs in older women increased with age, with a rate of 30,373.61 cases per 100,000 (95% UI, 22,800.60–34,892.60) in women over 95 years of age. The rate of stroke DALYs in older men showed a trend of an increase then followed by a decrease, and the highest rate of DALYs was in the age group of 90–94 years, which was 29,196.62 cases per 100,000 people (95% UI, 25,472.68 to 31,920.38). DALYs associated with stroke were higher among men than among women until the age of 90–94 years, and it was higher among women than among men after the age of 95 years (Figure 1F; Supplementary Table S1-1).

From 1990 to 2021, there was an increasing trend in DALYs cases in all age groups of the elderly population. In 2021, the lowest number of DALYs cases was in the 95+ age group at 1,588,783.39 (95% UI, 1,222,221.76–1,802,468.37), and the highest number of DALYs cases was among the 70–74 age group at 21,946,243.35 (95% UI, 20,233,361.82–23,618,316.73) (Figure 1I; Supplementary Tables S1-S2).

From 1990 to 2021, the total number of cases of stroke DALYs in the global elderly population increased from 75,956,899.12 cases (95% UI, 70,805,222.6–80,424,317.66) to 111,138,701.1 cases (95% UI, 100,826,175.2–120,130,503), with an age-standardised DALYs rate (ASDR) showed a decreasing trend from 16,791.36547 cases per 100,000 people (95% UI, 15,558.90–17,794.67) to 10,454.28 cases (95% UI, 9,462.83–11,305.68), with an EAPC of −1.596 (95% UI, −1.736 to - 1.457) (Supplementary Table S3-1; Supplementary Table S4-1; Supplementary Table S6-1).

### 3.2 Elderly population with STROKE: SDI regional trends

#### 3.2.1 Incidence

In 1990, the region with the highest ASIR for stroke in the elderly was High-middle SDI, with 1,243.59 cases per 100,000 people (95% UI, 1,544.32 to 985.85), and among the regions with the lowest ASIR was the High SDI region, with 798.34 cases per 100,000 people (95% UI, 990.23 to 634.28).

In 2021, the area with the highest ASIR for stroke in older adults was the Middle SDI, with 948.45 cases per 100,000 (95% UI, 1,165.45 to 764.35), and the area with the lowest ASIR was the High SDI, with 471.37 cases per 100,000 (95% UI, 576.23 to 381.42).

From 1990 to 2021, the ASIR for stroke in older adults showed a downward trend, with SDI declines fastest in High SDI, from 798.34 cases per 100,000 (95% UI, 990.23 to 634.28) to 471.37 cases per 100,000 (95% UI, 576.23 to 381.42).

**FIGURE 2:**
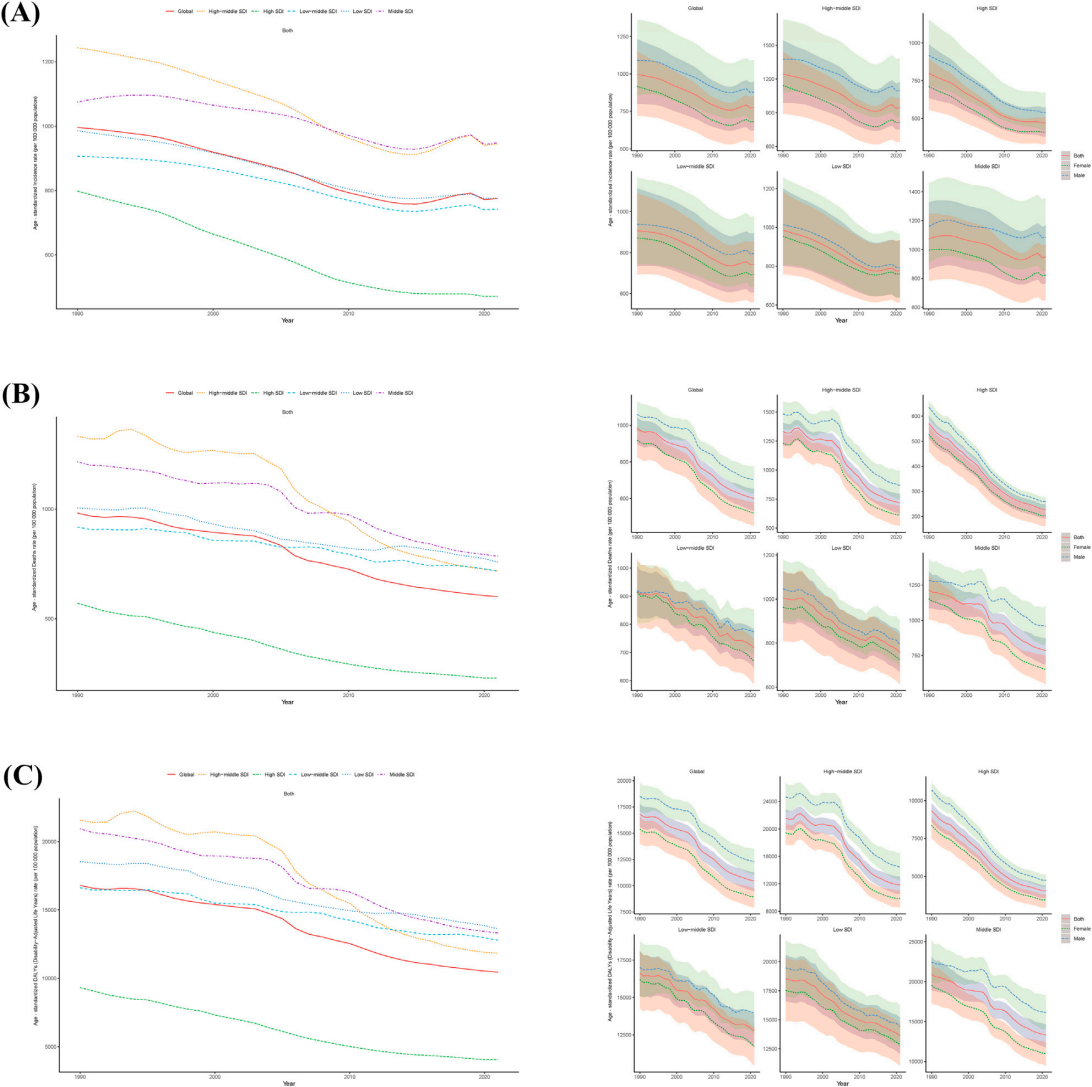
Epidemiologic Trends of Incidence, Death, and Disability-Adjusted Life-Years (DALYs) Age-standardized Rates (ASR) in 5 Sociodemographic Index (SDI) Regions of old people stroke From 1990 to 2021. **(A)**, Trends in Age-standardized incidence rate. **(B)**, Trends in Age-standardized death rate. **(C)**, Trends in Age-standardized DALYs rate.

From 1990 to 2021, the ASIR for stroke in older men was higher than the global level and the ASIR for stroke in women was lower than the global level in all 5 SDI regions. The High SDI region had the greatest reduction in ASIR for both males and females, from 916.68 (95% UI, 716.98–1,156.02) to 539.74 (95% UI, 426.15–674.26) per 100,000 for males, and from 710.56 (95% UI, 556.26–892.90) to 410.96 cases (95% UI, 329.60–508.68) per 100,000 people (Figure 2A; Supplementary Table S2-1; Supplementary Table S2-2).

#### 3.2.2 Mortality

In 1990, the highest ASMR for stroke in the elderly was in the High-middle SDI at 1,332.34 cases per 100,000 (95% UI, 1,213.00–1,409.37), and the lowest ASIR was in the High SDI at 570.36 cases per 100,000 (95% UI, 509.92–600.80).

In 2021, the region with the highest ASMR for stroke in older adults was Middle SDI, with 785.40 cases per 100,000 (95% UI, 685.69–873.14), and the region with the lowest ASIR was High SDI, with 228.90 cases per 100,000 (95% UI, 192.76–249.20).

From 1990 to 2021, the ASMR for stroke in the elderly showed a downward trend, with the fastest decline being in the High-middle SDI region, from 1,332.34 cases per 100,000 ((95% UI, 1,213.00–1,409.37) to 718.01 cases (95% UI, 625.14–797.24).

From 1990 to 2021, the ASMR for stroke in older men was higher than the global level and the ASMR for stroke in women was lower than the global level in all five SDI regions. The reduction in ASMR was greatest in the High-middle SDI region for both males and females, decreasing from 1,090.21 (95% UI, 856.67–1,364.37) to 879.23 (696.26–1,093.14) per 100,000 for males and from 916.19 (95% UI, 721.56–1,148.25) to 683.64 (95% UI, 544.99–853.18) per 100,000 people (Figure 2B; Supplementary Table S2-1; Supplementary Table S2-2).

#### 3.2.3 DALYs

In 1990, ASDR-highest region for stroke in the elderly was High-middle SDI, with 21,570.88 cases per 100,000 people (95% UI, 19,903.05–22,817.12), and ASDR-lowest region was High SDI, with 9,325.45 cases per 100,000 people (95% UI, 8,577.33–9,831.74).

In 2021, ASDR-highest region ASDR for stroke in older adults was Low SDI with 13,613.90 cases per 100,000 people (95% UI, 12,058.95–15,182.96), and ASDR-lowest was High SDI with 4,053.49 cases per 100,000 people (95% UI, 3,565.64 to 4,418.90).

From 1990 to 2021, the ASDR for stroke in the elderly showed a downward trend, with the fastest decline in the High-middle SDI region, from 21,570.88 cases per 100,000 people (19,903.05–22,817.12) to 11,828.12 cases per 100,000 people (95% UI, 10,514.83 to 13,067.78).

From 1990 to 2021, the ASDR for stroke in older men was higher than the global level and the ASDR for stroke in women was lower than the global level in all five SDI regions. The High SDI region had the greatest reduction in male and female ASDR, with male ASDR decreasing from 1,090.21 cases per 10,000 people (95% UI, 856.67–1,364.37) to 879.23 cases per 10,000 people (696.26–1,093.14), and female ASMR decreasing from 916.1916894 (721.5638162–1,148.245214) to 683.6444101 (544.9863882–853.1835689) (Figure 2C; Supplementary Table S2-1; Supplementary Table S2-2).

### 3.3 Elderly population with stroke: geographic regional trends

#### 3.3.1 Incidence

In 1990, the ASIR for stroke among older adults in the 21 geographic regions was highest in East Asia at 1,357.14 cases per 100,000 (95% UI, 1,072.28–1,697.86) and lowest in Andean Latin America at 554.66 cases per 100,000 (95% UI, 454.80–671.57). The highest number of males was in East Asia at 1,530.84 cases per 100,000 people (95% UI, 1,198.80–1943.56) and the lowest was in Andean Latin America at 577.97 cases per 100,000 people (95% UI, 458.95–715.29).

The highest number of females was in Eastern Europe at 1,326.99 cases per 100,000 people (95% UI, 975.04–1765.65) and the lowest was in Australasia at 587.73 cases per 100,000 people (95% UI, 488.77–696.61).

In 2021, the ASIR for stroke in older adults in the 21 geographic regions was highest in East Asia at 1,248.63 cases per 100,000 (95% UI, 989.09–1,566.21) and lowest in Andean Latin America at 367.66 cases per 100,000 (95% UI, 301.84–442.88). Among males, the highest was in East Asia with 1,476.04 cases per 100,000 (95% UI, 1,149.67–1877.58), and the lowest was in Andean Latin America with 392.75 cases per 100,000 (95% UI, 312.89–484.49). The highest number of females was in Central Asia at 1,132.99 cases per 100,000 people (95% UI, 927.15–1,360.24) and the lowest was in Australasia at 323.91 cases (95% UI, 259.48–399.19).

**FIGURE 3:**
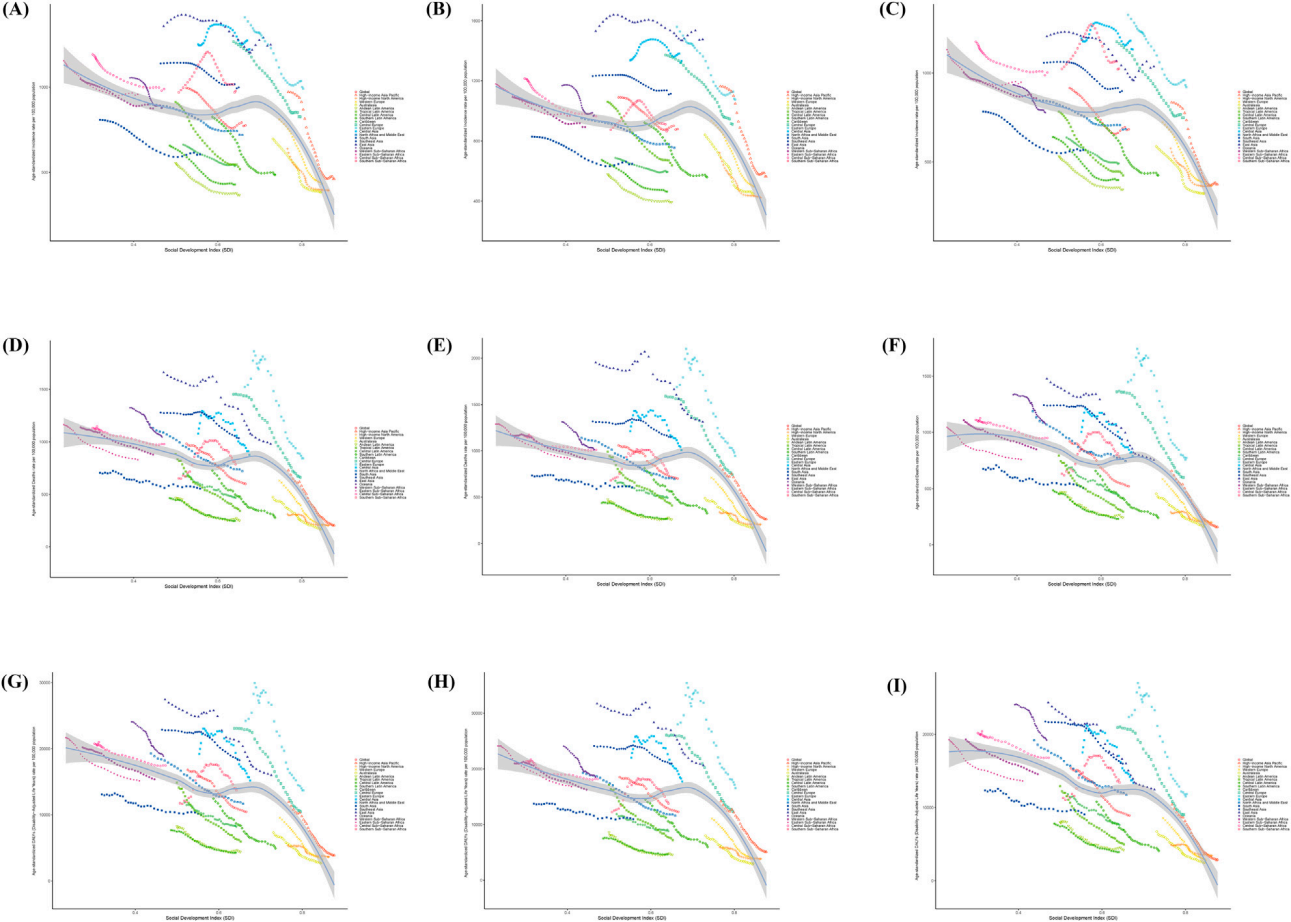
Incidence, Death, and Disability-Adjusted Life-Years (DALYs) Age-standardized Rates for old people stroke From 1990 to 2021. **(A–C)** Sex Stratification: Sex-Specific, Global and 21 GBD Regions Age-standardised Incidence Rates. **(D–F)** Gender Stratification: Age-standardised Death Rates for different genders, globally and for the 21 GBD regions. **(G–I)** Sex-stratified: gender-specific, global and 21 GBD regions Age-standardised Disability-Adjusted Life-Years (DALYs) Rates.

From 1990 to 2021, the ASIR for stroke in older people in 21 geographic regions, with the exception of Southern Sub-Saharan Africa and Central Asia, showed a decreasing trend. The fastest decline was in the region with the highest SDI, High-income Asia Pacific, from 1,163.71 (95% UI, 864.19–1,540.54) to 562.57 (95% UI, 441.43–703.80) cases per 100,000 people. The fastest declines for both older men and women were in High-income Asia Pacific, where males declined from 1,163.71 cases per 100,000 (95% UI, 864.19–1,540.54) to 422.49 cases per 10,000 (95% UI, 322.36 to 544.4u). Females decreased from 834.56 cases per 100,000 (95% UI, 638.73–1,078.49) to 366.62 (95% UI, 280.79–473.48) (Figures 3A–C; Supplementary Table S3-1).

#### 3.3.2 Mortality

In 1990, the ASMR for stroke in the elderly in the 21 geographic regions was highest in East Asia at 1,661.34 cases per 100,000 people (95% UI, 1,443.68–1871.67) and lowest in Central Latin America at 656.35 cases (95% UI, 527.57 to The lowest was in Central Latin America with 656.35 cases (95% UI, 527.57–808.22). The highest number of males was in East Asia at 1952.54 (95% UI, 1,664.45–2,225.58) per 100,000 and the lowest was in high-income North America at 339.40 (95% UI, 308.65–357.40) per 100,000 population. The highest number of females was in East Asia at 1,476.96 cases per 100,000 (95% UI, 1,235.17–1743.61), and the lowest was in high-income North America at 291.73 cases per 100,000 (95% UI, 245.58–316.46).

In 2021, the ASMR for stroke among older adults in the 21 geographic regions was highest in Oceania at 1,045.63 cases per 100,000 (95% UI, 838.15–1,274.64) and lowest in Australasia at 170.63 cases per 100,000 (95% UI, 139.99–190.98). The highest number of males was in East Asia at 1,313.34 cases per 100,000 (95% UI, 1,068.42–1,580.69) and the lowest was in Australasia at 172.58 cases per 100,000 (95% UI, 147.60–194.13). The highest for females was in Central Sub-Saharan Africa at 949.12 (653.02–1,330.73).

From 1990 to 2021, the ASMR for stroke in older people in 21 geographical regions showed a decreasing trend, except for Southern Sub-Saharan Africa, which showed an “inverted U” shape. The fastest decline was in Central Europe, from 1,453.62 cases per 100,000 (95% UI, 1,364.77–1,508.67) to 660.16 cases per 100,000 (95% UI, 590.06–711.57). The fastest declines in both older men and women were in Central Europe, where men declined from 1,590.62 cases per 100,000 (95% UI, 1,508.72–1,653.78) to 742.63 cases per 100,000 (671.63–801.30), and women from 1,360.19 (95% UI, 1,262.92–1,419.19) cases per 100,000 (95% UI, 1,262.92–1,419.30). 1,262.92–1,419.78) to 597.56 cases (95% UI, 526.02–649.56) per 100,000 in females (Figures 3D–F; Supplementary Table S3-1).

#### 3.3.3 DALYs

In 1990, the ASDR for stroke in the elderly in the 21 geographic regions was highest in East Asia at 27,428.30 cases per 100,000 (95% UI, 24,065.86–30,874.34) and lowest in Central Latin America at 7,667.55 (95% UI, 7,241.92–7,979.63). The largest number of males was in East Asia at 31,700.54 (95% UI, 26,828.17–36,434.73) per 100,000 population, and the lowest was in Central Latin America at 7,505.38 (95% UI, 7,092.31–7,855.77) per 100,000 population. The largest number of females was in East Asia with 24,351.63 cases (95% UI, 20,610.87–28,596.09) and the lowest was 6,719.08 cases (95% UI, 5,859.84–7,401.89).

In 2021, the ASDR for stroke in older adults in the 21 geographic regions was highest in Oceania at 18,883.04 cases per 100,000 people (95% UI, 15,227.06–22,940.52) and lowest in Australasia at 2,718.12 cases per 100,000 people (95% UI, 2,323.76–3,032.10). Among males, the highest was in Southeast Asia with 20,954.20 cases per 100,000 (18,499.08–23,378.10) and the lowest was in Australasia with 2,958.75 cases per 100,000 (95% UI, 2,593.50–3,312.92). The highest for females was Oceania at 19,216.51 (95% UI, 15,177.05–23,864.88) and the lowest was Australasia at 2,479.51 cases per 100,000 (95% UI, 2033.27–2,812.81).

From 1990 to 2021, the ASDR for stroke in older people declined in all 21 geographic regions, except for Southern Sub-Saharan Africa, which showed an “inverted U-shape”. The fastest decline was in Central Europe, from 23,074.63 cases per 100,000 people (95% UI, 21,907.18 to 23,920.57) to 10,492.90 cases per 100,000 people (95% UI, 9,529.75 to 11,274.16). The fastest declining region for both older males and females was Central Europe, from 26,069.53 (95% UI, 24,861.21 to 27,097.84) to 12,389.49 (95% UI, 11,312.35 to 13,356.70) per 100,000 for males. Females decreased from 20,952.95 cases (95% UI, 19,723.92–21,855.49) to 9,031.67 cases per 100,000 (8,097.09–9,769.70) (Figures 3G–I; Supplementary Table S3-1).

### 3.4 Elderly patients with stroke/national trends

#### 3.4.1 Incidence

**FIGURE 4:**
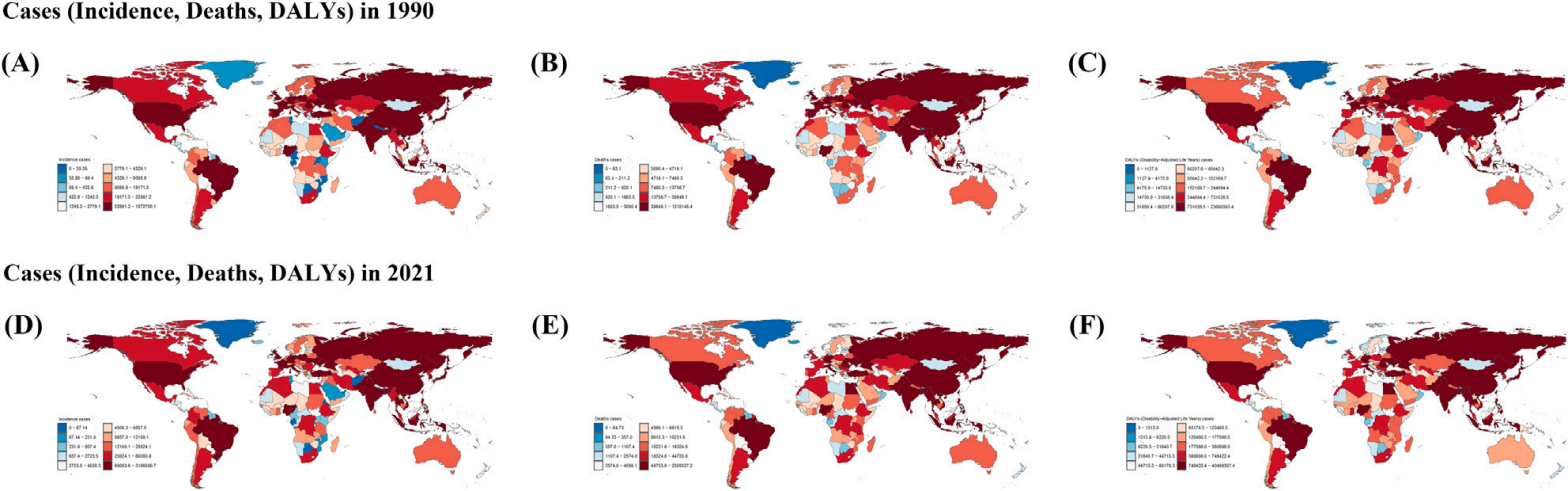
Incident, Death, and Disability-Adjusted Life-Years (DALYs) Cases of stroke in old people in 204 Countries and Territories. **(A–C)**: Cases for the three indicators Incident, Death, and Disability-Adjusted Life-Years (DALYs) in 1990. **(D–F)**: Cases for the three indicators Incident, Death, and Disability-Adjusted Life-Years (DALYs) in 2021.

**FIGURE 5:**
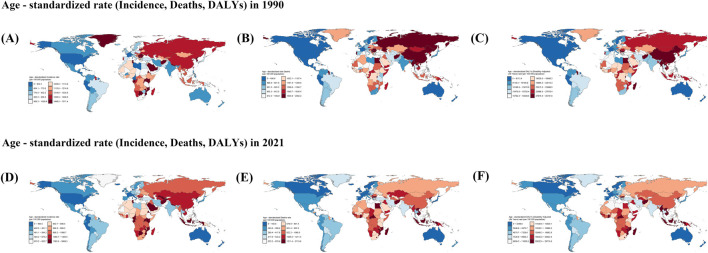
**(A–C)** Rate for the three indicators Incident, Death, and Disability‐Adjusted Life-Years (DALYs) in 1990. **(D–F)** Rate for the three indicators Incident, Death, and Disability‐Adjusted Life‐Years (DALYs) in 2021.

In 1990, among 204 countries, the Incidence at its highest of stroke in the elderly was in China, with 1,072,700.06 cases (95% UI, 838,117.95–1,357,338.11), and the lowest was in Botswana, with 1.71 (95% UI, 1.30–2.23) (Figure 4; Supplementary Table S4-1). The highest ASIR was in the United Arab Emirates at 7,371.35 cases per 100,000 people (95% UI, 6,081.447912–8,914.352914) and the lowest was in Puerto Rico at 464.03 cases per 100,000 people (95% UI, 377.28–566.24) (Figure 5; Supplementary Table S5-1).

In 2021, among 204 countries, the largest number of cases of stroke in the elderly was in China at 3,166,890.67 (95% UI, 2,487,278.39–4,008,386.59), and the lowest was in Tokelau at 1.39 (95% UI, 1.15–1.67) (Figure 4; Supplementary Table S4-1). The highest ASIR was in the United Arab Emirates at 5,999.35 cases per 100,000 people (95% UI, 4,894.31–7,250.12), and the lowest was in Puerto Rico at 283.36 cases per 100,000 people (95% UI, 228.93–348.76) (Figures 5, 7; Supplementary Table S5-1).

**FIGURE 6:**
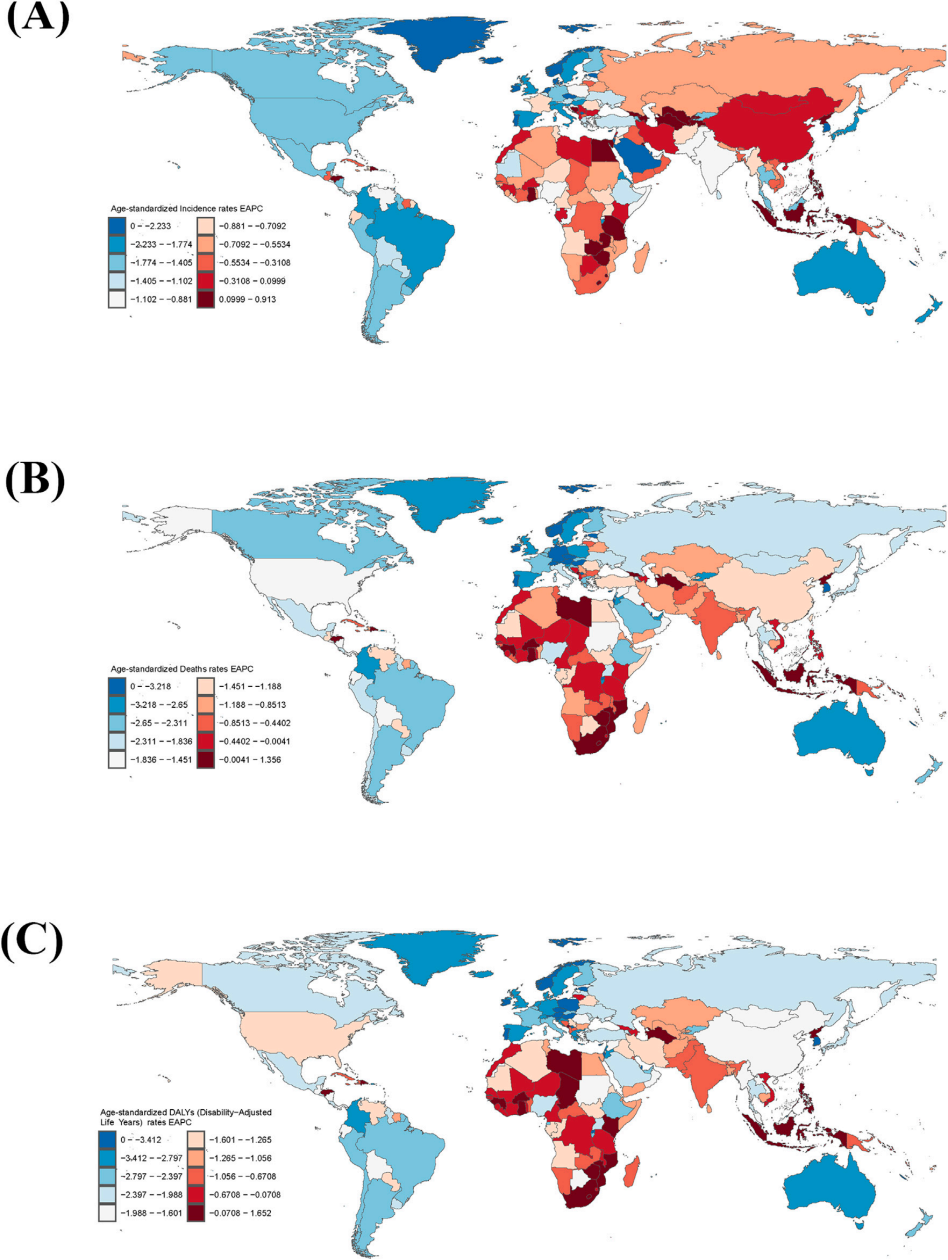
The national burden of stroke in old people in 204 countries and territories. **(A)** EAPC for Age-standardized incidence rate. **(B)** EAPC for Age-standardized deaths rate. **(C)** EAPC for Age-standardized DALYs rate. DALYs = disability-adjusted life-years; EAPC = estimated annual percentage change.

From 1990 to 2021, most-increasing country in ASIR for stroke in the elderly was Azerbaijan, with an EPAC of 0.91 (95% UI, 1.28 to −0.55), and the country with the greatest decrease was Singapore, with an EAPC of −4.178 (95% UI, −3.879 to −4.475) (Figure 6; Supplementary Table S6-1).

#### 3.4.2 Mortality

In 1990, highest death toll country among the elderly out of 204 countries was China, with 1,215,145.41 cases (95% UI, 1,058,315.22 to 1,374,886.84), and the lowest was Tokelau, with 2.18 cases (95% UI, 1.682214685–2.701595263) (Figure 4; Supplementary Table S4-1). The country with the highest ASMD was Serbia with 2,362.20 cases per 100,000 people (95% UI, 2063.99–2,646.98), and the lowest was Andorra with 297.81 cases per 100,000 people (95% UI, 203.03–408.66) (Figure 5; Supplementary Table S5-1).

**FIGURE 7:**
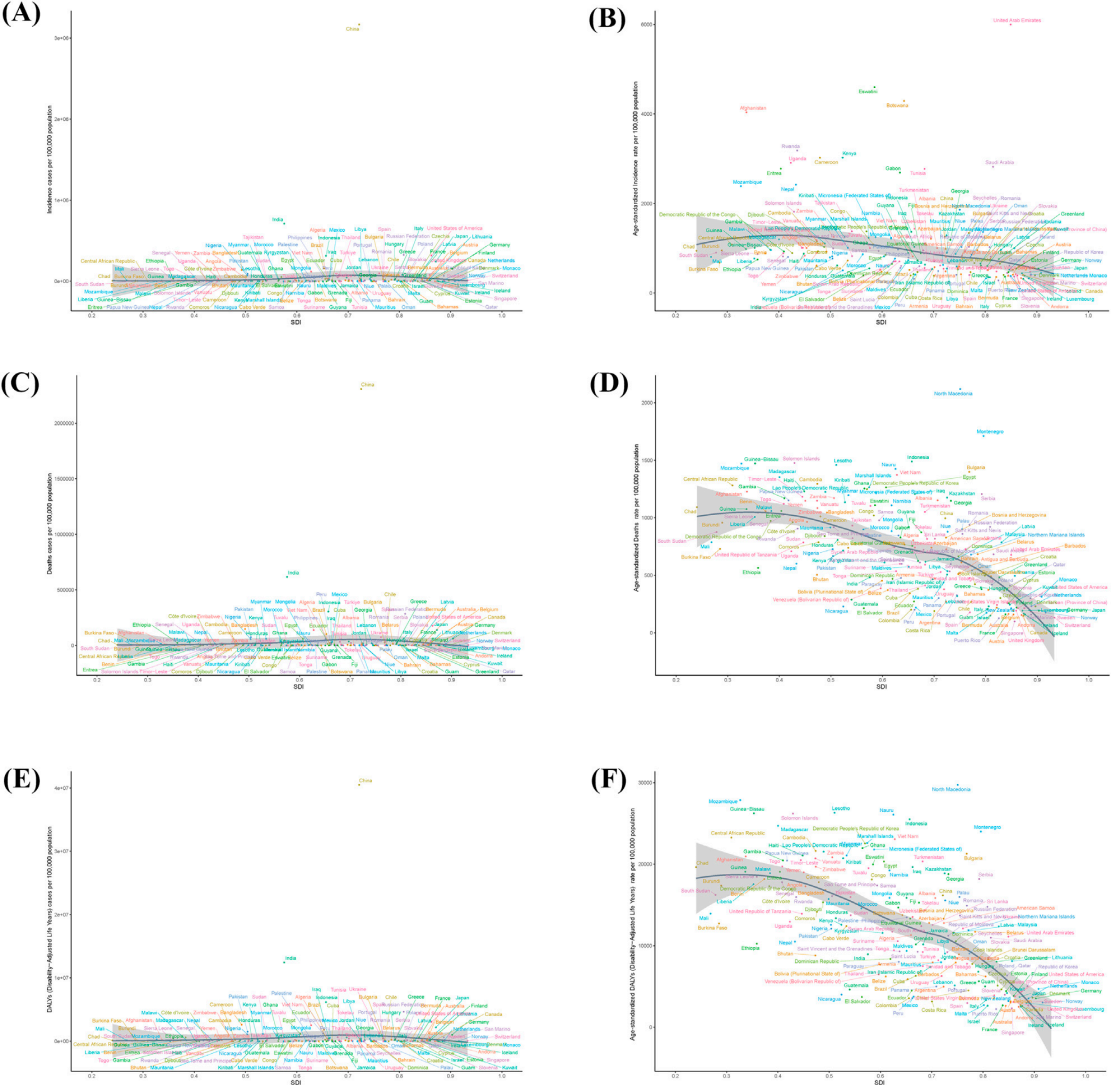
Incidence, deaths and DALYs Age-standardized rates of stroke in old people in 204 countries by SDI in 2021. **(A,B)** Age-standardised Incidence rate on the right. Number of cases on the left. **(C,D)** Age-standardised Deaths rate. And the number of cases. **(E,F)** Age-standardised DALYs rate. And number of cases. DALYs = disability-adjusted life-years; SDI = socio-demographic index.

In 2021, the highest number of deaths among the elderly among 204 countries was in China, at 2,309,327.19 (95% UI, 1,914,584.71–2,704,650.51), and the lowest was in Tokelau, at 1.54 (95% UI, 1.12–1.99) (Figure 4; Supplementary Table S4-1). The country with the highest ASMD was North Macedonia, at 297.81 cases per 100,000 (95% UI, 203.03–408.66) (Figure 5; Supplementary Table S5-1). The country with the highest ASMD was the United States. The country with the highest ASMD was North Macedonia with 2,119.77 cases per 100,000 people (95% UI, 1778.57–2,487.36), and the country with the highest ASMD was Singapore with 98.44 cases per 100,000 people (95% UI, 81.37–112.41) (Figures 5, 7; Supplementary Table S5-1).

From 1990 to 2021, the country with the most elevated ASMR for stroke in the elderly was Zimbabwe, with an EPAC of 1.36 (−95% UI, 0.72–2.00), and the country with the most declining ASMR was Singapore, with an EAPC of −5.764 (95% UI, −6.125 to 5.402) (Figure 6; Supplementary Tables S6-S1).

#### 3.4.3 DALYs

In 1990, the country with the largest number of cases of DALYs in the elderly out of 204 countries was China, with 23,690,393.42 cases (95% UI, 20,774,471.48–26,775,345.01), and the lowest was Tokelau, with 42.45 cases (95% UI, 33.10–52.25) (Figure 4; Supplementary Table S4-1). The highest ASDR was in Rwanda at 35,781.63 cases per 100,000 people (95% UI, 27,989.36–44,471.31), and the lowest was in Andorra at 4,745.39 cases per 100,000 people (95% UI, 3,398.02–6,382.21) (Figure 5; Supplementary Table S5-1).

In 2021, the country with the largest number of cases of DALYs in the elderly out of 204 countries was China, with 40,488,307.39 cases (95% UI, 33,961,030.88–47,055,553.98), and the lowest was Tokelau, with 27.55 cases (95% UI, 20.77–35.02) (Figure 4; Supplementary Table S4-1). The highest ASDR was in North Macedonia with 29,718.80 cases per 100,000 people (95% UI, 24,743.39–35,044.86) and the lowest was in Switzerland with 2,144.30 cases per 100,000 people (95% UI, 1796.218548–2,422.094322) (Figures 5, 7; Supplementary Table S5-1).

From 1990 to 2021, the country with the most elevated ASDR for stroke in the elderly was Lesotho, with an EAPC of 1.652 (95% UI, −1.222–2.084), and the country with the most declining ASDR was Estonia, with an EAPC of −5.729 (95% UI, −6.24 to −5.216) (Figure 6; Supplementary Table S6-1).

### 3.5 Attributable risk factors for stroke death and DALY in the elderly

Globally, death and DALY in old age stroke can be affected to 33 risk factors, covering three main categories: behavior (Dietary risks, Tobacco, Smoking, Diet high in sodium, Alcohol use diet low in fruits, Low physical activity, Diet low in fiber, Diet low in whole grains, Diet low in vegetables, Diet high in processed meat, Diet high in sugar-sweetened beverages, Diet low in polyunsaturated fatty acids, Diet high in red meat), environments (Air pollution, Solid fuel pollution in households, Particulate matter pollution, Ambient particulate matter pollution, Non-optimal temperature, Low temperature, Other environmental risks, Lead exposure, Secondhand smoke, High temperature), metabolic factor (Systolic hypertension, High LDL cholesterol, Hyperglycemia at fasting, Kidney dysfunction, High body-mass index).

From 1990 to 2021, all risk factors for stroke attributable to death in the elderly showed a decreasing trend except for High temperature, which increased from 5.46 (95% UI, −0.22–14.34) to 6.07 (95% UI, 0.51–14.75).

All risk factors attributable to death in the elderly in 1990 were 847.32 (95% UI, 752.89–922.82), with the highest being metabolic factors at 687.13 (95% UI, 559.15–795.31), and the lowest Diet high in red meat at −13.05 (95% UI, 13.05), and the lowest being Diet high in red meat, −0.22 to 14.34 (95% UI, 0.51–14.75). 13.05 (95% UI, −53.7 to 20.5).

**FIGURE 8:**
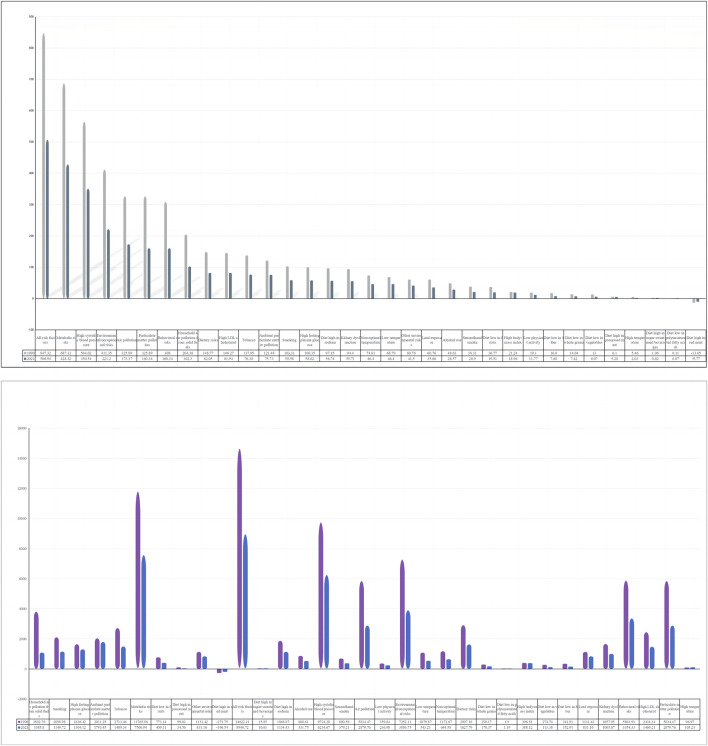
Proportion of old people stroke DALYs and deaths attributable to risk factors.

All risk factors for stroke attributable to death in older adults in 2021 were 506.94 (95% UI, 440.34–567.06), with the highest being metabolic factors at 428.12 (95% UI, 346.04–501.23), and the lowest being Diet high in red meat at - 9.77 (95% UI, −41.3 to 15.1) (Figure 8).

From 1990 to 2021, risk factors for stroke attributable to DALY in older adults showed a decreasing trend except for High temperature, which increased from 96.97 (95% UI, 0.62–247.61) to 105.21 (95% UI, 13.07–249.23).

All risk factors attributable to DALY for stroke in the elderly in 1990 were 14,622.21 (95% UI, 13,181.38–15,851.72), with the highest being metabolic factors at 11,765.96 (95% UI, 9,624.86–13,580.2), and the lowest at −273.75 (95% UI, −1,124.55 to 429.2).

All risk factors attributable to DALY for stroke in older adults in 2021 were 8,940.72 (95% UI, 7,935.91–9,893.65), with the highest being metabolic factors at 7,560.94 (95% UI, 6,180.41–8,766.83), and the lowest being Diet high in red meat with −196.54 (95% UI, −838.04 to 303.35).

## 4 Discussion

Globally, the second most common cause of disability and death is stroke. The disease has a disproportionate impact on low- and middle-income countries (Saini et al., 2021). From 1990 to 2019, the absolute incidence of stroke increased by 70.0% (67.0–73.0), an 85.0% increase in the prevalence of stroke (83.0–88.0), and a 43.0% deaths from strokes are on the rise (31.0–55.0) (Feigin et al., 2021), and the majority of the global burden of stroke (86.0% [95% UI 85.9–86.9] of deaths and 89.0% [88.9–89.3] of DALYs) occurs in low-income and lower-middle-income countries. Here, we investigated stroke incidence, mortality, and DALY by sex, globally and in 21 GBD regions, 204 countries, 1990–2021. The findings presented herein offer perspectives on the burden of stroke in regions and countries of varying income levels over the past 32 years. This study examines the period from 1990 to 2021, revealing that although global stroke incidence and mortality have decreased during this time, the burden of stroke among the elderly population has intensified in certain regions and countries. A comprehensive evaluation of stroke prevalence will significantly aid governments and healthcare organizations in developing strategies for stroke prevention and management.

From 1990 to 2021, age-standardised rates of stroke in all age groups in the global elderly population demonstrated a downward trajectory. However, the number of cases, deaths, and disability-adjusted life years (DALYs) in the elderly population exhibited an upward trend. Studies have indicated that stroke-related deaths significantly increased from 1990 to 2019, despite a significant decrease in standardized by age of stroke rates worldwide, especially among people aged 70 and older (Feigin et al., 2021). From 1990 to 2019, global stroke burden increased significantly. This was evidenced by a 70.0% rise in the number of cases, a 43.0% increase in mortality, a 102.0% rise in prevalence, and a 143.0% surge in disability-adjusted life years (DALYs). Furthermore, a substantial proportion of the global stroke burden, comprising 86.0% of stroke-related deaths and 89.0% of DALYs, is attributed to low- and lower-middle incomes Countries (LMICs) (Feigin et al., 2021) (Feigin et al., 2022). At the same time, the functional impact of stroke is more severe in the elderly, as evaluated by various prognostic scales, and stroke statistically accelerates age-dependent functional decline, with an incidence of almost three times the annual spontaneous increase. An inflammatory state in peripheral tissues combined with an immunosuppressive state increases the risk of post-stroke infection in the elderly (Gallizioli et al., 2023).

In addition, we found that SDI was significantly and negatively associated with stroke incidence, stroke-related mortality, and the number of stroke-related patients, and the highest decrease in stroke ASMR was in the high SDI regions, with the fastest decrease in Central Europe; the largest decreases in ASDR were observed in men and women in regions with a high-middle SDI. The reason for these declines is believed to be the backwardness of basic medical facilities and the inadequacy of measures for early identification and effective control of stroke-related risk factors compared with those in high SDI areas. In the majority of high SDI and medium-high SDI regions, a comprehensive stroke risk factor detection system has been established, which effectively controls the risk factors of stroke. Regardless of the stroke burden index used, the high-SDI and high-middle SDI regions consistently demonstrated the most significant downward trend (Mi et al., 2023).

With regard to gender differences, according to our study data, ASIR, ASMR, and ASDR levels were higher for older men and lower for women in all 5 SDI regions than global stroke levels. Conversely, in the same regions, the ASIR, ASMR, and ASDR were lower than global levels in women with stroke. Earlier studies have indicated that strokes are more common in men than in women, which is thought to be due to suboptimal temperature control. This phenomenon is linked to disparate physiological processes in males and females. For example, in a mouse model of stroke, it was observed that female astrocytes exhibited estrogen-dependent protection (Qu et al., 2024). Furthermore, men have been observed to sweat at a greater volume than women, and thus are more susceptible to electrolyte imbalances. This may result in an elevated risk of stroke in men relative to women when body temperature rises. In addition, the gender difference in stroke burden is associated with a higher proportion of men exposed to two of the top 10 most important risk factors for stroke—smoking and alcohol consumption, as well as lower health awareness and chronic disease treatment compliance among men (Truyen et al., 2025). The incidence of strokes in older women increases with age; in 2021, the incidence rate will be 2,252.77 cases per 100,000 people for men and 2,637.40 cases per 100,000 people for women over 95 years of age; related studies have shown that up to the age of 75 years, age-standardized stroke incidence is lower in women than in men. Beyond age 75, the trend is actually reversed. In fact, It is estimated that approximately 55,000 additional fatal strokes occur in women annually compared to men (Thomas et al., 2021). And because women have a longer life expectancy and therefore accumulate more stroke events, a recent analysis of data from the United Kingdom Biobank showed similar associations between blood pressure, body weight, lipids, diabetes, atrial fibrillation, and any stroke in two sexs, but that hypertensive, smoking and low socio-economic status were stronger risk factors for women (Dawson and MacDonald, 2023) Also gender was associated with differences in the incidence of stroke types, with a higher prevalence and incidence of intracranial aneurysms and a higher incidence of subarachnoid hemorrhage in women compared to men (Rexrode et al., 2022).

From 1990 to 2021, the ASIR for stroke in older people in 21 geographical regions, except Southern Sub-Saharan Africa and Central Asia, showed a decreasing trend. Previous studies suggest that stroke incidence and stroke prevalence may be 2–3 times higher in Africa than in Western Europe and the United States. In Africa, published data for 1990–2021 show an annual incidence of stroke as high as 316 per 100,000 people, a prevalence as high as 1,460 per 100,000 people, and a 3-year mortality rate of more than 80% (Akinyemi et al., 2021). In 2019, the three regions with the highest stroke burdens were Central Asia (ASMR 18.12, 95% UI 13.4–24.53; ASDR 327.35, 95% UI 240.24–440.61), Eastern Europe (ASMR 15.71, 95% UI 9.34–25.13; ASDR 268.96, 95% UI 159.42–430.71) and East Asia. Of particular concern is the marked increase in the age-standardized death rate (ASDR) among Central Asian women (Qu et al., 2024).

In 2021, China had the highest disease burden index of any country in the world, with 433,907 cases and 202,7919 cases associated with DALYs. Additionally, Deaths occurred most frequently in China associated with this figure at 90,516. China has the highest lifetime risk of stroke at 25 years and beyond. In 2020, 2.6% of people in China had a stroke, compared to 1.2% globally (Tu et al., 2023a). As the most populous developing country, Stroke-related risk factors are on the rise in China, including diabetes and hypertension, which are not adequately controlled. This is occurring concurrently with an ageing population, which is contributing to an overall rise in the burden of stroke disease. Furthermore, substantial regional disparities in stroke incidence (e.g., stroke bands) and treatment accessibility (greater availability in eastern coastal regions than in less developed western regions) present a significant obstacle to effective stroke prevention and control strategies in China (Tu et al., 2023b).

Stroke is a prevalent and recurrent neurological disorder that can result in a range of neurological impairments, impacting activities such as walking, communication, and other aspects of daily living (Saini et al., 2021). The substantial rise globally incidence of stroke is attributable not only to population growth and an ageing population, but also to a notable increase Individuals in number exposed to a number of significant risk factors, including high BMI, Pollution, high blood sugar, hypertension, alcohol, inactivity, kidney problems and heat (Feigin et al., 2021; Api et al., 2021). Meanwhile, in our study, we found that the risk factors attributed to DALY in older adults, with the exception of High temperature, showed a trend of downward from 1990 to 2021. This is due to the fact that the incidence of heat-induced stroke in the context of global warming represents a significant public health concern on a global scale, particularly among individuals aged 65–75 years, males, and in countries with a low SDI (Bo et al., 2023). All risk factors for stroke attributable to DALY in older adults in 1990 and 2021 were the highest. Of the metabolic factors, hypertension is the leading cause of stroke deaths (Gąsecki et al., 2021), while LDL-C is the most useful serum lipid marker for predicting stroke risk. There is a strong association between the risk of ischaemic stroke and elevated LDL-C. Dyslipidaemia represents a significant risk factor for stroke after hypertension, diabetes, and smoking (Chang et al., 2023). Stroke risk factors in total attributable to death in the elderly were followed by systolic hypertension and Environmental occupational risks. In 2019, 52.57% of stroke deaths and 55.54% of disability years of injury and brain injury were attributable to HSBP, globally. The prevalence is higher in men (Li et al., 2024). PM2.5 is the fourth biggest risk factor for ischaemic stroke worldwide, according to the 2019 GBD (Chen et al., 2022).

It should be noted that this study is subject to a number of limitations. Firstly, the analysis was mainly based on the GBD database, which is subject to certain limitations. Data from national registries limits the accuracy of the database,a significant number of older adults remain undiagnosed following a stroke, and the lack of information on additional risk factors associated with stroke in older adults, while the data in iterations of the GBD were similar, GBD’s inherent limitations meant that the results were not comparable across iterations, even in the same year. The discrepancies observed in the GBD results across iterations can be attributed to the incorporation of updated data sources and enhancements to the methodology. Secondly, the GBD 2021 estimates the burden of disease associated with stroke primarily through the analysis of data from individual countries and a review of the existing literature. It is regrettable that high-quality epidemiological studies of stroke are scarce in some countries, which may introduce some bias in the estimation of the model. Thirdly, alterations to screening and diagnostic techniques since 1990, coupled with a heightened awareness of stroke among both patients and physicians, may have resulted in an uptick in reported cases, potentially influencing the interpretation of trends in stroke incidence. Fourthly, In calculating the overall burden, the total population was taken into account, the minimum age of onset of stroke was set at 60 years in this study, which may have resulted in an underestimation of the burden of stroke in younger populations. Fifth, the distinction between ischemic and hemorrhagic stroke was not adequately considered in this study, which may have led to biased estimates of the disease burden of stroke. Sixth, the conclusions of this stroke epidemiologic study should be viewed with caution and are expected to be further validated in larger studies in the future.

## 5 Conclusion

In elderly stroke patients, whose biological changes with age may influence existing age/sex associations with stroke risk factors, hypertension, diabetes mellitus, and cardiovascular complications typically occur with age. For example, much of the increase in hypertension is attributable to changes in the cardiovascular system, arterial structure, and stiffness of large arteries with age. Cardiovascular problems and diabetes are often associated with these aging lifestyles (Teh et al., 2018). In addition, the greater impact of certain risk factors (e.g., tobacco use, poor diet, diabetes, hypertension, cardiovascular disease, rheumatic heart disease, dyslipidemia, and obesity) on prevalence in low-income countries compared with middle- and upper-income countries may also be associated with their diminished access to acute healthcare for stroke (Feigin et al., 2021). This failure may partially This failure may partly explain the lower stroke-related mortality in older people in areas with a higher SDI than in areas with a lower SDI.

Stroke is a significant global health concern, with an increasing prevalence, mortality, and burden DALYs among the elderly. Notwithstanding the global decline in ASIR, ASMR, and ASDR, older men bear a greater burden of disease than women. Preventing and controlling strokes in the elderly can be improved with a better understanding of its epidemiology.

## Data Availability

The datasets presented in this study can be found in online repositories. The names of the repository/repositories and accession number(s) can be found in the article/Supplementary Material.

## References

[B1] Aho K. Harmsen P. Hatano S. Marquardsen J. Smirnov V. E. Strasser T. (1980). Cerebrovascular disease in the community: results of a WHO Collaborative Study. Bull. World Health Organ 58 (1), 113–130. 6966542 PMC2395897

[B2] Akinyemi R. O. Ovbiagele B. Adeniji O. A. Sarfo F. S. Abd-Allah F. Adoukonou T. (2021). Stroke in Africa: profile, progress, prospects and priorities. Nat. Rev. Neurol. 17 (10), 634–656. 10.1038/s41582-021-00542-4 34526674 PMC8441961

[B3] Api A. M. Belsito D. Botelho D. Bruze M. Burton G. A. Jr Buschmann J. (2021). RIFM fragrance ingredient safety assessment, cis-3-hexenyl isovalerate, CAS Registry Number 35154-45-1. Food Chem. Toxicol. 156, 112533. 10.1016/j.fct.2021.112533 34487799

[B4] Bo Y. Zhu Y. Lu R. Chen L. Wen W. Jiang B. (2023). Burden of stroke attributable to high ambient temperature from 1990 to 2019: a global analysis. Int. J. Stroke 18 (9), 1121–1131. 10.1177/17474930231183858 37300302

[B5] Chang Y. Eom S. Kim M. Song T. J. (2023). Medical management of dyslipidemia for secondary stroke prevention: narrative review. Medicina 59 (4), 776. 10.3390/medicina59040776 37109734 PMC10141044

[B6] Chen W. Li D. (2023). Comorbidity and outcomes among hospitalized patients with stroke: a nationwide inpatient analysis. Front. Neurol. 14, 1217404. 10.3389/fneur.2023.1217404 37915378 PMC10616246

[B7] Chen Z. Liu P. Xia X. Wang L. Li X. (2022). The underlying mechanism of PM2.5-induced ischemic stroke. Environ. Pollut. 310, 119827. 10.1016/j.envpol.2022.119827 35917837

[B8] Dawson J. MacDonald A. (2023). Sex and hypertensive organ damage: stroke. J. Hum. Hypertens. 37 (8), 644–648. 10.1038/s41371-023-00830-0 37059829 PMC10403348

[B9] Feigin V. L. Brainin M. Norrving B. Martins S. Sacco R. L. Hacke W. (2022). World stroke organization (WSO): global stroke fact sheet 2022. Int. J. Stroke 17 (1), 18–29. 10.1177/17474930211065917 34986727

[B10] Feigin V. L. Stark B. A. Johnson C. O. (2021). Global, regional, and national burden of stroke and its risk factors, 1990–2019: a systematic analysis for the Global Burden of Disease Study 2019. Lancet Neurology 20 (10), 795–820. 10.1016/S1474-4422(21)00252-0 34487721 PMC8443449

[B11] Gallizioli M. Arbaizar-Rovirosa M. Brea D. Planas A. M. (2023). Differences in the post-stroke innate immune response between young and old. Semin. Immunopathol. 45 (3), 367–376. 10.1007/s00281-023-00990-8 37045990 PMC10279582

[B12] Gąsecki D. Kwarciany M. Kowalczyk K. Narkiewicz K. Karaszewski B. (2021). Blood pressure management in acute ischemic stroke. Curr. Hypertens. Rep. 23 (1), 3. 10.1007/s11906-020-01120-7 PMC7728631 33305339

[B13] GBD (2019). Global, regional, and national burden of stroke and its risk factors, 1990–2019: a systematic analysis for the global burden of disease study 2019 - the lancet neurology. Available online at: https://www.thelancet.com/journals/laneur/article/PIIS1474-4422(21)00252-0/fulltext (Accessed June 5, 2025). 10.1016/S1474-4422(21)00252-0 PMC8443449 34487721

[B14] GBD (2021a). Global age-sex-specific mortality, life expectancy, and population estimates in 204 countries and territories and 811 subnational locations, 1950–2021, and the impact of the COVID-19 pandemic: a comprehensive demographic analysis for the Global Burden of Disease Study 2021 - the Lancet. Available online at: https://www.thelancet.com/journals/lancet/article/PIIS0140-6736(24)00476-8/fulltext (Accessed June 5, 2025). 10.1016/S0140-6736(24)00476-8 PMC11126395 38484753

[B15] GBD (2021b). Global burden and strength of evidence for 88 risk factors in 204 countries and 811 subnational locations, 1990–2021: a systematic analysis for the Global Burden of Disease Study 2021 - the Lancet. Available online at: https://www.thelancet.com/journals/lancet/article/PIIS0140-6736(24)00933-4/fulltext (Accessed June 5, 2025). 10.1016/S0140-6736(24)00933-4 PMC11120204 38762324

[B16] Gorelick P. B. (2019). The global burden of stroke: persistent and disabling. Lancet Neurology 18 (5), 417–418. 10.1016/S1474-4422(19)30030-4 30871943

[B17] Johnson C. O. Nguyen G. Naghavi M. Feigin V. L. Murray C. J. L. Roth G. A. (2015). Methods for estimating the global burden of cerebrovascular diseases. Neuroepidemiology 45 (3), 146–151. Available online at: https://karger.com/ned/article-abstract/45/3/146/226556/Methods-for-Estimating-the-Global-Burden-of?redirectedFrom=fulltext (Accessed June 5, 2025). 26505980 10.1159/000441083

[B18] Joynt Maddox K. E. Elkind M. S. V. Aparicio H. J. Commodore-Mensah Y. de Ferranti S. D. Dowd W. N. (2024). Forecasting the burden of cardiovascular disease and stroke in the United States through 2050—prevalence of risk factors and disease: a presidential advisory from the American heart association. Circulation 150 (4), e65–e88. 10.1161/CIR.0000000000001256 38832505

[B19] Kim J. Thayabaranathan T. Donnan G. A. Howard G. Howard V. J. Rothwell P. M. (2020). Global stroke statistics 2019. Int. J. Stroke 15 (8), 819–838. 10.1177/1747493020909545 32146867

[B20] Li J. Zhong Q. Yuan S. Zhu F. (2024). Global burden of stroke attributable to high systolic blood pressure in 204 countries and territories, 1990–2019. Front. Cardiovasc Med. 11, 1339910. 10.3389/fcvm.2024.1339910 38737709 PMC11084284

[B21] Lozano R. Naghavi M. Foreman K. Lim S. Shibuya K. Aboyans V. (2012). Global and regional mortality from 235 causes of death for 20 age groups in 1990 and 2010: a systematic analysis for the Global Burden of Disease Study 2010. Lancet 380 (9859), 2095–2128. 10.1016/S0140-6736(12)61728-0 23245604 PMC10790329

[B22] Mi Y. Huai L. Yin Y. Yuan J. Liu Y. Huang J. (2023). Burden of stroke in China and the different SDI regions over the world. J. Glob. Health 13, 04169. 10.7189/jogh.13.04169 38131457 PMC10740341

[B23] Owolabi M. O. Thrift A. G. Mahal A. Ishida M. Martins S. Johnson W. D. (2022). Primary stroke prevention worldwide: translating evidence into action. Lancet Public Health 7 (1), e74–e85. 10.1016/S2468-2667(21)00230-9 34756176 PMC8727355

[B24] Qu C. Chen Y. Liu C. Hu Z. Zhang J. Yan L. (2024). Burden of stroke attributable to nonoptimal temperature in 204 countries and territories: a population-based study, 1990-2019. Neurology 102 (9), e209299. 10.1212/WNL.0000000000209299 38598742 PMC11175652

[B25] Rexrode K. M. Madsen T. E. Yu A. Y. X. Carcel C. Lichtman J. H. Miller E. C. (2022). The impact of sex and gender on stroke. Circulation Res. 130 (4), 512–528. 10.1161/CIRCRESAHA.121.319915 35175851 PMC8890686

[B26] Rochmah T. N. Rahmawati I. T. Dahlui M. Budiarto W. Bilqis N. (2021). Economic burden of stroke disease: a systematic review. IJERPH 18 (14), 7552. 10.3390/ijerph18147552 34299999 PMC8307880

[B27] Saini V. Guada L. Yavagal D. R. (2021). Global epidemiology of stroke and access to acute ischemic stroke interventions. Neurology 97 (20_Suppl. ment_2), S6–S16. 10.1212/WNL.0000000000012781 34785599

[B28] Teh W. L. Abdin E. Vaingankar J. A. Seow E. Sagayadevan V. Shafie S. (2018). Prevalence of stroke, risk factors, disability and care needs in older adults in Singapore: results from the WiSE study. BMJ Open 8 (3), e020285. 10.1136/bmjopen-2017-020285 PMC5875611 29599393

[B29] Thomas Q. Crespy V. Duloquin G. Ndiaye M. Sauvant M. Béjot Y. (2021). Stroke in women: when gender matters. Rev. Neurol. 177 (8), 881–889. 10.1016/j.neurol.2021.01.012 34172293

[B30] Truyen T. T. Vo N. L. Vo Q. P. Phan T. C. Le P. N. B. Nguyen H. D. T. (2025). Burden and risk factors of stroke in vietnam from 1990 to 2021 – a systematic analysis from global burden disease 2021. J. stroke Cerebrovasc. Dis. 34, 108241. Available online at: https://www.strokejournal.org/article/S1052-3057(25)00020-5/fulltext (Accessed June 5, 2025). 39826583 10.1016/j.jstrokecerebrovasdis.2025.108241

[B31] Tu W. J. Wang L. D. Special Writing Group of China Stroke Surveillance Report (2023b). China stroke surveillance report 2021. Mil. Med. Res. 10 (1), 33. 10.1186/s40779-023-00463-x 37468952 PMC10355019

[B32] Tu W. J. Zhao Z. Yin P. Cao L. Zeng J. Chen H. (2023a). Estimated burden of stroke in China in 2020. JAMA Netw. Open 6 (3), e231455. 10.1001/jamanetworkopen.2023.1455 36862407 PMC9982699

[B33] von Elm E. Altman D. G. Egger M. Pocock S. J. Gøtzsche P. C. Vandenbroucke J. P. (2007). The strengthening the reporting of observational studies in epidemiology (STROBE) statement: guidelines for reporting observational studies. Ann. Intern Med. 147 (8), 573–577. 10.7326/0003-4819-147-8-200710160-00010 17938396

[B34] von Elm E. Altman D. G. Egger M. Pocock S. J. Gøtzsche P. C. Vandenbroucke J. P. (2008). The strengthening the reporting of observational studies in epidemiology (STROBE) statement: guidelines for reporting observational studies. J. Clin. Epidemiol. 61 (4), 344–349. 10.1016/j.jclinepi.2007.11.008 18313558

[B35] Vos T. Lim S. S. Abbafati C. (2020). Global burden of 369 diseases and injuries in 204 countries and territories, 1990–2019: a systematic analysis for the Global Burden of Disease Study 2019. Lancet 396 (10258), 1204–1222. 10.1016/S0140-6736(20)30925-9 33069326 PMC7567026

