# Supplementary material for: Elderly stroke burden: a comprehensive global study over three decades
Source: Front Aging. 2025 Jul 15;6:1489914. doi: 10.3389/fragi.2025.1489914 (PMC12303880; doi:10.3389/fragi.2025.1489914)
Supplement: Supplementary file 1 [file Table1.docx]

**Supplementary Table 1-1**

| sex_name | age_name | metric_name | Incidence | | Deaths | | DALYs  (Disability-Adjusted Life Years) | |
| --- | --- | --- | --- | --- | --- | --- | --- | --- |
|  |  |  | 1990 | 2021 | 1990 | 2021 | 1990 | 2021 |
| Male | 60-64 years | Number | 421803.034310969(327479.638581487 to 530640.934737546) | 643500.353810842(509412.534928795 to 800918.835792648) | 271531.651764207(249722.338136859 to 293881.823254777) | 329851.204 (301454.9675 to 363621.2108) | 8285744.31707868(7630948.93111481 to 8935725.69686493) | 10325897.071272(9424364.22629047 to 11351573.0821895) |
| Female | 60-64 years | Number | 321399.359947658(255197.407064162 to 406862.487551172) | 473094.037534567(379207.01255391 to 594564.85101807) | 203798.320479114(188401.692192109 to 221641.798804082) | 217320.4387 (198925.4595 to 237253.6441) | 6332652.15678644(5861540.62974025 to 6835184.927159) | 7055637.98404001(6487986.19579725 to 7650063.84304941) |
| Male | 60-64 years | Percent | 0.0014514590403999(0.0010917093979468 to 0.0018911628281877) | 0.0010595582109929(0.0008025024408616 to 0.0013544903175835) | 0.140816110663817(0.133072100746682 to 0.14722963067844) | 0.103518599 (0.097832568 to 0.109678714) | 0.118827529421221(0.110057321506145 to 0.12625190153411) | 0.0861604698044783(0.0795233669138316 to 0.0927541563086832) |
| Female | 60-64 years | Percent | 0.0010080532736374(0.0007507456979972 to 0.0013194692205651) | 0.0006898861984989(0.0005286007293622 to 0.0008941328771005) | 0.164928887131304(0.155952554718893 to 0.175239380807671) | 0.11028534 (0.10336995 to 0.116797996) | 0.122090062688239(0.110248194919059 to 0.133095224754171) | 0.0783025828491953(0.069634385447931 to 0.0862222029438977) |
| Male | 60-64 years | Rate | 537.01327888107(416.92662256142 to 675.57889605375) | 413.72836258585(327.51872273057 to 514.93808283758) | 345.697140069386(317.930884095671 to 374.151982494384) | 212.0726084 (193.8156978 to 233.7844995) | 10548.8921646881(9715.24757560384 to 11376.4078617737) | 6638.87201029149(6059.24574347946 to 7298.31415982169) |
| Female | 60-64 years | Rate | 391.64892661723(310.97694335315 to 495.79207798107) | 287.57604679229(230.50566046967 to 361.41357922934) | 248.343349143693(229.581417125185 to 270.086949175223) | 132.1009095 (120.9192945 to 144.2175543) | 7716.8057218585(7142.72143023235 to 8329.17912589531) | 4288.85658678285(3943.80244476038 to 4650.18567800512) |
| Male | 65-69 years | Number | 446384.702058124(334954.793305765 to 594096.310384821) | 804714.019720192(596448.64295997 to 1071506.75236434) | 322393.809099131(295495.51789262 to 350368.741267766) | 467431.8995 (422841.6102 to 524251.4745) | 8269483.88025883(7611768.98984323 to 8926689.26842213) | 12313537.3621697(11168359.1107194 to 13668696.3311448) |
| Female | 65-69 years | Number | 380663.03134821(285531.60644426 to 512176.559477563) | 629935.322293767(476086.853738469 to 846633.54002156) | 275540.685285011(255662.286734191 to 297881.284517369) | 336988.9228 (306932.726 to 370858.4279) | 7165495.05202756(6676590.10703591 to 7722818.59920276) | 9129088.37251604(8342306.32658032 to 10006795.9786392) |
| Male | 65-69 years | Percent | 0.002013954351649(0.0014917585212133 to 0.0027070691760582) | 0.0015364517733743(0.0011401875153043 to 0.0020535516855636) | 0.154765999597336(0.145888029930616 to 0.162239213789535) | 0.1177801 (0.110724486 to 0.125777024) | 0.132405008043474(0.122919789075701 to 0.140643152832181) | 0.0996450044344662(0.0915352398672122 to 0.107602457102479) |
| Female | 65-69 years | Percent | 0.0014264548495267(0.0010366182243054 to 0.0020280985802564) | 0.0010335989217665(0.0007662918203715 to 0.001457751578817) | 0.180524206804324(0.171026249287968 to 0.193964503713508) | 0.12768915 (0.119583411 to 0.136426459) | 0.138556926091302(0.126281688714206 to 0.151238887245057) | 0.0945346185072299(0.0852128157756416 to 0.104423453460336) |
| Male | 65-69 years | Rate | 778.60671763909(584.24504910413 to 1036.2527569997) | 610.40299158597(452.42661003564 to 812.77436594828) | 562.33554674361(515.418190190304 to 611.130834780073) | 354.563016 (320.7397628 to 397.6625988) | 14424.0509831346(13276.8314892591 to 15570.3817774769) | 9340.23747403775(8471.5807668668 to 10368.1717071516) |
| Female | 65-69 years | Rate | 574.34087858124(430.80746014944 to 772.76727954698) | 437.42798265882(330.59538754375 to 587.90353288083) | 415.733250247538(385.740905448438 to 449.440540776118) | 234.0055867 (213.1345209 to 257.5246191) | 10811.2329928001(10073.5777110285 to 11652.1176319124) | 6339.25193425109(5792.90936388583 to 6948.73334277534) |
| Male | 70-74 years | Number | 417561.212876517(327553.764296887 to 519647.619750966) | 871144.565914304(688817.06710483 to 1077527.98285134) | 352541.630626727(324020.473571304 to 382247.528683388) | 562423.4267 (506562.0234 to 627615.7379) | 7432630.41482917(6849451.83572971 to 8037746.53337095) | 12206601.3907289(11008835.9972339 to 13474451.7041228) |
| Female | 70-74 years | Number | 399626.050303278(310599.668619409 to 502108.324455717) | 690904.894996222(542727.218904623 to 866864.158804082) | 349018.578883135(322670.965745834 to 377407.876305526) | 440120.4806 (398531.1555 to 482169.8671) | 7398768.04900196(6864817.85429039 to 8010199.73610913) | 9739641.96286188(8856143.29117563 to 10597736.4934042) |
| Male | 70-74 years | Percent | 0.0027346531491024(0.0021283925060652 to 0.0034808516403985) | 0.0021774880109866(0.0016880741541206 to 0.0027731136604896) | 0.168955074688383(0.159359597380507 to 0.176924710705829) | 0.129148255 (0.120821904 to 0.137450251) | 0.146758513783362(0.13744812010354 to 0.155032013474542) | 0.111009249287114(0.101989627756516 to 0.119926361027503) |
| Female | 70-74 years | Percent | 0.0020269264899102(0.0015425932084474 to 0.0026348259743223) | 0.001439029799066(0.0011105162819799 to 0.0018672201551559) | 0.19736618225516(0.186910289538971 to 0.210890301619941) | 0.139257471 (0.129029006 to 0.148021574) | 0.157426070382255(0.144025289622589 to 0.170958464517067) | 0.107104132858549(0.0969003108613149 to 0.117319926517225) |
| Male | 70-74 years | Rate | 1109.98863123605(870.72492194249 to 1381.36142051829) | 903.75744586184(714.60418579245 to 1117.8672010705) | 937.149308809987(861.332496555974 to 1016.11547737819) | 583.4787697 (525.5260933 to 651.1116736) | 19757.9061613644(18207.6625735524 to 21366.4655568408) | 12663.5776967287(11420.9717790409 to 13978.8922906567) |
| Female | 70-74 years | Rate | 849.49746383732(660.25132886272 to 1067.34720590171) | 631.26425931744(495.87765021569 to 792.03428011505) | 741.919595502652(685.911658779513 to 802.267603702173) | 402.1281817 (364.128951 to 440.5477602) | 15727.7902388443(14592.7557838114 to 17027.5295003692) | 8898.89174940173(8091.65888909174 to 9682.9134174637) |
| Male | 75-79 years | Number | 377943.751891494(294014.396048567 to 476643.005125027) | 782048.476861852(624669.187633614 to 974644.853951177) | 374919.324608706(348726.338784575 to 397735.108411773) | 558093.0734 (500878.5969 to 619546.0077) | 6334885.7959009(5908136.77494432 to 6739560.83330823) | 9725144.8297127(8758026.05895296 to 10736574.9190232) |
| Female | 75-79 years | Number | 439721.357853065(344087.64172155 to 555788.67616606) | 643411.008742004(509053.76386156 to 810132.25766967) | 461052.579293472(428675.168689779 to 493118.366042628) | 502344.6661 (447718.1265 to 551519.9925) | 7751640.44882231(7222111.25886287 to 8289884.517585) | 8782948.3747519(7887262.56402157 to 9611212.78235184) |
| Male | 75-79 years | Percent | 0.0035730219097334(0.0027528136190826 to 0.0045832345496612) | 0.0030307918107416(0.0023801108889116 to 0.0038064988265541) | 0.178087790834334(0.168187609327581 to 0.185408481201014) | 0.137391863 (0.127857034 to 0.146271032) | 0.156651083843528(0.147107250299921 to 0.164576256662) | 0.119984778430978(0.110359681435315 to 0.128970777921557) |
| Female | 75-79 years | Percent | 0.0028278331319759(0.0021989663166546 to 0.003611059531742) | 0.00197213334586(0.001563884114875 to 0.002551789101354) | 0.215624583768938(0.202737914396075 to 0.229420187665134) | 0.153401497 (0.13951988 to 0.163394021) | 0.17552093217843(0.161047223661202 to 0.188501786542816) | 0.120792470552791(0.109290952164163 to 0.130845964739336) |
| Male | 75-79 years | Rate | 1497.82539375698(1165.20573849333 to 1888.97949300634) | 1308.06378961748(1044.82927722349 to 1630.20283129554) | 1485.83931391559(1382.03413362261 to 1576.26033605889) | 933.4732592 (837.7756303 to 1036.260184) | 25105.7274109269(23414.4823059326 to 26709.4913155153) | 16266.3954689602(14648.7808559039 to 17958.1257321092) |
| Female | 75-79 years | Rate | 1210.59712163795(947.30788309466 to 1530.14212202635) | 892.41567302722(706.06121299008 to 1123.65923825721) | 1269.32411957788(1180.18585193881 to 1357.6044554049) | 696.7556464 (620.9882451 to 764.962176) | 21341.0457502779(19883.1986347362 to 22822.8858036341) | 12182.0122418211(10939.6896132938 to 13330.8209017755) |
| Male | 80-84 | Number | 253646.693764575(203444.29362754 to 308323.452604072) | 590184.231968667(468909.686153153 to 716880.900978548) | 291017.301793574(269222.468251709 to 308100.54980124) | 537634.6065 (476774.3472 to 593971.1289) | 3859911.54913844(3570180.02483237 to 4087509.15953823) | 7319353.45451729(6554597.49247288 to 8027578.20888151) |
| Female | 80-84 | Number | 391169.458034572(315262.88808373 to 470829.459587938) | 672086.511878797(544967.346466925 to 807708.344554542) | 450448.742416324(409210.179221082 to 482050.064682771) | 602669.7341 (518909.2195 to 669091.5803) | 5906220.47389636(5383336.35995681 to 6332600.12485488) | 8163696.94714322(7069323.2169231 to 8953976.03255442) |
| Male | 80-84 | Percent | 0.0042043284865951(0.00332487839474 to 0.0052154405566677) | 0.0034898093215424(0.0027687212988137 to 0.0043152107453608) | 0.175178872978487(0.162778706079663 to 0.182962984850197) | 0.136341425 (0.124633158 to 0.145865922) | 0.155371163258076(0.145077079158311 to 0.163477671991401) | 0.121292010899155(0.11017597863944 to 0.130965843291298) |
| Female | 80-84 | Percent | 0.0038747110162999(0.0030671179315171 to 0.0047186297136871) | 0.0027583120613778(0.002224596251478 to 0.0033691645559708) | 0.215809616639378(0.195389813841287 to 0.231073968172569) | 0.152626134 (0.132704724 to 0.165107744) | 0.179311084455418(0.160993593857151 to 0.193250584841721) | 0.124895533833298(0.109131371120834 to 0.136039330818789) |
| Male | 80-84 | Rate | 1909.49558945429(1531.56335537337 to 2321.1115592906) | 1610.24350850942(1279.3611508167 to 1955.91944794007) | 2190.82790310485(2026.75267743008 to 2319.43350895781) | 1466.868459 (1300.81889 to 1620.575581) | 29058.0727443054(26876.9243935139 to 30771.4663895311) | 19969.9360779402(17883.3955423379 to 21902.2383176605) |
| Female | 80-84 | Rate | 1770.59942645105(1427.01398935144 to 2131.17449222987) | 1319.59698906081(1070.00699586112 to 1585.88140168729) | 2038.92269344161(1852.25940764206 to 2181.96372573795) | 1183.301781 (1018.843603 to 1313.716641) | 26734.0672150589(24367.2712057449 to 28664.0429581314) | 16028.8738438451(13880.1440988177 to 17580.5340590027) |
| Male | 85-89 | Number | 120881.393344853(101075.18445661 to 142068.147826056) | 316669.302577149(267659.526912944 to 368876.221337968) | 168196.946120365(153415.853628375 to 177544.820114442) | 434865.8356 (389340.6811 to 474889.7695) | 1756828.79161441(1608356.8409134 to 1854441.68747532) | 4599985.24684662(4120700.39872351 to 4983401.74443614) |
| Female | 85-89 | Number | 251404.824083416(205900.093964732 to 298939.516323646) | 532920.858739637(445701.98224569 to 630980.338411356) | 320631.352026734(274976.378488219 to 344477.460770359) | 518223.1512 (427274.5697 to 583891.7751) | 3323945.88154242(2878730.05771676 to 3577291.94038858) | 5555828.92496343(4634198.7589974 to 6223396.30613586) |
| Male | 85-89 | Percent | 0.0048181557388222(0.0040035898739842 to 0.0057543240517988) | 0.0036814244295273(0.0030717557347922 to 0.0043592842945299) | 0.174094107015742(0.159268534129561 to 0.183636802771969) | 0.14028242 (0.126080352 to 0.150129455) | 0.15521380001553(0.141739525514439 to 0.164550093729922) | 0.125969285208127(0.112869054847815 to 0.135750557669837) |
| Female | 85-89 | Percent | 0.0051263753638636(0.0041423157655999 to 0.0062311771277616) | 0.0037029667612438(0.0030646483428774 to 0.0044630216664981) | 0.209990777815384(0.179642141627145 to 0.224971444012649) | 0.148541468 (0.122251374 to 0.162903311) | 0.17824800144639(0.154036572066491 to 0.192752538454337) | 0.124514294331057(0.104631406992614 to 0.137454293944191) |
| Male | 85-89 | Rate | 2387.04472393325(1995.9315416677 to 2805.4195383048) | 1835.48108979636(1551.41024454955 to 2138.08324088006) | 3321.38488570292(3029.50267065997 to 3505.97734182971) | 2520.57276 (2256.699503 to 2752.559798) | 34692.0959614814(31760.2205357752 to 36619.657694559) | 26662.4704864541(23884.4358989634 to 28884.8321903331) |
| Female | 85-89 | Rate | 2502.28454543467(2049.36649449712 to 2975.40723191603) | 1871.92523852566(1565.56227016484 to 2216.36665391388) | 3191.31058794685(2736.8971329777 to 3428.65587197421) | 1820.298418 (1500.834575 to 2050.964477) | 33083.9252570791(28652.5994883567 to 35605.5313161863) | 19515.2736377523(16277.9772550613 to 21860.1550750985) |
| Male | 90-94 | Number | 35382.6583877011(28875.9023967482 to 42901.3031456862) | 119270.982420305(98541.4536164038 to 143446.650372936) | 55068.2238564893(49013.3479178717 to 58632.3363382732) | 185924.4659 (160807.1081 to 204103.653) | 495559.763948534(444246.542968223 to 526754.384730192) | 1701724.9621078(1484675.18147572 to 1860478.78193498) |
| Female | 90-94 | Number | 92845.7450906635(74649.3209108877 to 113993.936773571) | 282423.222234213(232237.365373688 to 343015.260768311) | 132402.13053703(110221.417449736 to 144188.442570735) | 316963.555 (248663.5106 to 359580.161) | 1188173.59028118(992615.276568697 to 1294183.08653791) | 2930830.85927952(2340601.27053697 to 3307482.23472163) |
| Male | 90-94 | Percent | 0.0052329182764656(0.0041423414323709 to 0.0064314223629345) | 0.0038143498884749(0.0030567739748036 to 0.0046200343524502) | 0.158203838619226(0.140599964370894 to 0.167942013966092) | 0.118346178 (0.102030126 to 0.127584563) | 0.143037879357325(0.127579057144148 to 0.152106335557798) | 0.108502888079298(0.0939994756059294 to 0.117252467718981) |
| Female | 90-94 | Percent | 0.0059165549782183(0.0046586924513968 to 0.0073999221791527) | 0.0043630457079158(0.0034990073370487 to 0.0053492202961786) | 0.191033141158878(0.158979559652254 to 0.207604159395942) | 0.132677531 (0.104028542 to 0.147974639) | 0.166503757787464(0.137553645178756 to 0.182497946507612) | 0.115922828620099(0.0920125196874714 to 0.129621128641208) |
| Male | 90-94 | Rate | 2810.46595660609(2293.63039269401 to 3407.67645731502) | 2046.34129669066(1690.68319786762 to 2461.12506641104) | 4374.10232842265(3893.159870383 to 4657.20193857094) | 3189.92017 (2758.979757 to 3501.821864) | 39362.6117851344(35286.771606345 to 41840.4193815969) | 29196.6243163743(25472.6847584701 to 31920.3756507512) |
| Female | 90-94 | Rate | 3068.03639791746(2466.74560488238 to 3766.86671878931) | 2341.64795063826(1925.54332602982 to 2844.03306520473) | 4375.15532082554(3642.20590007252 to 4764.62749622041) | 2628.031269 (2061.736975 to 2981.37717) | 39262.5404477852(32800.423914611 to 42765.5657369136) | 24300.3178740303(19406.563401748 to 27423.2371383594) |
| Male | 95+ years | Number | 8270.46697907688(6339.55377201999 to 10651.1609875472) | 34062.7731254216(26937.3405293318 to 42748.1186786439) | 11984.4557693886(9998.86581944079 to 13042.2283785469) | 45031.15996 (35787.92493 to 49592.89734) | 102123.170700934(86110.8957361965 to 111009.849633832) | 392589.613163717(319727.422800782 to 431908.379109608) |
| Female | 95+ years | Number | 26785.2347921467(20026.848381222 to 34517.4950599438) | 103867.954654015(80801.6067922067 to 128903.820124965) | 41746.0534944704(32019.2188066416 to 46364.0965574988) | 139035.4924 (101046.9111 to 160279.8332) | 352835.783617505(275470.903395792 to 390756.148814241) | 1196193.77875662(897948.291991262 to 1374163.67003398) |
| Male | 95+ years | Percent | 0.0054991007807375(0.0040801142241766 to 0.0071236810152723) | 0.0039255156455981(0.0029989022918721 to 0.0049841779267411) | 0.137818812879649(0.115016509510092 to 0.149925462722858) | 0.092796313 (0.074161538 to 0.102002417) | 0.125646937861138(0.105536870756652 to 0.13651498640673) | 0.0865567860330017(0.0706366708382145 to 0.0948660326058848) |
| Female | 95+ years | Percent | 0.0064769881108364(0.0046633701500623 to 0.0083260017329654) | 0.0045901845912437(0.0034793119860481 to 0.0058016453708396) | 0.172131376217247(0.132053376102989 to 0.191642771071137) | 0.121275592 (0.088309548 to 0.138655425) | 0.154004204090428(0.119844642372756 to 0.171432739014808) | 0.10941747866446(0.082053593080775 to 0.125008020157838) |
| Male | 95+ years | Rate | 3178.12284418252(2436.12370568089 to 4092.96090985273) | 2252.7665097403(1781.52079346293 to 2827.178214735) | 4605.3110123221(3842.30104020408 to 5011.78519348033) | 2978.168826 (2366.860691 to 3279.862677) | 39243.2473941412(33090.1514466565 to 42658.1642781587) | 25964.2023082106(21145.4078527045 to 28564.5777621138) |
| Female | 95+ years | Rate | 3534.34676760271(2642.56884028488 to 4554.62862422336) | 2637.40269790571(2051.70474819438 to 3273.11040349626) | 5508.44636507016(4224.977804697 to 6117.80318792878) | 3530.372616 (2565.771096 to 4069.80638) | 46557.143179821(36348.746036416 to 51560.7849697632) | 30373.6095489658(22800.5957734148 to 34892.5997703905) |

**Supplementary Table 1-2**

| age_name | year | Incidence | Death | DALYs |
| --- | --- | --- | --- | --- |
| 95+ years | 1990 | 35055.7017712236(26754.4300069332 to 44515.7795885342) | 475329.972243321(448160.278427211 to 504541.685687003) | 14618396.4738651(13792298.5409232 to 15500919.3418656) |
| 95+ years | 1991 | 36076.1564009226(27762.3280273242 to 45562.4619057423) | 597934.494384143(561644.914347619 to 634217.583910102) | 15434978.9322864(14495043.6200462 to 16372451.7531761) |
| 95+ years | 1992 | 37321.4389238948(28934.0158130491 to 46780.4410925945) | 701560.209509863(661012.346019941 to 741858.210365394) | 14831398.4638311(13950431.0519613 to 15698767.3139447) |
| 95+ years | 1993 | 38492.5692473807(30057.5235704756 to 47892.0426043531) | 835971.903902178(785017.908560969 to 880913.099179186) | 14086526.2447232(13274068.9907331 to 14820577.5134378) |
| 95+ years | 1994 | 39935.7681819987(31389.0081764881 to 49423.5843584098) | 741466.044209899(678177.803200839 to 783911.894833995) | 9766132.02303481(8985448.46201642 to 10318221.6957129) |
| 95+ years | 1995 | 41720.9385226312(33003.2403201773 to 51429.0546499554) | 488828.298147099(429631.129133011 to 519310.325358212) | 5080774.67315684(4509170.42718267 to 5399262.05832804) |
| 95+ years | 1996 | 44257.9493282141(35094.5942608243 to 54503.5636562422) | 187470.35439352(158426.222515546 to 201834.063125723) | 1683733.35422971(1436728.05838507 to 1813574.86887287) |
| 95+ years | 1997 | 46320.1851071636(36824.1315101198 to 56934.7080999495) | 53730.509263859(42074.3917075354 to 59252.4779689945) | 454958.954318439(362033.448627622 to 500543.117230366) |
| 95+ years | 1998 | 48844.2638407869(38886.7031949744 to 59866.132925881) | 490201.802549892(464020.274738922 to 518826.074945188) | 14816452.1016286(14071772.4296083 to 15633000.3313776) |
| 95+ years | 1999 | 51506.4543149742(41031.1857246824 to 63054.9683649078) | 628378.030848487(594217.610734575 to 667156.535619529) | 15768802.5564145(14925625.0755948 to 16626444.590711) |
| 95+ years | 2000 | 54356.4324756235(43309.480523255 to 66414.5214076269) | 737118.997781743(696525.460410219 to 782768.174436026) | 15102407.0529843(14293682.9074134 to 15930097.6768033) |
| 95+ years | 2001 | 57154.9949448414(45640.9666296237 to 69811.9719686815) | 824755.51049974(778380.440241656 to 870250.374687711) | 13980323.0221018(13239848.1824316 to 14660377.7425262) |
| 95+ years | 2002 | 59701.4922556871(47760.2925488863 to 72931.1692225286) | 767318.88920888(697206.978083228 to 811330.944672275) | 9930354.64890291(9104248.8771986 to 10468544.5379365) |
| 95+ years | 2003 | 61565.6978676019(49317.761910201 to 75199.3305995815) | 520666.4449541(458637.221631963 to 554322.27840914) | 5220992.8996026(4595062.10244242 to 5549500.81242802) |
| 95+ years | 2004 | 63509.8974152785(50870.4846615868 to 77531.4429768461) | 206214.361347746(173993.214580325 to 222443.074144226) | 1770021.44771866(1508788.50374166 to 1906699.86952696) |
| 95+ years | 2005 | 65660.2387178877(52590.8126097744 to 80091.3369751839) | 58212.0270963703(45519.1437245708 to 64126.8515930841) | 471465.078166005(374879.300214895 to 518540.577217669) |
| 95+ years | 2006 | 68304.7511513786(54876.6068598584 to 83202.8572010633) | 481796.060627419(457874.337328967 to 508983.22161765) | 15375410.5817652(14645641.8831982 to 16165096.1888507) |
| 95+ years | 2007 | 70889.2375899484(57192.7671519573 to 86124.0391125692) | 610410.815687573(578351.547275735 to 645091.12561455) | 16909441.7384816(16112431.3853302 to 17768414.0238923) |
| 95+ years | 2008 | 73667.7730608029(59672.0592614265 to 89146.683927879) | 712925.093868665(675711.623966637 to 751867.475703691) | 16334282.3094193(15524350.5132854 to 17142516.5089904) |
| 95+ years | 2009 | 76607.9770778724(62216.0472537608 to 92473.4661334776) | 829913.011772378(784928.768690033 to 870685.660230675) | 13842292.8352235(13112026.9555891 to 14528972.5627773) |
| 95+ years | 2010 | 79705.5199091232(64843.7004590302 to 96188.8776526614) | 753134.863376663(686311.391893471 to 795762.664465603) | 10463785.8868046(9587415.28802039 to 11018082.2817033) |
| 95+ years | 2011 | 82268.5101185131(67150.0925361064 to 99314.6933955767) | 501981.897183207(440098.910926116 to 533532.955727449) | 5627869.64852067(4981907.25608296 to 5975177.2155623) |
| 95+ years | 2012 | 84642.911016066(69192.34478205 to 101935.970503914) | 196920.119350988(166334.758579792 to 212633.486430615) | 1976239.66461174(1678772.24766859 to 2127027.41881366) |
| 95+ years | 2013 | 87100.5673065814(71362.4672655822 to 104654.44019215) | 55662.2825447888(43613.3631993469 to 61325.4281480605) | 518266.603890085(413134.078073315 to 569805.170798829) |
| 95+ years | 2014 | 90723.4743323582(74501.5364379588 to 108827.660746434) | 505815.344703987(484320.357424504 to 529808.703268865) | 15063491.8422195(14271926.9802378 to 15944170.3019061) |
| 95+ years | 2015 | 96332.9783641662(79246.3105787761 to 115402.77083907) | 678030.417156819(650002.915257661 to 709832.834544515) | 16236449.7708638(15363615.8197322 to 17239553.45825) |
| 95+ years | 2016 | 103377.850476562(85072.7277251954 to 123756.344947706) | 801571.337288501(765061.429713237 to 838976.37018782) | 15626422.6289776(14792228.6642276 to 16517023.9482689) |
| 95+ years | 2017 | 111198.562111222(91431.7768688066 to 133134.371304514) | 820012.476839295(774692.990762624 to 863459.397152939) | 13887321.1081095(13092368.3363333 to 14631971.7649921) |
| 95+ years | 2018 | 119471.585006986(98070.5137039786 to 143121.57945463) | 812316.475317873(741436.186046824 to 853745.6474411) | 10123413.32362(9270924.08296079 to 10684444.2693523) |
| 95+ years | 2019 | 127921.168918077(104808.36720672 to 153520.611517608) | 558104.229625536(489545.845953879 to 591858.278797932) | 5414898.56123759(4765248.78692902 to 5766996.61091846) |
| 95+ years | 2020 | 131350.643607604(106131.656436726 to 160194.572410126) | 232174.304311782(195762.713749233 to 249844.652347753) | 1854330.76787914(1579296.48874059 to 1998585.90405619) |
| 95+ years | 2021 | 137930.727779437(111259.87565984 to 168710.378248073) | 63989.972498257(50164.0820353582 to 70362.2398612054) | 493138.430134872(392028.758864409 to 542852.489929858) |
| 90-94 | 1990 | 128228.403478365(107224.530022073 to 153190.689974543) | 504776.705671703(481858.218358833 to 527262.815521316) | 15499087.5796574(14786399.998857 to 16194679.4019084) |
| 90-94 | 1991 | 136414.680099558(114377.317984436 to 162546.227130024) | 687815.034841596(656742.628413252 to 716603.761202137) | 17750954.5827458(16908607.9178514 to 18491428.7256609) |
| 90-94 | 1992 | 143297.650706539(119964.840225396 to 170541.901430639) | 826334.650839553(786538.416404627 to 862191.825229581) | 17517521.0823179(16673455.8116104 to 18278722.3654425) |
| 90-94 | 1993 | 151639.320188543(127054.276338833 to 179957.013891696) | 824554.713205597(776158.739459942 to 862544.101051534) | 13951249.0645115(13122415.4970256 to 14600772.1374773) |
| 90-94 | 1994 | 160128.03217561(134827.829597888 to 189620.109282381) | 824062.579219429(750710.134215132 to 866130.147646873) | 10850910.7228484(9934165.0167682 to 11405690.0912439) |
| 90-94 | 1995 | 168394.247840397(142341.291426288 to 198682.130301169) | 573006.211845137(503768.434863976 to 606998.388918982) | 5959342.11767517(5281264.54471386 to 6322775.98654397) |
| 90-94 | 1996 | 175524.759028271(148528.808963682 to 206652.886487734) | 244615.907909539(206838.095844542 to 263383.622910549) | 2200021.56681525(1877134.53948014 to 2367438.23897797) |
| 90-94 | 1997 | 182865.842677202(154902.95746662 to 215001.057192133) | 66807.4413101513(52234.2828604423 to 73769.1180219741) | 566044.338477895(449008.717569904 to 623484.363184746) |
| 90-94 | 1998 | 188282.997701033(159623.373984704 to 221013.344338235) | 498886.798162455(477677.110099682 to 519234.124808901) | 15520428.2631269(14829289.4860814 to 16246788.3828675) |
| 90-94 | 1999 | 193335.482843963(163932.958543681 to 226410.552667168) | 692591.502253323(663566.274742707 to 719472.257346869) | 17499112.0974906(16746157.9482471 to 18284969.4220665) |
| 90-94 | 2000 | 197463.544591371(167329.575946766 to 230676.92613523) | 855828.345791324(819733.486932029 to 889256.976210852) | 16988582.1840449(16230927.6606449 to 17794056.4022791) |
| 90-94 | 2001 | 201968.68316899(171202.128200508 to 235576.731188904) | 852244.52893933(805569.83028108 to 889698.254343329) | 13832549.3443717(13090281.5295128 to 14561916.010434) |
| 90-94 | 2002 | 205115.913475349(173917.898690542 to 239013.084311142) | 797924.75800974(727345.961742633 to 837781.629153378) | 10705881.4335411(9851518.2734968 to 11262043.9869615) |
| 90-94 | 2003 | 208784.286526736(177077.6803462 to 243368.062198668) | 590421.940094722(519124.630016014 to 625296.725662896) | 5803036.50346071(5136180.9535952 to 6156911.37990245) |
| 90-94 | 2004 | 212072.708628072(179854.711964689 to 247285.441427841) | 260766.626717982(218615.87408563 to 280781.715792303) | 2087951.81351208(1776814.0364441 to 2250887.77552637) |
| 90-94 | 2005 | 215546.739614655(182652.747228964 to 251423.516684599) | 71391.8353742331(55297.6409854448 to 79003.0087291237) | 542165.477290577(431502.791332428 to 596079.453370367) |
| 90-94 | 2006 | 217136.969912424(183825.256340355 to 253201.352838779) | 499055.304188886(477248.041033489 to 523737.235072166) | 15401561.2582641(14697840.2983449 to 16127958.6270179) |
| 90-94 | 2007 | 217961.24698699(184322.016520533 to 253992.411666346) | 692352.504317141(665129.154254846 to 721421.849411213) | 17856612.1506946(17054672.7229244 to 18688716.743539) |
| 90-94 | 2008 | 218706.347663262(184759.894688119 to 254839.153937625) | 876924.209221231(842205.005770926 to 915769.40141203) | 17829093.4919035(16987772.0757402 to 18569698.6961111) |
| 90-94 | 2009 | 223368.980529541(188585.461389201 to 260338.521731553) | 881972.319602887(833675.261434344 to 921751.118784454) | 14150050.6086615(13383438.6887619 to 14834948.9331289) |
| 90-94 | 2010 | 233263.535402017(197486.26547453 to 272006.006116751) | 785909.928993452(716314.813148878 to 829032.937569588) | 10709892.6621528(9809443.88055109 to 11274708.1039593) |
| 90-94 | 2011 | 246276.840584869(208870.45110448 to 286769.197197976) | 606289.477125785(530156.023386985 to 642452.654899852) | 6063485.58891857(5360931.06940058 to 6432715.33577127) |
| 90-94 | 2012 | 259821.813842999(220729.668716265 to 302328.590413662) | 268538.783214501(225796.705453499 to 289338.652927989) | 2265759.67562554(1921948.27355673 to 2439694.89100845) |
| 90-94 | 2013 | 273760.103962755(232878.102597772 to 318530.508515281) | 74471.1260317624(57537.9159618737 to 82438.5029873868) | 593640.208321939(470844.914642199 to 654276.986845024) |
| 90-94 | 2014 | 287706.458164617(244905.341896104 to 334037.971891657) | 498753.321474753(477880.810670926 to 521045.354959233) | 15364885.7389688(14715100.1446461 to 15992390.7705778) |
| 90-94 | 2015 | 303343.210799719(258147.832182389 to 351598.71475436) | 689138.520222882(660322.826660054 to 721343.378271585) | 17875394.3450217(17091296.4204616 to 18610684.0146781) |
| 90-94 | 2016 | 321748.073447236(273904.188471749 to 373626.496058651) | 898530.527665079(861365.847779999 to 937662.869813852) | 18168922.6848947(17388895.8807948 to 18909295.8180403) |
| 90-94 | 2017 | 343348.587232022(292276.802166986 to 399645.270049132) | 916052.378204574(866737.342091923 to 960444.749113821) | 14481135.6046513(13692891.6239883 to 15147721.4519475) |
| 90-94 | 2018 | 365901.082404486(311518.420613927 to 426870.570010182) | 786184.394335578(714067.25291654 to 829339.156314448) | 10508672.6509808(9622873.65111548 to 11057397.6200168) |
| 90-94 | 2019 | 387586.895339742(330071.235767378 to 452701.357191204) | 625169.27704531(551629.250469807 to 661491.395888071) | 6151616.53126756(5458217.12778912 to 6524771.19788535) |
| 90-94 | 2020 | 389191.094471858(329810.594157269 to 459334.1155046) | 278746.313451985(234383.87352768 to 300265.539279687) | 2349002.04585715(1990527.39915666 to 2532287.8681653) |
| 90-94 | 2021 | 401694.204654518(339186.641036662 to 474188.213031913) | 79460.2684550795(61446.9001059747 to 87928.1763004511) | 605022.739511571(477173.952028005 to 668886.387593026) |
| 85-89 | 1990 | 372286.21742827(312741.368862022 to 432659.978786035) | 497528.935018368(478364.654888284 to 516825.168887038) | 15384779.8200607(14712857.1533284 to 16039682.838794) |
| 85-89 | 1991 | 387639.534513392(327151.22070064 to 448462.483875301) | 699405.570225545(671238.685278127 to 727134.899303977) | 17870105.8218259(17129020.8093227 to 18629812.7494349) |
| 85-89 | 1992 | 404241.179741694(342947.149904043 to 466242.542353875) | 934690.534645108(898212.950642121 to 974385.054521378) | 18618329.6194629(17882418.5134874 to 19382398.1336161) |
| 85-89 | 1993 | 416774.505639905(355682.207161019 to 480000.999427797) | 981854.037816857(930895.171238916 to 1028950.21852275) | 14989411.7224532(14223010.7496347 to 15700664.637095) |
| 85-89 | 1994 | 428770.099877932(367164.171662176 to 492553.767520382) | 819262.060671787(745409.414126445 to 863008.320589036) | 10361228.3142918(9527115.43425446 to 10938505.3505079) |
| 85-89 | 1995 | 439370.268164048(376254.143998679 to 504458.759097704) | 657712.522680489(582502.066436823 to 694687.923575386) | 6317668.0523708(5597186.01479033 to 6702577.39107909) |
| 85-89 | 1996 | 451083.560116393(386457.661687376 to 517517.637899037) | 297449.011575531(250167.079828202 to 320451.454340452) | 2420782.79682843(2054849.69644846 to 2614055.94593694) |
| 85-89 | 1997 | 460404.051613634(394527.42162966 to 527941.483330849) | 85808.9248110724(66032.2420308863 to 95407.3828572332) | 631415.597418288(496837.450738844 to 697643.970323965) |
| 85-89 | 1998 | 470412.715112508(403113.671013841 to 539422.274859156) | 500431.283067771(479853.112971869 to 521466.693822827) | 15385878.5352744(14757107.8941831 to 16129830.4712119) |
| 85-89 | 1999 | 477857.489997492(409333.138608084 to 547779.453136939) | 693551.710704394(664534.063331975 to 719991.62152489) | 17797929.9638844(17111757.8107334 to 18610891.1214682) |
| 85-89 | 2000 | 483494.949731399(413865.881873747 to 554487.473425941) | 916966.183622226(878887.226471476 to 951594.081942453) | 19075717.0979575(18339192.2654095 to 19885553.4132598) |
| 85-89 | 2001 | 483067.574730231(413312.823069085 to 554139.47583047) | 950674.974089746(899896.636878142 to 989849.464801331) | 15570651.6382322(14759537.4991748 to 16326582.9141101) |
| 85-89 | 2002 | 480363.53984828(410501.411672036 to 551465.121213404) | 794287.449461533(725004.306441103 to 836829.390063144) | 10387584.0428957(9521994.18057212 to 10956647.8236082) |
| 85-89 | 2003 | 477144.825297661(407284.746967049 to 548261.313719453) | 645929.052667809(571813.914820703 to 683419.596249172) | 6510481.79444761(5802612.96038413 to 6891411.09453886) |
| 85-89 | 2004 | 480559.632149502(409960.359718603 to 552391.520362575) | 286461.883551831(242549.624746807 to 308530.340844924) | 2513721.01620013(2134271.73472704 to 2703652.26003708) |
| 85-89 | 2005 | 492247.828733635(419973.485235363 to 565463.278261221) | 82407.7311432238(63681.1168056562 to 91368.3725640015) | 673843.706338196(531711.327246549 to 743911.081885036) |
| 85-89 | 2006 | 510536.664839874(435399.666061928 to 585581.323384992) | 489602.707095666(469932.440242829 to 507454.523998143) | 15436515.5963808(14841963.5536973 to 16087312.8177098) |
| 85-89 | 2007 | 529768.42343222(451809.713194868 to 606580.876922611) | 717910.838277079(690270.563201157 to 744227.776754324) | 17917484.0105912(17197606.9472286 to 18666569.441051) |
| 85-89 | 2008 | 548846.835802376(468652.66271863 to 625754.788940302) | 955297.960818227(916996.392889083 to 990030.739965502) | 19460412.3483168(18666853.0292633 to 20254349.9739478) |
| 85-89 | 2009 | 567187.826998489(485274.91188713 to 645263.813352379) | 1050136.81290188(994928.235215718 to 1088738.74658498) | 16159351.9460855(15315332.5388357 to 16888382.1005263) |
| 85-89 | 2010 | 587056.869423549(503633.507777506 to 666695.320035253) | 892371.621503784(810929.82640296 to 934207.443414619) | 10530272.2939953(9675454.18853359 to 11103225.0427817) |
| 85-89 | 2011 | 606335.227938934(521298.683276782 to 687941.633018121) | 665045.401354395(587345.453599677 to 703042.781456693) | 6716127.64015923(6001685.32045992 to 7100971.75830257) |
| 85-89 | 2012 | 627231.411201434(540540.659700589 to 711781.806121341) | 316090.034337166(265926.326279378 to 340522.579579073) | 2585058.04575023(2202095.69401031 to 2784086.59370264) |
| 85-89 | 2013 | 648965.488300655(560539.661462079 to 736876.142166609) | 94843.6906051378(72818.5049347171 to 105174.556934476) | 698829.445014905(550060.531263157 to 772375.644334698) |
| 85-89 | 2014 | 672474.09004825(581645.397263981 to 763651.870747424) | 476912.888475279(458928.330979215 to 495963.371251191) | 15350410.8260786(14761496.9975112 to 15992747.6235303) |
| 85-89 | 2015 | 697770.358566226(603409.116477433 to 792708.142426018) | 713007.462628767(683783.761377883 to 741296.45435614) | 18085094.5367189(17322123.6336669 to 18860572.7923728) |
| 85-89 | 2016 | 729320.822406049(630635.593586895 to 828708.034658178) | 949099.804415404(911027.947160905 to 985556.223513897) | 19822171.2449626(18948881.3522967 to 20621144.485439) |
| 85-89 | 2017 | 761798.471054142(658197.184416614 to 865998.79875014) | 1069688.02969547(1015605.63217192 to 1111463.14956581) | 16689218.9467798(15840256.2302912 to 17489109.378029) |
| 85-89 | 2018 | 795506.152408163(686681.819208403 to 905379.817343184) | 920483.793278169(837282.083093122 to 963224.343380565) | 10883642.133553(9947529.9632159 to 11470772.5456623) |
| 85-89 | 2019 | 827180.194882009(712975.050776761 to 942135.459051412) | 661998.071134269(583060.69849137 to 700521.551437293) | 6829167.54588941(6084810.66888906 to 7231494.94431706) |
| 85-89 | 2020 | 823142.099208769(703720.417076276 to 943696.502335994) | 314822.932962063(265060.258211947 to 339531.595832056) | 2684482.53157446(2288049.00596872 to 2893044.03241084) |
| 85-89 | 2021 | 849590.161316785(726994.64001582 to 974430.649764546) | 95855.0544640702(73385.0613126924 to 106770.252427988) | 727547.945440118(571880.000777214 to 806318.116449201) |
| 80-84 | 1990 | 644816.151799146(534607.606122468 to 772992.146202766) | 452149.153589609(432581.153933504 to 469269.380535897) | 15274335.7864777(14643639.2018319 to 15871255.2805381) |
| 80-84 | 1991 | 662353.104350553(551372.683499227 to 790630.268135054) | 672788.296258767(642772.337950107 to 699863.311268162) | 18324692.1403747(17493665.7643568 to 19134415.6164473) |
| 80-84 | 1992 | 677199.135712532(565088.636729492 to 804249.013239553) | 904707.448663467(863083.635039042 to 939091.662857286) | 20048085.0352895(19176604.0622781 to 20846762.0740311) |
| 80-84 | 1993 | 692859.007410187(578824.625338415 to 821753.535979128) | 1054836.64983256(997886.09835334 to 1093249.61504353) | 17257150.032183(16367345.2508275 to 18017978.873088) |
| 80-84 | 1994 | 704711.938883387(588555.050565595 to 832523.30474522) | 931694.708912992(848311.935059024 to 976761.029514216) | 11327529.1111496(10409317.0746292 to 11913618.7758226) |
| 80-84 | 1995 | 713002.636076934(595636.489970845 to 839492.430982844) | 645730.638524282(564751.154264252 to 684575.332685234) | 6830275.91291665(6071644.73547228 to 7211219.6064697) |
| 80-84 | 1996 | 711943.311681856(595548.663663442 to 838222.17235292) | 308709.55626921(257681.571099058 to 334645.96440064) | 2763539.75387254(2354891.76061553 to 2972081.33188222) |
| 80-84 | 1997 | 707082.120397864(591368.666455762 to 832812.049033247) | 100354.843841608(76333.0744630389 to 112217.985325192) | 769944.628010773(603540.774848761 to 851392.049699298) |
| 80-84 | 1998 | 701514.931426479(586484.151933969 to 826546.302647093) | 463439.729580416(445823.534231005 to 482009.7710429) | 15106750.2660104(14480679.9640077 to 15658656.6755008) |
| 80-84 | 1999 | 703948.325452001(588631.839709938 to 829470.752798504) | 704204.462439555(677390.51457043 to 732011.981153749) | 18576352.8698103(17802436.1751679 to 19311086.1832931) |
| 80-84 | 2000 | 718357.687419113(601120.898916988 to 846067.898790229) | 938091.730713125(897249.994854233 to 976256.237247404) | 20239096.6146162(19405408.3203942 to 21001543.8202481) |
| 80-84 | 2001 | 742825.428053791(622376.169208179 to 875162.565136439) | 1082566.62562412(1028785.91094152 to 1126520.43923492) | 17837831.6295908(16878773.2217938 to 18541936.3773362) |
| 80-84 | 2002 | 770288.894767924(646218.20617599 to 907375.422023355) | 942678.268367751(864026.748002741 to 986889.686653552) | 11858102.0451433(10926289.0540404 to 12415210.1808323) |
| 80-84 | 2003 | 799250.409289791(671330.094216141 to 941533.387082372) | 661419.04802411(582574.295969723 to 701604.39505199) | 6898967.94019117(6148086.87740792 to 7289284.89151224) |
| 80-84 | 2004 | 826478.364234296(694624.719237458 to 973888.733328867) | 316018.435071078(266790.394289762 to 341594.128416788) | 2854301.5768768(2428061.18062838 to 3072675.7772966) |
| 80-84 | 2005 | 852199.625041119(716257.473975796 to 1004654.99797988) | 97882.4153085272(74668.6879654395 to 109215.692504048) | 804868.156749382(633401.063269126 to 890990.297111109) |
| 80-84 | 2006 | 874683.627458413(734865.752153627 to 1029447.13222128) | 455923.997341027(437399.606552729 to 475499.821397834) | 14736872.7010129(14081787.7894192 to 15372658.6570307) |
| 80-84 | 2007 | 896259.795928612(752689.932424571 to 1052930.83391217) | 651370.076396394(623618.818942936 to 675861.822818246) | 18464676.4281796(17692512.8155352 to 19218070.7526371) |
| 80-84 | 2008 | 915857.53366976(768476.49157128 to 1075088.67672426) | 900079.26930926(861172.551664036 to 935707.281639617) | 20128960.3583226(19265990.2022702 to 20944673.590493) |
| 80-84 | 2009 | 935259.475832436(785067.340417583 to 1095692.64449489) | 1040024.69280035(984336.793301139 to 1083768.64381113) | 18176964.1087187(17260226.3772055 to 18927068.0646016) |
| 80-84 | 2010 | 954499.048797483(801401.816741594 to 1116298.56080898) | 945652.799328099(862822.5319731 to 995431.390364333) | 12238548.344272(11193187.1176039 to 12821256.8543247) |
| 80-84 | 2011 | 973809.445017046(817437.707725462 to 1136443.15567589) | 661966.674319272(576391.695650771 to 703389.484256971) | 6883861.11093164(6133289.73439145 to 7276933.04179046) |
| 80-84 | 2012 | 988596.468449767(829479.298720173 to 1151270.00444388) | 311214.64761582(259584.17962298 to 337082.622248555) | 2847931.25846435(2420988.8663421 to 3065408.50583175) |
| 80-84 | 2013 | 1004596.26233782(843215.256198437 to 1169355.06953817) | 104742.110487063(79539.880256799 to 117070.112986957) | 813954.749493365(636108.41826358 to 904405.732773785) |
| 80-84 | 2014 | 1024998.14833205(860958.633112468 to 1192948.90157742) | 467396.722075072(448450.631333432 to 489324.748899309) | 14351531.1693646(13790599.6348462 to 14967001.6899166) |
| 80-84 | 2015 | 1054433.35438385(885581.365327074 to 1229430.27825793) | 642617.755420931(613264.16266742 to 670517.591601103) | 18254400.3787965(17564114.5552892 to 19001168.5930597) |
| 80-84 | 2016 | 1099041.47896138(921805.98124987 to 1281367.82753515) | 902386.481670416(862318.613164045 to 942715.417151231) | 19928205.597077(19104509.4992247 to 20758673.6027234) |
| 80-84 | 2017 | 1153933.02806772(966931.777303174 to 1346294.23594976) | 1047624.32159114(986539.942724074 to 1095237.65520477) | 18401704.1710869(17570025.6682795 to 19209130.9126006) |
| 80-84 | 2018 | 1206927.87619914(1010309.44200777 to 1409714.66185362) | 980473.420256572(890947.302977641 to 1035577.92752531) | 12545480.3990151(11599094.0842831 to 13158045.0532922) |
| 80-84 | 2019 | 1249921.80591759(1045452.77466418 to 1461705.58719984) | 694563.509204488(603773.502345231 to 739572.604527592) | 6905384.17345917(6108568.26655876 to 7313965.95602997) |
| 80-84 | 2020 | 1240673.90210799(1028130.30580915 to 1462448.09737883) | 313188.953782943(261691.323389199 to 339242.302529644) | 2861674.88173121(2435736.09348472 to 3085492.81216089) |
| 80-84 | 2021 | 1262270.74384746(1043424.44426828 to 1488366.00349361) | 109895.829699504(83377.6680567746 to 123204.912446185) | 831895.546110375(651724.505900195 to 924791.021293961) |
| 75-79 years | 1990 | 817665.109744557(649688.205545962 to 1025347.12614644) | 497206.317032019(475191.096513846 to 518601.174830957) | 14054451.8493606(13472022.6418469 to 14666252.9820538) |
| 75-79 years | 1991 | 817225.466899727(653221.703152973 to 1020699.14171779) | 630525.213006071(603018.675676294 to 658464.610820771) | 17495383.4737475(16797223.3373313 to 18242191.7394344) |
| 75-79 years | 1992 | 813641.452900417(653346.543544742 to 1012715.2152912) | 890957.578166894(851334.862915319 to 929605.842886724) | 19297116.3874108(18491298.532998 to 20115125.1521472) |
| 75-79 years | 1993 | 809484.858926858(651815.726189424 to 1004722.04570767) | 1049449.29929057(992133.880214962 to 1099070.98579487) | 17974170.3841426(17083500.9270348 to 18747173.3797588) |
| 75-79 years | 1994 | 813046.239731456(659533.9336002 to 1006876.49318797) | 1036796.34713263(941988.427187208 to 1090793.09742448) | 12434719.5455442(11420019.6363527 to 13074856.0178056) |
| 75-79 years | 1995 | 829354.748207751(673217.270956667 to 1024286.6028906) | 746240.236216878(646603.967564047 to 794509.554307832) | 6780320.72525628(6023450.5655518 to 7187759.34992277) |
| 75-79 years | 1996 | 854262.867923441(695106.825219157 to 1051963.20672432) | 326607.924929621(271177.884525059 to 354039.80599619) | 2800922.8180508(2368219.48462955 to 3032277.81572974) |
| 75-79 years | 1997 | 880953.551191646(717841.190350207 to 1082635.37655398) | 117504.238439352(89147.9599374295 to 131836.323549546) | 853378.932697829(665596.584412962 to 951354.756992898) |
| 75-79 years | 1998 | 908518.951108591(741103.46450041 to 1115039.92626488) | 484786.261999976(463159.167277443 to 504815.548295133) | 14223119.3570454(13622082.3954908 to 14835737.710013) |
| 75-79 years | 1999 | 934791.501476449(762687.51706222 to 1146464.44924349) | 634791.679297102(606773.171481233 to 660791.445601028) | 16975892.5666788(16274970.339901 to 17623968.8315516) |
| 75-79 years | 2000 | 960350.089724993(783786.056110765 to 1177678.1150231) | 894964.937730282(855596.477220386 to 933999.910141074) | 19238194.5239147(18417542.4842774 to 20037142.4167917) |
| 75-79 years | 2001 | 986028.767427861(803900.33491422 to 1211262.89339141) | 1048568.97402549(990291.746080362 to 1096220.61038453) | 17751200.0119812(16814760.3659018 to 18501671.3176455) |
| 75-79 years | 2002 | 1013679.79934033(825418.952300654 to 1247703.99575377) | 1008587.17708025(918996.259692832 to 1060660.72940886) | 12637584.1787191(11641788.1681489 to 13327291.7595469) |
| 75-79 years | 2003 | 1041622.71764934(846691.065628399 to 1284605.60124125) | 720726.982040772(632666.229126631 to 766121.173835624) | 6966441.79108272(6145272.40434135 to 7412396.62143914) |
| 75-79 years | 2004 | 1068719.11801087(867355.633772205 to 1318555.69438064) | 315687.680769218(264396.179599094 to 342020.788949854) | 2825000.99594102(2390338.44685902 to 3054685.97019613) |
| 75-79 years | 2005 | 1091302.41345858(884079.170678894 to 1347647.98543609) | 113272.738324248(85596.8937495378 to 127296.072608364) | 891169.955163344(694834.178782673 to 992258.656025484) |
| 75-79 years | 2006 | 1108967.63591546(899685.760868377 to 1366895.69544921) | 555460.490795438(517713.293551506 to 594377.254694718) | 14601817.2893472(13923783.4840079 to 15318110.2108668) |
| 75-79 years | 2007 | 1118736.17756841(907057.482779459 to 1375508.99523989) | 770714.652812203(713215.104662045 to 823602.058711971) | 16762018.2551772(16046625.0428227 to 17523789.9481922) |
| 75-79 years | 2008 | 1125686.75333245(912207.868980576 to 1380420.82287582) | 924001.207959218(851616.856072232 to 990425.582098751) | 19310284.9874358(18488561.3333146 to 20175660.7003093) |
| 75-79 years | 2009 | 1134731.12410742(919194.098279644 to 1387912.79077723) | 1050124.46118207(967318.63355456 to 1124949.76442602) | 17888338.2200346(16922058.8566693 to 18710094.6573749) |
| 75-79 years | 2010 | 1149270.35628971(931469.147271926 to 1405245.00168265) | 1117530.22505985(1005547.22746261 to 1200307.67963032) | 13103045.2488878(12014899.9196376 to 13854106.656859) |
| 75-79 years | 2011 | 1170323.16306351(948906.041509661 to 1430853.90112043) | 913710.954704202(785264.495773081 to 989480.17016475) | 7310842.15512835(6413044.03522597 to 7790049.39291924) |
| 75-79 years | 2012 | 1193366.18546167(967948.244891067 to 1459753.22810117) | 479516.081949005(393921.975765098 to 526515.419804131) | 2846025.84741914(2402515.22796578 to 3081292.48090558) |
| 75-79 years | 2013 | 1215179.59792115(983970.11783713 to 1488261.3110427) | 173899.138850903(129640.165543391 to 196686.782344488) | 935472.766886772(728106.398294873 to 1044027.38840423) |
| 75-79 years | 2014 | 1237759.25500357(1000839.50495702 to 1515260.76742417) | 547171.642720017(508016.297651275 to 589216.621878927) | 15174599.7684117(14473331.8570454 to 15849858.8852264) |
| 75-79 years | 2015 | 1266083.72402029(1023770.37607587 to 1548613.83238489) | 804420.822265618(741167.417813551 to 871265.654842315) | 16577894.1045862(15860499.3626287 to 17253419.0729403) |
| 75-79 years | 2016 | 1299715.92944596(1050545.58125517 to 1589223.23799028) | 1002543.9072474(919714.653809738 to 1086294.74502542) | 19176679.9205274(18331373.3492187 to 20012688.2412476) |
| 75-79 years | 2017 | 1336703.56556811(1080296.20448598 to 1635097.5484298) | 1060437.73955709(965332.207329842 to 1149678.39588871) | 17929724.3458656(16997155.9048712 to 18777612.6802684) |
| 75-79 years | 2018 | 1373034.32839446(1109327.40032567 to 1680414.62133205) | 1140304.34062849(1006577.47933661 to 1235802.85478692) | 13484745.8380807(12425281.0129181 to 14198077.8145497) |
| 75-79 years | 2019 | 1401466.8245556(1131979.81421632 to 1716405.94069843) | 953088.986829219(817408.630308835 to 1040789.64268816) | 7590778.67174963(6716448.5714701 to 8054849.85278677) |
| 75-79 years | 2020 | 1384219.23254168(1104329.11703086 to 1715636.09601496) | 502888.020879966(412267.881136623 to 556597.772075193) | 2873910.24489409(2432641.30635892 to 3109474.64632127) |
| 75-79 years | 2021 | 1425459.48560386(1138104.20468492 to 1766190.81891581) | 184066.652380819(137067.850628302 to 209579.453005391) | 965005.938273299(748494.623024095 to 1079645.08669571) |
| 70-74 years | 1990 | 817187.263179795(657774.678238992 to 1005322.72915733) | 511494.687552238(488528.973673205 to 534535.422459803) | 15568917.1848158(14795913.1327473 to 16276347.2508415) |
| 70-74 years | 1991 | 843324.965075502(681259.476207819 to 1033023.88063938) | 626893.590637136(598762.174839089 to 656431.493376518) | 16486462.598139(15730813.7655418 to 17234548.0734931) |
| 70-74 years | 1992 | 874158.758054665(708215.46728023 to 1066461.34972787) | 875707.298889528(835344.762139128 to 915458.780493875) | 19109517.439384(18264765.5328529 to 19995790.2845457) |
| 70-74 years | 1993 | 907478.469455599(736992.447830248 to 1103345.25936584) | 1043310.28689746(990093.394303391 to 1093179.18949494) | 17974560.7930774(16959500.2403502 to 18868883.5190293) |
| 70-74 years | 1994 | 938994.835352075(764322.964686026 to 1137543.02281012) | 1031481.41863943(943561.402667999 to 1087332.50610608) | 13862027.5329509(12704127.7457751 to 14605748.9955902) |
| 70-74 years | 1995 | 967948.456959572(789294.68685539 to 1169234.65983849) | 755162.51508198(660403.35095224 to 807158.431524758) | 7864901.40184369(6889546.12922469 to 8354996.95876887) |
| 70-74 years | 1996 | 993024.111130972(810304.297858978 to 1199517.52489396) | 338780.247126405(283999.54131279 to 367935.503880348) | 2979867.83482852(2515528.93631995 to 3221115.14952394) |
| 70-74 years | 1997 | 1015655.2477623(829210.479159393 to 1227118.71513236) | 119705.238272252(90713.2338714616 to 134769.296532511) | 1001738.66133477(781901.120671056 to 1121624.0013769) |
| 70-74 years | 1998 | 1035998.03405598(846169.339552741 to 1250241.39137726) | 521126.375465669(494788.681457338 to 549153.473061001) | 16045350.8193482(15248587.9361465 to 16788684.9731741) |
| 70-74 years | 1999 | 1055306.55679534(862356.556889553 to 1272214.07596813) | 631745.571258302(597554.211222943 to 665783.810984528) | 16438327.9915926(15597633.0229809 to 17197610.2033189) |
| 70-74 years | 2000 | 1071848.0856039(876484.464254094 to 1290729.09367673) | 860373.738020169(813845.685629178 to 901406.071200024) | 18816005.2385772(17881847.7318193 to 19680171.3093442) |
| 70-74 years | 2001 | 1086840.85947585(889046.786123263 to 1309491.08089624) | 1041700.45111978(979669.850682141 to 1096207.79092483) | 17921217.3495267(16981444.3049954 to 18768251.1275824) |
| 70-74 years | 2002 | 1095557.81428589(895745.158635389 to 1320942.9302622) | 1015905.71822535(924514.245672023 to 1078890.47621213) | 13817255.0622818(12705251.179763 to 14583457.9269119) |
| 70-74 years | 2003 | 1101754.0764311(900455.950827166 to 1329333.2469828) | 766177.6282546(664738.673852001 to 820015.530033155) | 7970056.69217173(7041837.03427813 to 8479800.43666013) |
| 70-74 years | 2004 | 1107210.57212341(904685.576124762 to 1336312.37500435) | 353857.15073539(291701.397998185 to 384301.278721795) | 3097771.99057882(2630357.989329 to 3358999.82336281) |
| 70-74 years | 2005 | 1114143.47933023(910242.184789394 to 1345935.04861762) | 121409.067960666(91290.4558285248 to 136101.315227195) | 1021357.52847088(795666.885132472 to 1144478.05439794) |
| 70-74 years | 2006 | 1123442.57514461(918591.846274657 to 1358038.09763858) | 526882.111381201(499954.817680676 to 557287.247813465) | 16372172.5037011(15489365.5484733 to 17257899.3789634) |
| 70-74 years | 2007 | 1132311.9909393(924842.087866119 to 1366430.23975816) | 631909.030370583(598511.71421515 to 664413.244356019) | 16632981.149795(15724230.6506 to 17546811.3809343) |
| 70-74 years | 2008 | 1136972.51218003(926720.342280416 to 1368431.17049829) | 840178.516948338(796241.294242002 to 886828.68628164) | 18520558.9161872(17538164.2258106 to 19431903.1636844) |
| 70-74 years | 2009 | 1139172.5963677(927258.745186943 to 1370527.91128511) | 1041901.72227071(976229.213017291 to 1098155.41441985) | 17938039.232624(16917003.4445973 to 18884030.7649498) |
| 70-74 years | 2010 | 1144196.73891125(931498.307158474 to 1377273.01077873) | 1025715.22222667(928139.640782572 to 1088205.72374269) | 13642257.7068794(12495990.4396967 to 14523469.0631417) |
| 70-74 years | 2011 | 1146489.25431526(935349.372982895 to 1385597.30389457) | 796123.268092611(695251.844574734 to 851651.262048001) | 8097856.47336307(7083335.2172469 to 8660380.67621111) |
| 70-74 years | 2012 | 1143884.00666207(935189.804048 to 1384726.44168972) | 379257.756254242(314716.840070733 to 412194.753615461) | 3240614.07649548(2723413.85795502 to 3521915.70716467) |
| 70-74 years | 2013 | 1141733.05265063(934549.4087631 to 1383373.70333173) | 125663.360150901(94587.9343502233 to 141241.805414948) | 1036645.92050448(802893.285527352 to 1162417.52269164) |
| 70-74 years | 2014 | 1144377.51254443(937826.024023696 to 1388390.32082897) | 529278.193483211(501456.271368182 to 559470.29875931) | 16585651.3237404(15687753.2552639 to 17539005.3940643) |
| 70-74 years | 2015 | 1156270.30605626(948504.25797724 to 1404422.50822616) | 646615.477167747(607900.342783176 to 684198.207283915) | 16700017.2776969(15792033.5331344 to 17596189.5640223) |
| 70-74 years | 2016 | 1196054.0849986(979382.651594526 to 1453851.61266705) | 819941.039631571(769967.085387492 to 868274.558639591) | 18125087.5430928(17165415.301573 to 19107754.1374999) |
| 70-74 years | 2017 | 1269120.2801185(1036751.2205856 to 1543951.13501027) | 1043154.13769734(974791.113051844 to 1102291.9484426) | 17968657.8031617(16866308.9154849 to 18944402.7998907) |
| 70-74 years | 2018 | 1345617.00190884(1096399.12729016 to 1638036.52532931) | 1044383.4239369(950359.589311524 to 1115891.05389815) | 13785730.127424(12580486.6643539 to 14616640.7684046) |
| 70-74 years | 2019 | 1450384.85872582(1178374.97274693 to 1767065.50585704) | 825867.47740305(716678.050002062 to 884000.595715777) | 8415424.60399163(7371024.03021332 to 8996823.81933206) |
| 70-74 years | 2020 | 1478058.02319812(1186908.07312339 to 1804174.76899915) | 400915.715107739(331927.862319206 to 435878.353689787) | 3471489.46799776(2936564.58612068 to 3767507.56939808) |
| 70-74 years | 2021 | 1562049.46091053(1247981.47822286 to 1912002.85334534) | 131749.049113954(99718.1496715578 to 147349.6109929) | 1073722.02061401(833492.390797194 to 1206149.77487285) |
| 65-69 years | 1990 | 827047.733406335(620696.8651483 to 1080133.83737274) | 549433.006262107(520338.027107966 to 581770.153874033) | 16706870.5941796(15821844.2949974 to 17617982.5811664) |
| 65-69 years | 1991 | 851949.463525399(642702.302576251 to 1108410.98435922) | 668650.051282765(629654.577849008 to 705677.481998056) | 17157324.9052777(16222018.4939961 to 18088969.379467) |
| 65-69 years | 1992 | 877156.901799723(665006.805749392 to 1137079.82894241) | 814833.988891219(769518.999391571 to 860863.511787164) | 17733262.6164051(16758484.9983553 to 18763612.245945) |
| 65-69 years | 1993 | 901234.208195715(685429.039767826 to 1165354.07689471) | 1046360.21810733(981696.021764836 to 1105583.54877275) | 18006411.3936358(16811699.4344712 to 19026213.5036959) |
| 65-69 years | 1994 | 923820.937214991(705685.87029424 to 1193385.60597074) | 1043648.24010576(942844.974095439 to 1111532.04103056) | 14048866.1350133(12861189.6468533 to 14929958.7957355) |
| 65-69 years | 1995 | 941666.883729189(722486.190053027 to 1215575.17434375) | 841694.781092178(725887.677074647 to 903769.598398171) | 8732614.3966767(7654213.67911227 to 9329665.83122563) |
| 65-69 years | 1996 | 954057.916704196(733009.658745084 to 1229889.64511514) | 415549.999709695(344713.51669265 to 453493.60736848) | 3669557.17144818(3091511.56295428 to 3986594.5089205) |
| 65-69 years | 1997 | 956566.691872127(735784.568353459 to 1232122.74535925) | 138585.796549218(104284.234771892 to 155876.693286156) | 1126075.6456078(874456.092245972 to 1257601.55524694) |
| 65-69 years | 1998 | 954210.796510196(734610.36658711 to 1228610.57620392) | 555337.203285896(522823.681702776 to 591686.726221983) | 17334665.7724686(16305594.8791068 to 18436274.5816801) |
| 65-69 years | 1999 | 950221.24495283(731292.531356168 to 1223298.87881641) | 700424.623192138(656268.269040353 to 749961.232409443) | 17741276.4564876(16668618.8868089 to 18784533.0494251) |
| 65-69 years | 2000 | 950627.741214422(731564.42969208 to 1223562.18691038) | 830341.458441299(777216.689902644 to 886340.636004256) | 17652687.0151754(16681805.913154 to 18726142.6918759) |
| 65-69 years | 2001 | 958486.386889604(737574.468971118 to 1234390.3745883) | 1050314.40447274(968825.788422566 to 1119208.10035274) | 18079745.1009(16987939.0602291 to 19171190.782432) |
| 65-69 years | 2002 | 970891.390735527(746410.651778969 to 1250941.90657215) | 1059349.78316106(947719.118545065 to 1132561.34579879) | 14076801.6328409(12861263.2940525 to 15005630.331438) |
| 65-69 years | 2003 | 982076.604879547(754541.646985134 to 1265565.70309681) | 866526.969381353(743906.388750352 to 930569.599841637) | 8908551.15214495(7821125.55182297 to 9541189.76535842) |
| 65-69 years | 2004 | 989774.489061988(760143.629842221 to 1275495.0422986) | 432074.438804682(354467.626194906 to 471508.901520698) | 3807107.39806863(3194610.01222692 to 4147156.51236346) |
| 65-69 years | 2005 | 993782.005491275(763114.529955355 to 1280479.72110741) | 147139.451560915(110560.443043382 to 165832.539889494) | 1185569.86003112(919829.969473428 to 1329295.94082696) |
| 65-69 years | 2006 | 988312.280108955(757858.450647263 to 1271867.13003338) | 554659.741231892(519399.013284019 to 594670.995700156) | 17538653.1268487(16545890.0800084 to 18637955.1149779) |
| 65-69 years | 2007 | 974115.348211899(747746.719230067 to 1253012.53690364) | 721648.833149142(677903.490115287 to 772224.75726976) | 18596348.5593256(17482027.4888865 to 19828846.4861783) |
| 65-69 years | 2008 | 956299.34332241(735137.193241463 to 1230401.47440449) | 854659.660984969(799682.851664468 to 913738.295563042) | 18017043.856946(16968990.9612559 to 19091880.609042) |
| 65-69 years | 2009 | 941434.648327144(725351.242034715 to 1210407.22011872) | 1047960.14697392(975225.061078453 to 1121768.67867972) | 18164871.0439674(16944957.131034 to 19247001.0123705) |
| 65-69 years | 2010 | 933240.710671756(719202.490838239 to 1199122.18354877) | 1080795.30698695(971582.177696374 to 1168290.83329976) | 14317310.1795983(12965548.5176092 to 15283608.2778824) |
| 65-69 years | 2011 | 939991.982953056(725075.36377984 to 1204944.87381051) | 881902.80910304(764039.019376834 to 956175.874492248) | 9173248.64560661(8033539.4358723 to 9844472.1317908) |
| 65-69 years | 2012 | 964989.506050709(743137.550539661 to 1233909.66038199) | 446728.579921384(368714.036003337 to 493177.5590514) | 3961067.72289583(3316856.64597605 to 4316646.35474085) |
| 65-69 years | 2013 | 993564.992932258(764200.767126661 to 1268453.63892013) | 156611.644012987(118290.438352415 to 176500.995191434) | 1259434.73217998(976980.67744127 to 1415984.79232145) |
| 65-69 years | 2014 | 1047467.05985499(804012.293841322 to 1340681.90294116) | 557682.511190895(518958.244093465 to 592657.350670308) | 17536203.232024(16391300.856202 to 18702082.4413367) |
| 65-69 years | 2015 | 1093271.59761166(838006.670314106 to 1399149.69237864) | 753473.845649913(703453.73473626 to 803107.189762708) | 19174199.122797(17943620.3640774 to 20409944.9543369) |
| 65-69 years | 2016 | 1163140.0100685(891207.319401649 to 1491788.03215062) | 882358.366873149(822029.309712177 to 943105.589941242) | 18600836.5687315(17353853.9694414 to 19858208.9484087) |
| 65-69 years | 2017 | 1231240.71668332(941285.394755649 to 1581640.72335391) | 1047674.38891831(972282.90107489 to 1116674.16729361) | 18149213.5443598(16882903.1326993 to 19392839.1124056) |
| 65-69 years | 2018 | 1308362.76249978(997280.678239092 to 1683107.62333354) | 1100637.38892818(984882.442361102 to 1186965.86957671) | 14625084.1954131(13309617.1391847 to 15691228.6960236) |
| 65-69 years | 2019 | 1364597.82154366(1038192.52243529 to 1758071.82568521) | 894028.244624458(767491.219732755 to 966614.796930629) | 9343409.42852943(8144274.41949499 to 10080104.2933177) |
| 65-69 years | 2020 | 1395591.18751982(1047195.17267651 to 1823571.67203071) | 460952.625163968(380832.328217925 to 505850.097125944) | 4099349.65413696(3446324.7328231 to 4498820.8197764) |
| 65-69 years | 2021 | 1434649.34201396(1072983.59618192 to 1884349.2949482) | 165470.750216397(125751.516242225 to 186155.877360776) | 1341655.11246467(1045866.81365274 to 1503199.30580895) |
| 60-64 years | 1990 | 743202.394258627(589688.375426837 to 933601.16488274) | 500783.679139646(478670.034367311 to 525781.044122588) | 17625859.8705167(16564881.9279474 to 18750616.6795662) |
| 60-64 years | 1991 | 755836.072497044(603300.681634825 to 945265.564709826) | 691849.769565785(660819.998258866 to 723731.591257525) | 20009477.5526519(18757215.3537127 to 21371088.0367769) |
| 60-64 years | 1992 | 762592.546194042(610132.289683209 to 946845.782793296) | 840349.775477466(802944.840008486 to 875230.076017506) | 19235310.7841896(18029071.2986791 to 20494823.6137106) |
| 60-64 years | 1993 | 766443.108654457(615689.604464841 to 944447.237042794) | 834068.173676803(788295.804582815 to 875373.533730115) | 18164148.7378218(16855622.4761113 to 19381069.0590153) |
| 60-64 years | 1994 | 768047.539947795(619869.10619212 to 939749.425693083) | 813397.979259204(742714.875293138 to 855837.454613136) | 14905350.1887859(13361747.865036 to 16028126.045341) |
| 60-64 years | 1995 | 771197.339255743(624728.86721778 to 937713.239112134) | 582446.591036168(511836.361726267 to 616770.589809427) | 9483738.81959239(8180458.67805308 to 10282232.3831425) |
| 60-64 years | 1996 | 776710.147587042(630238.743680907 to 943377.119490207) | 251699.234418479(211506.266510086 to 271285.56385391) | 4234927.48285469(3525696.37535203 to 4647708.04250284) |
| 60-64 years | 1997 | 783258.234375107(636327.304429853 to 950747.250589434) | 70069.6017437769(54677.840643776 to 77382.357356389) | 1418100.98031361(1103873.73589983 to 1590939.50054866) |
| 60-64 years | 1998 | 788335.281746923(640693.592668621 to 956678.395246766) | 495032.243117519(474411.801446732 to 514404.582036246) | 17563470.955608(16369131.7442104 to 18838091.7716499) |
| 60-64 years | 1999 | 791969.223132569(643296.981229988 to 961073.866336901) | 708270.141965713(677070.220482943 to 736074.39545781) | 20499032.5586058(19048026.8072296 to 21843800.4233421) |
| 60-64 years | 2000 | 795080.682982924(645498.959173181 to 964845.828433717) | 945950.827910813(905560.727627181 to 981252.079740008) | 20193843.0346936(18619463.2538899 to 21616868.1866665) |
| 60-64 years | 2001 | 794657.323487341(645140.447043014 to 963716.098309887) | 1015492.11736803(965792.054521701 to 1056398.0076171) | 18226762.4295028(16820560.1790147 to 19540619.5380351) |
| 60-64 years | 2002 | 790262.48359075(641396.520978284 to 958068.947908535) | 852030.303339004(776424.388430028 to 893385.393338487) | 15139962.804525(13762995.183913 to 16256977.5408485) |
| 60-64 years | 2003 | 782602.830481937(635015.764350958 to 948873.03339089) | 658154.088811277(582184.452329633 to 696753.986815362) | 9702889.95358271(8458471.27235817 to 10480834.966849) |
| 60-64 years | 2004 | 775142.463999598(628789.821322621 to 940518.812171111) | 306099.733305543(257580.961698661 to 329135.989494538) | 4407700.04171529(3672279.78891627 to 4844499.39911414) |
| 60-64 years | 2005 | 768342.263432138(623094.572981939 to 932962.600271114) | 90791.1888063557(69857.266394082 to 100647.470957098) | 1490469.84369007(1145436.66898947 to 1671336.95728092) |
| 60-64 years | 2006 | 769589.225999857(624204.499340674 to 932254.353971769) | 548535.060379222(509665.789487706 to 587994.598193646) | 17381191.2428135(16209467.3674836 to 18584588.1626494) |
| 60-64 years | 2007 | 784735.364971298(636794.871930325 to 949527.20478634) | 795365.84513269(732802.399259824 to 857329.942438507) | 21159258.9790222(19619131.8328291 to 22620644.9047281) |
| 60-64 years | 2008 | 801245.409750534(651692.074418448 to 969309.806755198) | 959341.469452757(879959.088668176 to 1034533.75089355) | 20959179.2954035(19382606.2379256 to 22430661.4167746) |
| 60-64 years | 2009 | 833916.125750954(678511.838964594 to 1009759.10768572) | 1049256.31698386(961459.966866486 to 1125477.53577783) | 18249037.9941597(16845123.419818 to 19497132.5862372) |
| 60-64 years | 2010 | 858848.174793371(699144.291688539 to 1040681.89534342) | 1132214.95016375(1010396.97508203 to 1219420.87987309) | 15348620.1063107(13882274.8813893 to 16578280.58066) |
| 60-64 years | 2011 | 893059.002436283(727004.789040981 to 1081603.39393752) | 936606.222309904(798844.102925815 to 1016409.30295917) | 9956556.76559886(8614769.46762215 to 10789793.8912119) |
| 60-64 years | 2012 | 915284.386491722(745330.207236363 to 1107424.35964999) | 493971.110574305(404269.205125155 to 544105.866573923) | 4542384.46978742(3760984.10326429 to 4987013.78906715) |
| 60-64 years | 2013 | 941904.024891846(766456.755642765 to 1138516.89003863) | 179527.212308418(134046.817304131 to 203136.413159216) | 1544955.26391622(1195241.21819846 to 1743399.32056046) |
| 60-64 years | 2014 | 963016.959610188(784687.862636385 to 1162734.07256954) | 500928.703168373(474929.15190359 to 528000.121880667) | 17381535.0553121(16114611.7111275 to 18688860.7647733) |
| 60-64 years | 2015 | 1002378.01422994(816680.47234417 to 1209323.60474799) | 654845.059316842(623019.475573775 to 687871.951346134) | 21442625.7346857(19817583.9365341 to 23165515.9783066) |
| 60-64 years | 2016 | 1036145.37904079(844754.957168455 to 1250643.02818079) | 770732.030109028(732442.51605665 to 809415.16924826) | 21946243.3535908(20233361.8155238 to 23618316.725271) |
| 60-64 years | 2017 | 1071965.82829286(873062.045928306 to 1294999.12517255) | 822062.800567778(774901.792121247 to 865761.357104654) | 18508093.2044646(16930305.8725177 to 19917525.8763513) |
| 60-64 years | 2018 | 1099250.60572053(894236.869358685 to 1330773.54815363) | 793526.165678368(724616.132936008 to 836339.213577907) | 15483050.4016605(13869979.4833075 to 16721765.2779502) |
| 60-64 years | 2019 | 1113641.12615384(904759.221681556 to 1351257.60076474) | 541427.284903098(475465.423506184 to 575452.566883121) | 10155814.17181(8805779.47228231 to 11093582.8237355) |
| 60-64 years | 2020 | 1103310.85977223(887483.27960507 to 1351315.4017158) | 219794.343010504(184926.13439703 to 236657.768410654) | 4632555.82138732(3832331.10505302 to 5122467.14174491) |
| 60-64 years | 2021 | 1116594.39134541(898320.583498862 to 1365406.90716909) | 61151.4160214752(47940.036378452 to 67243.4439288624) | 1588783.39192034(1222221.75745211 to 1802468.37148506) |

**Supplementary Table 2-1**

|  | Age-standardized | Rate |  |
| --- | --- | --- | --- |
| **year** | Incidence | Deaths | DALYs  (Disability-Adjusted Life Years) |
| 1990 | 996.0622861 (1230.657419 to 798.7445574) | 981.8654905 (895.7902475 to 1042.223249) | 16791.36547 (15558.89798 to 17794.67313) |
| 1991 | 992.3834594 (1221.084881 to 799.5943611) | 968.1872997 (885.9246043 to 1025.828491) | 16583.15346 (15416.26429 to 17514.85305) |
| 1992 | 987.8790876 (1210.405144 to 798.6780837) | 962.1788425 (879.2205165 to 1022.584024) | 16503.80419 (15321.76216 to 17489.50302) |
| 1993 | 982.8898655 (1200.455815 to 797.0303982) | 965.9044016 (884.31175 to 1021.700062) | 16577.29861 (15456.65068 to 17468.60054) |
| 1994 | 977.6504582 (1190.189837 to 795.7233327) | 963.759597 (884.936341 to 1016.950238) | 16559.63159 (15483.85327 to 17420.17856) |
| 1995 | 972.4654575 (1180.574312 to 793.0765464) | 955.9157799 (876.1378137 to 1006.270658) | 16422.18961 (15302.19042 to 17228.90045) |
| 1996 | 965.5711434 (1170.878866 to 788.5222511) | 937.8743027 (860.714791 to 988.7247557) | 16130.52857 (15050.13719 to 16946.51197) |
| 1997 | 955.195419 (1157.512346 to 780.6734982) | 920.0810764 (844.7670984 to 966.7622085) | 15846.84757 (14812.78017 to 16607.78918) |
| 1998 | 943.041926 (1142.027984 to 771.1116946) | 908.4060647 (834.0983243 to 957.4194965) | 15653.77149 (14662.13745 to 16427.39564) |
| 1999 | 930.8296275 (1126.622544 to 761.1421502) | 901.1412365 (827.7104589 to 950.084348) | 15513.16134 (14546.73961 to 16301.49551) |
| 2000 | 919.6442777 (1112.583765 to 752.0678145) | 894.2000605 (823.1308174 to 939.485175) | 15399.11631 (14444.7625 to 16140.21799) |
| 2001 | 909.5180653 (1100.81981 to 743.8192393) | 888.4055964 (817.8029982 to 934.7952197) | 15286.10394 (14312.81686 to 16032.48685) |
| 2002 | 899.1533299 (1088.925458 to 735.0269175) | 881.9356451 (811.2910045 to 925.4111094) | 15172.69364 (14197.86506 to 15887.69655) |
| 2003 | 888.3718422 (1076.625653 to 725.8858714) | 877.9997761 (807.4754145 to 919.8196509) | 15080.56084 (14129.63191 to 15746.81305) |
| 2004 | 877.58703 (1063.945874 to 716.7725317) | 857.7224866 (789.0120767 to 899.9795561) | 14746.35693 (13798.04763 to 15431.09322) |
| 2005 | 866.3881774 (1050.828411 to 707.3153205) | 834.2514092 (768.6241737 to 876.4761711) | 14360.89598 (13489.56692 to 15043.69948) |
| 2006 | 853.2630924 (1033.631141 to 696.6853314) | 788.5566975 (722.2626789 to 827.9086761) | 13633.88573 (12774.36104 to 14297.46803) |
| 2007 | 837.1764395 (1012.537995 to 683.5068461) | 764.6216977 (700.5714765 to 804.746985) | 13220.18322 (12367.35825 to 13858.92827) |
| 2008 | 820.0986307 (990.2259771 to 669.6693305) | 754.1112506 (689.144498 to 796.4012883) | 13022.02543 (12150.29874 to 13705.46661) |
| 2009 | 805.0037603 (970.8410982 to 657.6575024) | 738.9927874 (676.9858279 to 778.9780193) | 12765.12805 (11928.40702 to 13402.33797) |
| 2010 | 793.2082399 (956.1700826 to 648.5584362) | 726.1803192 (663.2633286 to 766.3291816) | 12544.83464 (11679.03031 to 13197.67217) |
| 2011 | 783.568348 (944.2818546 to 641.3140342) | 703.5520418 (644.9344444 to 744.0512661) | 12190.19719 (11350.87134 to 12828.83279) |
| 2012 | 773.3222072 (931.348981 to 633.3340905) | 681.5729305 (619.5015454 to 723.718535) | 11843.97889 (10974.67611 to 12539.39031) |
| 2013 | 764.3595568 (920.4294591 to 626.0945544) | 667.1747646 (606.1124625 to 709.4925926) | 11554.78322 (10692.4008 to 12243.82257) |
| 2014 | 758.9662427 (914.1522629 to 621.8204451) | 655.3544899 (593.3027647 to 699.0317387) | 11315.4814 (10458.20142 to 12002.79803) |
| 2015 | 758.1614495 (913.2599231 to 621.1535954) | 643.5774695 (582.2473945 to 686.4571336) | 11137.99136 (10280.0554 to 11862.37419) |
| 2016 | 764.6489754 (921.6017816 to 626.1282319) | 636.635292 (570.7185385 to 683.0939844) | 11028.62391 (10148.71358 to 11760.50072) |
| 2017 | 775.3941423 (935.4248628 to 634.1476675) | 627.2139082 (564.6581321 to 677.2157812) | 10873.74816 (9965.391528 to 11649.49172) |
| 2018 | 785.8336405 (949.2043876 to 641.7292763) | 619.4025795 (555.1615672 to 666.44394) | 10761.79655 (9843.268817 to 11534.70971) |
| 2019 | 792.0944697 (957.9591579 to 645.9522636) | 611.9729522 (546.4445085 to 659.7105895) | 10642.33399 (9694.704843 to 11429.52317) |
| 2020 | 771.8979105 (942.1336015 to 622.6602571) | 605.5400282 (537.713038 to 655.009167) | 10531.56637 (9580.416413 to 11318.81924) |
| 2021 | 775.675428 (947.9602221 to 624.5969667) | 600.0754178 (531.6329648 to 653.2235085) | 10454.28207 (9462.831603 to 11305.68062) |
| 1990 | 798.3396962 (990.2266466 to 634.2812576) | 570.3618717 (509.9157523 to 600.799517) | 9325.450121 (8577.328638 to 9831.741977) |
| 1991 | 786.9815013 (971.2994704 to 628.1139883) | 552.8523811 (493.6590598 to 582.9185696) | 9076.443244 (8330.797141 to 9564.503053) |
| 1992 | 775.6404228 (953.7833293 to 622.195265) | 534.5889494 (476.3199697 to 563.5034876) | 8808.332894 (8078.961007 to 9290.856239) |
| 1993 | 764.3883672 (936.3211223 to 615.4040127) | 522.9214474 (465.4479618 to 551.4598281) | 8630.932878 (7920.23091 to 9104.217996) |
| 1994 | 754.1744384 (920.3577838 to 609.4327141) | 512.530427 (455.7407644 to 540.9585745) | 8475.272811 (7768.913237 to 8959.974203) |
| 1995 | 745.1438652 (906.516668 to 604.3243046) | 509.5766115 (452.6590819 to 538.2781414) | 8423.123145 (7704.356194 to 8900.736264) |
| 1996 | 733.6207393 (889.8549067 to 597.2359967) | 495.1076231 (438.78858 to 522.9598494) | 8194.326881 (7486.566486 to 8670.005632) |
| 1997 | 717.3657463 (867.9932709 to 585.9309585) | 478.4833705 (423.5775612 to 506.1374686) | 7949.347529 (7256.330205 to 8423.510267) |
| 1998 | 698.564769 (843.2088719 to 571.6337128) | 464.6110467 (411.189258 to 491.547902) | 7748.855247 (7060.311621 to 8205.660266) |
| 1999 | 680.250184 (819.4720336 to 557.9274633) | 455.6007722 (403.2926187 to 482.5280308) | 7603.041114 (6932.852628 to 8066.428964) |
| 2000 | 664.9591274 (799.138477 to 546.8160065) | 437.705102 (386.1391604 to 463.6841566) | 7326.682787 (6660.899544 to 7783.513356) |
| 2001 | 651.6957281 (781.869537 to 536.7401194) | 425.5168132 (375.3358389 to 451.6186182) | 7116.988551 (6459.236455 to 7570.038555) |
| 2002 | 637.5877412 (764.1339926 to 525.5308938) | 414.3637042 (365.2119067 to 439.9040456) | 6925.147199 (6296.590726 to 7374.152098) |
| 2003 | 622.7415302 (745.6358889 to 513.905224) | 400.9110664 (352.9575579 to 426.1136981) | 6715.843323 (6087.862414 to 7158.370211) |
| 2004 | 608.0238061 (727.5360465 to 502.436367) | 378.8406299 (331.7979047 to 403.2125594) | 6390.361945 (5779.645855 to 6826.725669) |
| 2005 | 593.3162898 (709.6478367 to 490.8569396) | 361.5987975 (316.1909794 to 385.2639079) | 6132.292272 (5540.775003 to 6561.1046) |
| 2006 | 577.1874901 (689.4110123 to 477.8615365) | 342.7957474 (298.4908617 to 365.7084035) | 5839.665288 (5264.106239 to 6251.57785) |
| 2007 | 558.8458773 (667.3400464 to 462.7509172) | 328.2148296 (285.4248398 to 350.5849317) | 5594.753476 (5031.707385 to 5996.913933) |
| 2008 | 540.6116559 (646.0184252 to 447.8304899) | 316.8343382 (274.0320812 to 339.1414319) | 5396.615274 (4830.653079 to 5791.753373) |
| 2009 | 525.0341739 (627.5572628 to 435.1129776) | 304.2086971 (263.0338586 to 325.6328692) | 5197.998054 (4654.034442 to 5587.867499) |
| 2010 | 513.7291333 (614.4255835 to 426.1906861) | 292.951806 (252.1645696 to 313.9843217) | 5021.945313 (4483.591229 to 5400.455174) |
| 2011 | 505.3130431 (603.9630669 to 419.1648387) | 283.0418663 (243.6180879 to 303.5427971) | 4865.089843 (4341.018244 to 5235.954454) |
| 2012 | 497.020422 (593.9659496 to 412.120714) | 273.5819773 (234.2031202 to 293.7740121) | 4710.276861 (4190.023685 to 5084.386654) |
| 2013 | 489.544937 (585.0869768 to 405.7652334) | 266.0627231 (227.5271809 to 286.0636929) | 4590.647517 (4074.212031 to 4960.653589) |
| 2014 | 483.8129461 (578.0639555 to 400.844759) | 259.0672708 (221.3956262 to 279.119928) | 4481.081921 (3982.301272 to 4850.00228) |
| 2015 | 480.219395 (574.1163764 to 397.3683369) | 253.8924406 (216.39639 to 273.4851543) | 4405.870714 (3911.791686 to 4765.899487) |
| 2016 | 479.0600702 (574.3035069 to 395.8015903) | 250.306718 (212.7888617 to 270.2611587) | 4356.200404 (3862.965106 to 4717.178052) |
| 2017 | 479.0329953 (576.4278892 to 394.7298372) | 245.6215589 (208.2451554 to 265.6355301) | 4288.684589 (3792.236046 to 4654.859524) |
| 2018 | 479.1523776 (578.7870682 to 393.782982) | 240.6692555 (204.0496978 to 260.4874311) | 4219.545749 (3727.72759 to 4576.042897) |
| 2019 | 478.4097786 (579.7191303 to 392.0463514) | 234.6019535 (198.5991817 to 254.6407935) | 4135.110642 (3648.163016 to 4503.248518) |
| 2020 | 471.0227928 (575.9250716 to 380.9634762) | 229.1763354 (193.4212997 to 248.9997226) | 4053.322267 (3583.062158 to 4412.918545) |
| 2021 | 471.3713532 (576.2259948 to 381.4176658) | 228.895229 (192.7626599 to 249.1957849) | 4053.493539 (3565.640461 to 4418.896623) |
| 1990 | 1243.588204 (1544.319737 to 985.8520611) | 1332.340687 (1212.986716 to 1409.368573) | 21570.87763 (19903.05291 to 22817.12333) |
| 1991 | 1237.330106 (1528.509504 to 986.8951378) | 1319.955372 (1208.829829 to 1396.896702) | 21387.76604 (19870.43333 to 22629.93244) |
| 1992 | 1229.803722 (1511.611163 to 986.9335074) | 1320.84113 (1208.2903 to 1396.142541) | 21432.6479 (19823.88622 to 22636.58346) |
| 1993 | 1221.8429 (1495.617304 to 985.7306018) | 1356.719685 (1245.462698 to 1427.670765) | 22053.73258 (20584.03096 to 23146.37193) |
| 1994 | 1213.478477 (1480.968265 to 982.2108843) | 1363.818241 (1256.197424 to 1432.571295) | 22237.28755 (20810.56795 to 23310.1039) |
| 1995 | 1205.923167 (1469.171846 to 978.586184) | 1336.280317 (1230.322939 to 1403.440078) | 21827.43411 (20391.14492 to 22851.05218) |
| 1996 | 1197.06358 (1456.045421 to 972.3457032) | 1297.754968 (1194.367104 to 1363.474403) | 21231.56449 (19822.62949 to 22259.22313) |
| 1997 | 1184.52698 (1439.688608 to 962.3999709) | 1271.67491 (1168.810278 to 1334.757836) | 20791.32963 (19421.09733 to 21748.87074) |
| 1998 | 1170.549216 (1422.229809 to 950.845609) | 1256.623583 (1151.15101 to 1323.038228) | 20496.32787 (19114.93377 to 21525.92979) |
| 1999 | 1156.436928 (1405.141291 to 938.7341591) | 1264.862545 (1165.31409 to 1332.164366) | 20634.97528 (19302.15766 to 21633.22456) |
| 2000 | 1142.829727 (1388.791092 to 926.9729696) | 1267.627315 (1171.151028 to 1331.669866) | 20709.14575 (19423.63241 to 21688.31526) |
| 2001 | 1129.329126 (1373.800189 to 916.1200385) | 1259.109279 (1158.554517 to 1324.981834) | 20578.32705 (19208.53092 to 21578.51219) |
| 2002 | 1115.696253 (1359.473444 to 904.526734) | 1253.09023 (1155.748885 to 1314.801095) | 20470.06918 (19166.68829 to 21400.69378) |
| 2003 | 1101.356341 (1343.820391 to 892.2574935) | 1254.120992 (1156.432507 to 1317.941679) | 20415.02865 (19097.78484 to 21372.96831) |
| 2004 | 1087.069369 (1327.545861 to 880.1000791) | 1217.883375 (1121.079959 to 1280.606244) | 19846.56498 (18553.54426 to 20827.40126) |
| 2005 | 1071.496319 (1308.87703 to 866.947038) | 1185.114638 (1093.785954 to 1245.310323) | 19313.23592 (18092.28933 to 20263.83732) |
| 2006 | 1051.403935 (1282.729564 to 851.0993884) | 1090.587902 (1000.772366 to 1145.521372) | 17817.50484 (16664.54491 to 18657.03323) |
| 2007 | 1027.128544 (1249.957996 to 831.782345) | 1036.779717 (948.8714403 to 1093.925904) | 16921.34029 (15779.80752 to 17804.20628) |
| 2008 | 1001.66924 (1217.89118 to 811.7121014) | 1007.383263 (917.5680559 to 1064.105971) | 16445.38077 (15243.00822 to 17339.31918) |
| 2009 | 979.3752199 (1190.039465 to 792.9770898) | 970.7241622 (887.4531335 to 1025.701972) | 15878.71662 (14766.77626 to 16716.72223) |
| 2010 | 962.5564829 (1168.662974 to 778.8267194) | 945.56967 (860.6351571 to 1003.201694) | 15481.07664 (14302.06294 to 16358.40031) |
| 2011 | 948.0388004 (1150.973695 to 767.1347867) | 895.7598927 (817.1053853 to 951.6939313) | 14716.00437 (13626.00093 to 15556.74225) |
| 2012 | 933.1047764 (1132.764978 to 755.3601358) | 857.9764273 (777.0131394 to 919.396194) | 14144.43446 (13023.02741 to 15096.20364) |
| 2013 | 920.2672532 (1116.658516 to 745.5082318) | 826.4449627 (746.3880824 to 885.5999899) | 13612.02803 (12476.62724 to 14551.87383) |
| 2014 | 912.6949263 (1107.169319 to 739.4114267) | 805.6903292 (725.3057445 to 866.8616401) | 13238.60157 (12112.60202 to 14208.89593) |
| 2015 | 912.3707547 (1106.23959 to 739.6638432) | 787.7303774 (707.3541854 to 849.9600434) | 12930.355 (11799.3803 to 13905.24686) |
| 2016 | 923.6013744 (1120.476635 to 747.5459313) | 775.5890174 (688.3557822 to 841.8353866) | 12727.47085 (11520.96694 to 13780.0436) |
| 2017 | 942.7393707 (1146.991564 to 761.5043063) | 757.1080453 (672.8130536 to 830.2504631) | 12407.09937 (11182.03879 to 13515.73017) |
| 2018 | 961.468348 (1173.312619 to 775.7538074) | 744.8637545 (657.8816526 to 815.7123455) | 12218.96616 (10975.60496 to 13373.91066) |
| 2019 | 972.0863398 (1189.481475 to 783.9665751) | 733.5645697 (644.4863455 to 806.4018391) | 12038.72078 (10752.65894 to 13196.88963) |
| 2020 | 940.218454 (1160.832984 to 750.2366723) | 725.9441631 (634.5414856 to 800.086359) | 11907.43586 (10587.33585 to 13059.36543) |
| 2021 | 944.8725063 (1171.708225 to 750.112078) | 718.0111186 (625.1379875 to 797.2417611) | 11828.11742 (10514.83263 to 13067.77705) |
| 1990 | 985.5121996 (1201.158334 to 801.2162594) | 1005.771996 (892.7825545 to 1125.868282) | 18527.67316 (16610.67917 to 20594.33521) |
| 1991 | 979.8408963 (1189.060751 to 799.6217581) | 1001.253344 (888.1165757 to 1117.634074) | 18440.31499 (16525.31705 to 20440.97485) |
| 1992 | 973.8781978 (1177.68536 to 797.5736443) | 997.7448933 (887.5072704 to 1115.892954) | 18362.8762 (16484.15592 to 20352.47731) |
| 1993 | 967.9424419 (1167.725187 to 795.4588908) | 995.6712932 (885.8877945 to 1111.74137) | 18322.29794 (16437.90676 to 20313.35532) |
| 1994 | 962.0573771 (1157.423491 to 792.7403973) | 1004.144522 (895.3081785 to 1124.27032) | 18418.98006 (16571.18774 to 20475.47365) |
| 1995 | 956.6065442 (1147.636054 to 789.194164) | 1004.919344 (893.6413986 to 1123.498088) | 18396.12843 (16507.95151 to 20430.0147) |
| 1996 | 950.8190661 (1139.521391 to 784.527855) | 989.3712881 (881.7642866 to 1103.361172) | 18164.95106 (16299.74351 to 20130.54692) |
| 1997 | 943.5309068 (1130.396982 to 778.3270307) | 976.6803284 (871.4534623 to 1086.470838) | 17989.70532 (16201.09117 to 19875.59734) |
| 1998 | 935.1969368 (1120.129155 to 770.9712348) | 968.4880988 (863.4291104 to 1080.263543) | 17874.80059 (16069.7167 to 19812.8453) |
| 1999 | 926.2413668 (1109.667726 to 763.1534971) | 945.2089604 (847.3953996 to 1052.441193) | 17420.57058 (15735.24636 to 19242.19313) |
| 2000 | 917.0969039 (1099.573309 to 755.1187764) | 932.2039747 (834.684601 to 1037.065779) | 17147.50391 (15460.22547 to 18935.52586) |
| 2001 | 907.2746985 (1087.948572 to 746.8623304) | 917.473367 (825.0579452 to 1018.779266) | 16893.54058 (15322.25998 to 18612.1603) |
| 2002 | 896.3835568 (1074.920906 to 737.7803931) | 909.7921406 (814.7916124 to 1009.502351) | 16725.29032 (15116.23522 to 18387.60851) |
| 2003 | 885.252827 (1061.711472 to 728.4317353) | 902.1139462 (809.1604851 to 997.4508434) | 16556.14785 (14984.14803 to 18166.47783) |
| 2004 | 874.2950535 (1049.917491 to 719.4057616) | 882.7120833 (792.5532551 to 976.8840854) | 16163.60026 (14642.03108 to 17749.18956) |
| 2005 | 863.6893003 (1038.034101 to 710.6263158) | 863.0191467 (778.2643072 to 954.5892023) | 15781.34288 (14374.90402 to 17334.95256) |
| 2006 | 852.4223508 (1022.188754 to 702.7103477) | 856.3093282 (772.0025249 to 948.5364074) | 15602.78082 (14188.84313 to 17161.93236) |
| 2007 | 839.7794915 (1005.122875 to 693.7270275) | 846.4601552 (762.2223504 to 936.2532461) | 15404.88364 (13999.1688 to 16905.1632) |
| 2008 | 826.9162865 (987.938917 to 683.759105) | 838.8296235 (753.8998074 to 927.7626116) | 15245.11329 (13843.8521 to 16769.79317) |
| 2009 | 814.9115281 (972.1966628 to 674.3451658) | 829.638379 (745.4838661 to 919.5009983) | 15093.6113 (13689.76496 to 16609.73756) |
| 2010 | 804.9911652 (959.9611404 to 666.2238368) | 821.1699935 (735.7391578 to 908.4629994) | 14948.83868 (13565.92455 to 16428.94963) |
| 2011 | 795.760211 (947.7138266 to 659.4917625) | 815.1068128 (728.4165559 to 904.1583223) | 14832.62208 (13399.76752 to 16347.10886) |
| 2012 | 786.5634689 (935.9199694 to 652.4393233) | 811.6284407 (726.9704405 to 901.7953669) | 14730.43233 (13348.28752 to 16224.29275) |
| 2013 | 779.4529296 (926.6503598 to 647.1812369) | 826.540574 (740.4449975 to 914.7341032) | 14778.16386 (13380.47318 to 16263.84427) |
| 2014 | 775.6338349 (922.0595963 to 644.6743771) | 831.6776365 (745.3004465 to 920.4351676) | 14750.71599 (13344.00574 to 16255.46465) |
| 2015 | 775.0798114 (921.1142665 to 644.127664) | 823.7002282 (734.8118068 to 917.9872158) | 14630.04502 (13182.82583 to 16203.34218) |
| 2016 | 778.0867121 (923.5471162 to 646.2563652) | 812.8210356 (726.3618393 to 903.7091574) | 14438.02905 (13018.49068 to 15929.2794) |
| 2017 | 782.9683528 (928.6819448 to 649.7514103) | 805.7654979 (717.9641669 to 895.6813053) | 14326.69458 (12875.45412 to 15823.51424) |
| 2018 | 787.1871119 (933.2315278 to 652.7471633) | 791.398087 (701.5740551 to 882.1088162) | 14145.62024 (12625.2977 to 15664.88083) |
| 2019 | 788.7756705 (935.6341886 to 653.8415524) | 783.1480439 (695.0466037 to 871.6694759) | 14008.51114 (12527.56617 to 15507.56616) |
| 2020 | 775.8708842 (929.6502452 to 639.1860921) | 774.0516595 (682.6125026 to 867.8521631) | 13854.87953 (12312.94718 to 15420.02974) |
| 2021 | 776.4100365 (931.6186008 to 638.2823118) | 757.9584188 (666.9315783 to 850.3752105) | 13613.90078 (12058.95306 to 15182.95996) |
| 1990 | 906.5143347 (1113.554885 to 734.009577) | 918.4870293 (827.9653364 to 1006.312846) | 16630.2177 (15112.92222 to 18120.12733) |
| 1991 | 905.0922184 (1107.839985 to 735.5190862) | 906.4860964 (819.9880755 to 989.8716892) | 16442.84818 (15022.01707 to 17820.72756) |
| 1992 | 903.4957553 (1101.949186 to 735.7865164) | 908.4127535 (822.1308538 to 988.2953017) | 16478.5458 (15064.07604 to 17823.14054) |
| 1993 | 901.5926922 (1095.733132 to 735.3262072) | 904.9861768 (820.391941 to 982.901746) | 16415.72912 (15015.25694 to 17716.59563) |
| 1994 | 899.294667 (1089.604829 to 735.1693206) | 904.6977286 (824.1510497 to 980.5986289) | 16420.03578 (15113.01486 to 17691.62345) |
| 1995 | 896.3878887 (1082.882989 to 734.3972199) | 911.3496285 (831.3860785 to 987.8280405) | 16485.05569 (15156.30459 to 17768.23837) |
| 1996 | 892.5698996 (1077.265119 to 731.7830943) | 903.7234002 (825.2062491 to 976.9533974) | 16350.73705 (15064.72183 to 17561.90455) |
| 1997 | 887.66684 (1070.9414 to 727.5617276) | 896.3019243 (818.8203663 to 967.3623753) | 16241.96275 (14974.41677 to 17433.89444) |
| 1998 | 881.9579606 (1064.133627 to 722.3455084) | 893.1832397 (816.9399692 to 963.7591908) | 16181.18577 (14930.30395 to 17361.89492) |
| 1999 | 875.4612282 (1056.436187 to 716.5624383) | 872.5195965 (799.5219228 to 939.9000615) | 15767.3936 (14595.24501 to 16891.44065) |
| 2000 | 868.2744568 (1048.552793 to 710.3819223) | 857.5459569 (787.9166027 to 921.53569) | 15501.14685 (14400.25271 to 16565.39018) |
| 2001 | 860.3627599 (1039.301209 to 703.9446363) | 855.4870118 (784.0647413 to 917.3249478) | 15460.74483 (14322.78552 to 16502.18843) |
| 2002 | 851.3307255 (1028.721535 to 696.1005096) | 855.0425249 (786.5882229 to 915.2958605) | 15438.7938 (14370.38717 to 16438.2589) |
| 2003 | 841.9412778 (1017.888182 to 687.9739966) | 854.2268186 (785.49564 to 912.8812686) | 15393.57263 (14298.94222 to 16382.80111) |
| 2004 | 832.9606753 (1007.606677 to 680.2092472) | 838.9983861 (771.18624 to 898.7047839) | 15089.80076 (14033.10432 to 16077.61767) |
| 2005 | 824.4784469 (997.6309944 to 673.4171516) | 826.5082934 (760.524698 to 884.9100216) | 14876.3463 (13822.78052 to 15868.18129) |
| 2006 | 814.9467246 (984.5084762 to 666.4808741) | 824.7966099 (758.957345 to 883.5363487) | 14820.39405 (13790.77484 to 15798.69386) |
| 2007 | 803.4310342 (967.4474586 to 658.2356528) | 827.7790156 (762.118638 to 886.5284222) | 14843.7427 (13819.70782 to 15795.48661) |
| 2008 | 791.1990075 (951.2942765 to 649.0889904) | 822.8224105 (758.3729374 to 882.2578831) | 14745.34914 (13718.88749 to 15729.19288) |
| 2009 | 779.4019045 (937.066877 to 639.7719252) | 804.7383231 (738.7023089 to 862.5030831) | 14430.54758 (13381.68001 to 15408.78339) |
| 2010 | 769.553105 (924.6106574 to 631.5797038) | 794.2723964 (729.9991084 to 853.9747415) | 14248.94035 (13220.0399 to 15198.39056) |
| 2011 | 760.125591 (912.1160784 to 624.547389) | 776.5612581 (712.2768692 to 834.2518008) | 14004.83004 (12965.47494 to 14975.8772) |
| 2012 | 750.1246579 (899.5068986 to 617.577318) | 757.7693467 (694.3619074 to 814.9419073) | 13712.74235 (12704.93452 to 14678.94186) |
| 2013 | 741.4970365 (889.3041986 to 611.4102591) | 762.6683397 (701.58507 to 821.0244259) | 13582.02706 (12612.58977 to 14530.87694) |
| 2014 | 736.1530747 (881.9021436 to 607.6803252) | 767.4110286 (703.5420417 to 825.2494485) | 13459.92786 (12480.6004 to 14379.421) |
| 2015 | 735.2332624 (879.1390027 to 607.414611) | 751.8709255 (686.8845668 to 813.5349268) | 13302.18244 (12299.07769 to 14309.56593) |
| 2016 | 738.8934123 (883.4685507 to 610.2500028) | 741.9685539 (677.6917192 to 801.2338795) | 13193.05542 (12181.5414 to 14179.68413) |
| 2017 | 744.9837356 (890.7292762 to 615.1137251) | 743.4811654 (678.7734756 to 804.0944879) | 13215.06722 (12198.5961 to 14206.70817) |
| 2018 | 751.29706 (898.0339042 to 619.744035) | 742.157637 (676.2873032 to 802.2133583) | 13234.74544 (12174.29185 to 14226.46988) |
| 2019 | 755.5138306 (903.3704187 to 622.8595351) | 735.80932 (670.0004427 to 797.1564292) | 13126.70723 (12062.13147 to 14154.3486) |
| 2020 | 740.3891485 (892.6340405 to 605.9511838) | 727.3659679 (660.5665353 to 790.6565228) | 12972.30717 (11894.90362 to 14038.37377) |
| 2021 | 742.4416509 (895.0788057 to 606.9949096) | 717.4753324 (646.3956371 to 782.3150371) | 12791.85576 (11678.10951 to 13882.88659) |
| 1990 | 1074.816434 (1328.563023 to 860.2206789) | 1215.482298 (1085.252763 to 1346.966558) | 20927.59107 (18902.07062 to 23109.33465) |
| 1991 | 1082.906871 (1334.784594 to 870.3592168) | 1199.732298 (1083.568218 to 1322.079491) | 20641.82566 (18851.63557 to 22630.57655) |
| 1992 | 1089.257026 (1338.942139 to 878.8204135) | 1197.717211 (1078.765365 to 1322.37264) | 20564.59852 (18709.3951 to 22620.38428) |
| 1993 | 1093.700996 (1340.556267 to 884.7473566) | 1190.200104 (1072.525238 to 1303.413312) | 20406.53434 (18643.98416 to 22257.93244) |
| 1994 | 1096.133713 (1340.151257 to 889.4929702) | 1183.169842 (1075.678346 to 1290.116462) | 20242.52182 (18667.65165 to 21990.1443) |
| 1995 | 1096.718962 (1338.477928 to 891.9229822) | 1176.012782 (1067.926936 to 1271.656837) | 20074.57563 (18425.14437 to 21601.00668) |
| 1996 | 1094.448101 (1335.890388 to 890.6032251) | 1163.926232 (1057.018487 to 1258.972053) | 19836.80387 (18267.62014 to 21382.64691) |
| 1997 | 1088.843881 (1330.116871 to 886.0132872) | 1142.779725 (1042.020122 to 1230.830342) | 19468.36305 (18000.12663 to 20907.88792) |
| 1998 | 1081.11092 (1322.054704 to 879.0242431) | 1129.92303 (1029.929402 to 1220.095425) | 19240.23665 (17808.64975 to 20658.63622) |
| 1999 | 1072.741867 (1313.272164 to 871.3760214) | 1115.831151 (1019.322462 to 1206.237064) | 18973.51397 (17562.9115 to 20387.78932) |
| 2000 | 1065.110589 (1305.255651 to 864.4674951) | 1118.595558 (1023.727481 to 1199.356878) | 18953.18807 (17571.98768 to 20215.38608) |
| 2001 | 1058.970305 (1298.058103 to 858.9234179) | 1120.994129 (1025.840664 to 1201.580088) | 18912.51884 (17489.80429 to 20199.03294) |
| 2002 | 1053.767686 (1292.188324 to 854.4806485) | 1114.090028 (1022.131339 to 1189.470691) | 18793.53672 (17463.21074 to 19979.48846) |
| 2003 | 1048.692763 (1286.45216 to 849.9613921) | 1117.369038 (1026.114609 to 1189.697518) | 18790.9084 (17477.74357 to 19925.42409) |
| 2004 | 1042.903134 (1279.971898 to 844.731376) | 1111.822973 (1022.351515 to 1180.542389) | 18663.91119 (17370.25247 to 19769.71042) |
| 2005 | 1036.228247 (1272.39046 to 838.8617612) | 1079.919658 (997.0449966 to 1146.508044) | 18127.55329 (16933.87324 to 19201.88903) |
| 2006 | 1026.920803 (1259.300318 to 831.6405276) | 1008.683472 (923.8209179 to 1070.890202) | 17043.36703 (15863.8861 to 18032.73374) |
| 2007 | 1014.09375 (1242.133612 to 822.2129529) | 980.8389135 (897.9858523 to 1043.805761) | 16572.52014 (15395.26162 to 17561.13369) |
| 2008 | 998.9022557 (1221.192775 to 810.4114792) | 983.2546244 (898.9958374 to 1050.061855) | 16557.78002 (15360.26152 to 17641.64049) |
| 2009 | 984.0472044 (1201.018774 to 798.7183437) | 982.6271416 (902.2623454 to 1046.351376) | 16499.05998 (15386.93352 to 17501.75402) |
| 2010 | 971.1776828 (1183.587199 to 788.7200369) | 973.7647412 (891.0382454 to 1038.680544) | 16315.62848 (15142.49655 to 17342.80905) |
| 2011 | 959.4350342 (1168.032868 to 780.7141767) | 947.1843713 (867.383976 to 1013.317543) | 15900.68316 (14693.1525 to 16949.8031) |
| 2012 | 946.7423079 (1151.127543 to 771.2076305) | 912.8831273 (830.1814796 to 979.6038157) | 15391.68351 (14166.47561 to 16490.33344) |
| 2013 | 935.6504966 (1136.455697 to 763.1329084) | 891.7934511 (808.7298019 to 962.7700424) | 15005.34454 (13817.83832 to 16140.77553) |
| 2014 | 928.9698132 (1128.300151 to 758.1631514) | 870.5682729 (788.0652759 to 942.5540581) | 14639.59581 (13428.6354 to 15759.11109) |
| 2015 | 928.053202 (1127.209916 to 757.7079348) | 851.6020711 (763.5009534 to 923.9570252) | 14360.97072 (13072.8642 to 15539.64923) |
| 2016 | 936.5595399 (1137.098334 to 764.4227485) | 841.0662372 (745.8146379 to 917.7820709) | 14192.64116 (12836.45614 to 15397.02946) |
| 2017 | 951.0221773 (1154.143962 to 775.6306566) | 823.5489224 (736.3886921 to 907.8885443) | 13926.23168 (12634.40582 to 15276.79727) |
| 2018 | 964.9479933 (1170.696549 to 785.8053711) | 809.4925246 (718.3755506 to 888.3557372) | 13714.45013 (12356.16894 to 14979.90781) |
| 2019 | 972.8432938 (1181.252571 to 791.0824558) | 800.3506768 (706.7751841 to 883.1304157) | 13557.44563 (12106.53962 to 14886.60128) |
| 2020 | 943.2741647 (1158.539368 to 760.6233241) | 792.9047083 (691.7324379 to 875.4822092) | 13428.13378 (11930.35356 to 14749.50121) |
| 2021 | 948.4471637 (1165.446448 to 764.3465342) | 785.3991733 (685.6859536 to 873.1366578) | 13315.79496 (11789.51838 to 14686.01517) |

**Supplementary Table 2-2**

| sex_name | year | Stroke_Global_Age-standardized_Rate_Incidence | Stroke_Global_Age-standardized_Rate_Deaths | Stroke_Global_Age-standardized_Rate_DALYs (Disability-Adjusted Life Years) |
| --- | --- | --- | --- | --- |
| Male | 1990 | 1090.213147(856.670391 to 1364.373282) | 1059.197687(970.4118252 to 1133.117281) | 18508.4452(17046.17057 to 19830.36816) |
| Male | 1991 | 1090.408812(861.2579437 to 1359.413082) | 1047.587829(961.7515449 to 1116.318202) | 18320.08984(16938.57961 to 19525.74117) |
| Male | 1992 | 1089.536701(864.4078483 to 1355.095833) | 1042.672713(961.3031773 to 1115.12594) | 18261.355(16936.58017 to 19547.3403) |
| Male | 1993 | 1087.586687(865.4693481 to 1349.877574) | 1045.217041(968.196363 to 1108.349287) | 18330.55616(17123.03686 to 19442.18332) |
| Male | 1994 | 1084.666317(864.2256399 to 1343.156559) | 1042.069125(970.1212108 to 1100.797832) | 18301.14964(17175.77093 to 19347.7657) |
| Male | 1995 | 1081.13747(860.912727 to 1337.499129) | 1038.508848(961.9600738 to 1096.630349) | 18226.79798(17002.51359 to 19277.88166) |
| Male | 1996 | 1074.79604(856.6792505 to 1328.308324) | 1023.523954(952.9094791 to 1083.260475) | 17961.21774(16851.63783 to 18964.81979) |
| Male | 1997 | 1064.349168(848.9944566 to 1314.552947) | 1008.259524(937.8509792 to 1061.182946) | 17692.88901(16604.68372 to 18599.98174) |
| Male | 1998 | 1051.719065(839.2558505 to 1298.98343) | 998.512356(927.9707771 to 1053.329004) | 17515.39411(16461.37521 to 18458.95271) |
| Male | 1999 | 1038.946522(829.1713635 to 1283.256443) | 988.2215287(920.0496686 to 1042.108526) | 17329.61379(16301.67329 to 18243.81188) |
| Male | 2000 | 1027.746289(820.2635439 to 1269.373542) | 987.9703656(925.1564675 to 1040.201379) | 17315.16965(16338.04158 to 18239.76599) |
| Male | 2001 | 1018.214529(812.4387591 to 1257.329018) | 986.2267662(920.1674015 to 1039.558055) | 17250.11905(16241.00135 to 18156.99821) |
| Male | 2002 | 1009.03147(804.6286066 to 1246.104834) | 979.5116624(914.8940106 to 1030.683482) | 17132.44615(16131.56607 to 18026.25769) |
| Male | 2003 | 999.5368769(796.5264902 to 1234.685346) | 983.8885373(920.8263153 to 1033.80304) | 17133.13804(16164.66001 to 17974.49784) |
| Male | 2004 | 989.9282796(788.538706 to 1223.130441) | 968.7446754(904.0383129 to 1018.381091) | 16865.05318(15856.54572 to 17742.34148) |
| Male | 2005 | 979.9902647(780.909103 to 1210.966119) | 942.8496543(881.5135667 to 989.894076) | 16429.51264(15510.64471 to 17253.99714) |
| Male | 2006 | 968.1634105(771.8178941 to 1196.099185) | 889.4597633(829.6863879 to 936.5120001) | 15568.02445(14685.73749 to 16406.70133) |
| Male | 2007 | 953.4149411(761.1024999 to 1177.112126) | 866.9299802(807.1542989 to 912.1045463) | 15159.97743(14260.15293 to 15954.79609) |
| Male | 2008 | 937.3636099(748.7135369 to 1155.127127) | 858.4772858(798.7920863 to 907.6732772) | 15006.44314(14102.18068 to 15841.02833) |
| Male | 2009 | 922.971822(737.4862068 to 1136.673668) | 846.8144466(787.4502603 to 893.497868) | 14793.44946(13879.81195 to 15595.83789) |
| Male | 2010 | 911.4266271(728.5640605 to 1121.843778) | 837.0692317(774.1037333 to 884.4849036) | 14615.25482(13662.18024 to 15435.39139) |
| Male | 2011 | 901.6366975(721.3935616 to 1108.282254) | 811.866457(752.1521891 to 864.8926266) | 14220.45319(13262.07708 to 15134.64597) |
| Male | 2012 | 890.8644811(713.6024994 to 1093.124205) | 788.7601767(728.5810247 to 840.4539101) | 13841.37315(12888.91767 to 14746.33022) |
| Male | 2013 | 881.1675069(706.5365227 to 1079.722194) | 776.4440217(712.8584487 to 832.23444) | 13558.43319(12569.03359 to 14530.68889) |
| Male | 2014 | 875.267466(702.2565016 to 1071.650864) | 763.6362632(701.8040928 to 822.279999) | 13294.30446(12327.9729 to 14268.18595) |
| Male | 2015 | 874.2168602(701.457876 to 1070.243685) | 749.6287195(683.9178708 to 805.6646091) | 13080.8683(12072.20714 to 14038.71667) |
| Male | 2016 | 879.7600605(705.6988954 to 1077.229) | 741.1236093(670.8505833 to 803.5182879) | 12946.48095(11906.26554 to 13973.9318) |
| Male | 2017 | 888.1251756(711.9922702 to 1088.375962) | 728.2258592(658.2260225 to 796.0184841) | 12736.48238(11615.77306 to 13869.40965) |
| Male | 2018 | 895.8837478(717.7971535 to 1099.316458) | 717.8809595(646.9387109 to 783.6282884) | 12586.60402(11487.54155 to 13690.68068) |
| Male | 2019 | 900.7177429(721.1437737 to 1106.396998) | 711.0347353(638.8953198 to 780.2676295) | 12469.31689(11299.50768 to 13636.74312) |
| Male | 2020 | 875.4215868(694.7045905 to 1087.943013) | 706.7771332(628.5963564 to 775.5483019) | 12382.09232(11123.9442 to 13570.79791) |
| Male | 2021 | 879.2319599(696.2634122 to 1093.140146) | 701.297769(625.77378 to 775.8006318) | 12285.55144(11059.77702 to 13521.39449) |
| Female | 1990 | 916.1916894(721.5638162 to 1148.245214) | 919.165911(824.4807794 to 991.0587698) | 15376.41268(14031.57204 to 16569.01071) |
| Female | 1991 | 909.2082832(719.7256281 to 1134.153453) | 904.1609498(812.5657424 to 972.5761014) | 15151.26177(13840.47312 to 16219.08654) |
| Female | 1992 | 901.6454576(716.7898869 to 1120.901156) | 897.072879(804.1088794 to 965.7797147) | 15049.22549(13750.54163 to 16147.19255) |
| Female | 1993 | 894.0730081(713.980589 to 1107.107682) | 900.9228495(810.360249 to 969.0319663) | 15113.16531(13855.43277 to 16215.75219) |
| Female | 1994 | 886.7957944(710.7056237 to 1094.2445) | 899.0169947(807.3998188 to 962.2986879) | 15095.05449(13813.2763 to 16116.74443) |
| Female | 1995 | 880.130676(707.7546398 to 1082.575126) | 888.1113935(800.9133966 to 950.4775041) | 14907.92706(13706.24384 to 15891.05981) |
| Female | 1996 | 872.5710942(702.7281568 to 1072.01533) | 868.3954074(782.4213045 to 929.6378002) | 14601.53246(13450.63925 to 15584.53055) |
| Female | 1997 | 862.1058519(694.7824533 to 1058.615121) | 849.5493864(764.305119 to 907.4737189) | 14313.34145(13170.10899 to 15212.24386) |
| Female | 1998 | 850.1724492(685.077691 to 1044.039989) | 836.7961569(753.948852 to 891.9517033) | 14111.6688(13007.3921 to 14985.8441) |
| Female | 1999 | 838.184744(675.0320762 to 1029.628786) | 831.1671136(750.4750907 to 887.1260508) | 13998.69138(12901.77665 to 14876.53786) |
| Female | 2000 | 826.8432635(665.4985734 to 1015.796504) | 819.5003637(738.383223 to 874.2965594) | 13807.80407(12721.75185 to 14627.58988) |
| Female | 2001 | 816.0698236(656.7352635 to 1002.724877) | 811.0662008(731.8385799 to 865.9255865) | 13661.32404(12562.45378 to 14508.55501) |
| Female | 2002 | 804.6363138(647.2338362 to 988.9916708) | 804.5395434(724.2561366 to 854.3977981) | 13547.89482(12469.56147 to 14336.22306) |
| Female | 2003 | 792.6393277(637.59219 to 974.6088026) | 795.6196961(715.4543187 to 845.9347244) | 13395.08418(12305.30033 to 14181.91857) |
| Female | 2004 | 780.6465722(627.9901094 to 960.4300301) | 771.7690149(694.1389559 to 821.4693795) | 13010.7755(11954.28703 to 13805.73942) |
| Female | 2005 | 768.2302257(617.8399797 to 945.4398374) | 750.010305(675.0381714 to 798.0995473) | 12663.30596(11673.40242 to 13398.90824) |
| Female | 2006 | 753.9131218(606.8538355 to 926.1039238) | 709.9589859(637.087339 to 754.9532536) | 12040.46025(11089.0269 to 12750.04486) |
| Female | 2007 | 736.6833692(593.1009841 to 903.2965445) | 685.3558105(613.8468831 to 732.6509925) | 11624.09768(10675.93029 to 12343.80454) |
| Female | 2008 | 718.7079292(578.717244 to 880.2702821) | 672.8106174(599.3401079 to 718.9584999) | 11381.11724(10409.17828 to 12070.80718) |
| Female | 2009 | 702.9092786(566.3650695 to 860.6696826) | 655.1837553(583.281213 to 700.5705647) | 11088.36217(10143.06914 to 11785.29201) |
| Female | 2010 | 690.8570388(557.0216391 to 846.2859521) | 640.0057915(570.0873352 to 684.2593106) | 10833.28375(9871.329498 to 11508.66276) |
| Female | 2011 | 681.2724984(549.5308298 to 835.2188837) | 619.0337133(550.9846334 to 664.2521343) | 10506.47325(9587.691343 to 11203.65323) |
| Female | 2012 | 671.4084634(541.5503402 to 823.8560517) | 598.0784234(528.9848155 to 643.5847539) | 10188.36312(9258.652196 to 10906.14332) |
| Female | 2013 | 662.937583(534.752116 to 813.6265852) | 582.5444644(515.2739124 to 628.4745277) | 9900.117535(8989.783973 to 10631.0073) |
| Female | 2014 | 657.7952259(530.2705378 to 807.5634004) | 571.4148856(504.8524789 to 616.1343983) | 9680.252391(8794.607971 to 10398.59373) |
| Female | 2015 | 657.0077298(529.1565948 to 805.9715953) | 561.012116(491.8672719 to 607.1037964) | 9527.721268(8596.724746 to 10262.55927) |
| Female | 2016 | 663.9895436(534.6046009 to 814.5667524) | 554.817018(487.3809135 to 603.7223235) | 9433.709131(8522.253881 to 10190.68395) |
| Female | 2017 | 676.4059769(544.0590615 to 830.385126) | 547.474681(478.6673103 to 597.6338554) | 9317.705627(8374.870752 to 10081.29688) |
| Female | 2018 | 688.7238381(552.8711279 to 846.8915052) | 541.1018509(473.1726194 to 592.2775635) | 9230.8904(8256.522269 to 10055.8021) |
| Female | 2019 | 695.8613136(557.6936082 to 857.0784512) | 533.1772775(462.0845434 to 585.6728604) | 9109.033974(8098.404788 to 9959.910293) |
| Female | 2020 | 679.9134002(542.1274554 to 845.6122397) | 525.3340139(456.048154 to 581.4980074) | 8982.853925(7999.048706 to 9840.682543) |
| Female | 2021 | 683.6444101(544.9863882 to 853.1835689) | 520.2805585(448.6406752 to 577.8471035) | 8927.199956(7905.375116 to 9803.706617) |
| Both | 1990 | 996.0622861(798.7445574 to 1230.657419) | 981.8654905(895.7902475 to 1042.223249) | 16791.36547(15558.89798 to 17794.67313) |
| Both | 1991 | 992.3834594(799.5943611 to 1221.084881) | 968.1872997(885.9246043 to 1025.828491) | 16583.15346(15416.26429 to 17514.85305) |
| Both | 1992 | 987.8790876(798.6780837 to 1210.405144) | 962.1788425(879.2205165 to 1022.584024) | 16503.80419(15321.76216 to 17489.50302) |
| Both | 1993 | 982.8898655(797.0303982 to 1200.455815) | 965.9044016(884.31175 to 1021.700062) | 16577.29861(15456.65068 to 17468.60054) |
| Both | 1994 | 977.6504582(795.7233327 to 1190.189837) | 963.759597(884.936341 to 1016.950238) | 16559.63159(15483.85327 to 17420.17856) |
| Both | 1995 | 972.4654575(793.0765464 to 1180.574312) | 955.9157799(876.1378137 to 1006.270658) | 16422.18961(15302.19042 to 17228.90045) |
| Both | 1996 | 965.5711434(788.5222511 to 1170.878866) | 937.8743027(860.714791 to 988.7247557) | 16130.52857(15050.13719 to 16946.51197) |
| Both | 1997 | 955.195419(780.6734982 to 1157.512346) | 920.0810764(844.7670984 to 966.7622085) | 15846.84757(14812.78017 to 16607.78918) |
| Both | 1998 | 943.041926(771.1116946 to 1142.027984) | 908.4060647(834.0983243 to 957.4194965) | 15653.77149(14662.13745 to 16427.39564) |
| Both | 1999 | 930.8296275(761.1421502 to 1126.622544) | 901.1412365(827.7104589 to 950.084348) | 15513.16134(14546.73961 to 16301.49551) |
| Both | 2000 | 919.6442777(752.0678145 to 1112.583765) | 894.2000605(823.1308174 to 939.485175) | 15399.11631(14444.7625 to 16140.21799) |
| Both | 2001 | 909.5180653(743.8192393 to 1100.81981) | 888.4055964(817.8029982 to 934.7952197) | 15286.10394(14312.81686 to 16032.48685) |
| Both | 2002 | 899.1533299(735.0269175 to 1088.925458) | 881.9356451(811.2910045 to 925.4111094) | 15172.69364(14197.86506 to 15887.69655) |
| Both | 2003 | 888.3718422(725.8858714 to 1076.625653) | 877.9997761(807.4754145 to 919.8196509) | 15080.56084(14129.63191 to 15746.81305) |
| Both | 2004 | 877.58703(716.7725317 to 1063.945874) | 857.7224866(789.0120767 to 899.9795561) | 14746.35693(13798.04763 to 15431.09322) |
| Both | 2005 | 866.3881774(707.3153205 to 1050.828411) | 834.2514092(768.6241737 to 876.4761711) | 14360.89598(13489.56692 to 15043.69948) |
| Both | 2006 | 853.2630924(696.6853314 to 1033.631141) | 788.5566975(722.2626789 to 827.9086761) | 13633.88573(12774.36104 to 14297.46803) |
| Both | 2007 | 837.1764395(683.5068461 to 1012.537995) | 764.6216977(700.5714765 to 804.746985) | 13220.18322(12367.35825 to 13858.92827) |
| Both | 2008 | 820.0986307(669.6693305 to 990.2259771) | 754.1112506(689.144498 to 796.4012883) | 13022.02543(12150.29874 to 13705.46661) |
| Both | 2009 | 805.0037603(657.6575024 to 970.8410982) | 738.9927874(676.9858279 to 778.9780193) | 12765.12805(11928.40702 to 13402.33797) |
| Both | 2010 | 793.2082399(648.5584362 to 956.1700826) | 726.1803192(663.2633286 to 766.3291816) | 12544.83464(11679.03031 to 13197.67217) |
| Both | 2011 | 783.568348(641.3140342 to 944.2818546) | 703.5520418(644.9344444 to 744.0512661) | 12190.19719(11350.87134 to 12828.83279) |
| Both | 2012 | 773.3222072(633.3340905 to 931.348981) | 681.5729305(619.5015454 to 723.718535) | 11843.97889(10974.67611 to 12539.39031) |
| Both | 2013 | 764.3595568(626.0945544 to 920.4294591) | 667.1747646(606.1124625 to 709.4925926) | 11554.78322(10692.4008 to 12243.82257) |
| Both | 2014 | 758.9662427(621.8204451 to 914.1522629) | 655.3544899(593.3027647 to 699.0317387) | 11315.4814(10458.20142 to 12002.79803) |
| Both | 2015 | 758.1614495(621.1535954 to 913.2599231) | 643.5774695(582.2473945 to 686.4571336) | 11137.99136(10280.0554 to 11862.37419) |
| Both | 2016 | 764.6489754(626.1282319 to 921.6017816) | 636.635292(570.7185385 to 683.0939844) | 11028.62391(10148.71358 to 11760.50072) |
| Both | 2017 | 775.3941423(634.1476675 to 935.4248628) | 627.2139082(564.6581321 to 677.2157812) | 10873.74816(9965.391528 to 11649.49172) |
| Both | 2018 | 785.8336405(641.7292763 to 949.2043876) | 619.4025795(555.1615672 to 666.44394) | 10761.79655(9843.268817 to 11534.70971) |
| Both | 2019 | 792.0944697(645.9522636 to 957.9591579) | 611.9729522(546.4445085 to 659.7105895) | 10642.33399(9694.704843 to 11429.52317) |
| Both | 2020 | 771.8979105(622.6602571 to 942.1336015) | 605.5400282(537.713038 to 655.009167) | 10531.56637(9580.416413 to 11318.81924) |
| Both | 2021 | 775.675428(624.5969667 to 947.9602221) | 600.0754178(531.6329648 to 653.2235085) | 10454.28207(9462.831603 to 11305.68062) |
| Male | 1990 | 916.6821173(716.9759818 to 1156.015904) | 634.7442975(590.0930202 to 660.4665717) | 10689.8007(10071.97466 to 11183.03268) |
| Male | 1991 | 904.3586894(710.9915485 to 1136.325051) | 617.6642709(573.4696062 to 643.0710958) | 10437.79193(9814.17734 to 10922.92295) |
| Male | 1992 | 892.1983778(702.7871487 to 1115.164192) | 598.5012968(555.139674 to 623.5124644) | 10145.59454(9535.530995 to 10626.80398) |
| Male | 1993 | 880.1942426(694.9259845 to 1095.933978) | 584.9469396(543.1626776 to 608.7100296) | 9939.604373(9354.63175 to 10396.26628) |
| Male | 1994 | 869.3454796(688.0475951 to 1079.271449) | 574.8706264(533.6486657 to 599.1817408) | 9784.536416(9191.84998 to 10249.36346) |
| Male | 1995 | 859.8325005(682.9977041 to 1062.977435) | 575.3003625(533.6374464 to 599.416641) | 9772.687513(9174.118474 to 10219.78411) |
| Male | 1996 | 847.3292768(675.5630676 to 1046.348994) | 559.123556(517.1417689 to 582.7751812) | 9512.292501(8913.799869 to 9966.50765) |
| Male | 1997 | 829.1756918(662.5052824 to 1022.119103) | 539.9089173(498.563355 to 562.7982387) | 9223.50055(8634.139132 to 9681.639335) |
| Male | 1998 | 808.0575123(647.1761853 to 993.8354608) | 523.8205479(483.9811801 to 546.4479208) | 8988.906469(8411.097748 to 9430.048621) |
| Male | 1999 | 787.6725906(631.9401635 to 966.6089206) | 513.6413877(474.020256 to 536.2599118) | 8822.001606(8268.05902 to 9263.154226) |
| Male | 2000 | 771.0420432(619.4576722 to 944.5455353) | 493.4585141(455.2194191 to 514.7929416) | 8506.590888(7950.85315 to 8933.227001) |
| Male | 2001 | 756.9922766(609.2686464 to 925.4016016) | 479.2469492(441.9940827 to 501.4339788) | 8260.888684(7728.453923 to 8708.938418) |
| Male | 2002 | 741.9835579(597.9810785 to 905.5363672) | 465.8946434(429.8537411 to 486.8568608) | 8031.672678(7488.688555 to 8456.78645) |
| Male | 2003 | 725.9016274(586.0007558 to 884.7396627) | 451.2132756(415.4600422 to 471.760796) | 7800.358212(7286.52161 to 8236.942987) |
| Male | 2004 | 709.8363859(573.8618907 to 864.1246089) | 425.6641034(390.283942 to 445.9795278) | 7420.367316(6907.345272 to 7845.822926) |
| Male | 2005 | 693.3767133(561.6512827 to 843.9481494) | 407.5231167(373.3168576 to 427.0619975) | 7137.30001(6642.46886 to 7553.067266) |
| Male | 2006 | 675.0992789(546.7943241 to 820.6367113) | 386.0183477(352.3664771 to 404.7674601) | 6796.973122(6312.375883 to 7196.911703) |
| Male | 2007 | 654.1805202(529.4533187 to 795.8525796) | 369.1105521(336.5672249 to 387.4308423) | 6515.088783(6044.281748 to 6919.216072) |
| Male | 2008 | 633.2021151(512.3572099 to 770.8117316) | 355.3149745(323.0142579 to 373.1277581) | 6280.176457(5808.322262 to 6665.630356) |
| Male | 2009 | 615.1332873(497.9456782 to 749.1064471) | 341.5116937(311.1551686 to 358.8539862) | 6057.213244(5602.387167 to 6437.486571) |
| Male | 2010 | 601.5878915(487.0906389 to 732.9921543) | 330.2939734(299.1610376 to 347.7247832) | 5873.596164(5410.638235 to 6253.58263) |
| Male | 2011 | 591.1911163(477.9969778 to 720.0106726) | 319.0266976(289.8086836 to 335.7471874) | 5692.151982(5236.721056 to 6056.745123) |
| Male | 2012 | 580.9879003(469.7143903 to 707.3323591) | 307.7766045(278.0233063 to 324.694925) | 5506.257778(5046.849053 to 5881.85309) |
| Male | 2013 | 571.6345749(462.0085703 to 695.9536443) | 299.2854189(270.2315825 to 315.9816866) | 5368.918043(4923.511453 to 5731.259343) |
| Male | 2014 | 564.1939934(455.6971902 to 687.0103738) | 290.7266054(262.350224 to 308.4211128) | 5230.809851(4797.122428 to 5592.506144) |
| Male | 2015 | 558.9648221(450.9084335 to 681.5965115) | 285.199444(256.8971252 to 301.6587498) | 5146.012141(4720.0911 to 5510.725451) |
| Male | 2016 | 555.9734907(447.7823722 to 679.4027245) | 281.2598159(251.6839369 to 298.8233914) | 5087.90554(4644.686378 to 5443.760114) |
| Male | 2017 | 553.7213676(444.8793416 to 678.4210219) | 277.0310092(248.5351006 to 295.0520906) | 5018.090042(4565.452023 to 5385.191402) |
| Male | 2018 | 551.5933165(441.5907036 to 678.0776435) | 271.4723891(242.9536897 to 289.2525168) | 4935.94752(4496.23672 to 5301.082218) |
| Male | 2019 | 549.1022539(438.4193766 to 676.976754) | 265.2669699(236.5507359 to 283.3358651) | 4843.282099(4394.40003 to 5232.067321) |
| Male | 2020 | 539.4629411(426.4175364 to 670.7693627) | 259.9621996(230.8703885 to 277.7570076) | 4755.523896(4316.798348 to 5132.185293) |
| Male | 2021 | 539.7364681(426.1520743 to 674.2557448) | 259.7758594(230.794329 to 278.5940537) | 4747.908786(4305.3376 to 5133.17811) |
| Female | 1990 | 710.5580331(556.2567148 to 892.8981085) | 526.5863494(458.54375 to 561.8186949) | 8358.851397(7518.400613 to 8892.247771) |
| Female | 1991 | 699.2932521(551.0863981 to 875.1347683) | 508.955087(442.9643913 to 543.0100446) | 8109.055109(7289.025585 to 8628.185506) |
| Female | 1992 | 687.9399337(544.2652127 to 857.5737621) | 491.3159626(426.3696284 to 524.4993923) | 7854.337394(7046.978637 to 8374.373371) |
| Female | 1993 | 676.6204571(537.6500408 to 839.852044) | 480.4327219(415.8736631 to 513.4078075) | 7688.566372(6900.537787 to 8198.605838) |
| Female | 1994 | 666.2373796(531.0792645 to 823.9864967) | 469.6938337(405.4666045 to 501.8201059) | 7527.513006(6746.121041 to 8045.527156) |
| Female | 1995 | 656.9345188(524.8714499 to 810.4232633) | 464.8547773(401.2495201 to 497.2643395) | 7447.401813(6661.640755 to 7956.444451) |
| Female | 1996 | 645.5228921(517.52783 to 794.6822159) | 451.2215109(388.0671443 to 483.3331778) | 7235.165698(6466.773488 to 7738.625747) |
| Female | 1997 | 630.0487465(505.9042712 to 773.8458733) | 435.9081489(374.4697558 to 467.3738974) | 7013.670961(6253.423583 to 7507.010864) |
| Female | 1998 | 612.3573261(492.7373129 to 750.1048631) | 422.9234833(362.4838716 to 453.5486139) | 6828.259466(6077.10916 to 7311.794515) |
| Female | 1999 | 595.0292018(479.6920939 to 726.9627714) | 414.2333911(355.4228012 to 444.9357053) | 6690.624669(5949.692985 to 7174.824348) |
| Female | 2000 | 580.246969(467.8302226 to 707.7338925) | 397.4106465(339.3626827 to 427.3399115) | 6435.098297(5710.110128 to 6910.093969) |
| Female | 2001 | 567.1511058(458.2063492 to 691.0141289) | 386.2574427(328.8793807 to 415.7853528) | 6245.207053(5516.921895 to 6716.788296) |
| Female | 2002 | 553.3615098(447.9269373 to 673.8367237) | 376.2379185(320.5269416 to 405.7485825) | 6074.031013(5376.830551 to 6541.948753) |
| Female | 2003 | 539.1236517(437.0373235 to 656.1694351) | 363.2970932(308.1264132 to 392.3656335) | 5874.614738(5182.712709 to 6337.661472) |
| Female | 2004 | 525.0913783(425.8960944 to 637.9192266) | 343.0863409(289.9725253 to 370.9570524) | 5580.590736(4913.35369 to 6031.706313) |
| Female | 2005 | 511.3664815(414.7364698 to 620.3184865) | 326.3914475(274.8465661 to 353.5892836) | 5338.271644(4691.629017 to 5779.866957) |
| Female | 2006 | 496.5318929(403.379256 to 602.2348706) | 309.1674225(259.6738696 to 335.509065) | 5075.893218(4448.914199 to 5509.177283) |
| Female | 2007 | 479.8576469(390.181215 to 581.4390772) | 295.7396559(247.3858098 to 321.4232318) | 4852.087837(4238.91094 to 5264.496241) |
| Female | 2008 | 463.4544291(377.1947624 to 560.7175918) | 285.5672408(237.7845157 to 311.2466156) | 4674.59836(4065.500207 to 5082.874364) |
| Female | 2009 | 449.5443785(366.3615667 to 543.9145339) | 273.6670291(226.726621 to 298.5746754) | 4491.404558(3907.120844 to 4888.346213) |
| Female | 2010 | 439.7222068(358.3546143 to 531.921698) | 262.3131382(216.1216817 to 286.5634656) | 4319.222513(3742.402434 to 4716.987311) |
| Female | 2011 | 432.6078383(352.5799877 to 523.5688725) | 253.1974599(208.4845358 to 277.0235949) | 4177.546945(3610.467023 to 4565.249636) |
| Female | 2012 | 425.5845394(346.712007 to 515.2007056) | 244.8987571(200.3831921 to 268.5754475) | 4043.522814(3488.213606 to 4425.503205) |
| Female | 2013 | 419.3716517(341.5038785 to 507.7416569) | 237.8682545(194.3391807 to 261.2416203) | 3934.237497(3382.971668 to 4316.716522) |
| Female | 2014 | 414.7752485(337.4411339 to 502.026125) | 231.8281977(188.6254702 to 254.5489241) | 3844.227959(3312.524107 to 4218.812817) |
| Female | 2015 | 412.2532309(335.3882931 to 499.0345107) | 226.8193224(183.794663 to 249.6056619) | 3774.124986(3240.965211 to 4148.035201) |
| Female | 2016 | 412.3717796(335.0152466 to 499.483359) | 223.3392601(181.1534989 to 246.3922151) | 3728.24986(3214.77678 to 4098.285083) |
| Female | 2017 | 413.9836395(335.667097 to 502.9176096) | 218.4217525(176.3441201 to 241.4627229) | 3662.755543(3138.187226 to 4032.074135) |
| Female | 2018 | 415.7738923(336.3384302 to 506.4469438) | 213.8498585(172.7038456 to 236.8358982) | 3602.036242(3081.402136 to 3970.58587) |
| Female | 2019 | 416.292156(336.1876886 to 508.3662707) | 207.9705688(167.2099738 to 230.6380389) | 3524.040755(3018.075233 to 3892.212486) |
| Female | 2020 | 410.6130165(328.5209383 to 507.4217537) | 202.6120022(162.208546 to 225.580465) | 3448.200832(2939.176331 to 3809.011341) |
| Female | 2021 | 410.9587086(329.6012841 to 508.6797619) | 202.3224608(161.6474043 to 225.6742812) | 3454.724223(2950.503272 to 3822.47239) |
| Both | 1990 | 798.3396962(634.2812576 to 990.2266466) | 570.3618717(509.9157523 to 600.799517) | 9325.450121(8577.328638 to 9831.741977) |
| Both | 1991 | 786.9815013(628.1139883 to 971.2994704) | 552.8523811(493.6590598 to 582.9185696) | 9076.443244(8330.797141 to 9564.503053) |
| Both | 1992 | 775.6404228(622.195265 to 953.7833293) | 534.5889494(476.3199697 to 563.5034876) | 8808.332894(8078.961007 to 9290.856239) |
| Both | 1993 | 764.3883672(615.4040127 to 936.3211223) | 522.9214474(465.4479618 to 551.4598281) | 8630.932878(7920.23091 to 9104.217996) |
| Both | 1994 | 754.1744384(609.4327141 to 920.3577838) | 512.530427(455.7407644 to 540.9585745) | 8475.272811(7768.913237 to 8959.974203) |
| Both | 1995 | 745.1438652(604.3243046 to 906.516668) | 509.5766115(452.6590819 to 538.2781414) | 8423.123145(7704.356194 to 8900.736264) |
| Both | 1996 | 733.6207393(597.2359967 to 889.8549067) | 495.1076231(438.78858 to 522.9598494) | 8194.326881(7486.566486 to 8670.005632) |
| Both | 1997 | 717.3657463(585.9309585 to 867.9932709) | 478.4833705(423.5775612 to 506.1374686) | 7949.347529(7256.330205 to 8423.510267) |
| Both | 1998 | 698.564769(571.6337128 to 843.2088719) | 464.6110467(411.189258 to 491.547902) | 7748.855247(7060.311621 to 8205.660266) |
| Both | 1999 | 680.250184(557.9274633 to 819.4720336) | 455.6007722(403.2926187 to 482.5280308) | 7603.041114(6932.852628 to 8066.428964) |
| Both | 2000 | 664.9591274(546.8160065 to 799.138477) | 437.705102(386.1391604 to 463.6841566) | 7326.682787(6660.899544 to 7783.513356) |
| Both | 2001 | 651.6957281(536.7401194 to 781.869537) | 425.5168132(375.3358389 to 451.6186182) | 7116.988551(6459.236455 to 7570.038555) |
| Both | 2002 | 637.5877412(525.5308938 to 764.1339926) | 414.3637042(365.2119067 to 439.9040456) | 6925.147199(6296.590726 to 7374.152098) |
| Both | 2003 | 622.7415302(513.905224 to 745.6358889) | 400.9110664(352.9575579 to 426.1136981) | 6715.843323(6087.862414 to 7158.370211) |
| Both | 2004 | 608.0238061(502.436367 to 727.5360465) | 378.8406299(331.7979047 to 403.2125594) | 6390.361945(5779.645855 to 6826.725669) |
| Both | 2005 | 593.3162898(490.8569396 to 709.6478367) | 361.5987975(316.1909794 to 385.2639079) | 6132.292272(5540.775003 to 6561.1046) |
| Both | 2006 | 577.1874901(477.8615365 to 689.4110123) | 342.7957474(298.4908617 to 365.7084035) | 5839.665288(5264.106239 to 6251.57785) |
| Both | 2007 | 558.8458773(462.7509172 to 667.3400464) | 328.2148296(285.4248398 to 350.5849317) | 5594.753476(5031.707385 to 5996.913933) |
| Both | 2008 | 540.6116559(447.8304899 to 646.0184252) | 316.8343382(274.0320812 to 339.1414319) | 5396.615274(4830.653079 to 5791.753373) |
| Both | 2009 | 525.0341739(435.1129776 to 627.5572628) | 304.2086971(263.0338586 to 325.6328692) | 5197.998054(4654.034442 to 5587.867499) |
| Both | 2010 | 513.7291333(426.1906861 to 614.4255835) | 292.951806(252.1645696 to 313.9843217) | 5021.945313(4483.591229 to 5400.455174) |
| Both | 2011 | 505.3130431(419.1648387 to 603.9630669) | 283.0418663(243.6180879 to 303.5427971) | 4865.089843(4341.018244 to 5235.954454) |
| Both | 2012 | 497.020422(412.120714 to 593.9659496) | 273.5819773(234.2031202 to 293.7740121) | 4710.276861(4190.023685 to 5084.386654) |
| Both | 2013 | 489.544937(405.7652334 to 585.0869768) | 266.0627231(227.5271809 to 286.0636929) | 4590.647517(4074.212031 to 4960.653589) |
| Both | 2014 | 483.8129461(400.844759 to 578.0639555) | 259.0672708(221.3956262 to 279.119928) | 4481.081921(3982.301272 to 4850.00228) |
| Both | 2015 | 480.219395(397.3683369 to 574.1163764) | 253.8924406(216.39639 to 273.4851543) | 4405.870714(3911.791686 to 4765.899487) |
| Both | 2016 | 479.0600702(395.8015903 to 574.3035069) | 250.306718(212.7888617 to 270.2611587) | 4356.200404(3862.965106 to 4717.178052) |
| Both | 2017 | 479.0329953(394.7298372 to 576.4278892) | 245.6215589(208.2451554 to 265.6355301) | 4288.684589(3792.236046 to 4654.859524) |
| Both | 2018 | 479.1523776(393.782982 to 578.7870682) | 240.6692555(204.0496978 to 260.4874311) | 4219.545749(3727.72759 to 4576.042897) |
| Both | 2019 | 478.4097786(392.0463514 to 579.7191303) | 234.6019535(198.5991817 to 254.6407935) | 4135.110642(3648.163016 to 4503.248518) |
| Both | 2020 | 471.0227928(380.9634762 to 575.9250716) | 229.1763354(193.4212997 to 248.9997226) | 4053.322267(3583.062158 to 4412.918545) |
| Both | 2021 | 471.3713532(381.4176658 to 576.2259948) | 228.895229(192.7626599 to 249.1957849) | 4053.493539(3565.640461 to 4418.896623) |
| Male | 1990 | 1376.77936(1076.307264 to 1732.77602) | 1485.276295(1354.616496 to 1596.388417) | 24679.24239(22522.27045 to 26667.42253) |
| Male | 1991 | 1377.841195(1083.488513 to 1728.676189) | 1472.715516(1348.135542 to 1580.220543) | 24479.09148(22543.00905 to 26379.81581) |
| Male | 1992 | 1377.257162(1089.078957 to 1721.708919) | 1474.612886(1352.746145 to 1579.932322) | 24578.83177(22665.75948 to 26416.80759) |
| Male | 1993 | 1375.338571(1093.12738 to 1714.453448) | 1499.64618(1384.390174 to 1594.994697) | 25097.49174(23339.58566 to 26749.03339) |
| Male | 1994 | 1371.542291(1091.525327 to 1705.07895) | 1497.816849(1389.058961 to 1587.774552) | 25184.37784(23473.81841 to 26762.93561) |
| Male | 1995 | 1367.176694(1089.818892 to 1697.021999) | 1474.496921(1365.894663 to 1561.987052) | 24819.06096(23154.78633 to 26303.7862) |
| Male | 1996 | 1358.777337(1082.899471 to 1685.577401) | 1438.424485(1332.929945 to 1523.8455) | 24214.33007(22581.88984 to 25617.4466) |
| Male | 1997 | 1345.23731(1071.547151 to 1668.012965) | 1410.479183(1308.477156 to 1488.397013) | 23716.23169(22161.04644 to 25016.15318) |
| Male | 1998 | 1329.461951(1058.576731 to 1646.783116) | 1397.740628(1294.183853 to 1482.070929) | 23429.26153(21867.97128 to 24826.24596) |
| Male | 1999 | 1313.434491(1045.131567 to 1626.824494) | 1402.585061(1299.775036 to 1486.265819) | 23547.64693(22025.2014 to 24883.02978) |
| Male | 2000 | 1299.499007(1033.125675 to 1609.956918) | 1421.276818(1324.282828 to 1509.633386) | 23861.86234(22376.14937 to 25309.42811) |
| Male | 2001 | 1287.051682(1023.045624 to 1594.088512) | 1422.714395(1320.985114 to 1509.847209) | 23826.7126(22301.90697 to 25225.11844) |
| Male | 2002 | 1275.807868(1013.169364 to 1581.977296) | 1422.30418(1324.650012 to 1509.252002) | 23799.68996(22296.08637 to 25199.9312) |
| Male | 2003 | 1264.254123(1002.990166 to 1569.866715) | 1441.205317(1341.365721 to 1528.717243) | 23914.38472(22401.70008 to 25301.87836) |
| Male | 2004 | 1252.092627(993.3323799 to 1555.973888) | 1420.10035(1317.057944 to 1509.485192) | 23498.86832(21965.091 to 24939.55292) |
| Male | 2005 | 1238.684819(982.6402587 to 1539.60869) | 1386.084474(1286.856684 to 1468.359205) | 22900.94219(21444.43292 to 24260.01631) |
| Male | 2006 | 1220.551808(969.6136762 to 1516.434639) | 1275.965688(1185.304742 to 1356.690323) | 21127.31927(19827.16061 to 22431.63529) |
| Male | 2007 | 1198.525803(952.7294615 to 1489.382401) | 1223.233448(1127.047009 to 1300.970941) | 20184.09842(18793.22114 to 21450.07459) |
| Male | 2008 | 1175.023292(934.7989429 to 1458.984936) | 1190.78372(1096.046521 to 1271.673052) | 19686.34829(18294.71541 to 21035.75202) |
| Male | 2009 | 1153.778563(918.1982365 to 1433.021825) | 1156.17463(1063.628391 to 1232.209683) | 19144.46076(17752.88195 to 20366.11946) |
| Male | 2010 | 1137.49162(904.7786124 to 1409.91065) | 1131.551277(1037.052271 to 1211.16311) | 18773.99292(17337.10124 to 20080.01476) |
| Male | 2011 | 1121.905189(892.5560785 to 1389.974492) | 1076.622445(990.185494 to 1162.123499) | 17922.08003(16535.34334 to 19347.437) |
| Male | 2012 | 1105.143011(878.9032746 to 1366.942907) | 1041.428564(953.8525293 to 1129.712763) | 17377.39933(16011.51123 to 18890.50402) |
| Male | 2013 | 1090.328605(866.8970355 to 1345.722349) | 1006.703672(911.8442988 to 1096.742158) | 16775.12217(15338.88441 to 18266.07998) |
| Male | 2014 | 1081.317653(860.047034 to 1332.902022) | 975.9447831(884.8369519 to 1067.835586) | 16253.34394(14861.67454 to 17791.83586) |
| Male | 2015 | 1081.007839(859.9054246 to 1332.395721) | 955.8329005(856.9356037 to 1051.557749) | 15898.72108(14388.73834 to 17451.7473) |
| Male | 2016 | 1091.780788(868.1487964 to 1347.205477) | 942.173722(835.5674473 to 1050.522383) | 15665.4643(14074.26735 to 17448.62989) |
| Male | 2017 | 1108.66849(881.170781 to 1370.95323) | 916.4520294(808.3930089 to 1028.702019) | 15224.11826(13472.17527 to 17079.07381) |
| Male | 2018 | 1124.874729(894.6972832 to 1396.335116) | 898.5779307(788.1773825 to 1011.791574) | 14952.3664(13280.62468 to 16841.63562) |
| Male | 2019 | 1134.744172(902.039956 to 1413.478865) | 886.7016296(770.4843991 to 1013.409575) | 14750.3831(12899.35358 to 16794.88148) |
| Male | 2020 | 1094.246723(857.9161582 to 1376.825715) | 880.3092906(759.4721529 to 998.7977098) | 14613.40796(12718.00708 to 16590.84334) |
| Male | 2021 | 1099.714182(864.5783761 to 1388.0959) | 866.8771077(744.7811163 to 997.3472338) | 14416.39541(12523.64744 to 16526.13755) |
| Female | 1990 | 1142.634128(890.3443513 to 1445.084013) | 1232.558017(1108.300264 to 1319.630312) | 19430.01397(17712.93877 to 20792.6386) |
| Female | 1991 | 1130.611525(886.453397 to 1421.052548) | 1219.891513(1101.307525 to 1303.366725) | 19244.67457(17642.42302 to 20516.51186) |
| Female | 1992 | 1117.770342(881.5547783 to 1398.009564) | 1218.558503(1099.3904 to 1303.524527) | 19225.13809(17606.62927 to 20563.03145) |
| Female | 1993 | 1105.185395(876.7769697 to 1376.719689) | 1258.1485(1138.925234 to 1336.195108) | 19866.34188(18322.19848 to 21049.77414) |
| Female | 1994 | 1093.155644(871.3450383 to 1358.683787) | 1268.291765(1148.469879 to 1345.550911) | 20071.86424(18493.20917 to 21259.43788) |
| Female | 1995 | 1082.819642(865.1057248 to 1341.988113) | 1238.118762(1127.3295 to 1317.826991) | 19625.49031(18135.39918 to 20830.58781) |
| Female | 1996 | 1072.834754(858.1700367 to 1328.040342) | 1198.707882(1084.400692 to 1272.443337) | 19039.06107(17553.22336 to 20160.92846) |
| Female | 1997 | 1060.475023(848.9651525 to 1312.656301) | 1174.254859(1063.879838 to 1249.505174) | 18637.08976(17138.66151 to 19764.38216) |
| Female | 1998 | 1047.238581(837.708563 to 1296.482377) | 1158.149717(1044.659552 to 1230.831642) | 18337.85055(16858.94781 to 19449.50525) |
| Female | 1999 | 1033.898491(825.9129394 to 1280.272566) | 1166.584225(1061.525241 to 1239.040608) | 18458.9743(17045.18256 to 19521.33178) |
| Female | 2000 | 1020.160869(814.5290091 to 1263.507309) | 1159.635625(1051.545294 to 1231.396905) | 18365.47254(16939.83012 to 19393.6302) |
| Female | 2001 | 1005.556039(802.7792442 to 1245.419052) | 1146.308075(1036.383821 to 1218.76197) | 18184.75973(16714.70177 to 19273.88577) |
| Female | 2002 | 990.0137403(789.9512552 to 1225.732779) | 1136.431005(1030.034333 to 1202.157854) | 18016.6541(16625.04796 to 19005.12271) |
| Female | 2003 | 973.4097972(775.6694739 to 1205.512774) | 1129.139569(1018.992387 to 1199.909763) | 17879.79078(16410.89372 to 18913.36394) |
| Female | 2004 | 956.9571914(761.4582872 to 1186.430876) | 1085.113649(983.6157278 to 1151.322727) | 17228.83665(15894.64313 to 18228.96401) |
| Female | 2005 | 939.3138442(746.9239368 to 1165.161286) | 1053.590603(955.5076017 to 1117.57515) | 16742.80437(15462.38901 to 17700.40605) |
| Female | 2006 | 917.402684(730.5166282 to 1135.544245) | 968.8631437(875.5146815 to 1029.114109) | 15438.51804(14240.94667 to 16303.33042) |
| Female | 2007 | 891.2769987(710.3705104 to 1101.706559) | 916.0544319(824.5808192 to 977.1118426) | 14587.93046(13384.97573 to 15478.5145) |
| Female | 2008 | 864.1431713(688.9076194 to 1067.439957) | 887.103128(793.0188199 to 947.9527105) | 14100.94441(12855.10688 to 15011.22414) |
| Female | 2009 | 840.5155424(669.9081766 to 1037.814044) | 848.3286074(756.9143817 to 908.2207845) | 13503.6535(12314.06587 to 14393.49595) |
| Female | 2010 | 822.9302365(655.962836 to 1016.810458) | 821.4110832(732.8380253 to 881.6981089) | 13067.51784(11861.58088 to 13957.2678) |
| Female | 2011 | 808.7766178(645.0297994 to 999.6649173) | 774.2823174(686.4123537 to 835.7054532) | 12353.58197(11185.39675 to 13267.79923) |
| Female | 2012 | 794.9454903(633.7869488 to 982.5985388) | 734.642218(646.6844649 to 794.2226332) | 11757.73597(10567.11084 to 12647.64331) |
| Female | 2013 | 783.233399(624.5422212 to 968.2889944) | 704.9553544(619.5337344 to 764.3140977) | 11273.16324(10103.53967 to 12171.08968) |
| Female | 2014 | 776.1916167(618.1719937 to 961.4956087) | 689.4671312(604.6688495 to 749.3725268) | 10992.47547(9879.972752 to 11908.21427) |
| Female | 2015 | 775.4268359(616.9855627 to 960.3198498) | 672.5528512(583.7509542 to 733.2569145) | 10713.23584(9513.885533 to 11656.98988) |
| Female | 2016 | 786.4249044(625.2977323 to 975.1624141) | 660.7538379(574.079938 to 727.2300271) | 10523.74445(9357.145994 to 11504.11473) |
| Female | 2017 | 806.7674914(640.1104769 to 1000.86706) | 646.3152125(560.0242043 to 716.0574019) | 10283.59123(9115.478149 to 11299.56077) |
| Female | 2018 | 826.7581808(654.5073694 to 1026.50551) | 636.8773605(546.6479893 to 708.1894731) | 10144.74384(8875.473776 to 11272.7667) |
| Female | 2019 | 837.2588576(661.2302084 to 1042.367238) | 625.6752052(531.0487177 to 701.4411235) | 9976.243739(8622.615697 to 11149.16038) |
| Female | 2020 | 811.5471763(637.8793899 to 1024.34932) | 617.4879565(525.9094085 to 697.8256031) | 9851.723454(8599.390261 to 11083.01586) |
| Female | 2021 | 814.9429436(638.5617817 to 1031.429629) | 613.4071543(517.4416653 to 694.0713587) | 9859.717255(8514.842688 to 11086.46376) |
| Both | 1990 | 1243.588204(985.8520611 to 1544.319737) | 1332.340687(1212.986716 to 1409.368573) | 21570.87763(19903.05291 to 22817.12333) |
| Both | 1991 | 1237.330106(986.8951378 to 1528.509504) | 1319.955372(1208.829829 to 1396.896702) | 21387.76604(19870.43333 to 22629.93244) |
| Both | 1992 | 1229.803722(986.9335074 to 1511.611163) | 1320.84113(1208.2903 to 1396.142541) | 21432.6479(19823.88622 to 22636.58346) |
| Both | 1993 | 1221.8429(985.7306018 to 1495.617304) | 1356.719685(1245.462698 to 1427.670765) | 22053.73258(20584.03096 to 23146.37193) |
| Both | 1994 | 1213.478477(982.2108843 to 1480.968265) | 1363.818241(1256.197424 to 1432.571295) | 22237.28755(20810.56795 to 23310.1039) |
| Both | 1995 | 1205.923167(978.586184 to 1469.171846) | 1336.280317(1230.322939 to 1403.440078) | 21827.43411(20391.14492 to 22851.05218) |
| Both | 1996 | 1197.06358(972.3457032 to 1456.045421) | 1297.754968(1194.367104 to 1363.474403) | 21231.56449(19822.62949 to 22259.22313) |
| Both | 1997 | 1184.52698(962.3999709 to 1439.688608) | 1271.67491(1168.810278 to 1334.757836) | 20791.32963(19421.09733 to 21748.87074) |
| Both | 1998 | 1170.549216(950.845609 to 1422.229809) | 1256.623583(1151.15101 to 1323.038228) | 20496.32787(19114.93377 to 21525.92979) |
| Both | 1999 | 1156.436928(938.7341591 to 1405.141291) | 1264.862545(1165.31409 to 1332.164366) | 20634.97528(19302.15766 to 21633.22456) |
| Both | 2000 | 1142.829727(926.9729696 to 1388.791092) | 1267.627315(1171.151028 to 1331.669866) | 20709.14575(19423.63241 to 21688.31526) |
| Both | 2001 | 1129.329126(916.1200385 to 1373.800189) | 1259.109279(1158.554517 to 1324.981834) | 20578.32705(19208.53092 to 21578.51219) |
| Both | 2002 | 1115.696253(904.526734 to 1359.473444) | 1253.09023(1155.748885 to 1314.801095) | 20470.06918(19166.68829 to 21400.69378) |
| Both | 2003 | 1101.356341(892.2574935 to 1343.820391) | 1254.120992(1156.432507 to 1317.941679) | 20415.02865(19097.78484 to 21372.96831) |
| Both | 2004 | 1087.069369(880.1000791 to 1327.545861) | 1217.883375(1121.079959 to 1280.606244) | 19846.56498(18553.54426 to 20827.40126) |
| Both | 2005 | 1071.496319(866.947038 to 1308.87703) | 1185.114638(1093.785954 to 1245.310323) | 19313.23592(18092.28933 to 20263.83732) |
| Both | 2006 | 1051.403935(851.0993884 to 1282.729564) | 1090.587902(1000.772366 to 1145.521372) | 17817.50484(16664.54491 to 18657.03323) |
| Both | 2007 | 1027.128544(831.782345 to 1249.957996) | 1036.779717(948.8714403 to 1093.925904) | 16921.34029(15779.80752 to 17804.20628) |
| Both | 2008 | 1001.66924(811.7121014 to 1217.89118) | 1007.383263(917.5680559 to 1064.105971) | 16445.38077(15243.00822 to 17339.31918) |
| Both | 2009 | 979.3752199(792.9770898 to 1190.039465) | 970.7241622(887.4531335 to 1025.701972) | 15878.71662(14766.77626 to 16716.72223) |
| Both | 2010 | 962.5564829(778.8267194 to 1168.662974) | 945.56967(860.6351571 to 1003.201694) | 15481.07664(14302.06294 to 16358.40031) |
| Both | 2011 | 948.0388004(767.1347867 to 1150.973695) | 895.7598927(817.1053853 to 951.6939313) | 14716.00437(13626.00093 to 15556.74225) |
| Both | 2012 | 933.1047764(755.3601358 to 1132.764978) | 857.9764273(777.0131394 to 919.396194) | 14144.43446(13023.02741 to 15096.20364) |
| Both | 2013 | 920.2672532(745.5082318 to 1116.658516) | 826.4449627(746.3880824 to 885.5999899) | 13612.02803(12476.62724 to 14551.87383) |
| Both | 2014 | 912.6949263(739.4114267 to 1107.169319) | 805.6903292(725.3057445 to 866.8616401) | 13238.60157(12112.60202 to 14208.89593) |
| Both | 2015 | 912.3707547(739.6638432 to 1106.23959) | 787.7303774(707.3541854 to 849.9600434) | 12930.355(11799.3803 to 13905.24686) |
| Both | 2016 | 923.6013744(747.5459313 to 1120.476635) | 775.5890174(688.3557822 to 841.8353866) | 12727.47085(11520.96694 to 13780.0436) |
| Both | 2017 | 942.7393707(761.5043063 to 1146.991564) | 757.1080453(672.8130536 to 830.2504631) | 12407.09937(11182.03879 to 13515.73017) |
| Both | 2018 | 961.468348(775.7538074 to 1173.312619) | 744.8637545(657.8816526 to 815.7123455) | 12218.96616(10975.60496 to 13373.91066) |
| Both | 2019 | 972.0863398(783.9665751 to 1189.481475) | 733.5645697(644.4863455 to 806.4018391) | 12038.72078(10752.65894 to 13196.88963) |
| Both | 2020 | 940.218454(750.2366723 to 1160.832984) | 725.9441631(634.5414856 to 800.086359) | 11907.43586(10587.33585 to 13059.36543) |
| Both | 2021 | 944.8725063(750.112078 to 1171.708225) | 718.0111186(625.1379875 to 797.2417611) | 11828.11742(10514.83263 to 13067.77705) |
| Male | 1990 | 1013.847993(806.7220792 to 1257.908997) | 1046.298564(903.1952606 to 1181.817547) | 19471.43203(17050.33949 to 21808.69958) |
| Male | 1991 | 1009.982589(806.7193442 to 1247.720496) | 1041.827662(899.3417715 to 1171.168197) | 19380.90386(16944.0672 to 21659.13201) |
| Male | 1992 | 1005.603738(805.7464002 to 1238.21614) | 1035.654227(895.9652087 to 1164.500524) | 19267.04847(16892.94147 to 21521.5375) |
| Male | 1993 | 1000.996965(804.1969778 to 1228.667162) | 1034.781689(900.4643901 to 1167.149906) | 19266.22635(16987.35285 to 21543.54673) |
| Male | 1994 | 996.3008097(801.354698 to 1219.994417) | 1043.374618(912.8876713 to 1172.715413) | 19376.86092(17187.10083 to 21586.86597) |
| Male | 1995 | 991.5791993(798.3189831 to 1211.70607) | 1041.007872(908.2845045 to 1165.342378) | 19326.08073(17060.87598 to 21451.00835) |
| Male | 1996 | 985.8568278(793.4408678 to 1203.992662) | 1027.271662(899.2863794 to 1151.699302) | 19119.68551(16911.58752 to 21263.10965) |
| Male | 1997 | 978.3597986(786.4732782 to 1195.425968) | 1019.110861(895.1860896 to 1141.154743) | 19053.53569(16905.54588 to 21156.4991) |
| Male | 1998 | 970.0675652(778.8329584 to 1185.49422) | 1017.580248(894.8878653 to 1137.179662) | 19022.34547(16907.09259 to 21072.11148) |
| Male | 1999 | 961.592169(770.912062 to 1175.902549) | 992.3975451(879.2716659 to 1110.104779) | 18463.22231(16548.64006 to 20458.23471) |
| Male | 2000 | 953.1967046(763.2855176 to 1166.472312) | 980.9383135(869.1813453 to 1094.866092) | 18186.73142(16270.61121 to 20111.1891) |
| Male | 2001 | 944.2541124(756.3651377 to 1155.117958) | 962.7168175(853.5453307 to 1074.651775) | 17895.33128(16007.02237 to 19817.93857) |
| Male | 2002 | 934.1732587(748.3938891 to 1142.956511) | 944.7215133(838.9565663 to 1053.15808) | 17541.12165(15715.53909 to 19348.52354) |
| Male | 2003 | 923.9389373(740.0180151 to 1130.783452) | 939.6327949(836.3263363 to 1046.204953) | 17372.09644(15571.85913 to 19180.15877) |
| Male | 2004 | 913.7794562(732.3282768 to 1118.493857) | 922.5875062(822.3282383 to 1028.932641) | 17056.19938(15314.69422 to 18847.51358) |
| Male | 2005 | 903.1943295(724.1900014 to 1105.681278) | 898.3279288(803.9355294 to 996.4576863) | 16619.3716(15011.86981 to 18282.21493) |
| Male | 2006 | 890.4394421(714.8776462 to 1090.170641) | 889.0325938(794.849069 to 991.2458324) | 16376.58149(14773.15114 to 18140.88241) |
| Male | 2007 | 874.9660973(703.2661884 to 1071.290343) | 880.1262352(786.8460519 to 977.9213168) | 16190.18305(14596.05596 to 17865.61947) |
| Male | 2008 | 858.7351239(691.1051184 to 1050.536561) | 870.8806849(778.1017241 to 969.2304861) | 16007.29829(14407.56922 to 17704.83873) |
| Male | 2009 | 843.5787193(680.0471135 to 1029.890532) | 861.2264348(767.3436254 to 958.2238773) | 15866.82173(14234.3575 to 17532.25716) |
| Male | 2010 | 831.484797(670.4968466 to 1013.99007) | 857.4405209(765.7239629 to 955.4410864) | 15807.37065(14212.26099 to 17470.75803) |
| Male | 2011 | 820.9150879(662.2531016 to 1000.471432) | 849.9466567(757.7106606 to 951.6003655) | 15634.01796(14077.25572 to 17334.96458) |
| Male | 2012 | 810.1021906(653.8842847 to 985.8316827) | 835.3874127(743.1657182 to 933.8526639) | 15328.30421(13796.97776 to 17004.88194) |
| Male | 2013 | 801.2258651(647.3990113 to 973.694428) | 850.8504248(760.3962449 to 953.0069827) | 15404.28793(13879.10883 to 17106.159) |
| Male | 2014 | 796.2194984(644.2346122 to 967.4732723) | 860.2016056(766.0559696 to 964.734877) | 15400.65393(13834.16102 to 17118.63063) |
| Male | 2015 | 795.4022603(644.0822928 to 966.061271) | 857.4267968(759.5629284 to 969.67298) | 15331.55626(13662.63822 to 17199.8944) |
| Male | 2016 | 798.5808279(646.354406 to 969.2217058) | 849.4607121(753.5161913 to 955.0359133) | 15163.9466(13558.2355 to 16926.53176) |
| Male | 2017 | 803.4084742(649.8204719 to 975.1744234) | 841.4062037(744.3470131 to 942.6041285) | 15039.75636(13429.21361 to 16739.18483) |
| Male | 2018 | 807.3592038(652.5997676 to 980.6779827) | 822.578513(724.409309 to 926.2229999) | 14820.33931(13139.03936 to 16590.99553) |
| Male | 2019 | 808.7844341(653.3744607 to 983.0623) | 818.0603066(720.491846 to 921.633169) | 14744.67315(13027.76541 to 16486.53756) |
| Male | 2020 | 794.203003(637.8210017 to 971.7360317) | 814.0842146(713.4491472 to 923.7228168) | 14680.75145(12968.07661 to 16521.1607) |
| Male | 2021 | 792.3613258(635.5895178 to 967.4006571) | 794.1426238(694.2481434 to 901.8811517) | 14404.32959(12668.56233 to 16239.44587) |
| Female | 1990 | 954.746741(759.4689908 to 1184.079571) | 962.9061325(807.8861709 to 1129.68076) | 17517.82843(14845.37066 to 20342.54466) |
| Female | 1991 | 947.2093892(756.1852549 to 1171.822524) | 958.3019591(807.5938662 to 1119.165445) | 17431.90989(14885.5923 to 20215.2919) |
| Female | 1992 | 939.6353741(753.8605001 to 1159.328992) | 957.0305895(806.2074275 to 1119.332063) | 17387.32653(14817.35406 to 20157.53906) |
| Female | 1993 | 932.3795298(751.5690116 to 1147.788625) | 953.5338466(804.892858 to 1115.250602) | 17303.48786(14802.09664 to 20078.13646) |
| Female | 1994 | 925.3813978(748.553514 to 1136.89429) | 961.9640977(809.3139793 to 1126.295535) | 17387.26523(14830.406 to 20200.32389) |
| Female | 1995 | 919.2916652(745.3434135 to 1126.987932) | 965.4351404(816.5690814 to 1130.289675) | 17391.37196(14863.35517 to 20164.74889) |
| Female | 1996 | 913.5892789(740.9172314 to 1119.228345) | 948.3132952(802.7989186 to 1110.322596) | 17142.11375(14663.75142 to 19885.40022) |
| Female | 1997 | 906.7317254(734.8539864 to 1110.61954) | 931.3209543(789.0457513 to 1085.486647) | 16861.20992(14453.68649 to 19514.93818) |
| Female | 1998 | 898.6718909(727.3930041 to 1100.571024) | 917.4936953(774.664831 to 1069.175326) | 16678.31174(14271.10142 to 19273.29013) |
| Female | 1999 | 889.6041573(718.986551 to 1089.835856) | 897.2620702(759.2952462 to 1040.718818) | 16348.61434(14007.7957 to 18812.06759) |
| Female | 2000 | 880.141619(709.7490681 to 1078.207616) | 883.4912773(748.8960897 to 1025.615667) | 16091.30769(13830.30202 to 18481.97713) |
| Female | 2001 | 869.8407951(701.6660035 to 1066.121279) | 872.1511798(740.0546324 to 1012.21927) | 15878.48667(13623.44143 to 18254.94899) |
| Female | 2002 | 858.4577523(692.7056372 to 1052.602445) | 874.6878887(743.9781636 to 1011.976891) | 15900.66324(13673.72006 to 18238.40586) |
| Female | 2003 | 846.6825986(683.7115933 to 1038.305265) | 865.220614(736.1812868 to 1001.124982) | 15740.9465(13546.79653 to 18024.96312) |
| Female | 2004 | 835.184356(674.8690052 to 1024.501545) | 843.2862502(718.3811223 to 967.8552296) | 15271.91163(13143.42312 to 17404.35372) |
| Female | 2005 | 824.705528(666.4231786 to 1011.850861) | 827.7881254(706.5834627 to 951.8740307) | 14942.25193(12929.0195 to 17032.9884) |
| Female | 2006 | 814.9524794(659.7670391 to 1000.562692) | 823.9371188(702.6975338 to 948.7283039) | 14831.29824(12810.92487 to 16913.38656) |
| Female | 2007 | 805.0781793(652.5363701 to 988.0771126) | 813.3355982(694.4149473 to 933.8042183) | 14623.52131(12633.73827 to 16641.2989) |
| Female | 2008 | 795.4797721(646.1485654 to 973.8193575) | 807.2592733(688.6828829 to 926.900854) | 14485.576(12539.92844 to 16479.60006) |
| Female | 2009 | 786.5217007(639.3008942 to 961.951678) | 798.2305892(681.0721968 to 918.8916888) | 14319.58178(12362.59417 to 16325.97826) |
| Female | 2010 | 778.7532655(632.9145518 to 951.2114984) | 785.3340527(672.4463198 to 902.1817937) | 14093.03122(12235.88444 to 16025.09792) |
| Female | 2011 | 770.8343194(626.6783264 to 939.6998786) | 781.1133784(669.0194078 to 899.2492887) | 14037.45949(12172.46343 to 16002.87172) |
| Female | 2012 | 763.195523(620.7960797 to 928.3282876) | 788.0898428(674.2679781 to 904.5050344) | 14131.61559(12243.15941 to 16071.41303) |
| Female | 2013 | 757.7207059(616.0315774 to 919.9137555) | 802.0829773(685.0119709 to 920.2973142) | 14149.45917(12232.832 to 16098.86128) |
| Female | 2014 | 755.0328003(613.3599101 to 915.0221973) | 804.1878037(686.7795196 to 918.8186965) | 14108.54252(12197.50062 to 15988.45772) |
| Female | 2015 | 754.8351544(612.9723884 to 913.8356501) | 791.9788215(677.8020388 to 909.0032088) | 13946.0636(12083.52598 to 15853.10875) |
| Female | 2016 | 757.8164158(616.7564873 to 917.2719531) | 778.8484535(665.7137664 to 891.4128198) | 13737.37524(11887.72828 to 15585.64864) |
| Female | 2017 | 762.8689585(620.7404573 to 924.0335229) | 772.7516454(660.4312474 to 884.3273594) | 13639.51353(11793.3262 to 15473.12351) |
| Female | 2018 | 767.4313209(624.4438471 to 929.7694235) | 762.1224436(652.11832 to 871.9383366) | 13490.9585(11683.03681 to 15321.93464) |
| Female | 2019 | 769.2500703(625.4848199 to 933.4592169) | 750.6338685(642.0801301 to 861.8839293) | 13299.30552(11501.70675 to 15112.04702) |
| Female | 2020 | 757.9679764(613.7072544 to 929.5374957) | 737.1122134(629.4647548 to 850.0306251) | 13066.53065(11281.86238 to 14918.0319) |
| Female | 2021 | 760.8924427(611.4600713 to 933.9664362) | 724.3200562(612.8400923 to 834.7144628) | 12859.46124(11023.26099 to 14649.94197) |
| Both | 1990 | 985.5121996(801.2162594 to 1201.158334) | 1005.771996(892.7825545 to 1125.868282) | 18527.67316(16610.67917 to 20594.33521) |
| Both | 1991 | 979.8408963(799.6217581 to 1189.060751) | 1001.253344(888.1165757 to 1117.634074) | 18440.31499(16525.31705 to 20440.97485) |
| Both | 1992 | 973.8781978(797.5736443 to 1177.68536) | 997.7448933(887.5072704 to 1115.892954) | 18362.8762(16484.15592 to 20352.47731) |
| Both | 1993 | 967.9424419(795.4588908 to 1167.725187) | 995.6712932(885.8877945 to 1111.74137) | 18322.29794(16437.90676 to 20313.35532) |
| Both | 1994 | 962.0573771(792.7403973 to 1157.423491) | 1004.144522(895.3081785 to 1124.27032) | 18418.98006(16571.18774 to 20475.47365) |
| Both | 1995 | 956.6065442(789.194164 to 1147.636054) | 1004.919344(893.6413986 to 1123.498088) | 18396.12843(16507.95151 to 20430.0147) |
| Both | 1996 | 950.8190661(784.527855 to 1139.521391) | 989.3712881(881.7642866 to 1103.361172) | 18164.95106(16299.74351 to 20130.54692) |
| Both | 1997 | 943.5309068(778.3270307 to 1130.396982) | 976.6803284(871.4534623 to 1086.470838) | 17989.70532(16201.09117 to 19875.59734) |
| Both | 1998 | 935.1969368(770.9712348 to 1120.129155) | 968.4880988(863.4291104 to 1080.263543) | 17874.80059(16069.7167 to 19812.8453) |
| Both | 1999 | 926.2413668(763.1534971 to 1109.667726) | 945.2089604(847.3953996 to 1052.441193) | 17420.57058(15735.24636 to 19242.19313) |
| Both | 2000 | 917.0969039(755.1187764 to 1099.573309) | 932.2039747(834.684601 to 1037.065779) | 17147.50391(15460.22547 to 18935.52586) |
| Both | 2001 | 907.2746985(746.8623304 to 1087.948572) | 917.473367(825.0579452 to 1018.779266) | 16893.54058(15322.25998 to 18612.1603) |
| Both | 2002 | 896.3835568(737.7803931 to 1074.920906) | 909.7921406(814.7916124 to 1009.502351) | 16725.29032(15116.23522 to 18387.60851) |
| Both | 2003 | 885.252827(728.4317353 to 1061.711472) | 902.1139462(809.1604851 to 997.4508434) | 16556.14785(14984.14803 to 18166.47783) |
| Both | 2004 | 874.2950535(719.4057616 to 1049.917491) | 882.7120833(792.5532551 to 976.8840854) | 16163.60026(14642.03108 to 17749.18956) |
| Both | 2005 | 863.6893003(710.6263158 to 1038.034101) | 863.0191467(778.2643072 to 954.5892023) | 15781.34288(14374.90402 to 17334.95256) |
| Both | 2006 | 852.4223508(702.7103477 to 1022.188754) | 856.3093282(772.0025249 to 948.5364074) | 15602.78082(14188.84313 to 17161.93236) |
| Both | 2007 | 839.7794915(693.7270275 to 1005.122875) | 846.4601552(762.2223504 to 936.2532461) | 15404.88364(13999.1688 to 16905.1632) |
| Both | 2008 | 826.9162865(683.759105 to 987.938917) | 838.8296235(753.8998074 to 927.7626116) | 15245.11329(13843.8521 to 16769.79317) |
| Both | 2009 | 814.9115281(674.3451658 to 972.1966628) | 829.638379(745.4838661 to 919.5009983) | 15093.6113(13689.76496 to 16609.73756) |
| Both | 2010 | 804.9911652(666.2238368 to 959.9611404) | 821.1699935(735.7391578 to 908.4629994) | 14948.83868(13565.92455 to 16428.94963) |
| Both | 2011 | 795.760211(659.4917625 to 947.7138266) | 815.1068128(728.4165559 to 904.1583223) | 14832.62208(13399.76752 to 16347.10886) |
| Both | 2012 | 786.5634689(652.4393233 to 935.9199694) | 811.6284407(726.9704405 to 901.7953669) | 14730.43233(13348.28752 to 16224.29275) |
| Both | 2013 | 779.4529296(647.1812369 to 926.6503598) | 826.540574(740.4449975 to 914.7341032) | 14778.16386(13380.47318 to 16263.84427) |
| Both | 2014 | 775.6338349(644.6743771 to 922.0595963) | 831.6776365(745.3004465 to 920.4351676) | 14750.71599(13344.00574 to 16255.46465) |
| Both | 2015 | 775.0798114(644.127664 to 921.1142665) | 823.7002282(734.8118068 to 917.9872158) | 14630.04502(13182.82583 to 16203.34218) |
| Both | 2016 | 778.0867121(646.2563652 to 923.5471162) | 812.8210356(726.3618393 to 903.7091574) | 14438.02905(13018.49068 to 15929.2794) |
| Both | 2017 | 782.9683528(649.7514103 to 928.6819448) | 805.7654979(717.9641669 to 895.6813053) | 14326.69458(12875.45412 to 15823.51424) |
| Both | 2018 | 787.1871119(652.7471633 to 933.2315278) | 791.398087(701.5740551 to 882.1088162) | 14145.62024(12625.2977 to 15664.88083) |
| Both | 2019 | 788.7756705(653.8415524 to 935.6341886) | 783.1480439(695.0466037 to 871.6694759) | 14008.51114(12527.56617 to 15507.56616) |
| Both | 2020 | 775.8708842(639.1860921 to 929.6502452) | 774.0516595(682.6125026 to 867.8521631) | 13854.87953(12312.94718 to 15420.02974) |
| Both | 2021 | 776.4100365(638.2823118 to 931.6186008) | 757.9584188(666.9315783 to 850.3752105) | 13613.90078(12058.95306 to 15182.95996) |
| Male | 1990 | 938.5013923(742.2344588 to 1167.071) | 920.1731284(804.9281838 to 1023.769935) | 17010.7296(15047.84235 to 18799.86199) |
| Male | 1991 | 937.5899107(743.5551503 to 1163.031776) | 911.6993516(803.8008336 to 1012.326988) | 16853.68916(15028.72484 to 18568.53557) |
| Male | 1992 | 936.533996(743.7701043 to 1158.63004) | 911.9835412(806.5094217 to 1010.418315) | 16870.01567(15081.77499 to 18534.18352) |
| Male | 1993 | 935.2189349(743.2601654 to 1154.696615) | 912.3662942(807.6519038 to 1009.420266) | 16865.19632(15088.69337 to 18534.9337) |
| Male | 1994 | 933.6196314(741.8717569 to 1152.065308) | 914.5620852(815.6730554 to 1008.187442) | 16911.08709(15223.40645 to 18522.67809) |
| Male | 1995 | 931.3366587(739.8211918 to 1149.930436) | 918.1591729(820.9535637 to 1007.826188) | 16961.20291(15326.02276 to 18506.92202) |
| Male | 1996 | 928.4336778(737.9697095 to 1145.40814) | 913.3489655(818.7726042 to 1002.515263) | 16858.38659(15276.33664 to 18406.88266) |
| Male | 1997 | 925.1040771(735.8393774 to 1140.536071) | 912.466012(821.314536 to 1001.155751) | 16809.84624(15267.9678 to 18345.91834) |
| Male | 1998 | 921.3421156(733.2137485 to 1135.652716) | 909.8629121(821.920616 to 993.05556) | 16733.76793(15259.80657 to 18155.1172) |
| Male | 1999 | 916.8050849(729.9381397 to 1130.003433) | 884.4322245(800.8704411 to 968.7545156) | 16251.6424(14866.07363 to 17663.23478) |
| Male | 2000 | 911.4282421(725.2157384 to 1122.915845) | 875.0538764(791.5333926 to 955.3121184) | 16099.0057(14719.09725 to 17439.93411) |
| Male | 2001 | 905.3176894(719.7046802 to 1114.790629) | 878.5170487(800.6580652 to 954.7684806) | 16132.17618(14819.28998 to 17423.93416) |
| Male | 2002 | 898.2239804(712.9519701 to 1105.658566) | 876.2856469(798.3724019 to 949.9304218) | 16076.78744(14774.72554 to 17342.65909) |
| Male | 2003 | 890.753692(706.2587463 to 1096.869363) | 882.4511742(807.1524769 to 954.5706319) | 16142.85717(14875.26695 to 17387.48175) |
| Male | 2004 | 883.3656546(699.658828 to 1088.435082) | 875.6205704(798.1936306 to 945.3687796) | 15959.57294(14672.67503 to 17161.51081) |
| Male | 2005 | 875.8890559(692.9513696 to 1078.882123) | 859.1339075(786.2617266 to 927.8245024) | 15664.4352(14459.99465 to 16843.4529) |
| Male | 2006 | 866.8933727(687.191935 to 1066.952057) | 851.5090759(781.8525707 to 919.3433181) | 15510.20576(14340.57086 to 16655.8971) |
| Male | 2007 | 855.578255(679.5255206 to 1052.005891) | 857.7602293(787.1704134 to 928.3830131) | 15617.42666(14428.31779 to 16812.84084) |
| Male | 2008 | 843.416252(671.2259859 to 1034.959607) | 857.3620562(784.604037 to 925.8298123) | 15621.35098(14395.79003 to 16801.36976) |
| Male | 2009 | 831.6768121(663.425204 to 1018.443017) | 840.8434445(771.5862192 to 907.7081869) | 15318.35263(14147.45237 to 16445.42074) |
| Male | 2010 | 822.1290094(658.2853315 to 1006.668438) | 834.9790202(765.6858145 to 902.6480884) | 15188.11426(14029.63578 to 16337.17685) |
| Male | 2011 | 813.255064(652.0410582 to 995.9354551) | 814.0574455(743.1199596 to 882.5752851) | 14899.98423(13722.71443 to 16052.75966) |
| Male | 2012 | 803.4599872(645.1205194 to 982.8285736) | 785.6312993(717.1823782 to 852.4880827) | 14429.4612(13315.04299 to 15573.33685) |
| Male | 2013 | 794.9654965(638.2999152 to 970.950818) | 798.4623069(733.8034999 to 865.7027518) | 14380.69015(13298.66508 to 15543.16776) |
| Male | 2014 | 789.9630874(634.5949173 to 964.4158711) | 814.002128(743.5864371 to 887.9081013) | 14405.42148(13273.30943 to 15651.24962) |
| Male | 2015 | 789.6433496(634.7756149 to 963.6651065) | 790.700218(722.7281334 to 861.32505) | 14162.22847(13031.9354 to 15354.04227) |
| Male | 2016 | 794.1209259(638.9958377 to 968.811771) | 777.2582228(708.5652712 to 850.1068159) | 14018.81785(12878.50442 to 15242.65334) |
| Male | 2017 | 800.9462908(644.8699765 to 977.2638127) | 781.0812571(710.1174391 to 854.8088731) | 14094.54841(12937.10097 to 15345.79224) |
| Male | 2018 | 807.9389832(650.4333589 to 986.4423906) | 784.4048925(709.7573531 to 858.5895827) | 14191.61114(12940.73676 to 15446.67698) |
| Male | 2019 | 812.3876419(653.7409026 to 993.84869) | 781.2930675(705.556665 to 859.4404381) | 14139.85449(12898.79254 to 15479.16064) |
| Male | 2020 | 793.4419801(632.5104462 to 975.755697) | 778.0976011(701.1933909 to 856.9328985) | 14069.28223(12790.18242 to 15428.32424) |
| Male | 2021 | 795.4546099(634.2815143 to 978.1332001) | 773.0747046(694.6614152 to 853.1127687) | 13955.7226(12621.73067 to 15347.74835) |
| Female | 1990 | 872.1942561(692.0202485 to 1088.566412) | 913.9120744(797.4032033 to 1031.78261) | 16205.49115(14281.33081 to 18079.9728) |
| Female | 1991 | 870.2951326(694.0094853 to 1082.021254) | 898.7903186(783.6407951 to 1010.898555) | 15992.06831(14076.68031 to 17819.06713) |
| Female | 1992 | 868.2320119(694.9109777 to 1076.764926) | 902.2408929(789.4988379 to 1011.315113) | 16046.3673(14191.75166 to 17835.57762) |
| Female | 1993 | 865.8894314(694.7378615 to 1071.330797) | 895.2526134(782.9755256 to 1001.823523) | 15928.65202(14091.76012 to 17654.82703) |
| Female | 1994 | 863.0878071(693.963104 to 1063.231156) | 892.669825(783.6573573 to 997.7343885) | 15894.81326(14107.86809 to 17607.10609) |
| Female | 1995 | 859.8529026(692.808545 to 1057.447148) | 902.0863484(795.3373584 to 1008.945348) | 15974.88622(14236.6575 to 17707.00067) |
| Female | 1996 | 855.4788228(690.047002 to 1051.319247) | 892.1805154(786.4290357 to 995.7164509) | 15817.2285(14104.20474 to 17483.45418) |
| Female | 1997 | 849.3699929(684.9311482 to 1042.713355) | 879.0983531(770.015455 to 979.4646676) | 15660.46281(13904.64345 to 17282.39919) |
| Female | 1998 | 842.0918034(678.6691736 to 1032.754662) | 875.8484804(769.5491725 to 971.1036315) | 15622.59585(13888.9166 to 17183.04671) |
| Female | 1999 | 834.0231302(671.2987383 to 1022.39918) | 859.7505395(757.2459448 to 951.3833062) | 15278.45914(13622.76058 to 16761.10781) |
| Female | 2000 | 825.4194898(663.4897778 to 1011.400275) | 839.533685(743.2906896 to 924.5229487) | 14906.44334(13368.84818 to 16309.51509) |
| Female | 2001 | 816.0723502(656.0026817 to 1000.663354) | 832.6294758(736.6436886 to 913.9505307) | 14802.95693(13261.91871 to 16146.8414) |
| Female | 2002 | 805.4486336(647.4054388 to 988.4083586) | 834.0591574(743.5340636 to 917.7464318) | 14817.05814(13374.76573 to 16200.20766) |
| Female | 2003 | 794.4428018(638.3129115 to 975.96772) | 827.0546831(734.3057872 to 908.1316885) | 14671.39501(13199.78948 to 15991.28003) |
| Female | 2004 | 784.1301896(629.5666119 to 964.0125277) | 804.481091(715.5221721 to 887.3031039) | 14260.65685(12846.54589 to 15610.01605) |
| Female | 2005 | 774.8100132(622.4851115 to 953.3376679) | 795.7279143(704.468853 to 876.4112595) | 14125.58298(12686.9722 to 15463.27214) |
| Female | 2006 | 764.8614711(615.1834331 to 939.5002547) | 799.2994637(710.6976357 to 879.5990276) | 14159.34779(12780.34754 to 15473.02148) |
| Female | 2007 | 753.2807646(607.3312568 to 924.3342957) | 799.1648775(711.9436135 to 878.0124752) | 14103.77929(12716.05928 to 15398.45092) |
| Female | 2008 | 741.1099(598.9854499 to 908.6899972) | 790.0391089(704.8478185 to 866.81198) | 13910.42266(12598.39007 to 15165.17258) |
| Female | 2009 | 729.4483426(590.4863275 to 894.0394527) | 770.8321068(687.983549 to 845.0232268) | 13588.73808(12307.35381 to 14803.26086) |
| Female | 2010 | 719.5081064(582.6401338 to 880.8757997) | 756.7085802(674.9735795 to 829.8734535) | 13366.13968(12073.78748 to 14591.56111) |
| Female | 2011 | 709.7716114(574.9810503 to 868.7010895) | 741.849757(661.0396165 to 815.5886771) | 13163.41692(11909.7049 to 14363.11189) |
| Female | 2012 | 699.7674299(566.7525668 to 855.6461452) | 731.7606129(650.3861454 to 806.9107536) | 13037.73695(11762.15015 to 14273.60941) |
| Female | 2013 | 691.1673785(560.58619 to 843.9421398) | 730.3460728(651.4284143 to 805.3194153) | 12843.68319(11616.68462 to 14090.23161) |
| Female | 2014 | 685.6883045(556.6591764 to 836.6226155) | 726.4675466(647.5009999 to 797.2146907) | 12597.38052(11399.73856 to 13735.75372) |
| Female | 2015 | 684.4025786(555.589973 to 834.053314) | 717.2732065(635.7503995 to 791.1560663) | 12509.88755(11242.2706 to 13719.44689) |
| Female | 2016 | 687.4776417(558.3676797 to 837.2106626) | 710.0854869(630.1570483 to 783.4836618) | 12428.2476(11163.41767 to 13615.6008) |
| Female | 2017 | 693.0325092(562.7026111 to 843.7889617) | 709.3273052(629.2225981 to 782.8541148) | 12400.03424(11147.95084 to 13595.43235) |
| Female | 2018 | 698.8782824(566.4095714 to 851.4107565) | 704.0681219(625.8563145 to 777.1437519) | 12352.51409(11127.86474 to 13551.63339) |
| Female | 2019 | 703.0359386(568.9929625 to 857.4668152) | 694.984554(617.7691098 to 770.0851816) | 12196.60781(10983.08882 to 13470.4835) |
| Female | 2020 | 691.5633233(557.2766352 to 850.9574608) | 682.3159122(602.1430271 to 758.869679) | 11974.12002(10716.98379 to 13196.00664) |
| Female | 2021 | 694.0695115(559.0759637 to 854.2428955) | 668.9389781(587.2323955 to 747.3478993) | 11746.59473(10435.68193 to 13031.05693) |
| Both | 1990 | 906.5143347(734.009577 to 1113.554885) | 918.4870293(827.9653364 to 1006.312846) | 16630.2177(15112.92222 to 18120.12733) |
| Both | 1991 | 905.0922184(735.5190862 to 1107.839985) | 906.4860964(819.9880755 to 989.8716892) | 16442.84818(15022.01707 to 17820.72756) |
| Both | 1992 | 903.4957553(735.7865164 to 1101.949186) | 908.4127535(822.1308538 to 988.2953017) | 16478.5458(15064.07604 to 17823.14054) |
| Both | 1993 | 901.5926922(735.3262072 to 1095.733132) | 904.9861768(820.391941 to 982.901746) | 16415.72912(15015.25694 to 17716.59563) |
| Both | 1994 | 899.294667(735.1693206 to 1089.604829) | 904.6977286(824.1510497 to 980.5986289) | 16420.03578(15113.01486 to 17691.62345) |
| Both | 1995 | 896.3878887(734.3972199 to 1082.882989) | 911.3496285(831.3860785 to 987.8280405) | 16485.05569(15156.30459 to 17768.23837) |
| Both | 1996 | 892.5698996(731.7830943 to 1077.265119) | 903.7234002(825.2062491 to 976.9533974) | 16350.73705(15064.72183 to 17561.90455) |
| Both | 1997 | 887.66684(727.5617276 to 1070.9414) | 896.3019243(818.8203663 to 967.3623753) | 16241.96275(14974.41677 to 17433.89444) |
| Both | 1998 | 881.9579606(722.3455084 to 1064.133627) | 893.1832397(816.9399692 to 963.7591908) | 16181.18577(14930.30395 to 17361.89492) |
| Both | 1999 | 875.4612282(716.5624383 to 1056.436187) | 872.5195965(799.5219228 to 939.9000615) | 15767.3936(14595.24501 to 16891.44065) |
| Both | 2000 | 868.2744568(710.3819223 to 1048.552793) | 857.5459569(787.9166027 to 921.53569) | 15501.14685(14400.25271 to 16565.39018) |
| Both | 2001 | 860.3627599(703.9446363 to 1039.301209) | 855.4870118(784.0647413 to 917.3249478) | 15460.74483(14322.78552 to 16502.18843) |
| Both | 2002 | 851.3307255(696.1005096 to 1028.721535) | 855.0425249(786.5882229 to 915.2958605) | 15438.7938(14370.38717 to 16438.2589) |
| Both | 2003 | 841.9412778(687.9739966 to 1017.888182) | 854.2268186(785.49564 to 912.8812686) | 15393.57263(14298.94222 to 16382.80111) |
| Both | 2004 | 832.9606753(680.2092472 to 1007.606677) | 838.9983861(771.18624 to 898.7047839) | 15089.80076(14033.10432 to 16077.61767) |
| Both | 2005 | 824.4784469(673.4171516 to 997.6309944) | 826.5082934(760.524698 to 884.9100216) | 14876.3463(13822.78052 to 15868.18129) |
| Both | 2006 | 814.9467246(666.4808741 to 984.5084762) | 824.7966099(758.957345 to 883.5363487) | 14820.39405(13790.77484 to 15798.69386) |
| Both | 2007 | 803.4310342(658.2356528 to 967.4474586) | 827.7790156(762.118638 to 886.5284222) | 14843.7427(13819.70782 to 15795.48661) |
| Both | 2008 | 791.1990075(649.0889904 to 951.2942765) | 822.8224105(758.3729374 to 882.2578831) | 14745.34914(13718.88749 to 15729.19288) |
| Both | 2009 | 779.4019045(639.7719252 to 937.066877) | 804.7383231(738.7023089 to 862.5030831) | 14430.54758(13381.68001 to 15408.78339) |
| Both | 2010 | 769.553105(631.5797038 to 924.6106574) | 794.2723964(729.9991084 to 853.9747415) | 14248.94035(13220.0399 to 15198.39056) |
| Both | 2011 | 760.125591(624.547389 to 912.1160784) | 776.5612581(712.2768692 to 834.2518008) | 14004.83004(12965.47494 to 14975.8772) |
| Both | 2012 | 750.1246579(617.577318 to 899.5068986) | 757.7693467(694.3619074 to 814.9419073) | 13712.74235(12704.93452 to 14678.94186) |
| Both | 2013 | 741.4970365(611.4102591 to 889.3041986) | 762.6683397(701.58507 to 821.0244259) | 13582.02706(12612.58977 to 14530.87694) |
| Both | 2014 | 736.1530747(607.6803252 to 881.9021436) | 767.4110286(703.5420417 to 825.2494485) | 13459.92786(12480.6004 to 14379.421) |
| Both | 2015 | 735.2332624(607.414611 to 879.1390027) | 751.8709255(686.8845668 to 813.5349268) | 13302.18244(12299.07769 to 14309.56593) |
| Both | 2016 | 738.8934123(610.2500028 to 883.4685507) | 741.9685539(677.6917192 to 801.2338795) | 13193.05542(12181.5414 to 14179.68413) |
| Both | 2017 | 744.9837356(615.1137251 to 890.7292762) | 743.4811654(678.7734756 to 804.0944879) | 13215.06722(12198.5961 to 14206.70817) |
| Both | 2018 | 751.29706(619.744035 to 898.0339042) | 742.157637(676.2873032 to 802.2133583) | 13234.74544(12174.29185 to 14226.46988) |
| Both | 2019 | 755.5138306(622.8595351 to 903.3704187) | 735.80932(670.0004427 to 797.1564292) | 13126.70723(12062.13147 to 14154.3486) |
| Both | 2020 | 740.3891485(605.9511838 to 892.6340405) | 727.3659679(660.5665353 to 790.6565228) | 12972.30717(11894.90362 to 14038.37377) |
| Both | 2021 | 742.4416509(606.9949096 to 895.0788057) | 717.4753324(646.3956371 to 782.3150371) | 12791.85576(11678.10951 to 13882.88659) |
| Male | 1990 | 1159.641792(911.1333822 to 1458.465243) | 1285.753841(1134.829928 to 1435.229261) | 22459.5453(19936.41976 to 25139.35672) |
| Male | 1991 | 1174.403405(927.3450242 to 1471.577305) | 1275.196898(1128.166125 to 1412.232279) | 22252.66(19776.22927 to 24678.42858) |
| Male | 1992 | 1186.452831(939.1786517 to 1483.638455) | 1275.911502(1137.744359 to 1419.82264) | 22220.9427(19898.03391 to 24722.89817) |
| Male | 1993 | 1195.434189(947.9472139 to 1493.521212) | 1276.424756(1151.659781 to 1401.847322) | 22181.1304(20132.57855 to 24383.62191) |
| Male | 1994 | 1201.041926(953.3029728 to 1498.136047) | 1270.696594(1151.924816 to 1386.628008) | 22034.5641(20043.75429 to 24030.745) |
| Male | 1995 | 1203.53179(956.0750397 to 1499.157039) | 1271.079684(1145.732148 to 1379.069638) | 22000.68638(19951.8633 to 23883.50582) |
| Male | 1996 | 1201.702138(955.5952366 to 1496.26053) | 1267.092647(1158.31932 to 1374.703838) | 21865.31936(20065.20731 to 23673.77286) |
| Male | 1997 | 1195.545183(951.0123048 to 1487.830518) | 1256.330618(1151.871457 to 1357.410755) | 21607.16308(19883.51503 to 23300.3088) |
| Male | 1998 | 1186.834501(943.7601004 to 1477.218241) | 1250.208432(1144.491809 to 1353.661653) | 21469.13676(19750.3118 to 23253.92878) |
| Male | 1999 | 1177.410567(935.3613154 to 1466.536026) | 1235.949194(1131.789149 to 1336.014858) | 21194.64229(19556.43487 to 22894.37467) |
| Male | 2000 | 1169.241078(928.0561166 to 1457.701895) | 1251.457405(1150.816608 to 1349.374744) | 21350.59587(19715.51988 to 22998.73684) |
| Male | 2001 | 1163.398696(922.9487324 to 1451.013583) | 1261.39088(1158.344823 to 1360.48655) | 21432.18164(19794.26243 to 23112.2727) |
| Male | 2002 | 1159.643131(919.1712692 to 1447.178077) | 1254.491545(1153.141724 to 1351.855746) | 21336.12299(19720.7413 to 22960.60312) |
| Male | 2003 | 1156.805963(916.2573029 to 1444.911136) | 1274.340577(1172.706041 to 1368.021584) | 21541.03905(19943.15352 to 23092.60467) |
| Male | 2004 | 1154.107918(913.9249562 to 1440.786558) | 1273.849307(1176.746626 to 1366.384054) | 21510.29673(19944.65248 to 23084.39871) |
| Male | 2005 | 1151.660761(911.84271 to 1436.842994) | 1239.897722(1146.533159 to 1325.289953) | 20957.81134(19480.18581 to 22371.67339) |
| Male | 2006 | 1147.649438(909.9168935 to 1430.594489) | 1155.752891(1067.647815 to 1236.625057) | 19684.19445(18298.33329 to 21074.55028) |
| Male | 2007 | 1140.74073(905.1271652 to 1420.42946) | 1131.986057(1040.664377 to 1213.721678) | 19247.94347(17806.90481 to 20663.91563) |
| Male | 2008 | 1131.214192(898.5807838 to 1408.326875) | 1143.588899(1053.220133 to 1230.793274) | 19379.83826(17974.36144 to 20842.17954) |
| Male | 2009 | 1121.518837(891.4864704 to 1393.387588) | 1153.15024(1059.352221 to 1240.846295) | 19458.12221(17981.3795 to 20891.9694) |
| Male | 2010 | 1112.540961(882.8255961 to 1378.703804) | 1151.501944(1058.086319 to 1239.52517) | 19368.71725(17866.34736 to 20872.98304) |
| Male | 2011 | 1104.116469(876.6001319 to 1366.957012) | 1122.166782(1023.303616 to 1220.45241) | 18925.03753(17320.73014 to 20609.55391) |
| Male | 2012 | 1094.289134(869.1409434 to 1353.233133) | 1092.159478(995.3613804 to 1187.897559) | 18460.20223(16888.82007 to 20055.62791) |
| Male | 2013 | 1085.468457(863.039781 to 1341.891778) | 1075.41042(970.9452732 to 1177.480745) | 18098.03956(16473.43911 to 19780.61415) |
| Male | 2014 | 1080.804077(860.4089574 to 1335.645437) | 1051.983629(948.1287704 to 1156.451894) | 17686.55898(16052.53108 to 19421.7406) |
| Male | 2015 | 1081.466432(861.698337 to 1335.134487) | 1032.265288(923.5038199 to 1139.933839) | 17377.76247(15626.91576 to 19179.98502) |
| Male | 2016 | 1089.521062(868.3747919 to 1343.856037) | 1020.000318(901.3702215 to 1138.694517) | 17174.93826(15353.59731 to 19153.63383) |
| Male | 2017 | 1100.70582(877.3382386 to 1356.807338) | 993.5317442(877.149604 to 1124.742793) | 16778.0003(14888.82507 to 18933.29494) |
| Male | 2018 | 1110.671874(884.9254433 to 1369.182932) | 974.2579059(853.4784975 to 1097.092343) | 16482.11038(14573.47832 to 18544.52204) |
| Male | 2019 | 1116.603923(889.8711117 to 1378.487084) | 965.716512(843.5816673 to 1098.431559) | 16319.15778(14313.42611 to 18500.56885) |
| Male | 2020 | 1081.034837(851.7188834 to 1348.185119) | 962.4719726(825.6178373 to 1091.24535) | 16234.27934(14048.83701 to 18397.60745) |
| Male | 2021 | 1086.104053(856.5269103 to 1356.582778) | 958.8291277(831.0926805 to 1097.681395) | 16143.00618(14101.16898 to 18351.6725) |
| Female | 1990 | 996.170898(782.0425742 to 1249.359274) | 1152.841285(1006.134082 to 1319.3344) | 19547.18608(17262.78279 to 22222.00241) |
| Female | 1991 | 998.5120947(786.7915482 to 1247.06716) | 1133.054035(999.3133517 to 1287.70101) | 19194.89408(17115.06697 to 21685.52113) |
| Female | 1992 | 999.8934978(790.9040977 to 1246.928997) | 1128.78426(994.3763166 to 1285.78186) | 19076.81637(17016.96796 to 21678.68347) |
| Female | 1993 | 1000.354929(794.1127007 to 1244.912496) | 1115.185564(980.157953 to 1262.498762) | 18821.53973(16778.79895 to 21186.00508) |
| Female | 1994 | 999.9308763(795.6120076 to 1241.042787) | 1107.105357(976.9314955 to 1245.659204) | 18642.27229(16707.49506 to 20889.12734) |
| Female | 1995 | 998.7438538(796.7496482 to 1237.002408) | 1093.730378(973.7053434 to 1223.634328) | 18358.30289(16585.46834 to 20416.09351) |
| Female | 1996 | 995.8438394(795.1871671 to 1232.074858) | 1075.947598(954.8893044 to 1202.525182) | 18043.33307(16283.58638 to 20087.57771) |
| Female | 1997 | 990.3474282(790.7887563 to 1224.742904) | 1047.979498(933.7787145 to 1163.798305) | 17600.78157(15941.75802 to 19429.03922) |
| Female | 1998 | 983.0559289(784.4675545 to 1214.956909) | 1030.404846(919.4083762 to 1140.109039) | 17304.55401(15651.82463 to 18990.67769) |
| Female | 1999 | 975.0855521(777.3324569 to 1205.386631) | 1016.691049(905.0180225 to 1124.907163) | 17045.90389(15448.36231 to 18729.23617) |
| Female | 2000 | 967.4462456(770.6870626 to 1196.489819) | 1010.363181(901.1905505 to 1110.74119) | 16890.64028(15337.84411 to 18417.49149) |
| Female | 2001 | 960.5673765(765.1294573 to 1188.924881) | 1006.982966(898.1110463 to 1108.604139) | 16751.15492(15153.33395 to 18363.7464) |
| Female | 2002 | 953.7146399(759.446781 to 1181.907951) | 999.65088(893.2861909 to 1087.012327) | 16606.32038(15143.70604 to 17998.11628) |
| Female | 2003 | 946.3143441(753.0199833 to 1174.225429) | 991.5246976(886.242364 to 1079.09214) | 16449.64921(14972.6458 to 17773.95165) |
| Female | 2004 | 937.5808647(745.5089383 to 1165.185712) | 981.6132574(879.9943223 to 1067.164648) | 16238.21049(14822.03656 to 17544.24968) |
| Female | 2005 | 927.2637428(736.6613405 to 1153.933823) | 950.7585357(856.7908917 to 1031.812591) | 15710.54781(14406.70081 to 16933.47131) |
| Female | 2006 | 913.5683311(726.8001534 to 1137.176823) | 889.205159(794.0989943 to 961.7390013) | 14777.04251(13465.13578 to 15858.81724) |
| Female | 2007 | 895.888542(714.2289447 to 1114.273456) | 858.8676601(765.4386926 to 932.979394) | 14285.15065(13011.99743 to 15421.59221) |
| Female | 2008 | 876.095148(698.3253026 to 1087.099044) | 854.0471265(755.7933956 to 928.0783719) | 14145.26997(12825.02135 to 15270.15368) |
| Female | 2009 | 857.0757258(683.7020453 to 1062.301465) | 845.8772179(752.4029232 to 920.1413603) | 13977.0287(12672.12392 to 15090.99606) |
| Female | 2010 | 841.2838958(671.9226627 to 1041.958802) | 831.6883878(737.299938 to 905.048991) | 13721.07294(12400.24363 to 14832.97467) |
| Female | 2011 | 827.1974181(661.5457675 to 1023.168712) | 806.9795007(715.0555868 to 878.4362477) | 13326.13466(12068.22521 to 14418.79728) |
| Female | 2012 | 812.542515(650.3547802 to 1003.85957) | 769.9389213(678.0027863 to 843.414147) | 12788.0884(11509.58356 to 13918.12211) |
| Female | 2013 | 799.838958(640.050481 to 987.6723868) | 746.3824263(655.2644385 to 818.1366906) | 12395.32809(11118.16384 to 13495.67407) |
| Female | 2014 | 791.6105007(633.3833612 to 977.1791371) | 726.9868095(636.9546265 to 799.9488141) | 12071.23023(10834.38013 to 13226.57683) |
| Female | 2015 | 789.4302163(631.7337014 to 974.3367481) | 709.0813408(616.7227226 to 784.7667009) | 11824.20078(10538.0708 to 13018.43225) |
| Female | 2016 | 798.2467281(638.7406018 to 985.1335607) | 700.0255324(606.2583807 to 778.2294765) | 11687.17935(10394.77417 to 12898.19803) |
| Female | 2017 | 815.3271829(652.4703088 to 1007.84854) | 688.877918(595.3339792 to 773.519144) | 11524.06945(10150.64414 to 12810.28084) |
| Female | 2018 | 832.3115685(665.4047352 to 1032.823231) | 678.6658229(584.3588257 to 765.1994099) | 11381.03084(10020.57209 to 12750.77678) |
| Female | 2019 | 841.6097333(671.6180355 to 1046.736551) | 669.4165873(568.8576376 to 759.4280015) | 11233.92369(9759.611703 to 12616.08596) |
| Female | 2020 | 817.5082396(645.963746 to 1027.199422) | 659.5250871(560.4105332 to 755.8209797) | 11079.3942(9663.963914 to 12539.98456) |
| Female | 2021 | 822.8528513(648.8602941 to 1036.763438) | 650.4649296(545.4752485 to 746.0486141) | 10968.56391(9454.528946 to 12397.87327) |
| Both | 1990 | 1074.816434(860.2206789 to 1328.563023) | 1215.482298(1085.252763 to 1346.966558) | 20927.59107(18902.07062 to 23109.33465) |
| Both | 1991 | 1082.906871(870.3592168 to 1334.784594) | 1199.732298(1083.568218 to 1322.079491) | 20641.82566(18851.63557 to 22630.57655) |
| Both | 1992 | 1089.257026(878.8204135 to 1338.942139) | 1197.717211(1078.765365 to 1322.37264) | 20564.59852(18709.3951 to 22620.38428) |
| Both | 1993 | 1093.700996(884.7473566 to 1340.556267) | 1190.200104(1072.525238 to 1303.413312) | 20406.53434(18643.98416 to 22257.93244) |
| Both | 1994 | 1096.133713(889.4929702 to 1340.151257) | 1183.169842(1075.678346 to 1290.116462) | 20242.52182(18667.65165 to 21990.1443) |
| Both | 1995 | 1096.718962(891.9229822 to 1338.477928) | 1176.012782(1067.926936 to 1271.656837) | 20074.57563(18425.14437 to 21601.00668) |
| Both | 1996 | 1094.448101(890.6032251 to 1335.890388) | 1163.926232(1057.018487 to 1258.972053) | 19836.80387(18267.62014 to 21382.64691) |
| Both | 1997 | 1088.843881(886.0132872 to 1330.116871) | 1142.779725(1042.020122 to 1230.830342) | 19468.36305(18000.12663 to 20907.88792) |
| Both | 1998 | 1081.11092(879.0242431 to 1322.054704) | 1129.92303(1029.929402 to 1220.095425) | 19240.23665(17808.64975 to 20658.63622) |
| Both | 1999 | 1072.741867(871.3760214 to 1313.272164) | 1115.831151(1019.322462 to 1206.237064) | 18973.51397(17562.9115 to 20387.78932) |
| Both | 2000 | 1065.110589(864.4674951 to 1305.255651) | 1118.595558(1023.727481 to 1199.356878) | 18953.18807(17571.98768 to 20215.38608) |
| Both | 2001 | 1058.970305(858.9234179 to 1298.058103) | 1120.994129(1025.840664 to 1201.580088) | 18912.51884(17489.80429 to 20199.03294) |
| Both | 2002 | 1053.767686(854.4806485 to 1292.188324) | 1114.090028(1022.131339 to 1189.470691) | 18793.53672(17463.21074 to 19979.48846) |
| Both | 2003 | 1048.692763(849.9613921 to 1286.45216) | 1117.369038(1026.114609 to 1189.697518) | 18790.9084(17477.74357 to 19925.42409) |
| Both | 2004 | 1042.903134(844.731376 to 1279.971898) | 1111.822973(1022.351515 to 1180.542389) | 18663.91119(17370.25247 to 19769.71042) |
| Both | 2005 | 1036.228247(838.8617612 to 1272.39046) | 1079.919658(997.0449966 to 1146.508044) | 18127.55329(16933.87324 to 19201.88903) |
| Both | 2006 | 1026.920803(831.6405276 to 1259.300318) | 1008.683472(923.8209179 to 1070.890202) | 17043.36703(15863.8861 to 18032.73374) |
| Both | 2007 | 1014.09375(822.2129529 to 1242.133612) | 980.8389135(897.9858523 to 1043.805761) | 16572.52014(15395.26162 to 17561.13369) |
| Both | 2008 | 998.9022557(810.4114792 to 1221.192775) | 983.2546244(898.9958374 to 1050.061855) | 16557.78002(15360.26152 to 17641.64049) |
| Both | 2009 | 984.0472044(798.7183437 to 1201.018774) | 982.6271416(902.2623454 to 1046.351376) | 16499.05998(15386.93352 to 17501.75402) |
| Both | 2010 | 971.1776828(788.7200369 to 1183.587199) | 973.7647412(891.0382454 to 1038.680544) | 16315.62848(15142.49655 to 17342.80905) |
| Both | 2011 | 959.4350342(780.7141767 to 1168.032868) | 947.1843713(867.383976 to 1013.317543) | 15900.68316(14693.1525 to 16949.8031) |
| Both | 2012 | 946.7423079(771.2076305 to 1151.127543) | 912.8831273(830.1814796 to 979.6038157) | 15391.68351(14166.47561 to 16490.33344) |
| Both | 2013 | 935.6504966(763.1329084 to 1136.455697) | 891.7934511(808.7298019 to 962.7700424) | 15005.34454(13817.83832 to 16140.77553) |
| Both | 2014 | 928.9698132(758.1631514 to 1128.300151) | 870.5682729(788.0652759 to 942.5540581) | 14639.59581(13428.6354 to 15759.11109) |
| Both | 2015 | 928.053202(757.7079348 to 1127.209916) | 851.6020711(763.5009534 to 923.9570252) | 14360.97072(13072.8642 to 15539.64923) |
| Both | 2016 | 936.5595399(764.4227485 to 1137.098334) | 841.0662372(745.8146379 to 917.7820709) | 14192.64116(12836.45614 to 15397.02946) |
| Both | 2017 | 951.0221773(775.6306566 to 1154.143962) | 823.5489224(736.3886921 to 907.8885443) | 13926.23168(12634.40582 to 15276.79727) |
| Both | 2018 | 964.9479933(785.8053711 to 1170.696549) | 809.4925246(718.3755506 to 888.3557372) | 13714.45013(12356.16894 to 14979.90781) |
| Both | 2019 | 972.8432938(791.0824558 to 1181.252571) | 800.3506768(706.7751841 to 883.1304157) | 13557.44563(12106.53962 to 14886.60128) |
| Both | 2020 | 943.2741647(760.6233241 to 1158.539368) | 792.9047083(691.7324379 to 875.4822092) | 13428.13378(11930.35356 to 14749.50121) |
| Both | 2021 | 948.4471637(764.3465342 to 1165.446448) | 785.3991733(685.6859536 to 873.1366578) | 13315.79496(11789.51838 to 14686.01517) |

**Supplementary Table 3-1**

| location_name | sex_name | year | Stroke_Incidence_Age-standardized_Rate | Stroke_Deaths_Age-standardized_Rate | Stroke_DALYs (Disability-Adjusted Life Years)_Age-standardized_Rate |
| --- | --- | --- | --- | --- | --- |
| Andean Latin America | Male | 1990 | 577.9723244(458.9546699 to 715.2901404) | 476.177712(394.2969628 to 573.6350876) | 8581.658865(7165.238271 to 10280.97549) |
| Andean Latin America | Male | 1991 | 572.5504399(456.6326694 to 704.5195091) | 448.2302604(373.0610424 to 533.6919385) | 8071.622674(6780.576929 to 9565.450346) |
| Andean Latin America | Male | 1992 | 566.062449(452.6008939 to 695.4264577) | 454.7541432(379.5685878 to 541.7366723) | 8145.299529(6849.637484 to 9666.901972) |
| Andean Latin America | Male | 1993 | 558.6812282(446.8888727 to 685.5701761) | 456.3430206(382.106194 to 544.3023132) | 8129.20666(6873.268308 to 9635.837062) |
| Andean Latin America | Male | 1994 | 550.651122(439.8003281 to 674.0343451) | 444.8034062(375.0625836 to 525.4289158) | 7907.85837(6743.439877 to 9259.109867) |
| Andean Latin America | Male | 1995 | 542.2421943(432.5041888 to 664.9349572) | 436.69858(367.124619 to 516.0571516) | 7733.935288(6567.700817 to 9067.980761) |
| Andean Latin America | Male | 1996 | 531.6295955(425.448683 to 650.0959986) | 413.4993112(348.0179234 to 488.1205859) | 7283.652681(6171.393401 to 8539.923586) |
| Andean Latin America | Male | 1997 | 518.0590224(415.1011085 to 633.1386894) | 393.1255355(329.3986457 to 465.5125934) | 6934.131417(5855.602129 to 8164.030298) |
| Andean Latin America | Male | 1998 | 503.466267(403.5440818 to 616.3672751) | 395.2907906(330.591818 to 467.4802084) | 6934.354779(5843.138117 to 8150.116872) |
| Andean Latin America | Male | 1999 | 489.8279645(391.9321028 to 601.9027639) | 374.5941563(314.337134 to 438.749492) | 6549.260799(5557.206957 to 7622.585512) |
| Andean Latin America | Male | 2000 | 479.0832945(382.2801391 to 591.2385932) | 365.4914182(305.2592434 to 430.110993) | 6381.003669(5379.01955 to 7446.610468) |
| Andean Latin America | Male | 2001 | 471.0674175(376.9523005 to 579.18389) | 352.0903397(294.2631908 to 415.925112) | 6150.531985(5183.363857 to 7201.32868) |
| Andean Latin America | Male | 2002 | 463.9954684(371.2757327 to 568.368172) | 348.1422464(289.6850514 to 410.1863037) | 6056.059091(5074.618696 to 7101.894091) |
| Andean Latin America | Male | 2003 | 457.5190737(365.5097023 to 558.6833241) | 340.1222005(283.5056881 to 401.4585033) | 5904.426985(4953.698255 to 6914.30649) |
| Andean Latin America | Male | 2004 | 451.2562488(360.2633255 to 552.3364327) | 330.1348292(275.9599209 to 389.8612772) | 5748.478029(4856.633738 to 6737.862406) |
| Andean Latin America | Male | 2005 | 444.7893778(353.8275167 to 547.3969467) | 318.5489375(269.0855201 to 374.3704852) | 5562.717254(4742.101871 to 6486.256844) |
| Andean Latin America | Male | 2006 | 437.2782125(350.0772423 to 535.7279586) | 310.2533808(260.8303677 to 365.1983428) | 5428.019976(4628.371016 to 6329.630347) |
| Andean Latin America | Male | 2007 | 428.8795032(344.0618081 to 524.9998355) | 301.9233663(253.5336991 to 356.3962549) | 5265.402438(4478.405301 to 6179.151763) |
| Andean Latin America | Male | 2008 | 420.6628725(338.4670426 to 514.5071656) | 290.8766527(244.4705155 to 344.5238589) | 5085.7312(4324.60038 to 5982.911656) |
| Andean Latin America | Male | 2009 | 413.7593145(332.0108155 to 507.601386) | 286.7522461(239.3265666 to 341.9872733) | 5029.187289(4245.185035 to 5954.112048) |
| Andean Latin America | Male | 2010 | 409.1618898(327.4914724 to 503.7027585) | 285.503502(236.8662419 to 342.89371) | 4991.422665(4194.010393 to 5933.403461) |
| Andean Latin America | Male | 2011 | 406.1809141(327.1717869 to 497.9270047) | 282.4294976(234.4298082 to 337.7834785) | 4927.583657(4138.844724 to 5834.407346) |
| Andean Latin America | Male | 2012 | 403.2466394(326.1022688 to 493.6495807) | 272.4388519(225.9871705 to 325.0260971) | 4747.157463(3980.38982 to 5609.758898) |
| Andean Latin America | Male | 2013 | 400.7005828(322.9504583 to 490.9720151) | 268.0744051(221.9802135 to 319.3805939) | 4647.219023(3899.438776 to 5494.171959) |
| Andean Latin America | Male | 2014 | 398.8431782(320.7252887 to 488.6806473) | 261.3034793(217.3186963 to 310.9792674) | 4539.979378(3816.975438 to 5363.499479) |
| Andean Latin America | Male | 2015 | 397.9715289(318.8577648 to 490.6474186) | 257.5263385(213.4138194 to 306.1156831) | 4464.171917(3736.94665 to 5262.504972) |
| Andean Latin America | Male | 2016 | 398.4874107(320.3392393 to 488.8973397) | 262.1625833(215.2210614 to 314.2737744) | 4527.027128(3764.632118 to 5367.437358) |
| Andean Latin America | Male | 2017 | 399.825147(322.5805673 to 488.3555591) | 268.1808712(219.1227999 to 322.4621839) | 4641.5187(3839.159233 to 5520.845032) |
| Andean Latin America | Male | 2018 | 401.214448(322.8750707 to 491.8241443) | 270.778332(220.1532619 to 329.3055561) | 4705.246259(3862.725597 to 5672.878849) |
| Andean Latin America | Male | 2019 | 401.9421442(322.3563194 to 493.5685595) | 277.4160421(220.482654 to 342.5378639) | 4802.909474(3867.934977 to 5883.078889) |
| Andean Latin America | Male | 2020 | 392.6689048(311.4077708 to 486.8989711) | 265.8625867(208.827281 to 332.1957685) | 4663.292881(3712.789811 to 5782.895425) |
| Andean Latin America | Male | 2021 | 392.7521951(312.8935036 to 484.4906694) | 260.4104347(202.5533495 to 330.1441194) | 4592.283258(3623.983909 to 5774.246079) |
| Australasia | Male | 1990 | 840.9721748(698.2509998 to 992.6845176) | 488.9309087(436.0841106 to 536.2899535) | 7994.735424(7203.955006 to 8737.164861) |
| Australasia | Male | 1991 | 826.3282779(693.5226815 to 969.3102882) | 466.9792962(416.2753093 to 512.2974897) | 7651.300067(6887.188808 to 8360.031112) |
| Australasia | Male | 1992 | 811.0539926(685.7684009 to 947.5647893) | 460.567022(410.4176061 to 504.6385224) | 7493.542808(6752.915468 to 8196.994069) |
| Australasia | Male | 1993 | 795.3032052(674.9671822 to 927.8777157) | 454.6573144(404.9227496 to 498.2553733) | 7353.146042(6621.658888 to 8047.491341) |
| Australasia | Male | 1994 | 779.1129827(662.4013591 to 910.4314734) | 461.7764631(410.798807 to 506.8183007) | 7391.373149(6652.561137 to 8096.803691) |
| Australasia | Male | 1995 | 762.6133531(645.5895319 to 892.9194234) | 448.3089661(397.524116 to 493.5135923) | 7174.797378(6441.129209 to 7882.745456) |
| Australasia | Male | 1996 | 744.8098692(632.1045029 to 871.4675098) | 430.1834575(380.8484005 to 474.1294507) | 6896.777472(6181.914683 to 7577.691358) |
| Australasia | Male | 1997 | 725.3275256(614.9892078 to 850.0433797) | 396.0073967(350.3748978 to 437.8000997) | 6428.59587(5756.446798 to 7058.705474) |
| Australasia | Male | 1998 | 705.4848014(594.448872 to 827.1654533) | 386.446721(341.285889 to 427.2905138) | 6248.569053(5586.737305 to 6881.060274) |
| Australasia | Male | 1999 | 686.5366361(575.8885204 to 808.109664) | 375.386644(331.1373791 to 414.0110402) | 6049.561138(5416.92263 to 6650.370173) |
| Australasia | Male | 2000 | 669.7371392(556.6701522 to 792.3788649) | 358.3811351(315.6447205 to 395.7743015) | 5775.913361(5177.101062 to 6357.148959) |
| Australasia | Male | 2001 | 655.5684746(547.8723684 to 773.1204506) | 343.1353606(301.6999932 to 379.1417437) | 5519.603279(4945.385038 to 6072.863472) |
| Australasia | Male | 2002 | 642.9890935(539.3972065 to 756.2385681) | 333.6689732(293.6741949 to 368.2375589) | 5356.053015(4809.318526 to 5890.039919) |
| Australasia | Male | 2003 | 630.9186803(529.9876405 to 742.0659016) | 313.3812178(275.1225348 to 346.1638091) | 5044.467648(4521.384172 to 5557.049528) |
| Australasia | Male | 2004 | 618.1162208(517.943688 to 727.8442678) | 297.179238(260.1382529 to 328.462125) | 4788.585317(4286.061692 to 5279.989617) |
| Australasia | Male | 2005 | 603.4275161(503.5109498 to 712.5503767) | 279.3354193(244.3408812 to 309.6717924) | 4541.119302(4045.25711 to 5009.902193) |
| Australasia | Male | 2006 | 582.8765205(487.6105399 to 686.3292842) | 264.6443651(230.9236167 to 292.8761951) | 4325.273133(3850.036677 to 4770.435611) |
| Australasia | Male | 2007 | 556.5121147(466.8969945 to 653.2565859) | 261.0467944(227.4635439 to 288.768343) | 4233.951526(3754.127494 to 4665.813919) |
| Australasia | Male | 2008 | 529.2301676(443.8691867 to 620.7541105) | 257.2322896(223.7552961 to 284.4015879) | 4142.591248(3669.308278 to 4568.688817) |
| Australasia | Male | 2009 | 505.9211091(424.0939697 to 595.4705246) | 244.4253906(212.9236533 to 270.485793) | 3962.943996(3504.803056 to 4371.326332) |
| Australasia | Male | 2010 | 491.5204975(409.1238164 to 580.7870378) | 234.4337354(203.6137194 to 259.6356689) | 3819.625796(3373.155646 to 4218.395118) |
| Australasia | Male | 2011 | 483.3787001(402.6197607 to 571.6263448) | 225.9560951(196.7160479 to 250.2975502) | 3695.0907(3277.702282 to 4080.779433) |
| Australasia | Male | 2012 | 475.9246966(395.9928271 to 563.4444238) | 214.7352468(186.8045949 to 238.1402109) | 3531.612745(3124.084671 to 3908.68292) |
| Australasia | Male | 2013 | 469.681585(389.0856717 to 558.8894985) | 204.9973208(178.0879251 to 228.2797973) | 3392.532886(2992.91515 to 3760.30228) |
| Australasia | Male | 2014 | 465.1161336(382.7142721 to 557.3382483) | 202.4925366(176.1369961 to 225.618213) | 3359.69473(2962.918662 to 3723.883512) |
| Australasia | Male | 2015 | 462.7752147(375.7647955 to 559.7027481) | 199.6174186(173.5997118 to 222.5967295) | 3316.929(2924.395106 to 3684.540879) |
| Australasia | Male | 2016 | 462.2819285(377.2119065 to 554.6285013) | 190.1265461(164.9370155 to 212.4887075) | 3201.913737(2822.946501 to 3548.763414) |
| Australasia | Male | 2017 | 462.4341933(378.6415263 to 552.6430735) | 186.5177534(161.2825751 to 208.0173884) | 3145.060086(2761.944378 to 3488.441427) |
| Australasia | Male | 2018 | 462.5982325(378.783814 to 552.743168) | 178.5262936(154.1472929 to 199.8124445) | 3040.916764(2664.634428 to 3378.683362) |
| Australasia | Male | 2019 | 462.09772(377.5108794 to 554.4611361) | 178.6249241(153.5832549 to 200.1105925) | 3044.152862(2671.26003 to 3385.249406) |
| Australasia | Male | 2020 | 449.7641378(359.6457173 to 550.4019923) | 167.1339402(142.4374886 to 187.6524131) | 2900.343423(2536.339625 to 3238.200985) |
| Australasia | Male | 2021 | 448.939084(355.4447825 to 548.6340441) | 172.5780193(147.5968286 to 194.1349379) | 2958.753669(2593.497851 to 3312.919154) |
| Caribbean | Male | 1990 | 687.1512938(554.9528161 to 834.7441947) | 676.1054405(607.8249584 to 740.9317775) | 11872.24657(10696.98911 to 13071.81474) |
| Caribbean | Male | 1991 | 683.4538584(555.0894636 to 826.8547907) | 653.2606802(584.6770229 to 718.4943155) | 11477.7037(10292.19826 to 12672.83797) |
| Caribbean | Male | 1992 | 679.7569782(554.4765369 to 818.9013688) | 656.5012772(585.4726527 to 724.6896155) | 11497.93813(10281.56026 to 12746.56174) |
| Caribbean | Male | 1993 | 676.0759322(552.5115611 to 814.2134288) | 659.6115438(587.056964 to 729.341346) | 11541.73624(10290.0481 to 12828.75337) |
| Caribbean | Male | 1994 | 672.6892139(550.0667542 to 810.1747387) | 654.0971732(582.4246847 to 723.3838899) | 11453.74532(10228.37929 to 12729.07923) |
| Caribbean | Male | 1995 | 669.8514404(546.7868796 to 805.615772) | 651.2771101(581.005823 to 719.0761326) | 11392.75447(10183.69767 to 12610.45716) |
| Caribbean | Male | 1996 | 666.3994632(546.0422154 to 800.211329) | 631.6559339(565.3829763 to 696.7985302) | 11041.45321(9902.519569 to 12213.69197) |
| Caribbean | Male | 1997 | 661.4892843(541.383252 to 794.3103647) | 618.9656945(551.7685323 to 684.0333627) | 10844.11233(9709.721127 to 11992.34294) |
| Caribbean | Male | 1998 | 655.8698756(536.5201179 to 788.5285493) | 619.0373795(554.0405096 to 681.3435054) | 10860.29427(9771.24405 to 11967.69528) |
| Caribbean | Male | 1999 | 650.229039(530.9060814 to 783.1980103) | 621.4176658(556.4100989 to 684.2672414) | 10860.02936(9762.90811 to 11973.30298) |
| Caribbean | Male | 2000 | 645.2262616(526.2795831 to 779.6613836) | 601.919386(536.198148 to 664.7015383) | 10491.76357(9374.644396 to 11614.93865) |
| Caribbean | Male | 2001 | 640.4217448(522.9171155 to 771.767638) | 596.5538448(532.3519154 to 659.4032174) | 10415.96547(9320.222647 to 11527.53915) |
| Caribbean | Male | 2002 | 635.5790302(520.5281224 to 763.9330524) | 572.2371731(510.3637697 to 635.8532871) | 10000.11811(8942.785151 to 11111.45729) |
| Caribbean | Male | 2003 | 630.6373651(516.3757033 to 756.9151882) | 574.1121799(511.6275197 to 639.2639916) | 9991.028486(8952.563923 to 11144.7755) |
| Caribbean | Male | 2004 | 625.8536243(511.1792414 to 753.0821158) | 572.9805171(510.340424 to 640.7693817) | 9943.205463(8887.497283 to 11144.89478) |
| Caribbean | Male | 2005 | 621.5772745(507.2389187 to 750.1238373) | 573.0541366(511.3317623 to 639.679357) | 9933.088123(8895.201443 to 11119.41962) |
| Caribbean | Male | 2006 | 617.7843342(504.5629845 to 742.4976386) | 549.5083837(487.0514061 to 616.9884663) | 9577.218491(8534.026011 to 10784.57883) |
| Caribbean | Male | 2007 | 614.337656(502.6847106 to 737.2340807) | 545.7762913(482.0384231 to 615.5969706) | 9516.637909(8433.276701 to 10775.14356) |
| Caribbean | Male | 2008 | 611.0309778(499.7572221 to 733.5475423) | 539.5088912(474.0691725 to 611.8175035) | 9368.728281(8263.074909 to 10677.18586) |
| Caribbean | Male | 2009 | 608.0141445(496.3691588 to 731.2001816) | 564.9197785(500.726623 to 635.031227) | 9786.052339(8691.024727 to 11047.4509) |
| Caribbean | Male | 2010 | 604.6911725(492.3733853 to 730.280945) | 538.0514394(475.4080289 to 602.1071368) | 9337.901056(8287.4469 to 10477.51993) |
| Caribbean | Male | 2011 | 601.2551416(491.5475412 to 723.8852631) | 522.2514054(461.6814752 to 583.3003215) | 9114.561849(8099.743255 to 10187.95647) |
| Caribbean | Male | 2012 | 597.5574236(490.1616972 to 717.6537211) | 512.517773(453.5941351 to 574.6193709) | 8972.944526(7979.397023 to 10072.56016) |
| Caribbean | Male | 2013 | 593.9312414(487.4340588 to 712.3942671) | 517.8634518(459.9106159 to 578.7970016) | 9084.634838(8111.136789 to 10157.28782) |
| Caribbean | Male | 2014 | 591.2517206(485.5914641 to 709.778353) | 517.1260574(459.1029313 to 577.2543832) | 9120.42885(8141.37794 to 10195.95676) |
| Caribbean | Male | 2015 | 590.4650127(484.3120587 to 710.8325081) | 513.656376(455.0043187 to 574.3879152) | 9071.290572(8066.308552 to 10161.92704) |
| Caribbean | Male | 2016 | 592.1113368(486.5243981 to 709.9579735) | 509.0415083(449.2867882 to 570.167107) | 9026.961568(8010.303952 to 10145.87757) |
| Caribbean | Male | 2017 | 595.0443874(488.6482868 to 712.3109423) | 510.0076331(450.3511362 to 571.5698225) | 9069.341716(8030.634068 to 10188.07481) |
| Caribbean | Male | 2018 | 598.0491436(490.3888949 to 716.9617993) | 501.9621429(439.2623714 to 566.6168563) | 8961.470562(7864.510996 to 10146.33387) |
| Caribbean | Male | 2019 | 599.96529(490.7703412 to 721.5012124) | 498.2345797(433.1159309 to 566.9860741) | 8919.288112(7772.132392 to 10181.722) |
| Caribbean | Male | 2020 | 593.1994415(481.1874331 to 723.3456626) | 505.4098997(437.0749735 to 578.1470738) | 9060.740557(7863.930153 to 10394.6487) |
| Caribbean | Male | 2021 | 594.7561512(481.0147367 to 724.4846656) | 493.4719281(419.5076847 to 572.7595546) | 8827.03634(7491.283904 to 10271.81212) |
| Central Asia | Male | 1990 | 1336.943525(1090.032578 to 1624.419846) | 1213.274631(1136.213975 to 1274.808967) | 21994.78228(20783.14018 to 23105.63118) |
| Central Asia | Male | 1991 | 1339.050983(1097.638061 to 1614.656498) | 1264.528133(1185.617664 to 1327.505369) | 22877.95316(21625.16522 to 24027.97604) |
| Central Asia | Male | 1992 | 1343.255122(1102.423397 to 1616.065787) | 1307.413478(1229.724299 to 1368.866766) | 23579.20377(22386.44806 to 24686.32523) |
| Central Asia | Male | 1993 | 1349.853693(1112.289234 to 1618.977967) | 1383.389369(1305.328592 to 1446.234188) | 24938.04285(23732.16213 to 26050.33888) |
| Central Asia | Male | 1994 | 1358.413104(1122.615504 to 1628.406241) | 1429.38338(1348.089746 to 1496.351377) | 25753.88416(24498.24542 to 26947.75994) |
| Central Asia | Male | 1995 | 1368.472024(1131.320557 to 1639.355339) | 1430.515149(1349.452353 to 1497.654329) | 25886.67476(24598.93744 to 27053.13355) |
| Central Asia | Male | 1996 | 1383.146532(1146.641954 to 1649.956242) | 1414.168032(1331.899869 to 1482.775475) | 25669.41738(24350.98716 to 26893.82755) |
| Central Asia | Male | 1997 | 1403.012679(1162.561936 to 1668.003184) | 1379.657348(1291.321587 to 1450.659489) | 25101.02132(23690.6912 to 26371.61516) |
| Central Asia | Male | 1998 | 1424.538761(1180.500892 to 1692.110283) | 1359.695661(1270.638785 to 1432.853195) | 24749.59831(23314.14996 to 26058.25859) |
| Central Asia | Male | 1999 | 1443.051855(1197.158819 to 1714.513338) | 1349.485769(1258.72709 to 1422.195511) | 24448.13483(22991.49969 to 25745.1975) |
| Central Asia | Male | 2000 | 1454.665198(1206.84846 to 1732.33211) | 1386.617909(1294.52772 to 1459.550546) | 25081.56668(23615.68866 to 26363.65103) |
| Central Asia | Male | 2001 | 1461.074451(1214.04951 to 1735.747315) | 1364.593457(1272.374667 to 1437.486966) | 24830.19402(23357.45192 to 26083.59941) |
| Central Asia | Male | 2002 | 1467.501735(1221.050196 to 1742.485406) | 1380.298065(1284.565154 to 1455.37691) | 25092.9068(23607.56193 to 26423.7274) |
| Central Asia | Male | 2003 | 1472.709086(1225.666361 to 1750.467474) | 1422.280179(1321.213985 to 1500.458439) | 25681.49333(24109.3861 to 27082.87884) |
| Central Asia | Male | 2004 | 1476.037374(1227.92906 to 1757.252945) | 1438.062023(1332.226085 to 1519.810542) | 25895.26586(24226.421 to 27328.78214) |
| Central Asia | Male | 2005 | 1477.028159(1229.476152 to 1761.095929) | 1433.225224(1330.885113 to 1515.574371) | 25843.23993(24205.55172 to 27316.9677) |
| Central Asia | Male | 2006 | 1474.799159(1227.183118 to 1754.743764) | 1381.46243(1284.478475 to 1464.212893) | 24927.32987(23302.07942 to 26423.13834) |
| Central Asia | Male | 2007 | 1469.338744(1222.792804 to 1746.364571) | 1369.891666(1275.600732 to 1449.186224) | 24660.0522(23118.10053 to 26054.10139) |
| Central Asia | Male | 2008 | 1460.828301(1216.852702 to 1737.296371) | 1374.682118(1280.647891 to 1452.576148) | 24636.96137(23099.23277 to 25944.97691) |
| Central Asia | Male | 2009 | 1450.564136(1205.468007 to 1728.736234) | 1330.585781(1241.195391 to 1404.460238) | 23774.52666(22300.98359 to 25014.81689) |
| Central Asia | Male | 2010 | 1439.136254(1191.238719 to 1716.433415) | 1332.707613(1241.836037 to 1403.627638) | 23682.33875(22236.8296 to 24884.1487) |
| Central Asia | Male | 2011 | 1423.821481(1181.478675 to 1697.957698) | 1277.658696(1192.28484 to 1346.350594) | 22624.53907(21231.57846 to 23774.13161) |
| Central Asia | Male | 2012 | 1402.536715(1167.31371 to 1668.583388) | 1218.356919(1140.132962 to 1286.620038) | 21628.96057(20336.91631 to 22765.55949) |
| Central Asia | Male | 2013 | 1381.292985(1152.106794 to 1639.697646) | 1169.113235(1093.296915 to 1235.492541) | 20847.82115(19589.21528 to 21993.28143) |
| Central Asia | Male | 2014 | 1365.487755(1137.111011 to 1623.999126) | 1152.546678(1077.043174 to 1218.225803) | 20486.96634(19303.55401 to 21614.72735) |
| Central Asia | Male | 2015 | 1359.523505(1128.855081 to 1620.142278) | 1137.072645(1060.833225 to 1199.8501) | 20125.76936(18938.33778 to 21238.19319) |
| Central Asia | Male | 2016 | 1363.968454(1135.43737 to 1623.186514) | 1134.040189(1056.665167 to 1195.260641) | 20055.74017(18815.96981 to 21152.78214) |
| Central Asia | Male | 2017 | 1370.846847(1142.532221 to 1631.980346) | 1113.290062(1035.996652 to 1174.889212) | 19626.79511(18394.78201 to 20706.82167) |
| Central Asia | Male | 2018 | 1377.971121(1147.789086 to 1643.049219) | 1083.241673(1005.505685 to 1144.712527) | 19064.42861(17823.10929 to 20149.31199) |
| Central Asia | Male | 2019 | 1381.545553(1144.659959 to 1654.378132) | 1050.271281(973.4194325 to 1112.318332) | 18446.17871(17215.32332 to 19551.09803) |
| Central Asia | Male | 2020 | 1321.575478(1090.52531 to 1584.214458) | 1042.907606(956.4118519 to 1114.27795) | 18274.93999(16898.15646 to 19563.24642) |
| Central Asia | Male | 2021 | 1331.990663(1100.393519 to 1588.965477) | 1006.438525(901.9926785 to 1100.232519) | 17720.2111(15982.12482 to 19450.33698) |
| Central Europe | Male | 1990 | 1372.399776(1102.790407 to 1681.624136) | 1590.620436(1508.715152 to 1653.78172) | 26069.534(24861.2139 to 27097.83802) |
| Central Europe | Male | 1991 | 1367.729277(1106.44966 to 1669.540212) | 1580.420168(1497.760212 to 1642.725577) | 25939.06372(24729.41512 to 26968.94564) |
| Central Europe | Male | 1992 | 1361.738938(1107.344718 to 1655.223557) | 1580.159424(1497.642763 to 1644.871411) | 26074.44747(24862.91109 to 27113.8896) |
| Central Europe | Male | 1993 | 1354.117297(1103.461388 to 1638.692808) | 1577.224358(1493.70479 to 1640.732675) | 26054.46545(24788.32411 to 27101.54028) |
| Central Europe | Male | 1994 | 1344.936592(1097.161135 to 1625.387154) | 1562.743393(1477.686117 to 1626.389086) | 25877.42737(24588.36146 to 26941.83938) |
| Central Europe | Male | 1995 | 1335.045306(1089.553415 to 1609.276973) | 1561.755177(1479.554651 to 1622.433123) | 25939.94998(24737.6498 to 26961.77526) |
| Central Europe | Male | 1996 | 1321.577787(1082.083062 to 1588.406222) | 1552.210636(1470.429566 to 1611.649301) | 25806.44975(24601.1124 to 26810.4393) |
| Central Europe | Male | 1997 | 1302.769151(1069.441691 to 1562.837397) | 1529.447044(1450.129101 to 1588.412837) | 25526.37583(24342.38159 to 26529.01834) |
| Central Europe | Male | 1998 | 1281.738739(1054.745811 to 1535.677677) | 1465.75408(1386.646817 to 1521.11818) | 24454.68785(23284.65618 to 25424.6985) |
| Central Europe | Male | 1999 | 1261.310134(1039.944353 to 1507.934503) | 1423.080567(1346.716194 to 1477.762634) | 23715.22324(22578.32291 to 24669.41831) |
| Central Europe | Male | 2000 | 1244.307481(1025.558174 to 1487.928982) | 1373.705851(1297.948158 to 1427.207811) | 22907.3258(21790.00566 to 23819.15317) |
| Central Europe | Male | 2001 | 1230.570118(1013.700566 to 1469.312743) | 1353.893608(1279.656874 to 1406.720188) | 22479.15206(21387.52215 to 23389.20099) |
| Central Europe | Male | 2002 | 1217.394829(1001.550316 to 1451.523029) | 1347.152338(1272.061892 to 1399.650797) | 22252.39189(21158.27487 to 23150.85936) |
| Central Europe | Male | 2003 | 1204.315098(990.1324768 to 1435.845587) | 1325.856383(1250.349153 to 1377.79546) | 21838.72656(20773.51276 to 22719.25876) |
| Central Europe | Male | 2004 | 1190.617005(977.326215 to 1423.113206) | 1268.18772(1194.896071 to 1318.9067) | 20919.36997(19863.74781 to 21772.5343) |
| Central Europe | Male | 2005 | 1175.742868(962.385551 to 1406.023432) | 1222.689464(1150.357667 to 1272.300522) | 20249.86896(19203.6519 to 21091.31534) |
| Central Europe | Male | 2006 | 1158.567333(949.5476803 to 1384.428388) | 1166.379873(1095.667563 to 1213.742495) | 19357.22402(18319.3547 to 20165.17409) |
| Central Europe | Male | 2007 | 1138.96801(933.3160114 to 1361.054047) | 1116.499209(1047.774949 to 1162.074382) | 18535.53685(17540.85498 to 19313.24186) |
| Central Europe | Male | 2008 | 1118.452022(916.2669799 to 1337.92123) | 1073.273193(1006.415907 to 1117.525267) | 17809.09369(16845.6733 to 18566.31047) |
| Central Europe | Male | 2009 | 1098.265361(900.0824631 to 1314.680303) | 1042.567043(978.1259296 to 1086.682964) | 17266.24934(16344.73217 to 18028.90942) |
| Central Europe | Male | 2010 | 1079.148821(884.8594547 to 1295.232633) | 1004.436588(940.4848283 to 1047.845531) | 16631.89323(15715.34899 to 17372.05221) |
| Central Europe | Male | 2011 | 1056.133049(865.6137855 to 1267.080162) | 952.7306328(893.3460349 to 995.1233625) | 15775.8206(14891.50463 to 16503.07948) |
| Central Europe | Male | 2012 | 1027.725255(842.5981734 to 1229.613783) | 932.8485193(874.7212289 to 974.7707733) | 15401.84009(14547.46352 to 16109.71904) |
| Central Europe | Male | 2013 | 999.1565367(819.2464801 to 1194.429903) | 889.8561368(832.9866838 to 930.7955839) | 14681.4182(13851.89977 to 15371.92467) |
| Central Europe | Male | 2014 | 975.5219323(799.5539026 to 1165.685831) | 870.5022676(814.5072792 to 911.1892623) | 14345.98014(13523.64064 to 15024.17502) |
| Central Europe | Male | 2015 | 961.9494882(786.5041358 to 1149.227636) | 851.1169533(796.3502569 to 891.5713807) | 14033.59751(13230.09713 to 14710.90762) |
| Central Europe | Male | 2016 | 956.8065125(782.0968701 to 1145.272646) | 818.0905088(762.2495876 to 858.530093) | 13524.19761(12715.48349 to 14202.03254) |
| Central Europe | Male | 2017 | 954.0924242(777.4457078 to 1143.410477) | 802.7209861(748.6174061 to 843.4827828) | 13281.38532(12497.08842 to 13957.91898) |
| Central Europe | Male | 2018 | 951.3307829(773.8196747 to 1143.38856) | 788.4183121(733.8549461 to 828.6779607) | 13085.60701(12294.7376 to 13754.782) |
| Central Europe | Male | 2019 | 946.2133034(768.1202221 to 1141.565321) | 766.2636589(711.7977725 to 807.2716485) | 12752.36911(11960.95302 to 13441.27326) |
| Central Europe | Male | 2020 | 914.5390746(736.3997353 to 1108.836837) | 764.0869435(701.3970861 to 816.9836838) | 12708.90131(11764.9478 to 13556.7607) |
| Central Europe | Male | 2021 | 911.3411703(733.2821087 to 1112.74332) | 742.632385(671.6315653 to 801.3001083) | 12389.49335(11312.35175 to 13356.70054) |
| Central Latin America | Male | 1990 | 673.3522861(531.4285693 to 844.6649733) | 439.383615(410.8026159 to 460.4118471) | 7505.379206(7092.313193 to 7855.772365) |
| Central Latin America | Male | 1991 | 666.4165683(526.5062483 to 831.6542943) | 443.7769635(415.1111235 to 465.0966007) | 7596.48839(7180.017669 to 7954.928842) |
| Central Latin America | Male | 1992 | 659.0883123(521.5491693 to 820.4039205) | 441.2444844(413.025933 to 462.9432431) | 7577.859222(7162.274974 to 7935.826621) |
| Central Latin America | Male | 1993 | 651.5242101(516.2113887 to 809.8501795) | 434.9755302(407.0120342 to 456.6942771) | 7496.86646(7087.570309 to 7862.817381) |
| Central Latin America | Male | 1994 | 643.8588092(510.919204 to 798.2471854) | 437.5700263(409.4239042 to 459.7312592) | 7540.363037(7123.326812 to 7912.013262) |
| Central Latin America | Male | 1995 | 636.2774856(505.3016618 to 786.9269715) | 432.521427(403.8807241 to 455.5319693) | 7409.823849(6997.921306 to 7785.201487) |
| Central Latin America | Male | 1996 | 628.0709132(499.2825136 to 774.8894668) | 426.4546529(397.9004719 to 448.1050411) | 7279.909448(6882.316434 to 7644.824885) |
| Central Latin America | Male | 1997 | 618.766598(491.8007789 to 762.926185) | 412.2632489(384.5385056 to 433.712657) | 7022.551369(6620.797706 to 7376.052931) |
| Central Latin America | Male | 1998 | 608.8387236(483.6477909 to 749.4941771) | 402.8638717(376.0358249 to 424.20398) | 6877.770172(6488.747514 to 7227.047704) |
| Central Latin America | Male | 1999 | 598.6626736(475.1224071 to 737.4003512) | 390.5408992(363.8197616 to 410.596316) | 6683.268364(6294.038058 to 7022.364458) |
| Central Latin America | Male | 2000 | 588.7129787(467.162838 to 726.7931343) | 377.9470416(351.6791385 to 397.5896111) | 6494.398502(6119.567464 to 6828.211299) |
| Central Latin America | Male | 2001 | 578.0888959(459.6672221 to 712.8581775) | 365.6032713(339.9618218 to 384.8122856) | 6287.99771(5926.376856 to 6615.74818) |
| Central Latin America | Male | 2002 | 566.1667015(451.0839742 to 697.4706345) | 355.3271885(330.5514185 to 374.0962349) | 6110.897227(5759.070765 to 6419.968572) |
| Central Latin America | Male | 2003 | 553.8832875(441.7917336 to 682.5559315) | 350.5603705(326.4292978 to 369.1591221) | 6028.051794(5685.88743 to 6330.735457) |
| Central Latin America | Male | 2004 | 542.1876397(432.5374035 to 668.5561346) | 334.7206267(311.3405807 to 352.3586632) | 5766.348495(5435.149368 to 6064.911541) |
| Central Latin America | Male | 2005 | 532.0372333(423.4920815 to 656.7688813) | 323.7033144(301.1327176 to 340.9254658) | 5586.462698(5272.977231 to 5872.162482) |
| Central Latin America | Male | 2006 | 522.5048951(417.7579583 to 644.1591621) | 319.6218037(297.3465433 to 336.4726627) | 5513.54916(5200.860174 to 5799.479575) |
| Central Latin America | Male | 2007 | 512.6902284(409.8417666 to 631.5518729) | 307.3200688(285.304346 to 323.7617769) | 5317.055758(4994.210666 to 5589.507693) |
| Central Latin America | Male | 2008 | 503.441864(401.9886358 to 619.2991141) | 309.9356684(287.9971392 to 326.3160303) | 5354.245273(5039.163701 to 5626.687294) |
| Central Latin America | Male | 2009 | 495.6337805(395.4146867 to 609.4131447) | 311.8037303(290.0646147 to 328.4022872) | 5371.89125(5059.996705 to 5644.894374) |
| Central Latin America | Male | 2010 | 490.0974015(390.8931397 to 602.8659852) | 300.3899941(279.0238731 to 316.2789699) | 5173.058821(4862.157991 to 5440.665613) |
| Central Latin America | Male | 2011 | 486.0918756(388.112599 to 597.0076358) | 289.8838944(268.7342373 to 305.4856728) | 5015.339438(4709.687277 to 5284.114344) |
| Central Latin America | Male | 2012 | 482.5089092(385.8138231 to 591.5995159) | 287.1353575(266.2529881 to 302.9843006) | 4971.069233(4658.115222 to 5245.209442) |
| Central Latin America | Male | 2013 | 479.5360505(384.4643852 to 587.6331849) | 286.2134628(265.4452243 to 301.9618055) | 4935.559366(4627.688649 to 5208.839762) |
| Central Latin America | Male | 2014 | 477.2991122(383.6856273 to 584.1778829) | 279.454618(258.9338412 to 295.0728069) | 4813.81331(4512.34636 to 5078.928654) |
| Central Latin America | Male | 2015 | 475.943138(383.1625811 to 582.1610491) | 276.0453916(255.2117362 to 291.4976862) | 4749.596331(4446.681008 to 5009.007417) |
| Central Latin America | Male | 2016 | 475.1760996(382.7605008 to 581.2059568) | 278.831284(257.6298196 to 294.6868457) | 4801.728464(4493.833926 to 5063.628546) |
| Central Latin America | Male | 2017 | 474.5190142(382.1513678 to 580.4130895) | 270.4845656(249.3554466 to 285.9640558) | 4668.515869(4365.049918 to 4927.850374) |
| Central Latin America | Male | 2018 | 473.7289502(380.9924373 to 579.8094998) | 268.0996377(246.5765054 to 285.1093941) | 4642.831204(4327.099564 to 4927.502799) |
| Central Latin America | Male | 2019 | 472.5219404(379.0281186 to 579.4754901) | 264.3647309(242.0367599 to 282.6465858) | 4590.795414(4249.295591 to 4900.515759) |
| Central Latin America | Male | 2020 | 465.0969342(371.0175958 to 574.9647979) | 265.4646756(237.8251735 to 292.1462925) | 4655.476578(4213.500561 to 5112.752446) |
| Central Latin America | Male | 2021 | 463.2010856(369.3954779 to 571.2826588) | 267.5637283(232.3783136 to 303.023849) | 4684.089378(4118.72865 to 5300.964131) |
| Central Sub-Saharan Africa | Male | 1990 | 1215.601287(964.1827262 to 1512.124751) | 1157.403232(862.9830699 to 1494.805385) | 21436.32884(16145.94791 to 27528.92462) |
| Central Sub-Saharan Africa | Male | 1991 | 1212.902201(970.5530934 to 1496.795335) | 1155.327624(863.9820688 to 1494.536938) | 21391.11347(16129.04082 to 27464.59044) |
| Central Sub-Saharan Africa | Male | 1992 | 1209.364901(970.8818924 to 1483.866157) | 1151.499284(863.0652157 to 1490.118414) | 21306.39548(16118.49295 to 27376.69087) |
| Central Sub-Saharan Africa | Male | 1993 | 1205.232536(970.1598843 to 1474.475232) | 1146.116145(861.3141816 to 1492.576247) | 21188.69399(16097.93517 to 27314.39536) |
| Central Sub-Saharan Africa | Male | 1994 | 1200.811253(967.2307253 to 1465.386398) | 1153.889934(872.7491075 to 1501.289898) | 21332.70141(16293.50587 to 27522.53854) |
| Central Sub-Saharan Africa | Male | 1995 | 1196.324717(962.1621786 to 1458.676023) | 1163.616082(880.2991324 to 1520.803935) | 21509.86579(16471.9825 to 27803.71553) |
| Central Sub-Saharan Africa | Male | 1996 | 1190.350899(958.7124893 to 1448.739609) | 1172.675917(887.2205986 to 1524.04558) | 21676.99001(16624.14076 to 27900.45538) |
| Central Sub-Saharan Africa | Male | 1997 | 1181.936892(953.9507836 to 1437.951634) | 1159.174074(881.6756309 to 1501.378889) | 21401.04637(16498.4767 to 27465.40189) |
| Central Sub-Saharan Africa | Male | 1998 | 1171.99937(945.447685 to 1426.578072) | 1155.362685(879.0136879 to 1494.212509) | 21322.64227(16441.44589 to 27270.30675) |
| Central Sub-Saharan Africa | Male | 1999 | 1161.544614(936.0839835 to 1416.595771) | 1146.182263(867.5803708 to 1476.634712) | 21135.89967(16231.7282 to 26951.56988) |
| Central Sub-Saharan Africa | Male | 2000 | 1151.520499(925.8543076 to 1407.043609) | 1140.4681(861.8656684 to 1472.070203) | 21004.76583(16074.38476 to 26910.35205) |
| Central Sub-Saharan Africa | Male | 2001 | 1141.483717(919.461826 to 1391.663048) | 1125.183372(846.9007556 to 1447.806221) | 20706.45131(15808.98705 to 26499.77372) |
| Central Sub-Saharan Africa | Male | 2002 | 1130.580621(911.8224416 to 1373.664783) | 1098.189001(826.2827171 to 1413.828236) | 20149.79523(15420.37308 to 25752.76877) |
| Central Sub-Saharan Africa | Male | 2003 | 1119.049154(902.5001757 to 1360.158016) | 1093.08112(824.0268868 to 1401.987997) | 20051.5732(15362.96775 to 25584.62529) |
| Central Sub-Saharan Africa | Male | 2004 | 1107.169834(890.9344428 to 1345.223774) | 1077.71623(816.3104397 to 1387.269823) | 19741.80326(15195.84481 to 25237.58911) |
| Central Sub-Saharan Africa | Male | 2005 | 1095.178023(879.4891616 to 1334.625608) | 1057.267507(802.1748692 to 1366.462261) | 19328.76148(14925.7194 to 24766.47035) |
| Central Sub-Saharan Africa | Male | 2006 | 1081.236771(871.4683121 to 1310.83906) | 1052.951361(797.3142395 to 1366.381807) | 19218.3662(14826.76433 to 24666.63655) |
| Central Sub-Saharan Africa | Male | 2007 | 1064.692962(862.6510708 to 1290.110635) | 1043.039549(790.6923759 to 1351.912377) | 19023.37937(14653.07371 to 24416.31936) |
| Central Sub-Saharan Africa | Male | 2008 | 1047.58246(851.7568265 to 1268.901926) | 1046.161705(792.701428 to 1360.983497) | 19060.74152(14662.15908 to 24561.69303) |
| Central Sub-Saharan Africa | Male | 2009 | 1032.003056(837.9547003 to 1251.125132) | 1044.117854(788.4513009 to 1367.257224) | 19019.62416(14593.05278 to 24654.86862) |
| Central Sub-Saharan Africa | Male | 2010 | 1020.020792(827.7537082 to 1240.024262) | 1042.642256(783.4115731 to 1368.563317) | 18985.47542(14474.42335 to 24627.67782) |
| Central Sub-Saharan Africa | Male | 2011 | 1009.817672(819.9671451 to 1224.44065) | 1044.159579(786.1532719 to 1379.726737) | 18998.11318(14489.1534 to 24863.79226) |
| Central Sub-Saharan Africa | Male | 2012 | 999.4860573(813.5286064 to 1210.853786) | 1041.563828(779.4994331 to 1379.150752) | 18948.86709(14387.63324 to 24820.03418) |
| Central Sub-Saharan Africa | Male | 2013 | 990.276622(806.6915631 to 1199.982003) | 1036.016828(773.3580115 to 1367.960974) | 18823.97698(14219.43659 to 24638.34798) |
| Central Sub-Saharan Africa | Male | 2014 | 983.4277676(799.3235116 to 1192.328202) | 1026.156434(764.8727525 to 1358.947645) | 18628.62244(14036.83098 to 24416.01684) |
| Central Sub-Saharan Africa | Male | 2015 | 980.2900667(794.7408764 to 1190.160953) | 1022.265393(762.0232417 to 1360.843892) | 18519.86027(13943.66145 to 24445.519) |
| Central Sub-Saharan Africa | Male | 2016 | 979.8119338(796.9643239 to 1187.827432) | 1012.951128(754.6744988 to 1349.379457) | 18337.9628(13787.15224 to 24320.14777) |
| Central Sub-Saharan Africa | Male | 2017 | 980.0132887(798.0832271 to 1186.369165) | 1006.55063(752.2207104 to 1345.246377) | 18193.16329(13688.88622 to 24120.23827) |
| Central Sub-Saharan Africa | Male | 2018 | 980.1755589(797.2601785 to 1184.741486) | 1003.657803(748.3609881 to 1344.872349) | 18107.54598(13593.03817 to 24073.76839) |
| Central Sub-Saharan Africa | Male | 2019 | 979.5795077(795.5197716 to 1183.632719) | 1003.375865(743.6933764 to 1345.783927) | 18053.37591(13455.84947 to 23992.51637) |
| Central Sub-Saharan Africa | Male | 2020 | 965.3247295(779.8359936 to 1180.679853) | 1013.196123(746.6855938 to 1354.735022) | 18204.8056(13518.54484 to 24099.77915) |
| Central Sub-Saharan Africa | Male | 2021 | 963.1376525(777.2994489 to 1171.903067) | 1010.345223(741.2620448 to 1352.908713) | 18184.78868(13430.50798 to 24098.88031) |
| East Asia | Male | 1990 | 1530.839133(1198.798517 to 1943.562028) | 1952.543463(1664.449866 to 2225.583259) | 31700.54095(26828.16558 to 36434.73346) |
| East Asia | Male | 1991 | 1564.21937(1230.85904 to 1977.965157) | 1930.790082(1655.06178 to 2188.022755) | 31308.2971(26831.936 to 35790.66346) |
| East Asia | Male | 1992 | 1592.202092(1254.876214 to 2006.460554) | 1918.617491(1666.245404 to 2172.632661) | 31062.50182(26881.8072 to 35426.62263) |
| East Asia | Male | 1993 | 1614.330414(1274.431273 to 2030.3832) | 1907.56868(1675.805243 to 2141.749537) | 30802.88823(27126.46486 to 34759.45751) |
| East Asia | Male | 1994 | 1629.94935(1289.063354 to 2046.234401) | 1891.27278(1674.014742 to 2101.88964) | 30429.86724(26870.43936 to 33987.70369) |
| East Asia | Male | 1995 | 1638.264894(1297.111377 to 2055.062691) | 1890.694658(1676.145465 to 2082.606319) | 30361.56142(26914.88686 to 33582.11551) |
| East Asia | Male | 1996 | 1636.862376(1295.762826 to 2054.072425) | 1880.644558(1679.181475 to 2074.132334) | 30112.54956(26886.54658 to 33237.65866) |
| East Asia | Male | 1997 | 1626.928214(1286.590741 to 2043.186551) | 1866.012807(1676.272113 to 2042.049846) | 29784.4661(26804.51251 to 32665.01948) |
| East Asia | Male | 1998 | 1612.922727(1274.137875 to 2026.084023) | 1864.97318(1670.151483 to 2050.261701) | 29684.80837(26606.27882 to 32747.73371) |
| East Asia | Male | 1999 | 1598.988374(1263.017738 to 2010.418497) | 1869.622707(1677.4506 to 2052.31839) | 29660.43553(26726.74863 to 32623.9113) |
| East Asia | Male | 2000 | 1589.514362(1255.608032 to 2000.706252) | 1933.888992(1749.013484 to 2121.837427) | 30457.39049(27551.75595 to 33506.15269) |
| East Asia | Male | 2001 | 1587.374298(1251.576389 to 1998.080709) | 1966.860611(1773.652624 to 2152.826968) | 30805.27881(27776.09065 to 33847.2176) |
| East Asia | Male | 2002 | 1590.642563(1251.971528 to 2002.981826) | 1962.689928(1769.037763 to 2146.35567) | 30761.72401(27698.81183 to 33698.46587) |
| East Asia | Male | 2003 | 1596.468746(1254.148181 to 2012.612744) | 2046.834272(1847.718382 to 2227.967569) | 31636.68041(28596.21019 to 34516.20595) |
| East Asia | Male | 2004 | 1601.963561(1258.307066 to 2020.99933) | 2070.684602(1878.290662 to 2258.754176) | 31907.94506(28950.93922 to 34884.32704) |
| East Asia | Male | 2005 | 1604.725758(1259.322693 to 2026.236377) | 2014.937995(1826.960844 to 2184.332126) | 30995.59036(28080.21863 to 33724.0108) |
| East Asia | Male | 2006 | 1600.038221(1254.632017 to 2018.825285) | 1824.871066(1659.54495 to 1986.771732) | 28341.52517(25826.10655 to 30913.02471) |
| East Asia | Male | 2007 | 1587.364518(1244.197532 to 1999.238433) | 1769.413403(1595.721152 to 1930.450542) | 27370.188(24776.17995 to 30007.47712) |
| East Asia | Male | 2008 | 1570.21962(1231.177336 to 1976.597903) | 1751.566033(1576.485246 to 1923.869856) | 27160.98552(24515.15023 to 29925.78303) |
| East Asia | Male | 2009 | 1551.320282(1215.995472 to 1948.739994) | 1752.685245(1579.192639 to 1922.460566) | 27205.55246(24464.93983 to 29910.39426) |
| East Asia | Male | 2010 | 1534.697911(1204.067165 to 1929.697359) | 1732.219131(1546.075318 to 1902.053193) | 26932.62697(24018.07233 to 29649.60993) |
| East Asia | Male | 2011 | 1517.412512(1192.123057 to 1904.196607) | 1663.889171(1484.343124 to 1850.77774) | 26019.12203(23198.44248 to 29066.85258) |
| East Asia | Male | 2012 | 1497.727277(1176.710284 to 1875.710724) | 1604.535587(1431.249172 to 1790.07634) | 25212.57876(22486.34131 to 28244.605) |
| East Asia | Male | 2013 | 1479.988356(1165.302834 to 1851.945898) | 1560.927575(1371.542308 to 1765.456303) | 24468.11309(21589.56532 to 27707.59798) |
| East Asia | Male | 2014 | 1468.486626(1157.749284 to 1835.130267) | 1499.766313(1311.653789 to 1701.06977) | 23540.13248(20666.75252 to 26757.82237) |
| East Asia | Male | 2015 | 1467.908357(1157.779079 to 1831.381694) | 1456.717897(1255.604217 to 1663.615996) | 22900.23115(19778.4443 to 26331.01476) |
| East Asia | Male | 2016 | 1481.217627(1169.135916 to 1848.516725) | 1438.36119(1227.162617 to 1674.421119) | 22575.18871(19335.52289 to 26391.45443) |
| East Asia | Male | 2017 | 1502.694499(1185.183563 to 1877.543241) | 1394.424444(1172.090577 to 1646.278238) | 21914.25519(18383.1812 to 25921.8445) |
| East Asia | Male | 2018 | 1523.386175(1199.844885 to 1905.412388) | 1355.846167(1130.161254 to 1596.357758) | 21349.59528(17862.04833 to 25225.74209) |
| East Asia | Male | 2019 | 1534.268504(1207.543476 to 1923.543753) | 1337.596077(1100.578273 to 1610.186218) | 21039.98341(17341.21363 to 25368.85807) |
| East Asia | Male | 2020 | 1472.208483(1147.175699 to 1866.437866) | 1328.131307(1074.242473 to 1578.785808) | 20862.62978(16958.3668 to 25054.96219) |
| East Asia | Male | 2021 | 1476.037702(1149.668495 to 1877.575505) | 1313.341483(1068.420898 to 1580.691702) | 20630.65632(16797.99825 to 24836.79805) |
| Eastern Europe | Male | 1990 | 1558.770034(1151.592243 to 2081.22571) | 1690.380516(1608.097692 to 1741.584995) | 28002.91051(26847.49568 to 28861.16441) |
| Eastern Europe | Male | 1991 | 1528.948066(1139.466367 to 2023.014029) | 1713.076275(1629.301122 to 1766.626433) | 28294.94307(27118.43281 to 29163.25287) |
| Eastern Europe | Male | 1992 | 1501.157747(1123.300123 to 1974.33562) | 1792.847201(1707.510604 to 1849.002161) | 29626.36137(28434.59715 to 30536.91018) |
| Eastern Europe | Male | 1993 | 1477.781392(1111.91421 to 1932.542715) | 1991.895561(1901.179133 to 2050.581313) | 33213.36975(31958.82221 to 34162.72753) |
| Eastern Europe | Male | 1994 | 1460.676343(1105.579467 to 1899.490176) | 2100.819962(2010.793044 to 2160.145769) | 35436.15113(34168.61273 to 36386.78978) |
| Eastern Europe | Male | 1995 | 1451.689956(1102.628774 to 1882.66646) | 2048.716176(1964.397245 to 2107.678489) | 34576.72992(33371.47656 to 35528.54886) |
| Eastern Europe | Male | 1996 | 1449.08966(1101.050561 to 1876.255315) | 1951.48596(1869.00933 to 2007.276943) | 32917.26822(31751.66449 to 33832.58607) |
| Eastern Europe | Male | 1997 | 1448.056375(1100.200763 to 1874.329414) | 1894.903577(1811.05512 to 1950.103841) | 31773.4181(30595.22495 to 32677.11967) |
| Eastern Europe | Male | 1998 | 1448.037249(1099.781865 to 1872.635765) | 1874.604994(1790.826952 to 1929.374012) | 31263.12091(30087.25136 to 32164.18774) |
| Eastern Europe | Male | 1999 | 1446.798329(1097.589337 to 1872.843769) | 1936.720619(1854.217724 to 1991.539895) | 32535.39099(31395.42821 to 33414.01504) |
| Eastern Europe | Male | 2000 | 1442.208977(1092.686535 to 1867.736037) | 1983.617801(1900.562582 to 2039.964683) | 33475.87837(32320.37527 to 34381.95209) |
| Eastern Europe | Male | 2001 | 1432.864122(1085.49391 to 1855.83044) | 1990.339699(1906.261902 to 2046.350116) | 33687.56581(32525.09757 to 34608.56392) |
| Eastern Europe | Male | 2002 | 1418.885245(1075.752498 to 1837.196752) | 2005.992223(1922.354002 to 2060.940623) | 34084.22001(32922.16457 to 34995.21238) |
| Eastern Europe | Male | 2003 | 1401.807109(1061.414367 to 1816.472209) | 1969.402815(1887.122951 to 2022.389834) | 33698.79366(32553.66273 to 34605.61943) |
| Eastern Europe | Male | 2004 | 1383.482943(1047.035005 to 1792.83075) | 1858.288238(1777.852921 to 1910.380304) | 32161.63373(31021.06806 to 33055.04861) |
| Eastern Europe | Male | 2005 | 1363.248197(1030.568162 to 1766.217249) | 1819.584061(1740.150219 to 1871.585318) | 31873.17571(30716.71362 to 32789.10806) |
| Eastern Europe | Male | 2006 | 1331.697144(1008.664062 to 1721.035586) | 1647.937503(1573.981682 to 1697.12791) | 28862.78878(27785.14591 to 29713.79589) |
| Eastern Europe | Male | 2007 | 1286.053345(976.5011963 to 1656.091371) | 1549.738606(1478.452413 to 1597.785607) | 27169.8789(26136.56577 to 27989.64658) |
| Eastern Europe | Male | 2008 | 1236.218282(940.2920099 to 1587.660148) | 1518.492603(1448.577946 to 1566.578225) | 26709.91999(25688.27149 to 27525.27548) |
| Eastern Europe | Male | 2009 | 1193.404246(910.0493783 to 1526.617672) | 1402.008402(1335.636295 to 1446.437204) | 24702.41121(23731.83311 to 25449.61859) |
| Eastern Europe | Male | 2010 | 1166.119343(891.2511876 to 1493.664228) | 1369.124341(1304.827868 to 1413.941808) | 24171.53027(23237.72091 to 24927.30338) |
| Eastern Europe | Male | 2011 | 1148.678819(878.0478523 to 1466.41464) | 1247.54268(1185.980711 to 1288.991368) | 22050.6436(21146.44978 to 22748.00714) |
| Eastern Europe | Male | 2012 | 1130.928714(865.5499302 to 1441.279857) | 1185.721216(1125.573255 to 1225.347741) | 20927.31491(20055.86277 to 21620.1845) |
| Eastern Europe | Male | 2013 | 1116.032797(853.7086761 to 1421.671297) | 1148.811216(1089.036768 to 1187.717244) | 20229.16095(19365.44049 to 20900.61737) |
| Eastern Europe | Male | 2014 | 1106.630416(845.8596927 to 1410.595153) | 1133.556376(1074.043565 to 1172.281495) | 19961.24905(19092.49886 to 20643.48929) |
| Eastern Europe | Male | 2015 | 1104.014075(842.974756 to 1408.207262) | 1101.581765(1042.834337 to 1139.729244) | 19369.26086(18515.19031 to 20038.51612) |
| Eastern Europe | Male | 2016 | 1108.907053(846.4113233 to 1414.175852) | 1085.017585(1025.80797 to 1123.985326) | 19049.36102(18197.02914 to 19715.56585) |
| Eastern Europe | Male | 2017 | 1117.645432(852.6199567 to 1426.99518) | 1032.864658(968.6028995 to 1078.841976) | 18135.8181(17185.80304 to 18954.33932) |
| Eastern Europe | Male | 2018 | 1125.804916(858.5387233 to 1440.164093) | 1029.434618(959.178836 to 1084.263331) | 18044.46705(16962.12106 to 18950.91016) |
| Eastern Europe | Male | 2019 | 1128.443604(858.729603 to 1448.527059) | 1005.816208(934.3022375 to 1064.821386) | 17621.47746(16550.03079 to 18624.17218) |
| Eastern Europe | Male | 2020 | 1080.476353(810.9673116 to 1403.390629) | 982.2082568(907.8453121 to 1045.638649) | 17152.71705(15969.23242 to 18225.97975) |
| Eastern Europe | Male | 2021 | 1075.758041(810.4288225 to 1389.519402) | 943.7304107(835.7208263 to 1039.408299) | 16563.448(14825.79661 to 18230.48505) |
| Eastern Sub-Saharan Africa | Male | 1990 | 1179.537641(944.9471678 to 1458.166774) | 1288.501627(1097.483219 to 1470.441067) | 24108.80957(20812.72718 to 27293.58923) |
| Eastern Sub-Saharan Africa | Male | 1991 | 1175.562638(944.8017157 to 1447.353246) | 1289.691177(1102.60619 to 1469.850016) | 24118.86778(20857.95886 to 27250.04202) |
| Eastern Sub-Saharan Africa | Male | 1992 | 1171.244813(943.4975576 to 1436.463534) | 1288.608603(1109.076038 to 1468.42152) | 24088.96579(21009.9582 to 27219.55626) |
| Eastern Sub-Saharan Africa | Male | 1993 | 1166.829798(941.196212 to 1425.90927) | 1291.123033(1112.346722 to 1464.24475) | 24140.44676(21027.26019 to 27224.9544) |
| Eastern Sub-Saharan Africa | Male | 1994 | 1161.670895(938.3345332 to 1415.160319) | 1281.360498(1109.210775 to 1451.224686) | 23937.03435(20912.33282 to 26905.00901) |
| Eastern Sub-Saharan Africa | Male | 1995 | 1156.134331(934.5830111 to 1407.401824) | 1278.800652(1105.760758 to 1444.288427) | 23851.38201(20792.73538 to 26771.68902) |
| Eastern Sub-Saharan Africa | Male | 1996 | 1151.05097(931.051409 to 1399.473061) | 1269.916037(1099.201116 to 1435.853621) | 23655.41497(20627.14074 to 26607.09668) |
| Eastern Sub-Saharan Africa | Male | 1997 | 1145.721631(926.5116055 to 1393.923492) | 1258.407645(1093.784681 to 1419.170526) | 23428.00857(20486.63847 to 26284.8407) |
| Eastern Sub-Saharan Africa | Male | 1998 | 1139.596831(920.9270488 to 1388.568031) | 1247.165325(1086.756742 to 1406.036561) | 23207.12687(20359.14331 to 26007.03799) |
| Eastern Sub-Saharan Africa | Male | 1999 | 1132.710331(914.4669035 to 1382.688265) | 1225.066199(1072.06604 to 1381.942513) | 22768.38259(20017.65131 to 25547.20124) |
| Eastern Sub-Saharan Africa | Male | 2000 | 1125.169824(907.2558991 to 1374.019063) | 1207.028054(1058.923427 to 1358.194086) | 22392.01259(19775.4651 to 25047.33676) |
| Eastern Sub-Saharan Africa | Male | 2001 | 1116.544039(900.6292313 to 1363.914818) | 1180.374054(1039.569643 to 1328.385437) | 21841.71076(19325.43851 to 24399.83554) |
| Eastern Sub-Saharan Africa | Male | 2002 | 1106.771357(892.7782838 to 1351.561991) | 1160.54036(1025.778967 to 1303.4134) | 21444.99093(19092.77675 to 23892.87043) |
| Eastern Sub-Saharan Africa | Male | 2003 | 1096.232838(883.6066809 to 1339.280998) | 1142.327047(1012.453053 to 1280.820126) | 21118.20394(18814.42499 to 23544.03517) |
| Eastern Sub-Saharan Africa | Male | 2004 | 1085.265236(874.3827423 to 1326.583106) | 1129.362472(1002.888922 to 1262.074131) | 20871.49958(18689.79087 to 23139.63877) |
| Eastern Sub-Saharan Africa | Male | 2005 | 1074.14054(864.3448261 to 1313.431453) | 1112.661252(989.4426435 to 1243.745936) | 20542.79286(18392.33835 to 22752.53157) |
| Eastern Sub-Saharan Africa | Male | 2006 | 1061.512333(855.0348991 to 1296.816307) | 1099.548349(973.4748377 to 1229.753633) | 20273.25863(18063.28758 to 22527.3833) |
| Eastern Sub-Saharan Africa | Male | 2007 | 1046.8966(844.5337277 to 1276.406238) | 1079.854936(954.7317571 to 1207.365558) | 19908.72547(17721.6512 to 22101.2196) |
| Eastern Sub-Saharan Africa | Male | 2008 | 1031.836922(833.1351542 to 1256.199717) | 1067.553324(944.4323368 to 1197.07674) | 19660.88739(17535.86297 to 21859.67575) |
| Eastern Sub-Saharan Africa | Male | 2009 | 1018.115284(822.5891036 to 1238.143) | 1054.780218(931.6650739 to 1185.618779) | 19419.91809(17337.76705 to 21646.08397) |
| Eastern Sub-Saharan Africa | Male | 2010 | 1007.657709(814.6954953 to 1224.547056) | 1040.411018(913.0883944 to 1170.077975) | 19150.61861(16977.1133 to 21380.78994) |
| Eastern Sub-Saharan Africa | Male | 2011 | 998.5816736(807.6717735 to 1212.923983) | 1021.418076(897.420354 to 1147.39139) | 18801.90488(16705.92917 to 20976.28159) |
| Eastern Sub-Saharan Africa | Male | 2012 | 989.4478817(801.4979923 to 1200.712379) | 1000.742727(876.4193511 to 1126.785411) | 18447.91717(16287.05712 to 20580.98901) |
| Eastern Sub-Saharan Africa | Male | 2013 | 981.3855031(796.0467541 to 1189.562601) | 982.9873195(858.9061533 to 1108.700849) | 18131.46499(15992.45926 to 20282.80778) |
| Eastern Sub-Saharan Africa | Male | 2014 | 975.6402581(791.942013 to 1181.444333) | 967.5686323(842.4760352 to 1093.619067) | 17854.70776(15718.08118 to 20003.19054) |
| Eastern Sub-Saharan Africa | Male | 2015 | 973.5239731(789.8958146 to 1179.571165) | 957.6021003(829.2979218 to 1086.202126) | 17652.10768(15435.07319 to 19900.79663) |
| Eastern Sub-Saharan Africa | Male | 2016 | 974.5894539(791.4358055 to 1180.593675) | 948.0181756(821.5290533 to 1072.40577) | 17471.84551(15260.22397 to 19663.59825) |
| Eastern Sub-Saharan Africa | Male | 2017 | 976.809203(791.8406591 to 1183.579197) | 938.9492779(805.145584 to 1067.82257) | 17286.50127(14957.26516 to 19491.63121) |
| Eastern Sub-Saharan Africa | Male | 2018 | 978.9649403(792.5281836 to 1186.652207) | 931.7224966(797.9117732 to 1064.515443) | 17125.74388(14788.69486 to 19480.01045) |
| Eastern Sub-Saharan Africa | Male | 2019 | 980.2195273(791.5396558 to 1191.188715) | 926.2737017(793.7984587 to 1061.983989) | 16995.65853(14681.72164 to 19339.60189) |
| Eastern Sub-Saharan Africa | Male | 2020 | 966.8933224(775.0567202 to 1183.681971) | 924.6829704(790.039538 to 1070.348127) | 16963.70726(14646.72375 to 19456.6951) |
| Eastern Sub-Saharan Africa | Male | 2021 | 966.5386244(773.0924754 to 1181.287612) | 912.2681687(777.085237 to 1053.591236) | 16818.49299(14460.37634 to 19287.43) |
| Global | Male | 1990 | 1090.213147(856.670391 to 1364.373282) | 1059.197687(970.4118252 to 1133.117281) | 18508.4452(17046.17057 to 19830.36816) |
| Global | Male | 1991 | 1090.408812(861.2579437 to 1359.413082) | 1047.587829(961.7515449 to 1116.318202) | 18320.08984(16938.57961 to 19525.74117) |
| Global | Male | 1992 | 1089.536701(864.4078483 to 1355.095833) | 1042.672713(961.3031773 to 1115.12594) | 18261.355(16936.58017 to 19547.3403) |
| Global | Male | 1993 | 1087.586687(865.4693481 to 1349.877574) | 1045.217041(968.196363 to 1108.349287) | 18330.55616(17123.03686 to 19442.18332) |
| Global | Male | 1994 | 1084.666317(864.2256399 to 1343.156559) | 1042.069125(970.1212108 to 1100.797832) | 18301.14964(17175.77093 to 19347.7657) |
| Global | Male | 1995 | 1081.13747(860.912727 to 1337.499129) | 1038.508848(961.9600738 to 1096.630349) | 18226.79798(17002.51359 to 19277.88166) |
| Global | Male | 1996 | 1074.79604(856.6792505 to 1328.308324) | 1023.523954(952.9094791 to 1083.260475) | 17961.21774(16851.63783 to 18964.81979) |
| Global | Male | 1997 | 1064.349168(848.9944566 to 1314.552947) | 1008.259524(937.8509792 to 1061.182946) | 17692.88901(16604.68372 to 18599.98174) |
| Global | Male | 1998 | 1051.719065(839.2558505 to 1298.98343) | 998.512356(927.9707771 to 1053.329004) | 17515.39411(16461.37521 to 18458.95271) |
| Global | Male | 1999 | 1038.946522(829.1713635 to 1283.256443) | 988.2215287(920.0496686 to 1042.108526) | 17329.61379(16301.67329 to 18243.81188) |
| Global | Male | 2000 | 1027.746289(820.2635439 to 1269.373542) | 987.9703656(925.1564675 to 1040.201379) | 17315.16965(16338.04158 to 18239.76599) |
| Global | Male | 2001 | 1018.214529(812.4387591 to 1257.329018) | 986.2267662(920.1674015 to 1039.558055) | 17250.11905(16241.00135 to 18156.99821) |
| Global | Male | 2002 | 1009.03147(804.6286066 to 1246.104834) | 979.5116624(914.8940106 to 1030.683482) | 17132.44615(16131.56607 to 18026.25769) |
| Global | Male | 2003 | 999.5368769(796.5264902 to 1234.685346) | 983.8885373(920.8263153 to 1033.80304) | 17133.13804(16164.66001 to 17974.49784) |
| Global | Male | 2004 | 989.9282796(788.538706 to 1223.130441) | 968.7446754(904.0383129 to 1018.381091) | 16865.05318(15856.54572 to 17742.34148) |
| Global | Male | 2005 | 979.9902647(780.909103 to 1210.966119) | 942.8496543(881.5135667 to 989.894076) | 16429.51264(15510.64471 to 17253.99714) |
| Global | Male | 2006 | 968.1634105(771.8178941 to 1196.099185) | 889.4597633(829.6863879 to 936.5120001) | 15568.02445(14685.73749 to 16406.70133) |
| Global | Male | 2007 | 953.4149411(761.1024999 to 1177.112126) | 866.9299802(807.1542989 to 912.1045463) | 15159.97743(14260.15293 to 15954.79609) |
| Global | Male | 2008 | 937.3636099(748.7135369 to 1155.127127) | 858.4772858(798.7920863 to 907.6732772) | 15006.44314(14102.18068 to 15841.02833) |
| Global | Male | 2009 | 922.971822(737.4862068 to 1136.673668) | 846.8144466(787.4502603 to 893.497868) | 14793.44946(13879.81195 to 15595.83789) |
| Global | Male | 2010 | 911.4266271(728.5640605 to 1121.843778) | 837.0692317(774.1037333 to 884.4849036) | 14615.25482(13662.18024 to 15435.39139) |
| Global | Male | 2011 | 901.6366975(721.3935616 to 1108.282254) | 811.866457(752.1521891 to 864.8926266) | 14220.45319(13262.07708 to 15134.64597) |
| Global | Male | 2012 | 890.8644811(713.6024994 to 1093.124205) | 788.7601767(728.5810247 to 840.4539101) | 13841.37315(12888.91767 to 14746.33022) |
| Global | Male | 2013 | 881.1675069(706.5365227 to 1079.722194) | 776.4440217(712.8584487 to 832.23444) | 13558.43319(12569.03359 to 14530.68889) |
| Global | Male | 2014 | 875.267466(702.2565016 to 1071.650864) | 763.6362632(701.8040928 to 822.279999) | 13294.30446(12327.9729 to 14268.18595) |
| Global | Male | 2015 | 874.2168602(701.457876 to 1070.243685) | 749.6287195(683.9178708 to 805.6646091) | 13080.8683(12072.20714 to 14038.71667) |
| Global | Male | 2016 | 879.7600605(705.6988954 to 1077.229) | 741.1236093(670.8505833 to 803.5182879) | 12946.48095(11906.26554 to 13973.9318) |
| Global | Male | 2017 | 888.1251756(711.9922702 to 1088.375962) | 728.2258592(658.2260225 to 796.0184841) | 12736.48238(11615.77306 to 13869.40965) |
| Global | Male | 2018 | 895.8837478(717.7971535 to 1099.316458) | 717.8809595(646.9387109 to 783.6282884) | 12586.60402(11487.54155 to 13690.68068) |
| Global | Male | 2019 | 900.7177429(721.1437737 to 1106.396998) | 711.0347353(638.8953198 to 780.2676295) | 12469.31689(11299.50768 to 13636.74312) |
| Global | Male | 2020 | 875.4215868(694.7045905 to 1087.943013) | 706.7771332(628.5963564 to 775.5483019) | 12382.09232(11123.9442 to 13570.79791) |
| Global | Male | 2021 | 879.2319599(696.2634122 to 1093.140146) | 701.297769(625.77378 to 775.8006318) | 12285.55144(11059.77702 to 13521.39449) |
| High-income Asia Pacific | Male | 1990 | 1163.713467(864.1862933 to 1540.544411) | 838.2888583(766.4843979 to 885.88691) | 14171.68018(13117.83571 to 15019.16527) |
| High-income Asia Pacific | Male | 1991 | 1161.646261(867.5367204 to 1534.053467) | 792.8495818(726.193328 to 835.9783332) | 13487.88767(12484.77493 to 14311.3295) |
| High-income Asia Pacific | Male | 1992 | 1156.706246(868.6179377 to 1524.082349) | 755.1704256(691.3821217 to 796.168474) | 12915.18055(11960.27764 to 13706.21496) |
| High-income Asia Pacific | Male | 1993 | 1148.963961(866.6302401 to 1507.352331) | 728.0636669(666.6836143 to 767.0209761) | 12507.83587(11585.77987 to 13280.98342) |
| High-income Asia Pacific | Male | 1994 | 1139.478725(861.2012816 to 1487.085177) | 721.1215104(659.773641 to 759.7568782) | 12357.64262(11451.70976 to 13115.71003) |
| High-income Asia Pacific | Male | 1995 | 1128.545482(854.1847126 to 1467.149871) | 749.3335603(686.3691669 to 788.8363496) | 12697.77798(11790.84106 to 13458.90568) |
| High-income Asia Pacific | Male | 1996 | 1100.314343(836.8729031 to 1421.074498) | 712.7495994(651.3429648 to 751.3180349) | 12143.3817(11243.78786 to 12883.53115) |
| High-income Asia Pacific | Male | 1997 | 1048.259702(802.0506911 to 1346.019895) | 675.0119573(617.5168334 to 712.5987379) | 11574.40555(10703.26011 to 12305.15288) |
| High-income Asia Pacific | Male | 1998 | 986.3418323(759.0848278 to 1260.897865) | 652.8642009(597.1829634 to 689.3968969) | 11241.95463(10388.72689 to 11970.42256) |
| High-income Asia Pacific | Male | 1999 | 928.684913(717.3888819 to 1181.577029) | 627.3679307(574.3409445 to 663.3447231) | 10856.64133(10031.53498 to 11567.78963) |
| High-income Asia Pacific | Male | 2000 | 888.0502413(689.1236419 to 1123.351135) | 578.2486605(527.3877318 to 612.9600533) | 10128.31946(9329.904642 to 10819.70888) |
| High-income Asia Pacific | Male | 2001 | 862.425497(672.759957 to 1089.306851) | 544.8452849(495.2956074 to 578.5829335) | 9598.947596(8816.398914 to 10257.0362) |
| High-income Asia Pacific | Male | 2002 | 839.147238(657.1085432 to 1057.316346) | 518.5069882(470.2153028 to 551.3040753) | 9164.814094(8398.912833 to 9801.171906) |
| High-income Asia Pacific | Male | 2003 | 816.5719052(641.2176161 to 1025.795864) | 503.7067217(455.958738 to 535.7748065) | 8908.514444(8162.910443 to 9534.998611) |
| High-income Asia Pacific | Male | 2004 | 793.9769657(625.1304402 to 996.3649255) | 481.5999367(434.804272 to 512.7325348) | 8533.752962(7811.750105 to 9142.02707) |
| High-income Asia Pacific | Male | 2005 | 770.686747(606.9516012 to 966.9019506) | 466.0751547(420.4663444 to 496.6087797) | 8280.33329(7579.576054 to 8877.499591) |
| High-income Asia Pacific | Male | 2006 | 744.419077(587.9848565 to 931.4088164) | 438.6263609(394.8991951 to 468.2937051) | 7855.752369(7175.185524 to 8433.009755) |
| High-income Asia Pacific | Male | 2007 | 714.1188369(564.0612866 to 891.5112215) | 417.5335692(374.5623818 to 445.8814272) | 7505.488564(6847.75243 to 8074.276982) |
| High-income Asia Pacific | Male | 2008 | 683.5095726(538.9215794 to 852.4434937) | 401.0600621(359.0381831 to 428.3307387) | 7218.41948(6588.289286 to 7765.116175) |
| High-income Asia Pacific | Male | 2009 | 655.9489577(517.1558127 to 817.8831405) | 384.3832226(343.5536468 to 410.7866428) | 6944.136417(6325.672394 to 7478.802858) |
| High-income Asia Pacific | Male | 2010 | 635.1545957(501.1214093 to 791.369063) | 373.9601022(334.4499969 to 399.7553343) | 6775.188048(6159.92326 to 7297.873094) |
| High-income Asia Pacific | Male | 2011 | 619.0258708(489.0156111 to 769.8485671) | 363.4087333(324.950318 to 388.5609539) | 6603.018967(5997.838714 to 7115.547526) |
| High-income Asia Pacific | Male | 2012 | 603.9796203(477.6163494 to 749.8006503) | 347.9891476(310.1240125 to 372.5369237) | 6355.161863(5765.11296 to 6863.751638) |
| High-income Asia Pacific | Male | 2013 | 590.87768(468.026597 to 733.1476894) | 330.0270926(293.5401661 to 353.6152668) | 6078.126213(5507.423383 to 6584.527818) |
| High-income Asia Pacific | Male | 2014 | 581.1998272(460.9686143 to 720.5145577) | 313.0314312(277.8569475 to 335.6770752) | 5826.499637(5263.326484 to 6327.835968) |
| High-income Asia Pacific | Male | 2015 | 576.0578488(457.066032 to 714.8030711) | 299.9437172(265.6282218 to 322.324875) | 5630.468573(5077.027498 to 6119.795453) |
| High-income Asia Pacific | Male | 2016 | 577.001739(458.1440761 to 715.329961) | 290.2667283(256.9145672 to 312.7226362) | 5486.693003(4934.629426 to 5979.36933) |
| High-income Asia Pacific | Male | 2017 | 582.2889905(462.3736107 to 721.7776933) | 281.5614707(248.8777926 to 303.6546486) | 5356.856218(4813.284002 to 5852.201324) |
| High-income Asia Pacific | Male | 2018 | 586.6693367(464.4060239 to 727.1965758) | 274.8159768(242.2405529 to 296.5675494) | 5255.367141(4708.126387 to 5745.340298) |
| High-income Asia Pacific | Male | 2019 | 585.4179036(462.548081 to 727.8147923) | 268.7957926(236.4641418 to 290.4999153) | 5164.288682(4611.661024 to 5650.20321) |
| High-income Asia Pacific | Male | 2020 | 563.1731461(441.5641997 to 706.4873452) | 261.5848508(228.4717406 to 283.319447) | 5056.367984(4498.352852 to 5539.781965) |
| High-income Asia Pacific | Male | 2021 | 562.5747901(441.4300744 to 703.7969302) | 264.1086073(231.3797608 to 286.0427055) | 5079.474804(4516.35158 to 5573.282722) |
| High-income North America | Male | 1990 | 720.6908074(523.3696348 to 962.5915958) | 339.4022484(308.6472515 to 357.39514) | 5961.742897(5521.664859 to 6347.839235) |
| High-income North America | Male | 1991 | 691.2730777(507.8137882 to 917.7626828) | 330.9979356(300.9240019 to 348.524723) | 5841.573008(5402.095569 to 6220.606601) |
| High-income North America | Male | 1992 | 664.0702586(491.480369 to 875.9851776) | 325.9881488(295.844501 to 343.1843063) | 5754.996412(5315.269257 to 6133.136538) |
| High-income North America | Male | 1993 | 639.9818216(477.0330679 to 839.0154417) | 331.7086577(301.356945 to 349.6300953) | 5833.050247(5385.38036 to 6213.294415) |
| High-income North America | Male | 1994 | 619.9626578(465.894402 to 808.1751816) | 333.7646265(303.078707 to 351.8286981) | 5857.778397(5404.136229 to 6241.384125) |
| High-income North America | Male | 1995 | 604.8907153(456.5415688 to 785.7014729) | 334.9685613(304.0183953 to 353.3472913) | 5873.190295(5418.444731 to 6260.056694) |
| High-income North America | Male | 1996 | 593.9311152(449.0291231 to 770.0473958) | 330.1931705(299.3999482 to 348.591497) | 5802.902689(5349.308774 to 6190.280854) |
| High-income North America | Male | 1997 | 584.8699017(442.6572857 to 757.7948863) | 324.3155012(294.0758023 to 342.5505384) | 5714.364702(5260.427648 to 6104.457608) |
| High-income North America | Male | 1998 | 576.6563071(436.8648473 to 746.7474884) | 319.1388649(289.2615596 to 337.310971) | 5640.562338(5183.032032 to 6032.24416) |
| High-income North America | Male | 1999 | 568.2843375(430.6315117 to 735.3558386) | 325.4420339(294.6496515 to 344.0011138) | 5711.089274(5252.948084 to 6108.212588) |
| High-income North America | Male | 2000 | 558.6829771(423.6886374 to 722.3451399) | 319.1555283(288.6203181 to 337.5531474) | 5620.837533(5168.037789 to 6017.867297) |
| High-income North America | Male | 2001 | 546.7827039(415.3263177 to 706.0216061) | 309.6530692(279.6414273 to 327.6449751) | 5471.419323(5020.435541 to 5867.242205) |
| High-income North America | Male | 2002 | 532.910389(405.8695045 to 686.9468291) | 301.5948663(271.940951 to 319.1716134) | 5337.508913(4892.59233 to 5727.014764) |
| High-income North America | Male | 2003 | 518.2135537(395.2346296 to 666.9184134) | 288.4426226(259.7887166 to 305.3834807) | 5137.25345(4703.544337 to 5518.956778) |
| High-income North America | Male | 2004 | 503.9372688(384.7105983 to 647.8418464) | 269.6857644(241.9976013 to 285.8262723) | 4866.835476(4442.452934 to 5244.875869) |
| High-income North America | Male | 2005 | 491.2180127(375.8983846 to 630.498984) | 256.6137342(230.2482088 to 272.1177428) | 4681.612051(4271.14792 to 5045.718441) |
| High-income North America | Male | 2006 | 478.5965553(368.0749418 to 612.33984) | 244.3626091(218.8478146 to 259.2300055) | 4501.304982(4099.620434 to 4854.801413) |
| High-income North America | Male | 2007 | 465.0020938(359.0799952 to 592.5685738) | 235.4287185(210.3933109 to 249.9099227) | 4352.529427(3956.937173 to 4696.459693) |
| High-income North America | Male | 2008 | 452.4017314(350.8392042 to 573.9986994) | 227.97538(203.1082611 to 241.9928647) | 4220.067325(3830.65788 to 4549.509186) |
| High-income North America | Male | 2009 | 442.8864464(344.8160379 to 560.1167202) | 219.7703126(195.2250638 to 233.5214025) | 4088.585829(3706.944086 to 4416.423025) |
| High-income North America | Male | 2010 | 438.3629175(342.0273101 to 553.4830259) | 213.7503356(189.4685402 to 227.500277) | 3987.950715(3611.575259 to 4309.341718) |
| High-income North America | Male | 2011 | 437.4083023(341.6979808 to 552.1426881) | 210.6474133(186.4375489 to 224.3509157) | 3934.990029(3562.60982 to 4252.443776) |
| High-income North America | Male | 2012 | 436.8440512(341.372495 to 550.7459723) | 208.1656342(183.8147576 to 222.1254575) | 3888.115357(3519.50643 to 4202.236758) |
| High-income North America | Male | 2013 | 436.4291501(341.3908284 to 550.036633) | 206.3918717(182.0152339 to 220.1973914) | 3861.978365(3498.854759 to 4179.800464) |
| High-income North America | Male | 2014 | 435.9292067(340.9419852 to 549.3443753) | 207.6500363(183.22023 to 221.5943173) | 3870.511208(3505.961927 to 4189.44341) |
| High-income North America | Male | 2015 | 434.997895(340.0411769 to 549.0651127) | 209.4462051(184.6322384 to 223.658399) | 3894.45549(3525.94779 to 4215.123924) |
| High-income North America | Male | 2016 | 433.2567103(338.5262805 to 547.4916349) | 211.1922301(186.2897533 to 225.5764193) | 3924.934062(3552.720176 to 4242.466165) |
| High-income North America | Male | 2017 | 430.8099371(336.1902619 to 545.2622359) | 212.3646786(187.0115561 to 226.92722) | 3934.357798(3558.815656 to 4253.957492) |
| High-income North America | Male | 2018 | 428.2235491(333.2377633 to 543.1079596) | 209.2153144(184.035592 to 223.6527523) | 3892.204779(3519.412438 to 4212.612357) |
| High-income North America | Male | 2019 | 426.0842648(330.3535673 to 542.2536103) | 207.8915988(182.812062 to 222.3477185) | 3868.436252(3495.832064 to 4186.630157) |
| High-income North America | Male | 2020 | 425.8515205(326.7925387 to 544.7115477) | 203.5925358(178.1341335 to 218.3927807) | 3793.655752(3417.073585 to 4105.307068) |
| High-income North America | Male | 2021 | 422.4855231(322.3637175 to 544.4684786) | 207.6753555(181.9068415 to 223.1158413) | 3864.910287(3487.619965 to 4186.507722) |
| North Africa and Middle East | Male | 1990 | 951.4451846(752.6009851 to 1191.921337) | 1139.132838(965.84214 to 1281.827861) | 19288.90177(16530.31801 to 21666.91605) |
| North Africa and Middle East | Male | 1991 | 946.8813047(753.3923868 to 1180.885915) | 1127.608087(961.0149345 to 1266.461663) | 19096.40378(16468.82628 to 21391.75354) |
| North Africa and Middle East | Male | 1992 | 942.2000183(753.044456 to 1167.763008) | 1113.1811(952.4001656 to 1250.336559) | 18846.11466(16329.53891 to 21092.44957) |
| North Africa and Middle East | Male | 1993 | 937.7550429(752.4482062 to 1159.038086) | 1109.940141(951.6220629 to 1247.478545) | 18775.51467(16271.57764 to 21013.90319) |
| North Africa and Middle East | Male | 1994 | 933.6643854(750.4870205 to 1152.366625) | 1106.682228(952.8555121 to 1245.337554) | 18681.7135(16270.4259 to 20940.23909) |
| North Africa and Middle East | Male | 1995 | 930.0827283(746.8996638 to 1145.70362) | 1091.174036(939.7591687 to 1228.29439) | 18382.04427(16026.02405 to 20621.59391) |
| North Africa and Middle East | Male | 1996 | 926.4160765(743.9492579 to 1141.69522) | 1067.266172(921.7492002 to 1204.52489) | 17968.62936(15710.86382 to 20161.46425) |
| North Africa and Middle East | Male | 1997 | 921.8802546(739.6148558 to 1136.24335) | 1049.738684(908.2571835 to 1185.248392) | 17677.02755(15472.98626 to 19852.46721) |
| North Africa and Middle East | Male | 1998 | 916.6749403(734.2548943 to 1131.765305) | 1033.808029(895.1158802 to 1163.243086) | 17362.91862(15246.77832 to 19473.76122) |
| North Africa and Middle East | Male | 1999 | 910.6614003(728.0820996 to 1125.358425) | 1012.338733(877.3011484 to 1139.53136) | 17022.63678(14937.59779 to 19055.44652) |
| North Africa and Middle East | Male | 2000 | 903.6606358(720.6516537 to 1118.841756) | 971.6643829(838.4385094 to 1096.433135) | 16416.26637(14390.93656 to 18431.82883) |
| North Africa and Middle East | Male | 2001 | 895.4024515(712.5072895 to 1107.798258) | 957.3780402(832.8255318 to 1075.62512) | 16169.79889(14254.27878 to 18064.13993) |
| North Africa and Middle East | Male | 2002 | 886.2843934(703.7006761 to 1096.548119) | 940.5496517(820.9488866 to 1055.333841) | 15833.91959(13988.3492 to 17659.76552) |
| North Africa and Middle East | Male | 2003 | 876.8930565(696.2164156 to 1085.613627) | 926.1747101(805.9707777 to 1037.613298) | 15546.01135(13694.72994 to 17316.77195) |
| North Africa and Middle East | Male | 2004 | 867.6437303(689.2632446 to 1075.761211) | 903.7643574(786.2329329 to 1011.373148) | 15128.41303(13326.71035 to 16831.55194) |
| North Africa and Middle East | Male | 2005 | 858.9671172(680.61223 to 1066.149179) | 880.3424988(764.5008233 to 985.8923687) | 14713.98682(12914.31566 to 16411.33345) |
| North Africa and Middle East | Male | 2006 | 849.2246851(673.8867408 to 1053.164877) | 862.8163456(750.7515953 to 967.1829509) | 14414.29335(12665.17504 to 16107.01106) |
| North Africa and Middle East | Male | 2007 | 837.4924759(666.051943 to 1035.613959) | 842.6923651(729.6158748 to 946.6155038) | 14068.20276(12305.70856 to 15722.38003) |
| North Africa and Middle East | Male | 2008 | 825.2592445(656.5905025 to 1018.906759) | 827.8936375(717.0892797 to 934.102364) | 13812.744(12083.75634 to 15512.06575) |
| North Africa and Middle East | Male | 2009 | 814.2454755(648.4713944 to 1004.958776) | 815.5314491(707.1061642 to 921.4057075) | 13608.10535(11917.69323 to 15296.84916) |
| North Africa and Middle East | Male | 2010 | 806.057088(642.1607979 to 994.7638414) | 797.132835(691.5974102 to 900.4615501) | 13298.81265(11643.49851 to 14957.96344) |
| North Africa and Middle East | Male | 2011 | 799.3461899(637.6472252 to 984.4879261) | 783.3197616(679.0625415 to 885.3452261) | 13082.9309(11480.36958 to 14724.83363) |
| North Africa and Middle East | Male | 2012 | 792.1134317(632.4670501 to 973.4125653) | 770.0272027(668.486667 to 868.0334368) | 12898.5139(11346.84113 to 14496.27968) |
| North Africa and Middle East | Male | 2013 | 785.3691822(627.2446568 to 961.7637841) | 760.0677661(661.0029435 to 856.5980729) | 12744.28084(11188.30754 to 14312.49228) |
| North Africa and Middle East | Male | 2014 | 780.313729(623.9587016 to 955.4549607) | 751.7220604(652.4887249 to 847.8491008) | 12620.33742(11084.17841 to 14193.54616) |
| North Africa and Middle East | Male | 2015 | 778.0796939(621.201097 to 951.6412744) | 747.0261626(651.653577 to 842.0465963) | 12535.51422(11044.61153 to 14066.52493) |
| North Africa and Middle East | Male | 2016 | 778.2483684(623.4465688 to 950.6776135) | 733.7497925(639.7645377 to 827.5784924) | 12305.18011(10849.62243 to 13806.38255) |
| North Africa and Middle East | Male | 2017 | 778.9751926(625.9150268 to 951.2273134) | 717.3576303(624.9065397 to 809.9030857) | 12015.65288(10574.76607 to 13487.99294) |
| North Africa and Middle East | Male | 2018 | 779.1950197(624.8513715 to 953.6380728) | 707.1162011(612.3318568 to 798.3808984) | 11841.08302(10399.06416 to 13297.79969) |
| North Africa and Middle East | Male | 2019 | 777.9168947(623.1379594 to 952.2417642) | 706.4298927(612.3691506 to 798.4051748) | 11799.58417(10347.04001 to 13267.14368) |
| North Africa and Middle East | Male | 2020 | 758.9396242(606.5255831 to 941.9731653) | 706.8399841(607.6348871 to 805.7593463) | 11797.30914(10221.69929 to 13385.59338) |
| North Africa and Middle East | Male | 2021 | 754.491033(601.7385919 to 933.6438121) | 696.1872438(593.1352972 to 802.8511093) | 11630.07294(10018.12802 to 13337.674) |
| Oceania | Male | 1990 | 1167.717331(942.9527242 to 1423.313857) | 1313.322631(1001.309044 to 1648.570789) | 24063.29989(18490.69367 to 30163.88741) |
| Oceania | Male | 1991 | 1170.847985(950.8290602 to 1414.859461) | 1308.975724(1000.035032 to 1640.277425) | 23963.71907(18445.44437 to 29913.90022) |
| Oceania | Male | 1992 | 1172.560299(956.1323712 to 1410.995273) | 1299.61752(989.7465772 to 1632.09983) | 23760.22139(18240.20319 to 29780.9661) |
| Oceania | Male | 1993 | 1172.983628(959.04775 to 1407.75515) | 1288.471165(990.1917162 to 1620.551049) | 23544.65804(18268.33841 to 29602.48744) |
| Oceania | Male | 1994 | 1172.260114(960.1155104 to 1407.61691) | 1271.265862(978.4008374 to 1598.052491) | 23226.49378(18027.79053 to 29138.83371) |
| Oceania | Male | 1995 | 1170.538121(959.3217039 to 1408.817827) | 1253.887846(966.5171114 to 1572.279553) | 22904.62473(17805.1168 to 28674.1383) |
| Oceania | Male | 1996 | 1166.604017(956.1856315 to 1398.807248) | 1240.388594(954.9244472 to 1560.054921) | 22683.11901(17664.15897 to 28370.53323) |
| Oceania | Male | 1997 | 1159.857574(952.6241266 to 1387.469462) | 1223.354077(942.5531297 to 1536.440434) | 22385.46805(17513.98672 to 27857.25133) |
| Oceania | Male | 1998 | 1151.584174(943.7081264 to 1379.073564) | 1206.026765(930.2626232 to 1519.118238) | 22047.42051(17164.13638 to 27585.13757) |
| Oceania | Male | 1999 | 1142.714038(933.8481604 to 1370.145446) | 1202.451711(930.3319003 to 1508.179468) | 21948.16869(17126.99675 to 27331.74458) |
| Oceania | Male | 2000 | 1134.29577(925.7958712 to 1362.686184) | 1194.68594(922.3219739 to 1498.854794) | 21768.41192(17023.08616 to 27095.65461) |
| Oceania | Male | 2001 | 1125.912424(922.4658408 to 1350.31131) | 1179.059463(909.9438673 to 1482.719549) | 21483.37649(16742.09374 to 26705.07483) |
| Oceania | Male | 2002 | 1115.890127(917.1039648 to 1339.652557) | 1163.943656(898.4167644 to 1461.080003) | 21167.62179(16554.84846 to 26192.21866) |
| Oceania | Male | 2003 | 1104.986525(909.579567 to 1328.29005) | 1150.590784(890.5412343 to 1438.465523) | 20887.81659(16410.85668 to 25746.17169) |
| Oceania | Male | 2004 | 1093.734117(900.3447414 to 1312.780073) | 1128.476303(872.2256433 to 1411.082159) | 20472.58842(16067.6875 to 25257.70697) |
| Oceania | Male | 2005 | 1082.607054(889.9212063 to 1300.466477) | 1117.609169(864.464654 to 1396.106813) | 20326.15379(15927.81817 to 25141.60991) |
| Oceania | Male | 2006 | 1069.828335(881.7852307 to 1282.047039) | 1113.610741(862.3678272 to 1391.287238) | 20269.96124(15904.04155 to 25033.92143) |
| Oceania | Male | 2007 | 1053.568111(870.3772155 to 1262.080579) | 1095.930337(852.6192446 to 1371.085551) | 19971.3476(15799.92737 to 24636.90668) |
| Oceania | Male | 2008 | 1036.570433(855.9039185 to 1241.573813) | 1084.385392(837.376031 to 1361.000191) | 19777.78051(15565.98241 to 24525.25512) |
| Oceania | Male | 2009 | 1021.253976(842.3595983 to 1226.481828) | 1068.754192(824.6298663 to 1341.742371) | 19515.03151(15402.3291 to 24145.73188) |
| Oceania | Male | 2010 | 1009.91246(831.4512619 to 1216.752453) | 1065.665566(820.9955038 to 1337.481386) | 19498.39031(15348.47139 to 24109.46877) |
| Oceania | Male | 2011 | 1001.487648(827.007806 to 1200.224763) | 1070.883502(818.5170239 to 1346.733512) | 19594.05279(15322.81423 to 24248.66072) |
| Oceania | Male | 2012 | 992.9373896(821.978506 to 1183.928425) | 1064.344281(818.5107292 to 1345.742672) | 19501.84438(15304.91719 to 24304.5262) |
| Oceania | Male | 2013 | 985.5499815(816.6144046 to 1173.696506) | 1057.617846(813.2452756 to 1330.336062) | 19359.10239(15226.91756 to 24064.72704) |
| Oceania | Male | 2014 | 980.2450073(811.7162837 to 1169.031755) | 1051.049453(806.193648 to 1327.787415) | 19236.22373(15092.99141 to 23986.72352) |
| Oceania | Male | 2015 | 977.8769775(807.0060369 to 1167.551739) | 1041.753177(796.2298631 to 1324.449014) | 19082.13913(14846.65864 to 23972.47821) |
| Oceania | Male | 2016 | 979.5472279(811.3477543 to 1167.765145) | 1034.833839(790.6833734 to 1311.202321) | 18891.27975(14689.87065 to 23653.62795) |
| Oceania | Male | 2017 | 982.9252984(815.4298249 to 1171.56448) | 1034.988073(786.0895597 to 1307.796685) | 18878.84236(14630.7814 to 23603.02201) |
| Oceania | Male | 2018 | 986.1396727(816.1283348 to 1174.461595) | 1033.751272(782.6248962 to 1307.640207) | 18845.29419(14514.13122 to 23575.7731) |
| Oceania | Male | 2019 | 987.0333455(811.4693103 to 1182.048032) | 1031.919109(781.8357943 to 1315.051087) | 18851.65026(14476.57842 to 23810.38146) |
| Oceania | Male | 2020 | 966.4854669(791.4313382 to 1167.046312) | 1026.713257(774.6100616 to 1315.99541) | 18773.65953(14388.19882 to 23817.59562) |
| Oceania | Male | 2021 | 971.5334231(795.6005874 to 1173.320325) | 1012.067165(762.5849251 to 1305.157061) | 18526.30541(14186.0445 to 23599.59373) |
| South Asia | Male | 1990 | 828.9691983(644.7175646 to 1047.606237) | 732.3130272(612.010547 to 843.5006075) | 13772.40925(11674.97215 to 15703.46235) |
| South Asia | Male | 1991 | 828.1050582(647.063474 to 1042.104068) | 725.3146234(609.9089761 to 831.319131) | 13628.86179(11608.00419 to 15460.27968) |
| South Asia | Male | 1992 | 826.7838896(648.4387859 to 1038.03307) | 727.9819125(614.5133245 to 836.6812514) | 13673.85814(11686.49812 to 15556.74459) |
| South Asia | Male | 1993 | 824.8955366(647.8216504 to 1033.5386) | 725.4688034(614.4457026 to 828.8516088) | 13618.26386(11706.63932 to 15402.39405) |
| South Asia | Male | 1994 | 822.3962703(646.5233796 to 1027.838291) | 729.6179492(617.7117365 to 832.2746393) | 13710.54574(11798.8433 to 15509.12225) |
| South Asia | Male | 1995 | 819.1902365(643.7344286 to 1022.18824) | 738.7027935(632.636137 to 837.3553138) | 13844.30612(12049.59284 to 15517.79925) |
| South Asia | Male | 1996 | 814.3669868(639.884988 to 1015.551905) | 737.305208(635.0570743 to 834.7848427) | 13781.05016(12029.19366 to 15439.22123) |
| South Asia | Male | 1997 | 807.6570459(634.5742513 to 1007.809327) | 738.3757778(639.7841591 to 838.1124941) | 13782.25885(12086.16156 to 15484.59819) |
| South Asia | Male | 1998 | 799.9510959(628.6017961 to 999.1031385) | 732.6564047(635.086967 to 823.9336872) | 13669.6767(12045.68716 to 15227.84448) |
| South Asia | Male | 1999 | 791.8081848(621.9201233 to 989.6100568) | 689.786102(599.183605 to 783.7855897) | 12860.59654(11302.24692 to 14454.66092) |
| South Asia | Male | 2000 | 783.8347829(615.013674 to 979.8309565) | 683.1582297(598.122895 to 771.3623192) | 12720.65221(11267.29765 to 14226.93374) |
| South Asia | Male | 2001 | 775.7757384(608.5246741 to 969.7118929) | 688.2389619(605.4849377 to 770.6867187) | 12779.60812(11389.11996 to 14169.61492) |
| South Asia | Male | 2002 | 766.8535852(600.9022371 to 958.6878401) | 677.90948(597.4264033 to 758.6514885) | 12597.67727(11223.12166 to 13988.03404) |
| South Asia | Male | 2003 | 757.5322688(592.9067878 to 947.3722225) | 686.6796924(606.0056945 to 765.9222132) | 12707.50357(11347.56767 to 14062.48748) |
| South Asia | Male | 2004 | 748.2015961(585.1129405 to 936.2730369) | 683.1608635(601.120683 to 760.9517064) | 12573.72435(11179.44562 to 13921.00771) |
| South Asia | Male | 2005 | 738.7930032(577.4824521 to 925.6338348) | 666.9972831(590.6233341 to 739.8883295) | 12291.4672(10991.77587 to 13542.00715) |
| South Asia | Male | 2006 | 727.2065083(569.1661436 to 910.1025833) | 658.0129474(582.7970124 to 731.1264176) | 12100.16956(10817.8594 to 13358.27225) |
| South Asia | Male | 2007 | 712.7377383(558.8136234 to 892.09233) | 668.1694162(595.437999 to 743.5685795) | 12268.29645(11037.55066 to 13564.52912) |
| South Asia | Male | 2008 | 697.3299553(547.5845678 to 872.9166002) | 665.3225338(595.6054507 to 737.2594529) | 12260.62564(11073.05815 to 13499.68381) |
| South Asia | Male | 2009 | 682.8766306(536.393638 to 854.4248103) | 641.4481656(571.0935407 to 711.8789491) | 11861.60102(10671.2495 to 13078.49409) |
| South Asia | Male | 2010 | 671.3869583(527.6041614 to 838.628634) | 639.9429662(570.1693071 to 707.504104) | 11813.10531(10631.31664 to 12961.12512) |
| South Asia | Male | 2011 | 660.8464715(520.7464368 to 824.3754888) | 620.7105124(554.2400338 to 690.8111413) | 11571.27448(10438.58698 to 12766.88626) |
| South Asia | Male | 2012 | 649.4026161(513.4944666 to 807.9634758) | 589.655676(525.7743075 to 655.1528614) | 11039.41179(9961.013391 to 12137.21836) |
| South Asia | Male | 2013 | 639.2284849(506.4613582 to 794.1092464) | 619.0466384(556.3299951 to 688.4970625) | 11138.4028(10070.91408 to 12328.19419) |
| South Asia | Male | 2014 | 632.7213797(502.3906051 to 785.1692963) | 650.1852394(579.0180136 to 724.3791436) | 11302.06128(10114.6811 to 12532.82235) |
| South Asia | Male | 2015 | 631.8324897(502.2939337 to 783.2311947) | 625.7632115(560.0316917 to 699.79437) | 11075.61264(9983.268806 to 12285.51748) |
| South Asia | Male | 2016 | 637.6025727(506.7282409 to 790.582195) | 608.9573829(543.0235766 to 682.9069714) | 10920.86852(9847.774205 to 12147.55939) |
| South Asia | Male | 2017 | 647.177298(513.824632 to 802.9804891) | 614.1033236(546.6692937 to 682.8290713) | 11067.05938(9976.025897 to 12197.35386) |
| South Asia | Male | 2018 | 656.6334649(521.0308164 to 814.3478016) | 616.4222714(547.2092866 to 688.2893487) | 11208.41313(10065.61714 to 12418.80382) |
| South Asia | Male | 2019 | 662.2829133(524.5416033 to 821.0804181) | 616.7943607(548.1010944 to 689.575224) | 11214.83412(10050.23503 to 12441.78039) |
| South Asia | Male | 2020 | 645.0813291(506.0811466 to 805.7183382) | 614.8431951(542.7692815 to 692.5535462) | 11132.76197(9930.723867 to 12487.00461) |
| South Asia | Male | 2021 | 647.9443005(508.7475713 to 810.8865973) | 609.0925395(529.885291 to 695.0087022) | 10990.97402(9640.681161 to 12508.37325) |
| Southeast Asia | Male | 1990 | 1231.957861(982.3923272 to 1519.187946) | 1318.611475(1126.204489 to 1505.077429) | 24107.13417(20846.74071 to 27428.43438) |
| Southeast Asia | Male | 1991 | 1233.706942(987.8764987 to 1517.376241) | 1309.863905(1133.737635 to 1481.96105) | 23981.50574(21005.6324 to 26962.47021) |
| Southeast Asia | Male | 1992 | 1235.228059(991.7982623 to 1517.619564) | 1312.289205(1145.493812 to 1477.459196) | 24036.79211(21185.65117 to 26914.7692) |
| Southeast Asia | Male | 1993 | 1236.349282(995.345031 to 1516.846349) | 1308.099465(1147.498487 to 1463.632068) | 23976.94362(21252.23242 to 26635.92688) |
| Southeast Asia | Male | 1994 | 1237.166832(997.1944374 to 1516.721986) | 1307.499371(1157.308338 to 1460.565354) | 23968.56188(21384.25645 to 26615.58955) |
| Southeast Asia | Male | 1995 | 1237.536642(997.670998 to 1516.504639) | 1302.433633(1153.548724 to 1442.253358) | 23866.61748(21371.2589 to 26284.02865) |
| Southeast Asia | Male | 1996 | 1237.890271(999.7963401 to 1516.966394) | 1295.645022(1149.780855 to 1435.328126) | 23713.96018(21274.6885 to 26165.92402) |
| Southeast Asia | Male | 1997 | 1238.481315(1000.072858 to 1517.735703) | 1286.81308(1145.772159 to 1422.334474) | 23524.32465(21154.74128 to 25869.7883) |
| Southeast Asia | Male | 1998 | 1239.016813(998.9543823 to 1517.884086) | 1293.045113(1154.726238 to 1423.292) | 23597.92304(21243.97554 to 25868.01935) |
| Southeast Asia | Male | 1999 | 1239.269722(997.5126108 to 1516.355151) | 1295.303145(1160.90381 to 1425.789114) | 23630.88377(21374.87708 to 25899.86977) |
| Southeast Asia | Male | 2000 | 1238.856481(996.7758756 to 1515.521731) | 1296.655085(1165.43495 to 1423.948731) | 23617.691(21396.74146 to 25807.14922) |
| Southeast Asia | Male | 2001 | 1237.323088(996.7549041 to 1513.398699) | 1299.374263(1166.752617 to 1428.588908) | 23604.05509(21389.98612 to 25895.24639) |
| Southeast Asia | Male | 2002 | 1234.788822(994.165518 to 1509.239793) | 1317.086381(1187.362666 to 1442.349371) | 23875.22638(21675.17091 to 26022.50285) |
| Southeast Asia | Male | 2003 | 1231.161414(992.1809965 to 1504.27583) | 1326.257901(1197.46506 to 1451.772014) | 23999.09087(21882.71115 to 26168.14333) |
| Southeast Asia | Male | 2004 | 1226.386(989.3311036 to 1497.454231) | 1335.834042(1214.602946 to 1466.109561) | 24142.51185(22099.28619 to 26465.35431) |
| Southeast Asia | Male | 2005 | 1220.5355(985.5490014 to 1489.993011) | 1344.649945(1222.877783 to 1468.296972) | 24235.27287(22204.97625 to 26337.04416) |
| Southeast Asia | Male | 2006 | 1213.518289(979.620827 to 1477.374966) | 1338.349017(1213.715311 to 1460.953533) | 24070.23038(21968.27684 to 26229.95418) |
| Southeast Asia | Male | 2007 | 1205.13501(972.857238 to 1463.913126) | 1332.019454(1204.581222 to 1455.832812) | 23953.51683(21849.83873 to 26147.04313) |
| Southeast Asia | Male | 2008 | 1195.587449(967.9051177 to 1450.639081) | 1333.131374(1208.902018 to 1453.000725) | 23954.46521(21869.82663 to 26061.05698) |
| Southeast Asia | Male | 2009 | 1185.893898(961.8778391 to 1436.729399) | 1326.477658(1204.247284 to 1454.917217) | 23840.5231(21784.5792 to 26066.16524) |
| Southeast Asia | Male | 2010 | 1176.385122(954.5365756 to 1425.776772) | 1317.484006(1194.88065 to 1443.503113) | 23671.71524(21629.33974 to 25873.63155) |
| Southeast Asia | Male | 2011 | 1167.425399(947.9421945 to 1413.804895) | 1299.984268(1174.324676 to 1427.299702) | 23370.61202(21273.80087 to 25556.36694) |
| Southeast Asia | Male | 2012 | 1157.125638(939.4092676 to 1399.843928) | 1273.214321(1136.877314 to 1396.58349) | 22920.42934(20607.83574 to 25092.90824) |
| Southeast Asia | Male | 2013 | 1147.160563(931.9424387 to 1386.531893) | 1251.556698(1119.800753 to 1380.175451) | 22561.52988(20283.25194 to 24781.83192) |
| Southeast Asia | Male | 2014 | 1139.17571(926.775778 to 1375.588104) | 1232.721701(1098.399138 to 1360.363567) | 22253.34569(20030.27629 to 24518.06187) |
| Southeast Asia | Male | 2015 | 1134.651582(922.4707836 to 1369.656864) | 1217.106538(1087.5262 to 1349.639876) | 22025.91391(19772.67505 to 24338.39761) |
| Southeast Asia | Male | 2016 | 1134.487684(921.72979 to 1368.65291) | 1198.361835(1063.743158 to 1328.342162) | 21737.36344(19418.98532 to 24022.22933) |
| Southeast Asia | Male | 2017 | 1135.515443(921.9830573 to 1370.356049) | 1181.067228(1046.408403 to 1311.450748) | 21456.11389(19076.87385 to 23842.1965) |
| Southeast Asia | Male | 2018 | 1136.605159(922.925554 to 1370.34736) | 1171.726364(1036.747162 to 1304.313326) | 21293.80358(18963.58979 to 23687.37805) |
| Southeast Asia | Male | 2019 | 1136.439019(922.5613253 to 1370.12871) | 1165.633072(1029.822864 to 1297.615331) | 21194.23116(18762.92704 to 23566.78126) |
| Southeast Asia | Male | 2020 | 1109.42596(891.9000383 to 1353.775525) | 1150.207972(1010.925257 to 1280.682144) | 20970.45913(18507.04184 to 23339.44416) |
| Southeast Asia | Male | 2021 | 1114.594635(896.573399 to 1358.432075) | 1148.932154(1010.935487 to 1285.097537) | 20954.20062(18499.08101 to 23378.10254) |
| Southern Latin America | Male | 1990 | 921.5253446(734.3346182 to 1141.486692) | 812.6223377(742.9410048 to 877.6868119) | 14507.48598(13333.95563 to 15624.84416) |
| Southern Latin America | Male | 1991 | 914.0744019(733.044316 to 1124.784488) | 783.6280534(717.4299233 to 847.099422) | 14067.46644(12936.55438 to 15127.38123) |
| Southern Latin America | Male | 1992 | 905.3432544(728.2399923 to 1104.802276) | 765.2484595(700.0625107 to 827.2711579) | 13832.04291(12749.18911 to 14911.80502) |
| Southern Latin America | Male | 1993 | 895.7161738(720.4407064 to 1088.53204) | 736.9920433(673.3770417 to 798.3203587) | 13372.58505(12286.50768 to 14424.66812) |
| Southern Latin America | Male | 1994 | 885.5897336(713.1990744 to 1078.41233) | 686.7980985(627.712836 to 743.6241785) | 12597.6304(11578.97691 to 13578.32891) |
| Southern Latin America | Male | 1995 | 875.3578023(702.199953 to 1070.209876) | 662.0570443(604.2852601 to 717.4439621) | 12213.13914(11246.42992 to 13173.66894) |
| Southern Latin America | Male | 1996 | 862.2288106(694.8628219 to 1050.008481) | 640.3694256(584.6737236 to 694.4817386) | 11837.43229(10907.4766 to 12777.99413) |
| Southern Latin America | Male | 1997 | 844.4146675(681.5307964 to 1027.920754) | 624.8607668(569.4925158 to 677.6305012) | 11507.57718(10593.75347 to 12447.31687) |
| Southern Latin America | Male | 1998 | 823.7842852(666.9933143 to 1002.870139) | 629.468824(574.5307673 to 682.9719744) | 11508.90354(10585.71818 to 12428.2427) |
| Southern Latin America | Male | 1999 | 802.159623(650.2053842 to 978.9572148) | 618.6588034(563.0928495 to 672.331638) | 11272.28705(10374.89992 to 12182.3999) |
| Southern Latin America | Male | 2000 | 781.389338(632.0555217 to 956.0568479) | 579.0749874(526.0737614 to 628.9270839) | 10588.92171(9724.178599 to 11453.52521) |
| Southern Latin America | Male | 2001 | 759.8834982(617.6459408 to 923.4370492) | 564.9591984(513.1780195 to 613.952999) | 10358.75493(9504.100664 to 11204.47422) |
| Southern Latin America | Male | 2002 | 736.1875256(600.1296734 to 889.5891761) | 563.5108074(511.509186 to 612.3664489) | 10319.43551(9470.368737 to 11170.13003) |
| Southern Latin America | Male | 2003 | 712.3598567(580.4599291 to 860.2563084) | 561.1548623(508.4289964 to 610.0089135) | 10211.48475(9351.868332 to 11051.73252) |
| Southern Latin America | Male | 2004 | 690.3724455(561.1596701 to 833.7789086) | 531.4623593(481.3378193 to 577.5832654) | 9653.000844(8854.18751 to 10434.29193) |
| Southern Latin America | Male | 2005 | 672.1977798(543.1406266 to 814.9265201) | 496.0361379(449.0409827 to 539.6362562) | 9036.091668(8284.503449 to 9771.665255) |
| Southern Latin America | Male | 2006 | 656.4906309(535.0164166 to 792.2965741) | 475.3420967(430.4366652 to 517.3388722) | 8667.602153(7942.685587 to 9381.55774) |
| Southern Latin America | Male | 2007 | 641.0667306(523.5390344 to 771.1565429) | 468.956078(425.1447798 to 508.8062701) | 8539.856097(7829.502599 to 9245.952919) |
| Southern Latin America | Male | 2008 | 626.638024(512.2508577 to 754.0753981) | 448.5944319(406.3732824 to 487.0111908) | 8193.197379(7525.376638 to 8854.917841) |
| Southern Latin America | Male | 2009 | 613.9516761(502.4872818 to 742.0576431) | 434.8710851(394.3113636 to 472.1061365) | 7914.637755(7265.962978 to 8546.748763) |
| Southern Latin America | Male | 2010 | 603.7492171(492.3760319 to 732.5722519) | 435.8631228(396.109881 to 474.1784131) | 7871.875932(7222.33812 to 8508.103601) |
| Southern Latin America | Male | 2011 | 594.6860432(487.5029713 to 719.639273) | 423.7332373(384.4183111 to 460.9246323) | 7634.751227(6997.236119 to 8260.680124) |
| Southern Latin America | Male | 2012 | 585.5339281(479.3132462 to 706.0948382) | 412.8282658(374.1808213 to 449.3257304) | 7437.926604(6806.499032 to 8058.061507) |
| Southern Latin America | Male | 2013 | 577.3844014(471.1268738 to 695.0448322) | 413.2788451(374.6500574 to 450.622636) | 7416.623956(6787.886164 to 8037.759969) |
| Southern Latin America | Male | 2014 | 571.3188966(463.6231299 to 688.841352) | 408.0116102(369.7791639 to 444.5023802) | 7299.952067(6674.75114 to 7914.194743) |
| Southern Latin America | Male | 2015 | 568.4008747(457.8496106 to 688.10009) | 407.9652557(369.882884 to 445.7751802) | 7275.6753(6648.250665 to 7905.315188) |
| Southern Latin America | Male | 2016 | 568.7380006(461.4065915 to 686.5177401) | 409.8494179(371.2714033 to 448.259628) | 7289.316665(6672.123636 to 7914.248039) |
| Southern Latin America | Male | 2017 | 570.6746733(463.8994342 to 689.0361527) | 388.7625134(352.9835868 to 424.7492284) | 6915.987112(6321.120755 to 7520.414139) |
| Southern Latin America | Male | 2018 | 572.6328119(464.4414741 to 693.4859297) | 376.019142(340.9593212 to 411.3524787) | 6715.304452(6131.971708 to 7296.215798) |
| Southern Latin America | Male | 2019 | 573.0114941(462.5097174 to 699.8793084) | 367.2965765(331.5445322 to 401.4595898) | 6570.420373(5988.391394 to 7150.127775) |
| Southern Latin America | Male | 2020 | 562.6306136(452.1041464 to 686.348275) | 347.9019941(313.1018003 to 381.2259388) | 6322.096316(5753.631099 to 6896.663677) |
| Southern Latin America | Male | 2021 | 559.2833119(446.7408335 to 681.5343722) | 326.88115(291.9107796 to 359.4353959) | 5972.009341(5415.314088 to 6518.171139) |
| Southern Sub-Saharan Africa | Male | 1990 | 874.1648106(664.7328606 to 1125.897252) | 681.2739481(556.552598 to 784.2695539) | 12250.00165(10102.77059 to 14029.36385) |
| Southern Sub-Saharan Africa | Male | 1991 | 888.0245449(677.6818938 to 1139.817756) | 695.3907315(571.4396987 to 797.410222) | 12476.36788(10375.84574 to 14201.08998) |
| Southern Sub-Saharan Africa | Male | 1992 | 902.6597008(692.28234 to 1154.479117) | 708.0556745(582.8096929 to 811.8750453) | 12691.15136(10596.33695 to 14452.79612) |
| Southern Sub-Saharan Africa | Male | 1993 | 917.7962951(705.9023493 to 1172.360669) | 716.5792523(598.5863361 to 815.9219809) | 12817.78311(10856.04374 to 14498.27915) |
| Southern Sub-Saharan Africa | Male | 1994 | 933.0243189(717.188372 to 1189.667035) | 752.041288(638.2469994 to 851.0323011) | 13443.86486(11574.07076 to 15099.93292) |
| Southern Sub-Saharan Africa | Male | 1995 | 947.9988592(728.7495187 to 1208.462765) | 802.2930499(700.0687786 to 895.5244721) | 14351.0828(12649.83308 to 15942.10462) |
| Southern Sub-Saharan Africa | Male | 1996 | 965.2318906(742.9113874 to 1229.333834) | 867.3142086(771.4143369 to 957.2544206) | 15533.33126(13961.04777 to 17063.21386) |
| Southern Sub-Saharan Africa | Male | 1997 | 985.525987(758.5575491 to 1255.120014) | 933.2046659(839.5872355 to 1021.220075) | 16777.66296(15222.03037 to 18258.12694) |
| Southern Sub-Saharan Africa | Male | 1998 | 1006.299716(774.2664207 to 1282.024689) | 970.9416702(877.5996964 to 1059.936065) | 17495.90168(15935.89292 to 19023.29036) |
| Southern Sub-Saharan Africa | Male | 1999 | 1024.733496(788.1950055 to 1306.860905) | 946.7481274(854.6608272 to 1032.470265) | 17058.20276(15529.32768 to 18520.89734) |
| Southern Sub-Saharan Africa | Male | 2000 | 1038.041627(797.8590442 to 1326.183671) | 969.132406(874.9966477 to 1056.689723) | 17528.68332(15977.23079 to 19053.75592) |
| Southern Sub-Saharan Africa | Male | 2001 | 1047.827434(804.8762276 to 1340.740681) | 972.9037971(880.5833624 to 1061.82258) | 17551.06398(15996.74338 to 19087.83221) |
| Southern Sub-Saharan Africa | Male | 2002 | 1056.933293(811.8347042 to 1355.151888) | 994.710643(900.1553536 to 1087.418264) | 17936.23969(16331.39322 to 19569.12676) |
| Southern Sub-Saharan Africa | Male | 2003 | 1064.374196(816.7060467 to 1365.916712) | 1016.239611(921.1235637 to 1110.646246) | 18306.91763(16679.77009 to 19958.81819) |
| Southern Sub-Saharan Africa | Male | 2004 | 1068.731142(818.6324881 to 1371.921386) | 1002.949258(909.3453645 to 1095.703927) | 18090.67598(16520.87523 to 19708.97928) |
| Southern Sub-Saharan Africa | Male | 2005 | 1068.798165(817.5374696 to 1374.136827) | 999.6579644(905.0329442 to 1091.85844) | 18036.95783(16444.8851 to 19641.90703) |
| Southern Sub-Saharan Africa | Male | 2006 | 1060.926041(812.8631365 to 1361.339952) | 1011.51724(919.2399077 to 1104.409574) | 18217.99176(16683.44461 to 19820.68533) |
| Southern Sub-Saharan Africa | Male | 2007 | 1044.924564(801.9388281 to 1338.586031) | 1006.202363(916.4310699 to 1097.035323) | 18080.97431(16540.50812 to 19630.53566) |
| Southern Sub-Saharan Africa | Male | 2008 | 1024.748866(787.0126208 to 1310.708438) | 1005.92043(916.6972424 to 1097.428167) | 18063.9937(16544.03144 to 19612.30954) |
| Southern Sub-Saharan Africa | Male | 2009 | 1004.233163(770.8589781 to 1282.191271) | 1007.73691(920.261313 to 1098.175753) | 18084.82055(16589.78952 to 19599.58484) |
| Southern Sub-Saharan Africa | Male | 2010 | 987.3885139(757.9072686 to 1259.051848) | 1007.603582(920.1502456 to 1100.069449) | 18001.71888(16505.02767 to 19582.50552) |
| Southern Sub-Saharan Africa | Male | 2011 | 972.2322847(747.7961222 to 1237.037332) | 986.4779655(901.4346609 to 1074.903219) | 17599.48456(16154.62761 to 19090.70395) |
| Southern Sub-Saharan Africa | Male | 2012 | 955.5561458(736.1850402 to 1214.975054) | 958.6081888(874.9914385 to 1043.532948) | 17120.72542(15717.71261 to 18569.28991) |
| Southern Sub-Saharan Africa | Male | 2013 | 939.6887667(725.4031308 to 1194.206575) | 931.5996225(849.706265 to 1016.05157) | 16636.25269(15281.6226 to 18053.74861) |
| Southern Sub-Saharan Africa | Male | 2014 | 926.9304555(716.3456989 to 1176.536213) | 917.8823645(836.789568 to 999.7291528) | 16400.02984(15064.90504 to 17786.84014) |
| Southern Sub-Saharan Africa | Male | 2015 | 919.5824646(712.7795601 to 1168.591709) | 911.7211285(830.9573052 to 996.6830427) | 16280.5763(14945.63537 to 17714.26457) |
| Southern Sub-Saharan Africa | Male | 2016 | 916.9480739(709.112446 to 1164.208718) | 898.9045992(816.2665899 to 981.2737185) | 16034.60652(14670.73825 to 17448.76701) |
| Southern Sub-Saharan Africa | Male | 2017 | 915.6410975(707.3160416 to 1160.822067) | 867.1208786(786.5461549 to 950.6400245) | 15459.06212(14108.64002 to 16874.40531) |
| Southern Sub-Saharan Africa | Male | 2018 | 913.9162787(705.7490409 to 1156.022689) | 843.5360283(761.4802062 to 926.0970652) | 15024.9119(13666.0257 to 16435.58304) |
| Southern Sub-Saharan Africa | Male | 2019 | 909.9367477(703.7356416 to 1151.400883) | 807.5866551(726.6685266 to 887.9472516) | 14321.89927(12959.9609 to 15690.13362) |
| Southern Sub-Saharan Africa | Male | 2020 | 863.5443328(663.6467576 to 1101.726409) | 786.5665688(704.4736488 to 867.3157752) | 14070.66265(12678.20593 to 15464.66589) |
| Southern Sub-Saharan Africa | Male | 2021 | 872.5034621(669.0377392 to 1115.222982) | 761.5489932(680.7933077 to 843.7949721) | 13799.15298(12412.12603 to 15229.64062) |
| Tropical Latin America | Male | 1990 | 1060.19708(779.0350928 to 1402.476994) | 990.6216554(918.078752 to 1043.10211) | 17129.98346(16088.8146 to 17949.91982) |
| Tropical Latin America | Male | 1991 | 1053.151247(777.8428174 to 1386.421925) | 940.2679959(869.4363367 to 989.4437868) | 16342.98279(15344.61316 to 17112.75675) |
| Tropical Latin America | Male | 1992 | 1044.529584(774.8511442 to 1370.325942) | 928.1418524(858.9187669 to 976.0518854) | 16185.38865(15208.72968 to 16940.79862) |
| Tropical Latin America | Male | 1993 | 1034.798501(771.4291613 to 1353.05915) | 930.4753364(862.7580846 to 976.9637147) | 16253.85606(15297.29083 to 16973.15302) |
| Tropical Latin America | Male | 1994 | 1024.388092(765.8183692 to 1337.560276) | 889.136368(821.5426825 to 934.9438543) | 15584.16678(14653.9262 to 16301.09812) |
| Tropical Latin America | Male | 1995 | 1013.756093(758.8255162 to 1322.424647) | 844.9809842(777.8416779 to 888.9453646) | 14865.35096(13945.96845 to 15560.57834) |
| Tropical Latin America | Male | 1996 | 999.3571039(749.4918599 to 1300.681562) | 804.6469114(739.1104315 to 847.5109123) | 14195.11211(13305.64683 to 14879.8971) |
| Tropical Latin America | Male | 1997 | 979.2356656(735.1406124 to 1274.730916) | 773.9577674(710.6752472 to 816.1177444) | 13689.81169(12813.85954 to 14347.7107) |
| Tropical Latin America | Male | 1998 | 956.2078319(718.8218334 to 1244.185966) | 758.7383893(696.5127234 to 799.1264117) | 13445.56219(12585.07803 to 14090.24406) |
| Tropical Latin America | Male | 1999 | 933.1452185(701.8659774 to 1215.776891) | 735.9312921(675.5166585 to 775.645992) | 13072.79177(12250.90403 to 13695.50391) |
| Tropical Latin America | Male | 2000 | 912.9309358(686.5655888 to 1190.886457) | 707.7655811(648.0076579 to 747.1759989) | 12555.41926(11730.81127 to 13180.31173) |
| Tropical Latin America | Male | 2001 | 894.6227558(672.6786824 to 1167.80806) | 696.2910353(637.7560521 to 735.6847501) | 12304.99614(11507.15142 to 12924.00099) |
| Tropical Latin America | Male | 2002 | 875.6538772(658.4735026 to 1142.761892) | 683.2950567(624.9043248 to 721.480767) | 12038.31308(11245.94607 to 12625.89726) |
| Tropical Latin America | Male | 2003 | 856.5418918(643.1697707 to 1117.987766) | 672.935966(616.0520706 to 710.184833) | 11832.94389(11056.73437 to 12402.7386) |
| Tropical Latin America | Male | 2004 | 837.7612314(628.0305471 to 1092.743942) | 657.8144192(602.2622572 to 693.2282882) | 11566.22878(10808.40072 to 12121.19419) |
| Tropical Latin America | Male | 2005 | 819.7829802(614.0619156 to 1069.474743) | 624.2469562(569.5712517 to 659.9796223) | 10963.99939(10218.18326 to 11501.96963) |
| Tropical Latin America | Male | 2006 | 801.2806188(601.4961278 to 1044.340981) | 619.4321366(565.1649056 to 654.3433299) | 10830.91488(10089.74548 to 11357.67995) |
| Tropical Latin America | Male | 2007 | 781.5107548(587.9033322 to 1014.928435) | 608.3128222(554.662111 to 642.0926629) | 10601.59721(9871.888248 to 11105.01528) |
| Tropical Latin America | Male | 2008 | 761.940978(574.2069506 to 986.0028941) | 596.3966332(542.726924 to 629.1848531) | 10383.20549(9663.242627 to 10882.23177) |
| Tropical Latin America | Male | 2009 | 744.0555961(560.7488691 to 961.6706372) | 588.2423485(535.8572086 to 619.7522912) | 10206.01056(9500.867192 to 10690.34066) |
| Tropical Latin America | Male | 2010 | 729.1944296(549.5691535 to 941.7769761) | 577.9169596(526.547957 to 609.641546) | 9983.220881(9289.579593 to 10468.93305) |
| Tropical Latin America | Male | 2011 | 715.7971238(542.0771482 to 922.842531) | 562.7772858(511.9682568 to 594.4341968) | 9694.931054(9007.823901 to 10169.29703) |
| Tropical Latin America | Male | 2012 | 702.3385089(533.8515601 to 904.8244231) | 540.7430099(491.0391729 to 571.1895938) | 9308.370076(8648.011218 to 9759.331681) |
| Tropical Latin America | Male | 2013 | 690.216608(526.3121581 to 887.7800034) | 522.3096673(474.5906644 to 551.5003912) | 8986.483621(8354.00782 to 9442.735519) |
| Tropical Latin America | Male | 2014 | 680.898249(519.9935246 to 874.6064276) | 499.5484202(452.6128213 to 527.1351541) | 8605.605643(7981.12588 to 9039.597416) |
| Tropical Latin America | Male | 2015 | 675.7719462(515.7014053 to 869.8827968) | 487.3327033(442.5430299 to 514.5450215) | 8386.777495(7797.3951 to 8811.559111) |
| Tropical Latin America | Male | 2016 | 674.2342065(514.4551622 to 866.8935302) | 486.5690069(441.2156653 to 514.0632969) | 8383.277972(7789.426492 to 8822.981296) |
| Tropical Latin America | Male | 2017 | 673.8372132(513.7870289 to 866.5908521) | 463.7331393(419.7252187 to 490.2901728) | 8010.194549(7429.528968 to 8437.906818) |
| Tropical Latin America | Male | 2018 | 673.1346119(512.274721 to 866.7643239) | 448.4213153(405.4663577 to 474.9252069) | 7767.217282(7184.228477 to 8188.938549) |
| Tropical Latin America | Male | 2019 | 670.6699258(508.7726488 to 865.6159599) | 440.7277752(399.6090474 to 467.3911558) | 7635.529288(7064.78126 to 8056.21863) |
| Tropical Latin America | Male | 2020 | 653.8988186(491.3037042 to 849.8391564) | 432.9885136(390.3459331 to 460.615565) | 7537.55545(6963.383802 to 7967.385342) |
| Tropical Latin America | Male | 2021 | 637.4049522(479.1012811 to 824.8164962) | 410.3987873(368.5192055 to 440.3830476) | 7193.824183(6603.941003 to 7671.732678) |
| Western Europe | Male | 1990 | 934.22474(753.1140026 to 1140.203949) | 708.1406085(655.7575315 to 740.0088675) | 11054.32096(10370.87636 to 11566.21255) |
| Western Europe | Male | 1991 | 919.4643419(743.4868897 to 1115.664979) | 690.9021469(639.2802315 to 722.3750061) | 10788.47673(10117.38263 to 11285.34559) |
| Western Europe | Male | 1992 | 903.9633933(732.3839932 to 1093.690164) | 662.8123174(612.2909623 to 692.8964942) | 10372.9295(9704.00272 to 10857.02892) |
| Western Europe | Male | 1993 | 888.3156392(723.0994028 to 1071.193144) | 637.9397122(589.3559336 to 666.8783158) | 10000.4241(9343.459967 to 10472.60507) |
| Western Europe | Male | 1994 | 873.111814(713.7170197 to 1052.671171) | 611.4956979(564.1595479 to 639.596083) | 9619.099535(8980.228511 to 10081.08147) |
| Western Europe | Male | 1995 | 858.6904802(704.5098347 to 1035.037121) | 595.1940779(549.1249796 to 623.8412899) | 9368.974673(8739.995567 to 9827.95738) |
| Western Europe | Male | 1996 | 842.3746947(693.7230515 to 1010.836451) | 576.9337929(531.3750391 to 605.479155) | 9088.672713(8472.897452 to 9542.103893) |
| Western Europe | Male | 1997 | 822.9180314(679.7990379 to 983.0648898) | 554.1486333(509.2960174 to 581.7377841) | 8751.060627(8142.897424 to 9185.667366) |
| Western Europe | Male | 1998 | 802.3747943(664.2312961 to 955.7120951) | 535.281726(491.4563343 to 561.9010539) | 8478.324673(7890.240111 to 8900.765596) |
| Western Europe | Male | 1999 | 782.8111431(649.5789121 to 929.1648177) | 512.9146429(469.3046124 to 538.2302948) | 8160.251104(7588.614523 to 8580.684198) |
| Western Europe | Male | 2000 | 766.0052154(636.7681058 to 906.0302162) | 483.5379723(440.9652051 to 508.2412115) | 7731.407405(7171.909256 to 8143.60306) |
| Western Europe | Male | 2001 | 750.2837802(626.2620014 to 884.7990461) | 467.1229372(425.4337942 to 491.59091) | 7464.991587(6904.516198 to 7869.790772) |
| Western Europe | Male | 2002 | 733.2010702(613.8993804 to 862.5730527) | 455.3824136(414.567656 to 479.2069092) | 7263.99492(6719.895561 to 7668.009848) |
| Western Europe | Male | 2003 | 715.5677488(600.3180428 to 841.0513879) | 438.5829292(398.6936008 to 461.9412974) | 6998.715121(6468.136246 to 7392.578931) |
| Western Europe | Male | 2004 | 697.8918557(585.8854941 to 819.794118) | 402.3614555(363.9706471 to 424.6038964) | 6490.858446(5976.482797 to 6875.774474) |
| Western Europe | Male | 2005 | 680.334448(572.1113 to 799.7292871) | 381.579838(344.3850562 to 403.1487593) | 6178.295045(5683.910375 to 6557.09196) |
| Western Europe | Male | 2006 | 661.3445937(556.3055088 to 776.6885509) | 360.7336988(324.8460501 to 381.5977017) | 5868.647665(5387.208689 to 6239.163318) |
| Western Europe | Male | 2007 | 639.770386(538.7516472 to 750.5128016) | 344.7114518(310.3544907 to 365.0194298) | 5620.31333(5157.318957 to 5983.111347) |
| Western Europe | Male | 2008 | 617.9644767(521.1171641 to 724.8203935) | 334.1090623(300.4709354 to 353.8913418) | 5442.328727(4990.065621 to 5791.60944) |
| Western Europe | Male | 2009 | 598.1759767(504.8337597 to 702.6668643) | 318.1334773(285.7159788 to 337.2162986) | 5205.781039(4768.498252 to 5546.59161) |
| Western Europe | Male | 2010 | 582.3927655(491.2938861 to 684.183686) | 301.8925503(270.5728682 to 319.9471305) | 4969.171511(4542.29498 to 5300.951171) |
| Western Europe | Male | 2011 | 568.8855586(479.4986117 to 667.6838986) | 289.233425(258.5948222 to 306.9461149) | 4774.355072(4355.17796 to 5098.771498) |
| Western Europe | Male | 2012 | 555.452045(468.26532 to 651.6072365) | 282.3931275(252.2371914 to 299.8088469) | 4660.633615(4242.854814 to 4975.027466) |
| Western Europe | Male | 2013 | 542.9987846(457.76164 to 637.1020094) | 272.2390256(242.4362886 to 289.4015466) | 4516.463181(4102.124462 to 4830.594086) |
| Western Europe | Male | 2014 | 532.5742137(448.6773549 to 625.7752126) | 261.1406011(232.1423896 to 277.7632428) | 4352.81515(3939.43432 to 4664.265454) |
| Western Europe | Male | 2015 | 525.0046417(441.6384648 to 617.7935912) | 260.3310016(231.9175892 to 276.9554033) | 4336.093862(3926.257653 to 4647.50963) |
| Western Europe | Male | 2016 | 518.7698809(434.7756549 to 610.9697678) | 252.6572085(224.6128832 to 269.082792) | 4228.034928(3829.348573 to 4537.05803) |
| Western Europe | Male | 2017 | 512.377157(427.1710129 to 605.6847847) | 246.4493696(219.0426985 to 262.4263053) | 4135.480752(3739.568591 to 4441.216927) |
| Western Europe | Male | 2018 | 506.6031589(419.9967214 to 602.2960982) | 239.9774928(212.7899899 to 255.8611679) | 4044.976007(3656.578233 to 4347.306678) |
| Western Europe | Male | 2019 | 502.2231833(414.1980025 to 599.7098241) | 230.9030527(204.056965 to 246.9014374) | 3916.733678(3528.624144 to 4222.805459) |
| Western Europe | Male | 2020 | 496.6927307(407.8407775 to 596.6394542) | 227.1201633(200.3967863 to 242.9444666) | 3844.778487(3463.318659 to 4149.791199) |
| Western Europe | Male | 2021 | 495.8815092(406.4684266 to 596.2511079) | 219.9057313(193.394297 to 235.4602607) | 3743.438979(3371.950199 to 4041.246345) |
| Western Sub-Saharan Africa | Male | 1990 | 1092.423632(862.968722 to 1361.984393) | 1164.635521(951.4077626 to 1387.091085) | 20975.81522(17288.33694 to 24893.83504) |
| Western Sub-Saharan Africa | Male | 1991 | 1089.451281(862.9439477 to 1353.69618) | 1162.414407(948.4925175 to 1371.807586) | 20940.11131(17237.28703 to 24645.94091) |
| Western Sub-Saharan Africa | Male | 1992 | 1086.539337(863.6893655 to 1346.698919) | 1163.910057(961.3025085 to 1380.397163) | 20977.97769(17526.31265 to 24786.48412) |
| Western Sub-Saharan Africa | Male | 1993 | 1083.568244(865.1994952 to 1339.828002) | 1165.3747(962.4152537 to 1383.105939) | 21015.17808(17491.84649 to 24836.21769) |
| Western Sub-Saharan Africa | Male | 1994 | 1080.786194(865.4452838 to 1333.828063) | 1165.441072(965.3679048 to 1382.351815) | 21021.7368(17521.41704 to 24906.13293) |
| Western Sub-Saharan Africa | Male | 1995 | 1078.214115(865.1147959 to 1328.183939) | 1171.641828(970.8152542 to 1394.373913) | 21145.81596(17636.77234 to 25076.99249) |
| Western Sub-Saharan Africa | Male | 1996 | 1075.273443(863.0146482 to 1323.374838) | 1177.158452(973.9007968 to 1393.024821) | 21250.02793(17728.71702 to 25082.05356) |
| Western Sub-Saharan Africa | Male | 1997 | 1071.457144(859.497531 to 1318.195496) | 1172.150796(967.5389416 to 1387.591629) | 21151.47993(17513.77999 to 24971.52692) |
| Western Sub-Saharan Africa | Male | 1998 | 1066.984305(855.1983982 to 1312.268171) | 1166.736573(970.2062902 to 1387.854826) | 21052.99286(17623.31608 to 25001.82494) |
| Western Sub-Saharan Africa | Male | 1999 | 1062.295389(850.2750556 to 1306.436047) | 1160.640719(963.071839 to 1379.004639) | 20941.34021(17486.40444 to 24812.82022) |
| Western Sub-Saharan Africa | Male | 2000 | 1057.720166(845.747184 to 1300.622074) | 1158.764682(975.8018872 to 1360.666489) | 20865.92035(17606.41163 to 24431.46949) |
| Western Sub-Saharan Africa | Male | 2001 | 1053.564889(842.5235164 to 1295.166289) | 1147.945992(961.9309955 to 1355.520548) | 20713.10775(17384.92514 to 24407.0993) |
| Western Sub-Saharan Africa | Male | 2002 | 1049.259066(838.7799241 to 1289.96807) | 1143.312643(961.1304997 to 1350.264656) | 20576.73966(17394.895 to 24335.92754) |
| Western Sub-Saharan Africa | Male | 2003 | 1044.426747(834.9166849 to 1284.497509) | 1120.406957(931.8447468 to 1308.73188) | 20183.04398(16791.19814 to 23508.67139) |
| Western Sub-Saharan Africa | Male | 2004 | 1038.753471(830.226297 to 1277.244024) | 1102.474836(938.9667951 to 1294.15511) | 19840.80051(16910.76439 to 23255.70129) |
| Western Sub-Saharan Africa | Male | 2005 | 1031.852778(824.5221897 to 1269.704481) | 1081.515657(914.0515027 to 1259.465502) | 19446.93702(16480.28034 to 22670.74342) |
| Western Sub-Saharan Africa | Male | 2006 | 1021.538598(817.2730454 to 1255.571823) | 1069.384778(904.8429717 to 1247.431908) | 19170.53633(16241.4693 to 22301.68356) |
| Western Sub-Saharan Africa | Male | 2007 | 1007.209132(807.5252931 to 1236.131061) | 1047.151537(893.9260021 to 1229.553507) | 18795.30204(16124.04286 to 22046.31974) |
| Western Sub-Saharan Africa | Male | 2008 | 991.2489216(795.7790682 to 1214.37493) | 1033.841612(884.5581663 to 1208.562695) | 18499.45967(15893.70091 to 21515.28218) |
| Western Sub-Saharan Africa | Male | 2009 | 975.919988(783.9848305 to 1195.025281) | 1023.523361(867.8543639 to 1198.156126) | 18323.2929(15619.04266 to 21440.75057) |
| Western Sub-Saharan Africa | Male | 2010 | 962.7731931(774.1812364 to 1179.442415) | 1014.453328(864.4499916 to 1190.850863) | 18155.45748(15561.78841 to 21219.48834) |
| Western Sub-Saharan Africa | Male | 2011 | 950.6914145(765.537926 to 1162.463617) | 1005.740598(858.0683994 to 1173.467339) | 17992.92779(15430.68693 to 20934.58177) |
| Western Sub-Saharan Africa | Male | 2012 | 937.4070895(755.0487692 to 1143.652448) | 997.2169403(842.8528275 to 1159.102563) | 17861.52355(15170.10968 to 20660.05794) |
| Western Sub-Saharan Africa | Male | 2013 | 925.2188261(746.1618066 to 1127.26406) | 995.2978124(841.1772929 to 1173.817744) | 17834.08621(15197.86931 to 20987.21742) |
| Western Sub-Saharan Africa | Male | 2014 | 916.2087967(739.6565245 to 1116.289934) | 983.291307(838.1057079 to 1143.254022) | 17661.3366(15167.99795 to 20418.16473) |
| Western Sub-Saharan Africa | Male | 2015 | 912.2861586(737.2609129 to 1112.48987) | 978.9639393(832.643056 to 1149.673921) | 17535.73686(14965.08848 to 20458.08282) |
| Western Sub-Saharan Africa | Male | 2016 | 913.2420538(738.0687203 to 1113.450923) | 964.5272366(824.3018352 to 1132.611884) | 17288.85716(14860.11162 to 20179.29641) |
| Western Sub-Saharan Africa | Male | 2017 | 915.8433191(739.6472635 to 1116.691045) | 952.2148794(805.4465118 to 1118.347942) | 17048.08535(14493.0694 to 19897.89656) |
| Western Sub-Saharan Africa | Male | 2018 | 918.4505201(741.0788535 to 1120.005254) | 940.9324865(795.2300557 to 1109.761615) | 16811.22674(14259.75665 to 19705.11674) |
| Western Sub-Saharan Africa | Male | 2019 | 919.209645(741.5093566 to 1120.972366) | 930.6282037(787.3389751 to 1095.621455) | 16596.40556(14132.45255 to 19437.50922) |
| Western Sub-Saharan Africa | Male | 2020 | 893.5327828(716.8243961 to 1096.366538) | 921.4884696(778.8551519 to 1094.915361) | 16429.16367(13997.32503 to 19394.66422) |
| Western Sub-Saharan Africa | Male | 2021 | 892.8085621(715.2037692 to 1095.291389) | 912.3539958(769.0050811 to 1077.064013) | 16264.54101(13771.92598 to 19076.82348) |
| Andean Latin America | Female | 1990 | 532.8081667(427.2716886 to 657.3557565) | 458.9687807(377.585111 to 557.2479769) | 7975.944152(6627.596519 to 9628.308056) |
| Andean Latin America | Female | 1991 | 527.4199447(425.6314999 to 646.8205231) | 452.4168507(373.4318222 to 545.3294677) | 7917.642718(6603.779842 to 9493.35418) |
| Andean Latin America | Female | 1992 | 521.2952889(422.1031744 to 637.4156999) | 469.7750228(387.2218576 to 568.4239944) | 8170.940156(6811.69286 to 9800.721632) |
| Andean Latin America | Female | 1993 | 514.5845382(416.6654638 to 628.2610777) | 463.7227237(383.245758 to 562.4816018) | 8036.284619(6706.824825 to 9685.57337) |
| Andean Latin America | Female | 1994 | 507.4351709(410.8672872 to 620.3046957) | 468.9663309(386.3228186 to 569.7910166) | 8108.485743(6740.04521 to 9830.992232) |
| Andean Latin America | Female | 1995 | 500.024548(404.4906159 to 610.8733566) | 465.9292158(382.9784286 to 566.8189881) | 8010.675997(6645.956314 to 9698.898338) |
| Andean Latin America | Female | 1996 | 491.3445658(398.9453043 to 596.8659669) | 438.795149(362.5757194 to 529.8602078) | 7497.374728(6264.544677 to 9004.210561) |
| Andean Latin America | Female | 1997 | 480.7206156(391.4838484 to 582.8566819) | 424.8669169(351.9505202 to 512.6722676) | 7236.438589(6046.757491 to 8703.199488) |
| Andean Latin America | Female | 1998 | 469.3752145(382.3609768 to 570.4090619) | 412.2414668(341.997648 to 492.4265386) | 6992.51505(5842.254011 to 8327.057399) |
| Andean Latin America | Female | 1999 | 458.2538281(373.0194338 to 558.378676) | 383.8931792(319.424935 to 457.5966207) | 6508.956291(5469.660006 to 7703.944418) |
| Andean Latin America | Female | 2000 | 448.1885614(363.7773553 to 548.2695742) | 367.6347982(304.5342168 to 435.7554561) | 6226.644475(5225.165318 to 7328.712051) |
| Andean Latin America | Female | 2001 | 439.2628275(356.8983564 to 536.6205183) | 356.1214348(294.3762877 to 422.2442626) | 6018.736782(5021.218069 to 7086.80731) |
| Andean Latin America | Female | 2002 | 430.5857139(349.594382 to 526.6362609) | 359.1945115(296.665763 to 426.5746709) | 6044.859488(5031.927776 to 7129.139827) |
| Andean Latin America | Female | 2003 | 422.3577094(342.1130027 to 516.7925148) | 351.3768532(290.9075038 to 419.9581735) | 5908.577421(4924.199259 to 7030.983821) |
| Andean Latin America | Female | 2004 | 414.3740941(334.69015 to 508.6394468) | 335.5518103(277.1339644 to 401.1273073) | 5648.125888(4733.418657 to 6712.753717) |
| Andean Latin America | Female | 2005 | 406.3874187(327.6104463 to 502.3422907) | 328.9899255(272.9164423 to 393.8596367) | 5550.465825(4675.700854 to 6607.141102) |
| Andean Latin America | Female | 2006 | 397.6203097(321.4047598 to 489.4107536) | 310.1604332(256.6066313 to 371.9146363) | 5253.426349(4422.673436 to 6254.389998) |
| Andean Latin America | Female | 2007 | 388.0253532(313.9529741 to 477.1403511) | 296.4122023(244.2574185 to 356.0466326) | 5042.489358(4212.413589 to 6023.669225) |
| Andean Latin America | Female | 2008 | 378.8624678(306.509689 to 466.3001929) | 288.9019323(237.3184862 to 348.1744262) | 4928.224993(4106.776077 to 5904.68171) |
| Andean Latin America | Female | 2009 | 371.3125527(300.4797166 to 457.2648259) | 289.1351934(235.5874896 to 351.2222629) | 4954.336765(4082.344563 to 5973.247593) |
| Andean Latin America | Female | 2010 | 366.4576008(296.5684265 to 452.0857402) | 285.6295104(231.7237708 to 348.05447) | 4897.835542(4046.596959 to 5902.426644) |
| Andean Latin America | Female | 2011 | 363.4455015(295.1180452 to 447.599304) | 271.818778(220.3355285 to 331.5492256) | 4665.742903(3846.422425 to 5642.460367) |
| Andean Latin America | Female | 2012 | 360.49015(293.2638553 to 442.1554031) | 266.8306419(216.6201281 to 325.1435609) | 4572.8351(3774.344207 to 5523.303583) |
| Andean Latin America | Female | 2013 | 357.9514653(290.6445826 to 438.4307254) | 264.6249239(215.2323169 to 320.7258536) | 4514.566617(3738.768381 to 5430.878951) |
| Andean Latin America | Female | 2014 | 356.0488628(288.6263978 to 436.7377205) | 257.8280942(210.6292661 to 311.3002964) | 4398.852593(3655.149725 to 5280.398879) |
| Andean Latin America | Female | 2015 | 355.0555151(286.8481433 to 436.1043232) | 250.8308114(205.4216231 to 301.4294896) | 4288.350774(3577.302662 to 5120.683618) |
| Andean Latin America | Female | 2016 | 355.2051947(288.0932281 to 435.3326034) | 248.9429417(204.979755 to 299.8542762) | 4269.623837(3575.612329 to 5095.180929) |
| Andean Latin America | Female | 2017 | 356.0503866(289.3968623 to 436.6552362) | 259.4673578(211.4665163 to 314.8211395) | 4430.981806(3687.692629 to 5335.717614) |
| Andean Latin America | Female | 2018 | 356.9853139(289.9939501 to 437.428048) | 262.2686721(211.8947756 to 321.8346416) | 4470.360263(3686.20563 to 5429.540265) |
| Andean Latin America | Female | 2019 | 357.3799855(289.5124261 to 439.2201923) | 267.8471673(215.7729352 to 333.0291072) | 4534.048382(3721.823261 to 5572.287597) |
| Andean Latin America | Female | 2020 | 345.8471592(277.8871312 to 428.5765779) | 250.4479267(198.7774666 to 314.7255771) | 4321.656434(3509.178526 to 5343.496414) |
| Andean Latin America | Female | 2021 | 345.8359816(277.322207 to 426.2687234) | 242.9355044(192.1024077 to 308.2343334) | 4216.738478(3397.705369 to 5259.469841) |
| Australasia | Female | 1990 | 587.7267953(488.7657889 to 696.610696) | 454.2193326(385.1221786 to 504.9313517) | 6719.075224(5859.839642 to 7401.889689) |
| Australasia | Female | 1991 | 578.6433892(482.5788095 to 680.8620094) | 427.8155753(361.7197947 to 477.2645819) | 6352.235008(5512.035351 to 7020.118687) |
| Australasia | Female | 1992 | 569.4043732(476.4024121 to 668.3154503) | 416.2670481(351.0370839 to 465.2880625) | 6170.443455(5343.766812 to 6828.272831) |
| Australasia | Female | 1993 | 560.1093168(469.6736895 to 655.9235261) | 414.4954729(347.0411974 to 462.9371968) | 6109.171333(5274.991778 to 6765.844708) |
| Australasia | Female | 1994 | 550.8664754(461.5233935 to 645.162562) | 411.4899043(343.8113698 to 460.1228556) | 6027.294692(5192.462077 to 6682.460649) |
| Australasia | Female | 1995 | 541.8224013(452.9944786 to 634.4750457) | 401.4222486(334.3083653 to 449.016918) | 5879.517378(5067.771577 to 6521.213621) |
| Australasia | Female | 1996 | 532.6787974(445.8973288 to 623.197178) | 383.4316518(318.8162575 to 428.837769) | 5636.198431(4843.891289 to 6252.943128) |
| Australasia | Female | 1997 | 523.1204251(438.1180909 to 610.1590106) | 358.6040162(296.9829828 to 402.2128853) | 5287.781679(4530.148784 to 5872.046944) |
| Australasia | Female | 1998 | 513.2038289(428.6220331 to 600.3903648) | 347.6715946(286.8959282 to 389.8536423) | 5124.166528(4380.182341 to 5702.929483) |
| Australasia | Female | 1999 | 503.0205184(418.5123058 to 590.1273345) | 337.9898564(278.8290017 to 379.0395329) | 4963.792251(4239.208576 to 5530.242292) |
| Australasia | Female | 2000 | 492.6561871(407.3634396 to 579.671337) | 324.7180677(267.3738423 to 364.4556563) | 4752.499033(4047.79976 to 5302.351272) |
| Australasia | Female | 2001 | 482.0599094(398.8832285 to 567.1978487) | 311.2301626(255.4666673 to 349.4592338) | 4549.497001(3859.043338 to 5068.270225) |
| Australasia | Female | 2002 | 471.1215435(390.6606666 to 553.7541582) | 305.7722279(250.7112674 to 343.5664264) | 4446.864773(3766.864849 to 4957.348927) |
| Australasia | Female | 2003 | 459.7447496(381.188487 to 541.8698669) | 292.0203998(238.5009943 to 328.4496329) | 4264.095886(3609.120964 to 4764.697938) |
| Australasia | Female | 2004 | 447.7689446(369.6312059 to 528.4365272) | 278.8268293(226.5785848 to 313.4214652) | 4074.493076(3439.16787 to 4558.753471) |
| Australasia | Female | 2005 | 435.0545784(357.2746726 to 516.3853358) | 263.2770953(213.1183776 to 297.1078641) | 3849.646461(3246.100915 to 4310.784528) |
| Australasia | Female | 2006 | 418.8512708(345.4150972 to 495.5511509) | 255.1060347(205.9229905 to 288.2509226) | 3729.186184(3131.804253 to 4181.343456) |
| Australasia | Female | 2007 | 398.8558877(330.1242321 to 471.281043) | 252.479252(203.31273 to 285.5960845) | 3669.686293(3065.857204 to 4119.902886) |
| Australasia | Female | 2008 | 378.532145(312.8996656 to 449.4348262) | 248.8378746(200.0826974 to 281.5613586) | 3595.175935(2999.948312 to 4031.313129) |
| Australasia | Female | 2009 | 361.3571423(299.4707926 to 430.8542031) | 236.9840861(190.0038859 to 268.6200502) | 3422.437818(2849.931702 to 3841.335953) |
| Australasia | Female | 2010 | 350.8256973(289.5019305 to 421.2364743) | 230.5118114(184.5982064 to 260.7193032) | 3337.074331(2781.936123 to 3752.323806) |
| Australasia | Female | 2011 | 345.0536148(285.1774522 to 412.5201999) | 225.7515302(180.4070622 to 255.5406807) | 3261.046327(2709.488648 to 3671.856897) |
| Australasia | Female | 2012 | 339.9287419(280.3680071 to 405.8757828) | 215.2888103(171.0340693 to 244.2425116) | 3125.288517(2592.541792 to 3521.504753) |
| Australasia | Female | 2013 | 335.588512(276.3847989 to 402.1974826) | 205.3113537(162.1295997 to 233.2386887) | 2994.785646(2479.191433 to 3378.353931) |
| Australasia | Female | 2014 | 332.1820754(272.3259201 to 399.8302806) | 203.0552747(161.1692979 to 230.3223752) | 2957.500981(2444.325317 to 3333.70064) |
| Australasia | Female | 2015 | 329.8778455(268.3089262 to 399.0052417) | 199.068886(157.5949027 to 226.0606806) | 2900.451366(2387.824786 to 3271.301533) |
| Australasia | Female | 2016 | 328.0302319(267.5981225 to 395.7369423) | 188.4592027(148.8059173 to 214.3819007) | 2768.775845(2276.055343 to 3126.619857) |
| Australasia | Female | 2017 | 326.0605689(266.9987322 to 392.4325664) | 179.7502038(141.6213791 to 204.3779933) | 2661.032358(2193.444061 to 3000.398702) |
| Australasia | Female | 2018 | 324.2572794(264.816311 to 390.8231279) | 175.7716297(137.8831701 to 200.3091464) | 2612.003271(2144.877163 to 2953.934915) |
| Australasia | Female | 2019 | 322.9293925(261.916141 to 392.3385785) | 175.2698661(137.3123257 to 200.2013321) | 2598.609352(2142.776917 to 2944.957796) |
| Australasia | Female | 2020 | 323.5015097(259.2166044 to 399.3120767) | 160.9417856(123.9690854 to 185.3429635) | 2428.466115(1979.917856 to 2759.935013) |
| Australasia | Female | 2021 | 323.9081111(259.4816321 to 399.1919259) | 165.7243161(128.7758153 to 190.2191798) | 2479.506118(2033.272942 to 2812.806688) |
| Caribbean | Female | 1990 | 637.3045968(520.569565 to 774.0143321) | 708.3634269(625.868633 to 780.4834817) | 11832.25458(10464.21949 to 13124.09401) |
| Caribbean | Female | 1991 | 631.1800575(516.4661768 to 761.4688532) | 683.0931618(601.8233193 to 754.1835083) | 11434.93909(10068.27141 to 12706.20486) |
| Caribbean | Female | 1992 | 625.1204369(512.601478 to 751.7029107) | 684.3684757(603.796593 to 756.9047911) | 11443.01325(10093.86372 to 12747.64501) |
| Caribbean | Female | 1993 | 619.0849093(509.1643942 to 744.8619594) | 687.1543594(605.946706 to 761.060535) | 11523.86662(10151.56884 to 12882.22888) |
| Caribbean | Female | 1994 | 613.3539647(503.8363082 to 738.6262218) | 669.0458986(588.0320561 to 740.436771) | 11253.56367(9898.986301 to 12543.21124) |
| Caribbean | Female | 1995 | 608.1807598(498.6867926 to 732.2510351) | 659.4588855(580.307303 to 730.2911193) | 11103.07639(9780.953086 to 12378.03555) |
| Caribbean | Female | 1996 | 602.7184697(495.3524286 to 725.5466069) | 641.910685(564.7009114 to 711.997796) | 10827.92632(9527.421691 to 12079.16855) |
| Caribbean | Female | 1997 | 596.1738809(490.6512919 to 717.6290885) | 618.7472763(540.9155846 to 689.3389702) | 10511.36729(9214.30422 to 11760.28325) |
| Caribbean | Female | 1998 | 589.1765049(484.9985876 to 709.7713873) | 608.7658864(532.2327369 to 677.81108) | 10391.80115(9119.447052 to 11612.2762) |
| Caribbean | Female | 1999 | 582.0203423(478.4235021 to 702.3710206) | 600.3085222(523.7969802 to 665.6970377) | 10244.36577(8962.07569 to 11413.92523) |
| Caribbean | Female | 2000 | 575.3150711(471.8392336 to 696.1312112) | 590.4365837(513.4371826 to 655.5357205) | 10039.3758(8770.33814 to 11204.36754) |
| Caribbean | Female | 2001 | 568.7226006(467.0029062 to 686.7167379) | 582.8756094(507.3903438 to 648.8089882) | 9941.287224(8678.780837 to 11132.27421) |
| Caribbean | Female | 2002 | 562.062385(461.8581398 to 676.4530189) | 561.1187351(486.4952962 to 628.4982613) | 9584.024736(8330.678702 to 10771.0556) |
| Caribbean | Female | 2003 | 555.2635257(457.0241095 to 668.9075665) | 569.0990076(492.9727934 to 638.4324404) | 9650.5732(8394.293049 to 10867.24983) |
| Caribbean | Female | 2004 | 548.6319948(451.2044121 to 661.6935561) | 574.6259223(497.5982771 to 644.5821983) | 9678.350171(8403.954037 to 10910.31315) |
| Caribbean | Female | 2005 | 542.3106204(445.0380166 to 655.4461849) | 566.7057447(491.7115607 to 638.0076765) | 9561.442532(8314.506921 to 10830.21694) |
| Caribbean | Female | 2006 | 535.7525077(440.2366238 to 645.6806174) | 538.9015966(462.700328 to 610.5101565) | 9133.314988(7872.860968 to 10400.739) |
| Caribbean | Female | 2007 | 528.7685059(433.2249083 to 637.5016777) | 532.5605776(455.7176328 to 604.515915) | 9004.092093(7737.468625 to 10279.33642) |
| Caribbean | Female | 2008 | 521.7548557(426.7742137 to 628.7562206) | 536.1493892(461.9268732 to 608.89275) | 9021.12694(7796.998112 to 10313.11441) |
| Caribbean | Female | 2009 | 515.4950994(420.5605659 to 621.8111242) | 531.1365151(458.3342618 to 600.5410325) | 8898.725922(7702.553024 to 10130.42049) |
| Caribbean | Female | 2010 | 509.8931658(414.0783239 to 615.4236607) | 519.2157521(448.2843536 to 586.092108) | 8701.012892(7543.308681 to 9880.992925) |
| Caribbean | Female | 2011 | 505.7759108(412.1325123 to 609.7014203) | 494.1747272(424.4360056 to 559.7788713) | 8314.641263(7197.541078 to 9480.541081) |
| Caribbean | Female | 2012 | 502.6723035(410.4750909 to 605.8547635) | 483.4528613(414.7661333 to 546.7472552) | 8172.771334(7050.14247 to 9303.00935) |
| Caribbean | Female | 2013 | 500.070007(408.0694943 to 604.2237428) | 481.9947296(412.5294049 to 545.8056332) | 8155.672243(7020.85765 to 9300.304448) |
| Caribbean | Female | 2014 | 498.2327995(406.0433694 to 602.1078896) | 476.2876308(408.0612367 to 541.1440456) | 8087.71407(6964.281643 to 9253.295284) |
| Caribbean | Female | 2015 | 497.3593972(404.9802739 to 601.9125579) | 470.6767024(402.6466603 to 535.8122338) | 8008.645821(6891.704399 to 9184.904155) |
| Caribbean | Female | 2016 | 497.269127(405.8007532 to 600.5454424) | 462.3548711(395.9542826 to 527.9352919) | 7903.648732(6812.80734 to 9086.214499) |
| Caribbean | Female | 2017 | 497.2965786(406.7056041 to 601.356404) | 459.6204447(393.4965437 to 524.1686103) | 7888.936184(6793.318402 to 9044.203166) |
| Caribbean | Female | 2018 | 497.2026483(406.8178113 to 602.826306) | 454.5026341(388.1197816 to 518.0124892) | 7831.03849(6711.51982 to 8973.407619) |
| Caribbean | Female | 2019 | 496.8429336(406.1569393 to 603.7467767) | 452.1736298(384.9349089 to 515.2223283) | 7800.888041(6680.294478 to 8941.126319) |
| Caribbean | Female | 2020 | 490.1487353(400.8390905 to 593.8796597) | 446.8029418(379.7520529 to 510.5597467) | 7701.570138(6574.920837 to 8842.81023) |
| Caribbean | Female | 2021 | 491.3540205(400.8659692 to 597.8828893) | 442.5384705(373.8855971 to 510.0515803) | 7628.889913(6457.704307 to 8816.38198) |
| Central Asia | Female | 1990 | 1194.366804(975.1075952 to 1445.794636) | 1004.048657(919.0954376 to 1059.400154) | 17534.56131(16287.05145 to 18491.63004) |
| Central Asia | Female | 1991 | 1184.969371(972.0965042 to 1422.675691) | 1046.012083(960.8964436 to 1101.897718) | 18235.59562(16995.01153 to 19201.44624) |
| Central Asia | Female | 1992 | 1179.12426(970.6099069 to 1411.584244) | 1101.815715(1013.528334 to 1157.301574) | 19190.69929(17919.53991 to 20122.89783) |
| Central Asia | Female | 1993 | 1177.337589(973.2117164 to 1405.352761) | 1175.008848(1085.195941 to 1233.913597) | 20491.97601(19198.53394 to 21479.8423) |
| Central Asia | Female | 1994 | 1179.285518(977.1605424 to 1409.509972) | 1214.429087(1123.418865 to 1273.673617) | 21276.06598(19960.29211 to 22253.94333) |
| Central Asia | Female | 1995 | 1184.280586(982.2553077 to 1421.357719) | 1202.116112(1113.970581 to 1263.998811) | 21121.09701(19840.44765 to 22164.08152) |
| Central Asia | Female | 1996 | 1196.867073(993.864453 to 1431.221932) | 1180.407009(1090.354309 to 1244.581205) | 20753.33275(19440.39371 to 21803.54319) |
| Central Asia | Female | 1997 | 1217.959027(1011.507906 to 1452.236039) | 1169.589938(1081.390505 to 1234.433331) | 20562.37509(19279.63366 to 21626.2095) |
| Central Asia | Female | 1998 | 1242.504303(1031.896084 to 1482.43124) | 1158.820535(1069.878864 to 1224.787112) | 20397.27651(19100.59748 to 21469.63284) |
| Central Asia | Female | 1999 | 1264.568879(1047.352697 to 1512.993196) | 1143.786722(1051.408904 to 1206.925383) | 20133.49966(18817.74841 to 21200.54152) |
| Central Asia | Female | 2000 | 1278.489583(1057.078232 to 1530.337443) | 1154.913886(1061.967721 to 1221.921785) | 20300.81461(18974.86064 to 21373.01079) |
| Central Asia | Female | 2001 | 1282.965004(1061.806416 to 1531.66333) | 1137.599739(1046.699788 to 1202.903642) | 20057.26949(18747.25452 to 21135.12782) |
| Central Asia | Female | 2002 | 1282.915922(1063.530429 to 1531.3089) | 1142.733286(1052.414152 to 1206.005595) | 20083.19567(18796.59654 to 21148.00842) |
| Central Asia | Female | 2003 | 1279.443392(1061.780886 to 1525.998469) | 1173.073079(1080.425882 to 1239.230977) | 20495.92866(19173.35069 to 21606.07065) |
| Central Asia | Female | 2004 | 1274.242222(1057.271922 to 1516.652652) | 1172.959933(1076.856308 to 1239.497729) | 20385.88916(19045.61153 to 21493.47271) |
| Central Asia | Female | 2005 | 1269.379942(1052.393258 to 1513.547919) | 1159.461731(1063.468654 to 1226.262192) | 20196.25263(18798.4801 to 21344.95538) |
| Central Asia | Female | 2006 | 1265.174345(1052.323598 to 1506.932091) | 1095.424747(999.3572816 to 1165.863538) | 19137.84714(17716.69866 to 20294.35306) |
| Central Asia | Female | 2007 | 1261.281604(1048.56904 to 1500.138654) | 1072.371941(977.3497154 to 1143.366665) | 18691.62331(17292.14523 to 19870.47833) |
| Central Asia | Female | 2008 | 1255.828698(1041.338312 to 1493.694564) | 1068.254279(974.6667165 to 1137.89279) | 18549.8642(17192.7543 to 19708.47567) |
| Central Asia | Female | 2009 | 1248.451698(1031.987748 to 1485.525084) | 1067.659448(972.7029652 to 1135.386096) | 18393.7449(17053.40714 to 19518.93207) |
| Central Asia | Female | 2010 | 1238.7731(1019.798462 to 1476.352614) | 1072.383952(977.5576778 to 1140.63263) | 18270.01775(16929.097 to 19395.29506) |
| Central Asia | Female | 2011 | 1222.997093(1008.287421 to 1456.457681) | 1005.519255(919.8473029 to 1066.515052) | 17065.20019(15842.16989 to 18089.15098) |
| Central Asia | Female | 2012 | 1201.465129(992.6887759 to 1433.943869) | 954.0493344(872.3528651 to 1013.119649) | 16215.31414(15044.86347 to 17178.81567) |
| Central Asia | Female | 2013 | 1178.996153(975.9256717 to 1409.296256) | 897.7049244(818.8949643 to 954.0623436) | 15382.90563(14291.34558 to 16327.8483) |
| Central Asia | Female | 2014 | 1161.927894(960.2747975 to 1394.367435) | 880.0668172(804.6121568 to 935.066517) | 15075.69673(13987.17335 to 15982.13305) |
| Central Asia | Female | 2015 | 1155.696722(954.0664626 to 1391.353503) | 867.8896463(792.9080298 to 920.9504001) | 14791.46315(13717.80696 to 15681.63477) |
| Central Asia | Female | 2016 | 1160.22473(959.4638239 to 1393.623659) | 868.5726567(794.0385627 to 923.6755011) | 14734.05298(13681.03404 to 15621.64454) |
| Central Asia | Female | 2017 | 1169.017329(964.367653 to 1402.165405) | 866.7106066(790.4294285 to 922.8718849) | 14581.99591(13512.52739 to 15481.93516) |
| Central Asia | Female | 2018 | 1177.675285(970.0465114 to 1411.914327) | 844.8152042(767.1245939 to 899.8383902) | 14201.24536(13130.86555 to 15104.6325) |
| Central Asia | Female | 2019 | 1182.32636(973.211125 to 1415.879457) | 817.4906065(738.6320132 to 873.2430897) | 13742.94797(12648.79937 to 14657.118) |
| Central Asia | Female | 2020 | 1135.859712(930.1325716 to 1366.807697) | 821.7612366(738.4010836 to 887.4359513) | 13759.81544(12563.06136 to 14817.43265) |
| Central Asia | Female | 2021 | 1132.992404(927.1464313 to 1360.235591) | 789.2488185(697.3789176 to 870.7895399) | 13369.14619(11973.64296 to 14672.77483) |
| Central Europe | Female | 1990 | 1176.038415(945.4550305 to 1436.30489) | 1360.185106(1262.917223 to 1419.78497) | 20952.95228(19723.92303 to 21855.49135) |
| Central Europe | Female | 1991 | 1166.147974(939.7175365 to 1419.311955) | 1366.465991(1268.668021 to 1428.746583) | 21022.22966(19785.60283 to 21949.89117) |
| Central Europe | Female | 1992 | 1155.010728(931.975255 to 1401.283947) | 1356.84132(1258.555032 to 1418.926863) | 20872.429(19630.0918 to 21830.35014) |
| Central Europe | Female | 1993 | 1142.547563(924.3754199 to 1382.687211) | 1357.152513(1256.421698 to 1421.17264) | 20829.00008(19536.50171 to 21790.80009) |
| Central Europe | Female | 1994 | 1129.137243(914.8713734 to 1362.850738) | 1350.186846(1248.681736 to 1413.229178) | 20713.77895(19426.13013 to 21671.82117) |
| Central Europe | Female | 1995 | 1115.260577(904.7319992 to 1344.350176) | 1346.239171(1248.527591 to 1406.856027) | 20681.90177(19432.48734 to 21584.48245) |
| Central Europe | Female | 1996 | 1097.919091(893.858512 to 1318.691928) | 1328.851909(1230.389826 to 1388.025451) | 20427.94948(19184.80791 to 21312.01274) |
| Central Europe | Female | 1997 | 1075.704248(878.3282742 to 1287.882483) | 1309.689017(1212.732259 to 1368.582495) | 20173.35437(18935.68977 to 21061.47102) |
| Central Europe | Female | 1998 | 1051.32033(860.5771806 to 1254.028536) | 1250.828786(1157.230569 to 1307.659267) | 19259.08891(18057.58186 to 20115.00616) |
| Central Europe | Female | 1999 | 1027.719349(843.8475764 to 1223.332914) | 1227.799912(1135.478088 to 1283.672544) | 18894.96739(17701.7007 to 19744.01008) |
| Central Europe | Female | 2000 | 1007.909247(828.1391462 to 1200.76522) | 1168.196939(1079.235321 to 1224.072471) | 18006.64715(16842.62846 to 18846.06941) |
| Central Europe | Female | 2001 | 991.0654858(814.2325735 to 1180.999233) | 1133.745875(1046.219055 to 1188.565773) | 17439.59183(16309.49505 to 18271.52069) |
| Central Europe | Female | 2002 | 974.1053851(799.8376305 to 1160.239279) | 1109.151308(1021.956414 to 1164.10339) | 16983.6728(15866.78598 to 17795.27102) |
| Central Europe | Female | 2003 | 957.1460404(783.424035 to 1141.021283) | 1087.455888(1002.652447 to 1139.760027) | 16573.05692(15485.35464 to 17355.09775) |
| Central Europe | Female | 2004 | 940.4298914(767.8946477 to 1122.277241) | 1031.613101(946.9501739 to 1082.964221) | 15732.81359(14678.86545 to 16493.84749) |
| Central Europe | Female | 2005 | 923.6143546(752.3831025 to 1102.450692) | 997.1986791(913.6195223 to 1046.547942) | 15238.39043(14183.43868 to 15976.72536) |
| Central Europe | Female | 2006 | 905.2470453(738.192775 to 1079.610657) | 948.3030932(866.0924092 to 996.6325739) | 14502.7697(13462.72871 to 15217.45748) |
| Central Europe | Female | 2007 | 885.265458(721.7292482 to 1056.119061) | 910.8253858(831.9520686 to 957.9254939) | 13873.77053(12842.83556 to 14556.3504) |
| Central Europe | Female | 2008 | 865.3317411(705.9466518 to 1031.775025) | 876.6419148(798.0522072 to 921.8338617) | 13295.16768(12291.11178 to 13962.23358) |
| Central Europe | Female | 2009 | 846.6707499(691.7903522 to 1009.695014) | 852.5698234(774.8209164 to 897.5256132) | 12883.29476(11908.89699 to 13539.3682) |
| Central Europe | Female | 2010 | 829.899915(677.6924265 to 991.4700316) | 814.6815339(738.5338883 to 858.6360688) | 12311.50869(11350.8387 to 12958.54729) |
| Central Europe | Female | 2011 | 812.5963565(663.1378572 to 970.2631786) | 775.45532(700.3930818 to 818.0433271) | 11696.99503(10749.18477 to 12332.28456) |
| Central Europe | Female | 2012 | 793.8204257(647.7045822 to 948.8141137) | 755.5411503(682.8359081 to 797.2147689) | 11347.6404(10414.2992 to 11969.12999) |
| Central Europe | Female | 2013 | 776.6729333(634.2789922 to 927.8283682) | 723.2258228(651.9114891 to 764.8901757) | 10825.19305(9913.531468 to 11447.72374) |
| Central Europe | Female | 2014 | 763.6726881(624.5827194 to 912.0605444) | 705.8674373(636.4271452 to 747.1548337) | 10544.46847(9658.703879 to 11158.77354) |
| Central Europe | Female | 2015 | 757.4906687(619.8083267 to 904.4060838) | 684.9360202(617.7485386 to 725.3131837) | 10231.42812(9380.684319 to 10838.30778) |
| Central Europe | Female | 2016 | 758.1621067(620.8043252 to 904.6730987) | 653.4930988(586.8926607 to 693.8748587) | 9781.25271(8939.310437 to 10394.20071) |
| Central Europe | Female | 2017 | 761.9391542(623.0788269 to 908.8746159) | 643.5327584(577.026967 to 683.9020683) | 9629.751895(8800.374678 to 10223.10396) |
| Central Europe | Female | 2018 | 765.8807836(624.3296946 to 916.0808275) | 628.9143575(560.9803584 to 668.7506066) | 9414.582677(8577.708911 to 10006.96564) |
| Central Europe | Female | 2019 | 767.1015079(623.4610438 to 920.1864744) | 606.6117137(540.2137739 to 646.1133737) | 9099.573389(8276.265861 to 9692.140898) |
| Central Europe | Female | 2020 | 748.5529937(605.9170038 to 905.5496296) | 598.4448075(532.8172351 to 643.970797) | 8995.148318(8155.329926 to 9653.992757) |
| Central Europe | Female | 2021 | 749.1183805(607.5163438 to 906.4004969) | 597.5612437(526.02365 to 649.5634059) | 9031.668422(8097.088456 to 9769.700395) |
| Central Latin America | Female | 1990 | 640.7906385(505.2657112 to 806.4225996) | 485.0407657(444.9273031 to 510.7601133) | 7807.83179(7284.269872 to 8206.279098) |
| Central Latin America | Female | 1991 | 636.9235521(504.8036168 to 799.2729259) | 474.2801892(434.3663328 to 499.3191796) | 7666.382426(7148.098056 to 8055.569162) |
| Central Latin America | Female | 1992 | 631.7161628(502.1822221 to 789.0025449) | 467.5311999(427.5933939 to 492.7451316) | 7591.11891(7080.172946 to 7982.30672) |
| Central Latin America | Female | 1993 | 625.4210881(497.9163735 to 778.4235955) | 454.0205376(414.9733274 to 479.0735486) | 7409.886666(6905.687815 to 7789.411472) |
| Central Latin America | Female | 1994 | 618.1794862(492.042828 to 767.9079047) | 457.0853154(417.8468613 to 481.8498924) | 7469.021039(6955.694607 to 7852.606504) |
| Central Latin America | Female | 1995 | 610.2154546(486.7593038 to 757.6881814) | 453.17672(413.8133616 to 478.1763554) | 7362.205353(6859.32651 to 7744.113017) |
| Central Latin America | Female | 1996 | 599.684108(479.2005457 to 743.4395092) | 445.5225585(406.645253 to 470.7067531) | 7215.909426(6717.036542 to 7598.50497) |
| Central Latin America | Female | 1997 | 585.8675637(468.7990912 to 724.8855314) | 425.3164173(387.0300178 to 449.6374968) | 6954.251527(6460.351501 to 7323.717877) |
| Central Latin America | Female | 1998 | 570.469933(457.3945822 to 703.8868747) | 399.3905047(363.0360925 to 422.0871182) | 6626.511078(6148.316817 to 6979.379003) |
| Central Latin America | Female | 1999 | 555.0516163(445.1413915 to 684.2098176) | 385.4172561(349.5910974 to 408.1531828) | 6424.881147(5952.466777 to 6770.506119) |
| Central Latin America | Female | 2000 | 541.1661111(433.3641237 to 667.0019668) | 364.8331047(330.9105565 to 386.9023113) | 6106.356014(5660.750944 to 6449.231588) |
| Central Latin America | Female | 2001 | 528.3524109(423.1537728 to 651.9430202) | 352.9075303(319.6800842 to 374.418803) | 5901.853014(5469.138506 to 6239.19007) |
| Central Latin America | Female | 2002 | 515.3108212(413.2207834 to 635.5832857) | 345.6563607(312.9027243 to 367.2258419) | 5773.507649(5345.179654 to 6103.250659) |
| Central Latin America | Female | 2003 | 502.2534294(402.6360193 to 620.1812994) | 336.1920108(303.8136968 to 357.2497063) | 5607.178521(5184.51712 to 5925.675762) |
| Central Latin America | Female | 2004 | 489.4280925(392.1812876 to 604.6503148) | 321.0589218(289.4839517 to 341.3946968) | 5358.910283(4947.345342 to 5671.676649) |
| Central Latin America | Female | 2005 | 477.0458442(381.5858742 to 589.9959114) | 312.6724304(281.977936 to 332.8397725) | 5210.142332(4811.513947 to 5515.463717) |
| Central Latin America | Female | 2006 | 463.9377123(372.468236 to 572.3689466) | 303.0997113(272.8388387 to 323.0287214) | 5055.260939(4665.486366 to 5352.216413) |
| Central Latin America | Female | 2007 | 449.782229(361.5448785 to 554.7466033) | 296.0593241(266.5236391 to 315.5539865) | 4936.642713(4547.221692 to 5238.349105) |
| Central Latin America | Female | 2008 | 436.0797619(350.6141403 to 537.1678177) | 295.3997542(265.8764835 to 314.8618743) | 4905.705749(4514.469405 to 5212.028006) |
| Central Latin America | Female | 2009 | 424.3817427(341.1340381 to 522.7348242) | 295.0184956(265.7232562 to 314.7031282) | 4872.802629(4485.506879 to 5179.193175) |
| Central Latin America | Female | 2010 | 416.1508292(334.7773809 to 512.6723123) | 285.8101654(256.7418009 to 305.0217026) | 4724.035109(4336.423531 to 5028.951244) |
| Central Latin America | Female | 2011 | 410.2131931(330.4227801 to 503.8650917) | 274.1630442(245.2853158 to 293.0332428) | 4536.165946(4159.215392 to 4835.122846) |
| Central Latin America | Female | 2012 | 404.758616(326.0826008 to 495.9634065) | 267.6391193(239.2153196 to 285.9262741) | 4442.501397(4065.547977 to 4733.961107) |
| Central Latin America | Female | 2013 | 400.2624995(322.8340282 to 489.3778709) | 266.4219924(238.5297629 to 284.7627152) | 4414.898055(4041.691565 to 4706.732373) |
| Central Latin America | Female | 2014 | 397.177005(320.6385287 to 485.3972255) | 263.9124243(236.633634 to 282.0721321) | 4362.07955(3992.45601 to 4648.60103) |
| Central Latin America | Female | 2015 | 395.9268408(319.4807301 to 483.5555604) | 258.7473162(231.8744878 to 276.6077721) | 4272.974296(3910.207395 to 4553.902222) |
| Central Latin America | Female | 2016 | 397.0755971(320.629218 to 484.7031612) | 255.1573345(228.7088303 to 272.8500286) | 4222.295204(3863.349689 to 4501.696965) |
| Central Latin America | Female | 2017 | 399.8681614(322.5167444 to 488.6888417) | 245.8211574(219.802936 to 262.9698212) | 4082.802297(3733.590173 to 4355.377286) |
| Central Latin America | Female | 2018 | 402.7549624(324.6798981 to 493.4439026) | 241.3434323(215.6313246 to 258.6525683) | 4022.468803(3678.684112 to 4299.277821) |
| Central Latin America | Female | 2019 | 404.1579358(325.7657648 to 497.2221511) | 238.2391556(212.3205994 to 256.822431) | 3979.193827(3628.459459 to 4274.466775) |
| Central Latin America | Female | 2020 | 398.2227244(319.0508516 to 496.3077705) | 230.9988096(201.3018479 to 254.7651056) | 3913.779981(3493.773839 to 4277.830399) |
| Central Latin America | Female | 2021 | 398.7839174(319.7355149 to 494.2909455) | 232.0563162(198.838075 to 262.4533245) | 3928.480673(3443.918664 to 4415.703441) |
| Central Sub-Saharan Africa | Female | 1990 | 1176.447943(929.7643002 to 1470.799349) | 1110.801656(793.4410911 to 1473.803973) | 20182.93858(14649.31674 to 26475.87103) |
| Central Sub-Saharan Africa | Female | 1991 | 1167.627073(926.8463792 to 1449.887079) | 1105.942337(787.6253566 to 1479.870544) | 20104.25488(14594.27939 to 26566.53442) |
| Central Sub-Saharan Africa | Female | 1992 | 1159.128915(925.9316737 to 1434.290558) | 1098.437285(782.6887559 to 1475.471878) | 19958.50471(14528.11884 to 26479.58361) |
| Central Sub-Saharan Africa | Female | 1993 | 1151.189607(923.6357888 to 1419.001548) | 1097.700535(786.0316302 to 1471.464056) | 19933.28032(14546.47528 to 26467.83716) |
| Central Sub-Saharan Africa | Female | 1994 | 1144.023662(916.6682298 to 1407.24962) | 1104.094588(794.1193747 to 1481.082587) | 20034.51383(14643.8017 to 26561.1187) |
| Central Sub-Saharan Africa | Female | 1995 | 1137.849838(910.1825047 to 1401.336057) | 1105.887671(793.4656762 to 1480.80435) | 20064.60141(14627.89824 to 26556.29724) |
| Central Sub-Saharan Africa | Female | 1996 | 1132.305168(908.3048382 to 1393.375768) | 1126.305106(802.3542712 to 1507.685284) | 20435.81559(14762.46433 to 26994.15473) |
| Central Sub-Saharan Africa | Female | 1997 | 1126.624179(905.6276675 to 1384.806425) | 1093.945163(778.9959875 to 1457.725231) | 19824.62886(14343.26156 to 26072.61338) |
| Central Sub-Saharan Africa | Female | 1998 | 1120.79176(900.9327928 to 1379.395297) | 1093.023719(775.8655721 to 1463.955919) | 19804.10937(14263.487 to 26277.59202) |
| Central Sub-Saharan Africa | Female | 1999 | 1114.827388(893.284132 to 1379.107083) | 1085.83804(767.3059724 to 1464.120324) | 19666.50839(14105.1062 to 26223.71509) |
| Central Sub-Saharan Africa | Female | 2000 | 1108.696524(881.2172366 to 1375.816502) | 1087.675916(768.059818 to 1470.512334) | 19686.38298(14169.33336 to 26376.59755) |
| Central Sub-Saharan Africa | Female | 2001 | 1101.206128(881.3462247 to 1363.747252) | 1085.472363(765.3744155 to 1476.404589) | 19639.09753(14130.42992 to 26350.12266) |
| Central Sub-Saharan Africa | Female | 2002 | 1091.979122(875.8039682 to 1352.941868) | 1079.814966(759.6634308 to 1466.052761) | 19517.30338(14002.10489 to 26172.26317) |
| Central Sub-Saharan Africa | Female | 2003 | 1082.177488(870.9157444 to 1342.888491) | 1087.571024(762.3277235 to 1475.269111) | 19641.91247(14046.83048 to 26297.75101) |
| Central Sub-Saharan Africa | Female | 2004 | 1073.011349(861.7565934 to 1335.384075) | 1082.477475(759.6110113 to 1469.488787) | 19529.68721(13968.38731 to 26136.64884) |
| Central Sub-Saharan Africa | Female | 2005 | 1065.632524(853.8222543 to 1332.867274) | 1067.654823(749.1792091 to 1457.886611) | 19245.51434(13781.49223 to 25844.9229) |
| Central Sub-Saharan Africa | Female | 2006 | 1059.227836(850.2671334 to 1317.151232) | 1063.465561(747.7654091 to 1454.686981) | 19152.52102(13751.74526 to 25752.90942) |
| Central Sub-Saharan Africa | Female | 2007 | 1052.33899(846.1091036 to 1302.185777) | 1055.0559(742.6725813 to 1438.040851) | 18985.32117(13673.30121 to 25458.21669) |
| Central Sub-Saharan Africa | Female | 2008 | 1045.36677(839.1476206 to 1292.660265) | 1056.100275(743.3031277 to 1437.302766) | 18982.6374(13708.75118 to 25478.46359) |
| Central Sub-Saharan Africa | Female | 2009 | 1038.724593(830.5736577 to 1286.869673) | 1041.494006(727.6531063 to 1423.678424) | 18708.73234(13466.31631 to 25163.74329) |
| Central Sub-Saharan Africa | Female | 2010 | 1032.849246(826.920322 to 1280.165467) | 1028.500403(716.9124113 to 1401.517082) | 18467.80742(13236.42826 to 24717.71807) |
| Central Sub-Saharan Africa | Female | 2011 | 1026.701347(823.8015914 to 1267.484762) | 1015.787181(703.5166627 to 1382.418118) | 18226.25854(12965.6574 to 24418.55174) |
| Central Sub-Saharan Africa | Female | 2012 | 1019.909468(818.9079414 to 1249.6366) | 1000.591069(695.7180224 to 1362.270569) | 17950.53427(12850.2884 to 24044.82547) |
| Central Sub-Saharan Africa | Female | 2013 | 1013.528794(815.4268243 to 1242.004175) | 985.8532369(688.9370921 to 1345.658468) | 17670.47201(12678.11919 to 23688.98148) |
| Central Sub-Saharan Africa | Female | 2014 | 1008.638236(810.2001393 to 1238.588629) | 970.9566139(683.0476823 to 1333.277195) | 17386.08492(12569.6397 to 23398.70957) |
| Central Sub-Saharan Africa | Female | 2015 | 1006.328817(804.6919108 to 1237.330325) | 962.0976085(677.0693351 to 1322.30658) | 17205.34948(12443.69181 to 23121.56857) |
| Central Sub-Saharan Africa | Female | 2016 | 1006.627353(807.7426552 to 1234.374278) | 953.7009814(674.8923794 to 1307.302945) | 17037.04784(12387.08675 to 22947.92407) |
| Central Sub-Saharan Africa | Female | 2017 | 1008.080246(811.5215738 to 1234.76058) | 948.6496014(664.1469377 to 1295.747245) | 16914.95288(12186.07333 to 22687.49215) |
| Central Sub-Saharan Africa | Female | 2018 | 1009.197368(810.7939526 to 1233.660445) | 945.4676623(661.8077087 to 1292.736218) | 16826.20678(12115.79556 to 22577.54019) |
| Central Sub-Saharan Africa | Female | 2019 | 1008.546932(808.9818023 to 1243.282939) | 945.0729922(652.9018207 to 1302.439346) | 16776.43129(11981.6198 to 22667.81389) |
| Central Sub-Saharan Africa | Female | 2020 | 986.9726339(785.7257988 to 1221.481679) | 949.0059701(653.6603024 to 1316.80277) | 16815.26015(11916.22904 to 22859.00373) |
| Central Sub-Saharan Africa | Female | 2021 | 1004.177456(795.9842636 to 1238.820762) | 949.1179973(653.0201343 to 1330.726872) | 16795.32894(11885.66163 to 23049.6736) |
| East Asia | Female | 1990 | 1205.978813(931.9457477 to 1532.671032) | 1476.961419(1235.171811 to 1743.614113) | 24351.62511(20610.86748 to 28596.09386) |
| East Asia | Female | 1991 | 1214.466014(943.0130073 to 1535.929956) | 1444.81379(1229.077378 to 1688.043988) | 23796.63706(20402.63971 to 27671.25779) |
| East Asia | Female | 1992 | 1221.205951(951.4960086 to 1536.155705) | 1422.942122(1201.940514 to 1691.905394) | 23405.71998(19947.02023 to 27687.93056) |
| East Asia | Female | 1993 | 1226.259827(960.4379594 to 1535.812695) | 1404.115381(1194.027235 to 1630.506927) | 23065.51212(19837.3297 to 26686.75171) |
| East Asia | Female | 1994 | 1229.700761(966.5568433 to 1534.289218) | 1391.260748(1188.297703 to 1622.709632) | 22800.3696(19735.72698 to 26491.21919) |
| East Asia | Female | 1995 | 1231.61614(970.1359286 to 1534.76699) | 1369.699004(1188.637304 to 1586.610672) | 22408.64371(19657.81302 to 25797.53373) |
| East Asia | Female | 1996 | 1230.14158(968.6602955 to 1533.700917) | 1347.765172(1164.618984 to 1564.392029) | 22007.88693(19292.90989 to 25389.04555) |
| East Asia | Female | 1997 | 1224.649021(963.4768048 to 1527.544643) | 1322.685095(1146.738256 to 1511.30879) | 21543.04198(18948.72839 to 24503.99271) |
| East Asia | Female | 1998 | 1217.104944(956.4380366 to 1519.694394) | 1313.310389(1133.389143 to 1505.70026) | 21331.94651(18704.2919 to 24287.40131) |
| East Asia | Female | 1999 | 1209.383232(949.2639888 to 1512.473068) | 1312.770747(1137.210373 to 1498.242543) | 21262.16202(18649.68549 to 24099.89171) |
| East Asia | Female | 2000 | 1203.216388(943.186474 to 1506.282969) | 1332.00904(1162.099433 to 1505.887346) | 21443.18074(18978.57563 to 24057.08213) |
| East Asia | Female | 2001 | 1200.222958(939.5280094 to 1502.381481) | 1343.149037(1169.700598 to 1517.994366) | 21498.03682(18943.62676 to 24170.01216) |
| East Asia | Female | 2002 | 1199.220665(937.9852487 to 1501.900642) | 1329.408008(1165.0865 to 1475.181271) | 21299.00411(18993.017 to 23572.8742) |
| East Asia | Female | 2003 | 1197.617696(936.8619599 to 1500.968775) | 1337.797077(1173.165391 to 1490.091654) | 21380.95809(19021.47422 to 23711.21265) |
| East Asia | Female | 2004 | 1193.05287(932.9370561 to 1497.33592) | 1339.28027(1184.018525 to 1483.295939) | 21321.00594(19091.0478 to 23533.1263) |
| East Asia | Female | 2005 | 1183.372486(924.5420269 to 1487.483015) | 1290.991078(1142.970105 to 1430.402636) | 20503.78507(18401.06565 to 22602.39244) |
| East Asia | Female | 2006 | 1163.706276(910.3964284 to 1462.675965) | 1163.57198(1019.344924 to 1280.844973) | 18659.57209(16676.58448 to 20418.30632) |
| East Asia | Female | 2007 | 1134.385877(889.9473628 to 1426.755459) | 1096.403071(957.6700144 to 1225.083743) | 17644.44064(15712.30645 to 19550.1497) |
| East Asia | Female | 2008 | 1100.876736(865.3701421 to 1385.205374) | 1076.907362(930.6237151 to 1206.728901) | 17291.4137(15261.5284 to 19230.13239) |
| East Asia | Female | 2009 | 1068.448205(840.869764 to 1343.784907) | 1060.086008(916.2980903 to 1186.383282) | 17020.67141(15013.21677 to 18943.79091) |
| East Asia | Female | 2010 | 1042.766276(821.4212861 to 1311.935131) | 1037.96834(893.5898594 to 1165.501177) | 16646.23464(14556.41084 to 18500.09126) |
| East Asia | Female | 2011 | 1020.333284(804.5756953 to 1283.231145) | 995.5119848(853.8764346 to 1121.20805) | 16018.42484(13996.49653 to 17868.52275) |
| East Asia | Female | 2012 | 997.0463819(785.9312992 to 1254.054764) | 924.8157531(781.6491058 to 1047.138266) | 15059.7999(13000.65299 to 16861.48534) |
| East Asia | Female | 2013 | 976.640156(769.7428397 to 1227.378917) | 882.4897773(742.1036834 to 1003.365052) | 14408.46609(12396.12369 to 16243.27842) |
| East Asia | Female | 2014 | 962.8134209(757.8050194 to 1209.128965) | 853.6402609(717.6155919 to 973.8853326) | 13938.70917(12035.57224 to 15855.1058) |
| East Asia | Female | 2015 | 959.2561501(754.058428 to 1204.757541) | 818.8783303(678.7456758 to 941.9009279) | 13455.15386(11444.91608 to 15372.2127) |
| East Asia | Female | 2016 | 976.6039337(768.3909706 to 1226.849903) | 808.2758238(662.3402348 to 944.8955678) | 13279.40614(11213.73577 to 15302.08758) |
| East Asia | Female | 2017 | 1011.704186(795.4160199 to 1272.122759) | 797.8051466(648.4996732 to 945.2249699) | 13095.14909(10903.74268 to 15263.86474) |
| East Asia | Female | 2018 | 1047.09082(822.9272605 to 1318.662284) | 788.4131426(634.6724795 to 938.8215575) | 12947.32665(10646.35368 to 15313.3398) |
| East Asia | Female | 2019 | 1065.25406(836.4385093 to 1345.36858) | 777.0881648(612.5269761 to 940.4394857) | 12767.52881(10240.83506 to 15260.63393) |
| East Asia | Female | 2020 | 1028.852968(795.2352208 to 1315.044387) | 766.4485918(606.8807762 to 940.1850228) | 12596.70247(10316.82584 to 15238.81245) |
| East Asia | Female | 2021 | 1037.262986(801.3729614 to 1331.938922) | 752.9815158(584.2205033 to 921.448544) | 12452.36311(9978.296183 to 15016.50706) |
| Eastern Europe | Female | 1990 | 1326.987766(975.037966 to 1765.647758) | 1447.57095(1340.488582 to 1503.138289) | 22365.85058(20964.68417 to 23200.06127) |
| Eastern Europe | Female | 1991 | 1303.817905(964.2957337 to 1726.425685) | 1464.32398(1355.247655 to 1521.702109) | 22637.03403(21212.72851 to 23494.48984) |
| Eastern Europe | Female | 1992 | 1282.322433(954.2920716 to 1687.821558) | 1513.128657(1401.984454 to 1570.895468) | 23344.69713(21929.21811 to 24205.29245) |
| Eastern Europe | Female | 1993 | 1263.694747(949.0291204 to 1653.287167) | 1666.299905(1553.973911 to 1725.983199) | 25747.95442(24336.10032 to 26618.20927) |
| Eastern Europe | Female | 1994 | 1249.110844(943.8202488 to 1624.935216) | 1742.007896(1627.605734 to 1803.07225) | 27030.20726(25596.74101 to 27928.52469) |
| Eastern Europe | Female | 1995 | 1239.670599(936.2434085 to 1607.218017) | 1703.353433(1589.257608 to 1763.677789) | 26398.50657(24966.575 to 27278.35743) |
| Eastern Europe | Female | 1996 | 1235.12278(934.8143751 to 1602.174022) | 1634.051373(1521.377853 to 1692.677788) | 25330.01901(23917.46167 to 26199.87111) |
| Eastern Europe | Female | 1997 | 1232.142672(934.7085945 to 1599.618881) | 1603.355277(1492.20554 to 1661.596495) | 24770.97412(23364.1199 to 25616.89722) |
| Eastern Europe | Female | 1998 | 1229.66053(934.6187161 to 1594.639817) | 1591.325194(1479.483811 to 1649.106915) | 24436.89827(23024.00325 to 25281.71011) |
| Eastern Europe | Female | 1999 | 1226.251411(932.4903492 to 1589.133858) | 1644.808274(1533.636369 to 1701.485846) | 25181.43508(23793.6913 to 26016.46972) |
| Eastern Europe | Female | 2000 | 1219.69573(927.7037861 to 1581.421266) | 1655.495692(1543.592868 to 1712.066178) | 25306.97726(23912.51023 to 26149.42165) |
| Eastern Europe | Female | 2001 | 1208.217304(918.5090436 to 1566.08335) | 1658.496498(1546.804957 to 1716.445448) | 25350.56477(23959.2387 to 26194.18482) |
| Eastern Europe | Female | 2002 | 1191.64943(904.6946802 to 1544.454962) | 1682.419073(1572.544342 to 1740.119236) | 25555.87236(24173.07446 to 26397.74321) |
| Eastern Europe | Female | 2003 | 1171.232108(888.2276603 to 1516.857976) | 1660.850186(1552.4123 to 1716.42357) | 25241.50487(23887.43781 to 26053.28836) |
| Eastern Europe | Female | 2004 | 1149.975682(871.8406206 to 1488.438271) | 1546.175072(1441.373599 to 1599.962867) | 23751.95238(22441.0188 to 24549.99783) |
| Eastern Europe | Female | 2005 | 1127.635231(855.3579519 to 1460.095951) | 1506.068925(1403.632163 to 1559.328822) | 23313.4285(22017.39864 to 24085.8083) |
| Eastern Europe | Female | 2006 | 1098.794519(835.4006665 to 1419.292078) | 1367.29182(1267.944025 to 1417.520298) | 21170.6342(19918.2219 to 21912.10059) |
| Eastern Europe | Female | 2007 | 1061.710635(810.1125993 to 1369.302085) | 1277.552818(1182.972108 to 1326.402432) | 19743.24954(18555.12871 to 20476.49277) |
| Eastern Europe | Female | 2008 | 1022.861867(781.6816364 to 1318.60979) | 1229.209438(1137.411486 to 1276.386644) | 18997.40797(17841.54245 to 19699.78718) |
| Eastern Europe | Female | 2009 | 990.17273(758.6668803 to 1276.051102) | 1130.121471(1042.298646 to 1175.244796) | 17555.19306(16439.01444 to 18233.52207) |
| Eastern Europe | Female | 2010 | 968.6058676(742.199431 to 1249.238042) | 1090.103667(1006.06138 to 1134.162692) | 16938.32616(15868.65009 to 17598.34085) |
| Eastern Europe | Female | 2011 | 955.8390238(733.5760601 to 1230.073199) | 987.835461(906.8816491 to 1030.547211) | 15393.95541(14364.34277 to 16037.14215) |
| Eastern Europe | Female | 2012 | 945.4254106(725.7342385 to 1215.098778) | 932.8338812(854.1245263 to 974.0351961) | 14488.8136(13498.07303 to 15122.55474) |
| Eastern Europe | Female | 2013 | 938.169044(719.4124518 to 1205.252157) | 902.6763063(826.3215951 to 944.2180243) | 13938.51809(12972.53609 to 14557.71408) |
| Eastern Europe | Female | 2014 | 935.1504232(717.2830626 to 1200.3325) | 886.186325(810.8334368 to 927.5345702) | 13635.66616(12679.50357 to 14252.33841) |
| Eastern Europe | Female | 2015 | 935.5977545(716.4186462 to 1200.572771) | 862.895139(786.8871201 to 902.4570813) | 13225.82099(12260.10269 to 13833.19088) |
| Eastern Europe | Female | 2016 | 941.1440118(720.1938315 to 1209.880322) | 843.7502778(767.0854902 to 883.5020574) | 12884.19274(11928.79029 to 13490.80574) |
| Eastern Europe | Female | 2017 | 950.0360566(726.742621 to 1222.511701) | 800.8509539(723.9626128 to 844.5859749) | 12194.10674(11186.85073 to 12849.31189) |
| Eastern Europe | Female | 2018 | 958.209204(732.903422 to 1234.888633) | 794.4444031(711.1032155 to 844.07871) | 12077.07207(11003.9613 to 12813.16277) |
| Eastern Europe | Female | 2019 | 961.1567151(734.2710917 to 1240.748925) | 776.1997697(695.9416682 to 828.8780472) | 11795.66093(10753.67716 to 12575.01474) |
| Eastern Europe | Female | 2020 | 932.0937454(704.6185621 to 1212.718922) | 755.9045482(672.6306813 to 809.643972) | 11540.47072(10446.19697 to 12360.19364) |
| Eastern Europe | Female | 2021 | 920.840026(697.1643146 to 1197.119217) | 771.6608054(673.3155873 to 855.4147366) | 11857.95963(10493.0065 to 13087.34819) |
| Eastern Sub-Saharan Africa | Female | 1990 | 1131.602674(898.331062 to 1410.222685) | 1045.181162(855.8566016 to 1270.616231) | 19274.31273(16066.62988 to 23123.27278) |
| Eastern Sub-Saharan Africa | Female | 1991 | 1118.551805(891.5055772 to 1388.408433) | 1035.637775(849.9051886 to 1254.834925) | 19109.76219(15958.19557 to 22888.7418) |
| Eastern Sub-Saharan Africa | Female | 1992 | 1105.771993(885.6278476 to 1368.812476) | 1029.400141(843.4667333 to 1245.791581) | 18991.12039(15867.43339 to 22659.17177) |
| Eastern Sub-Saharan Africa | Female | 1993 | 1093.89499(879.0174555 to 1351.221317) | 1023.938877(839.304354 to 1233.980248) | 18892.67974(15801.14835 to 22456.93938) |
| Eastern Sub-Saharan Africa | Female | 1994 | 1082.563529(872.2603838 to 1332.824848) | 1015.481201(834.5716377 to 1228.482735) | 18725.29998(15680.86635 to 22350.7077) |
| Eastern Sub-Saharan Africa | Female | 1995 | 1072.743377(865.2684527 to 1318.382203) | 1010.411305(829.8263767 to 1222.902313) | 18602.44308(15585.16738 to 22249.20875) |
| Eastern Sub-Saharan Africa | Female | 1996 | 1065.128488(859.487006 to 1308.841289) | 998.9491316(823.3294225 to 1210.465806) | 18380.60317(15421.19441 to 21947.67016) |
| Eastern Sub-Saharan Africa | Female | 1997 | 1057.817771(852.8666869 to 1299.535727) | 989.487029(817.81994 to 1195.38563) | 18206.41353(15303.50858 to 21678.01499) |
| Eastern Sub-Saharan Africa | Female | 1998 | 1050.017059(845.372791 to 1291.443861) | 977.8278538(810.5467414 to 1178.738548) | 17987.79365(15177.03744 to 21329.37744) |
| Eastern Sub-Saharan Africa | Female | 1999 | 1041.629672(837.2846271 to 1282.351626) | 958.2146556(792.3292246 to 1153.432678) | 17618.524(14870.40777 to 20923.57239) |
| Eastern Sub-Saharan Africa | Female | 2000 | 1032.66251(829.348713 to 1273.027312) | 941.3068166(780.8791612 to 1129.54349) | 17282.54538(14596.14653 to 20448.25963) |
| Eastern Sub-Saharan Africa | Female | 2001 | 1021.528666(820.556686 to 1260.296766) | 910.9386205(752.3555311 to 1091.968571) | 16706.92565(14085.45424 to 19708.34406) |
| Eastern Sub-Saharan Africa | Female | 2002 | 1007.776538(810.4027937 to 1244.998155) | 896.1403054(738.0392519 to 1072.341642) | 16397.59395(13836.63559 to 19281.11556) |
| Eastern Sub-Saharan Africa | Female | 2003 | 993.3783172(799.2852306 to 1229.276515) | 881.4464576(732.0872942 to 1050.279361) | 16118.58209(13713.35189 to 18863.95138) |
| Eastern Sub-Saharan Africa | Female | 2004 | 980.0199004(787.9901777 to 1214.838441) | 871.9664336(724.7379649 to 1037.690082) | 15919.25894(13587.07336 to 18642.80018) |
| Eastern Sub-Saharan Africa | Female | 2005 | 969.2838718(777.9777751 to 1202.878709) | 859.2981593(716.6035684 to 1020.638506) | 15654.2159(13360.60303 to 18250.44909) |
| Eastern Sub-Saharan Africa | Female | 2006 | 961.5354164(773.6537219 to 1190.784664) | 851.3209739(703.6882515 to 1010.18716) | 15463.62792(13157.29178 to 17994.34899) |
| Eastern Sub-Saharan Africa | Female | 2007 | 955.5472527(769.3864693 to 1180.902153) | 841.2381368(697.0260669 to 997.0941813) | 15258.04959(13017.1191 to 17732.9397) |
| Eastern Sub-Saharan Africa | Female | 2008 | 950.7744727(766.5219449 to 1170.107036) | 838.3186727(692.5693769 to 991.7407668) | 15155.44228(12885.47197 to 17570.51255) |
| Eastern Sub-Saharan Africa | Female | 2009 | 947.0233177(763.8165646 to 1163.852986) | 837.6157311(692.0640203 to 990.2751412) | 15098.09891(12847.87582 to 17491.02769) |
| Eastern Sub-Saharan Africa | Female | 2010 | 944.3682347(761.9473089 to 1160.416597) | 830.5191355(687.0201271 to 984.9272816) | 14967.75705(12693.77174 to 17404.6267) |
| Eastern Sub-Saharan Africa | Female | 2011 | 940.7856576(759.8674117 to 1154.111246) | 821.4970056(677.0194255 to 974.3459982) | 14807.3603(12548.97823 to 17236.20901) |
| Eastern Sub-Saharan Africa | Female | 2012 | 936.8557887(756.8967387 to 1147.671624) | 811.9611638(667.7722568 to 966.0143436) | 14645.86914(12371.74838 to 17089.1315) |
| Eastern Sub-Saharan Africa | Female | 2013 | 933.463448(754.3841814 to 1142.095631) | 803.2063532(658.0548841 to 953.3534754) | 14488.30811(12256.99845 to 16885.34047) |
| Eastern Sub-Saharan Africa | Female | 2014 | 931.5938737(752.5613829 to 1140.140992) | 793.5073366(646.6550066 to 940.1327431) | 14323.99946(12023.05549 to 16690.0003) |
| Eastern Sub-Saharan Africa | Female | 2015 | 932.34153(751.1663984 to 1142.857262) | 786.8854017(639.2436065 to 937.6948209) | 14197.74832(11899.67658 to 16599.1304) |
| Eastern Sub-Saharan Africa | Female | 2016 | 936.5118808(756.7950887 to 1146.03704) | 777.4164093(632.9174921 to 922.3613674) | 14035.55394(11776.73296 to 16361.7917) |
| Eastern Sub-Saharan Africa | Female | 2017 | 943.0415754(760.6440537 to 1152.731433) | 772.9347558(622.7928173 to 921.9375381) | 13940.79998(11582.87702 to 16319.14643) |
| Eastern Sub-Saharan Africa | Female | 2018 | 949.047709(764.4814275 to 1161.331113) | 768.883865(620.4672414 to 915.8665483) | 13846.91525(11516.73123 to 16221.01828) |
| Eastern Sub-Saharan Africa | Female | 2019 | 951.7843088(764.9205561 to 1166.017678) | 765.1637977(613.7325996 to 908.511734) | 13761.17886(11420.63849 to 16034.92543) |
| Eastern Sub-Saharan Africa | Female | 2020 | 940.1228453(750.0025889 to 1161.294398) | 763.4770601(608.505178 to 913.9917499) | 13724.35916(11304.77349 to 16121.77448) |
| Eastern Sub-Saharan Africa | Female | 2021 | 949.1199142(758.3860374 to 1175.439241) | 756.6888156(603.7960935 to 907.4217174) | 13638.23505(11274.60242 to 16125.14042) |
| Global | Female | 1990 | 916.1916894(721.5638162 to 1148.245214) | 919.165911(824.4807794 to 991.0587698) | 15376.41268(14031.57204 to 16569.01071) |
| Global | Female | 1991 | 909.2082832(719.7256281 to 1134.153453) | 904.1609498(812.5657424 to 972.5761014) | 15151.26177(13840.47312 to 16219.08654) |
| Global | Female | 1992 | 901.6454576(716.7898869 to 1120.901156) | 897.072879(804.1088794 to 965.7797147) | 15049.22549(13750.54163 to 16147.19255) |
| Global | Female | 1993 | 894.0730081(713.980589 to 1107.107682) | 900.9228495(810.360249 to 969.0319663) | 15113.16531(13855.43277 to 16215.75219) |
| Global | Female | 1994 | 886.7957944(710.7056237 to 1094.2445) | 899.0169947(807.3998188 to 962.2986879) | 15095.05449(13813.2763 to 16116.74443) |
| Global | Female | 1995 | 880.130676(707.7546398 to 1082.575126) | 888.1113935(800.9133966 to 950.4775041) | 14907.92706(13706.24384 to 15891.05981) |
| Global | Female | 1996 | 872.5710942(702.7281568 to 1072.01533) | 868.3954074(782.4213045 to 929.6378002) | 14601.53246(13450.63925 to 15584.53055) |
| Global | Female | 1997 | 862.1058519(694.7824533 to 1058.615121) | 849.5493864(764.305119 to 907.4737189) | 14313.34145(13170.10899 to 15212.24386) |
| Global | Female | 1998 | 850.1724492(685.077691 to 1044.039989) | 836.7961569(753.948852 to 891.9517033) | 14111.6688(13007.3921 to 14985.8441) |
| Global | Female | 1999 | 838.184744(675.0320762 to 1029.628786) | 831.1671136(750.4750907 to 887.1260508) | 13998.69138(12901.77665 to 14876.53786) |
| Global | Female | 2000 | 826.8432635(665.4985734 to 1015.796504) | 819.5003637(738.383223 to 874.2965594) | 13807.80407(12721.75185 to 14627.58988) |
| Global | Female | 2001 | 816.0698236(656.7352635 to 1002.724877) | 811.0662008(731.8385799 to 865.9255865) | 13661.32404(12562.45378 to 14508.55501) |
| Global | Female | 2002 | 804.6363138(647.2338362 to 988.9916708) | 804.5395434(724.2561366 to 854.3977981) | 13547.89482(12469.56147 to 14336.22306) |
| Global | Female | 2003 | 792.6393277(637.59219 to 974.6088026) | 795.6196961(715.4543187 to 845.9347244) | 13395.08418(12305.30033 to 14181.91857) |
| Global | Female | 2004 | 780.6465722(627.9901094 to 960.4300301) | 771.7690149(694.1389559 to 821.4693795) | 13010.7755(11954.28703 to 13805.73942) |
| Global | Female | 2005 | 768.2302257(617.8399797 to 945.4398374) | 750.010305(675.0381714 to 798.0995473) | 12663.30596(11673.40242 to 13398.90824) |
| Global | Female | 2006 | 753.9131218(606.8538355 to 926.1039238) | 709.9589859(637.087339 to 754.9532536) | 12040.46025(11089.0269 to 12750.04486) |
| Global | Female | 2007 | 736.6833692(593.1009841 to 903.2965445) | 685.3558105(613.8468831 to 732.6509925) | 11624.09768(10675.93029 to 12343.80454) |
| Global | Female | 2008 | 718.7079292(578.717244 to 880.2702821) | 672.8106174(599.3401079 to 718.9584999) | 11381.11724(10409.17828 to 12070.80718) |
| Global | Female | 2009 | 702.9092786(566.3650695 to 860.6696826) | 655.1837553(583.281213 to 700.5705647) | 11088.36217(10143.06914 to 11785.29201) |
| Global | Female | 2010 | 690.8570388(557.0216391 to 846.2859521) | 640.0057915(570.0873352 to 684.2593106) | 10833.28375(9871.329498 to 11508.66276) |
| Global | Female | 2011 | 681.2724984(549.5308298 to 835.2188837) | 619.0337133(550.9846334 to 664.2521343) | 10506.47325(9587.691343 to 11203.65323) |
| Global | Female | 2012 | 671.4084634(541.5503402 to 823.8560517) | 598.0784234(528.9848155 to 643.5847539) | 10188.36312(9258.652196 to 10906.14332) |
| Global | Female | 2013 | 662.937583(534.752116 to 813.6265852) | 582.5444644(515.2739124 to 628.4745277) | 9900.117535(8989.783973 to 10631.0073) |
| Global | Female | 2014 | 657.7952259(530.2705378 to 807.5634004) | 571.4148856(504.8524789 to 616.1343983) | 9680.252391(8794.607971 to 10398.59373) |
| Global | Female | 2015 | 657.0077298(529.1565948 to 805.9715953) | 561.012116(491.8672719 to 607.1037964) | 9527.721268(8596.724746 to 10262.55927) |
| Global | Female | 2016 | 663.9895436(534.6046009 to 814.5667524) | 554.817018(487.3809135 to 603.7223235) | 9433.709131(8522.253881 to 10190.68395) |
| Global | Female | 2017 | 676.4059769(544.0590615 to 830.385126) | 547.474681(478.6673103 to 597.6338554) | 9317.705627(8374.870752 to 10081.29688) |
| Global | Female | 2018 | 688.7238381(552.8711279 to 846.8915052) | 541.1018509(473.1726194 to 592.2775635) | 9230.8904(8256.522269 to 10055.8021) |
| Global | Female | 2019 | 695.8613136(557.6936082 to 857.0784512) | 533.1772775(462.0845434 to 585.6728604) | 9109.033974(8098.404788 to 9959.910293) |
| Global | Female | 2020 | 679.9134002(542.1274554 to 845.6122397) | 525.3340139(456.048154 to 581.4980074) | 8982.853925(7999.048706 to 9840.682543) |
| Global | Female | 2021 | 683.6444101(544.9863882 to 853.1835689) | 520.2805585(448.6406752 to 577.8471035) | 8927.199956(7905.375116 to 9803.706617) |
| High-income Asia Pacific | Female | 1990 | 834.5601117(638.7257852 to 1078.490574) | 656.5228945(550.4040604 to 717.4449601) | 10419.33699(9107.228388 to 11302.91265) |
| High-income Asia Pacific | Female | 1991 | 832.7416119(638.3637553 to 1072.794588) | 615.9587261(516.5872274 to 673.0101702) | 9839.183495(8591.01885 to 10666.83722) |
| High-income Asia Pacific | Female | 1992 | 827.6624181(636.8542352 to 1061.142538) | 580.6361839(486.1790837 to 635.1748683) | 9352.310215(8174.543631 to 10154.46693) |
| High-income Asia Pacific | Female | 1993 | 819.6810061(633.9762465 to 1048.364223) | 555.048258(463.4107982 to 608.2016762) | 8978.168744(7850.125675 to 9756.883507) |
| High-income Asia Pacific | Female | 1994 | 810.0213692(627.595626 to 1033.240916) | 538.5553711(447.9332079 to 590.7672216) | 8709.69326(7588.358451 to 9490.574291) |
| High-income Asia Pacific | Female | 1995 | 799.0828511(620.404248 to 1015.662062) | 536.9179415(444.7353549 to 590.2976736) | 8652.871332(7533.447634 to 9429.328083) |
| High-income Asia Pacific | Female | 1996 | 775.4896703(605.1804292 to 982.5014407) | 501.9021614(412.4286451 to 553.391413) | 8146.317847(7049.509443 to 8911.431071) |
| High-income Asia Pacific | Female | 1997 | 734.3170605(576.1943983 to 928.4212134) | 468.9297883(383.1862208 to 519.0436741) | 7665.220235(6602.135361 to 8403.953698) |
| High-income Asia Pacific | Female | 1998 | 685.2658577(538.1874295 to 863.2354069) | 447.3062941(363.9507807 to 496.1517483) | 7333.48169(6306.80406 to 8058.801881) |
| High-income Asia Pacific | Female | 1999 | 638.4628594(502.2427946 to 801.3752642) | 426.9913318(347.3436076 to 474.069794) | 7026.701687(6045.439475 to 7725.056924) |
| High-income Asia Pacific | Female | 2000 | 602.950492(475.8043119 to 754.5556779) | 391.8468487(316.3791108 to 436.788079) | 6526.669387(5589.036085 to 7194.002268) |
| High-income Asia Pacific | Female | 2001 | 578.1815045(458.7851043 to 720.2630859) | 362.9348573(290.2580309 to 405.5860074) | 6086.316874(5191.307317 to 6734.250154) |
| High-income Asia Pacific | Female | 2002 | 555.9252044(443.2236449 to 689.3981699) | 342.7077361(272.5695522 to 384.296435) | 5773.397453(4897.515596 to 6411.023799) |
| High-income Asia Pacific | Female | 2003 | 535.2752063(428.35088 to 660.4941327) | 326.4667115(257.9353062 to 366.4344356) | 5506.874444(4654.474287 to 6122.482476) |
| High-income Asia Pacific | Female | 2004 | 515.7862977(413.3762418 to 633.9755923) | 311.6767312(244.6954436 to 350.6927501) | 5252.943253(4418.324458 to 5853.175833) |
| High-income Asia Pacific | Female | 2005 | 497.0072621(399.2652485 to 610.0514918) | 297.0887842(232.6564379 to 334.8476812) | 5021.587526(4209.583545 to 5606.02164) |
| High-income Asia Pacific | Female | 2006 | 477.3644812(383.844237 to 584.2313647) | 277.5595788(215.9842776 to 313.9788324) | 4716.453114(3937.675557 to 5276.637741) |
| High-income Asia Pacific | Female | 2007 | 456.2429522(367.9393152 to 557.868599) | 260.7145021(201.4245971 to 295.7794452) | 4440.39913(3694.6048 to 4984.773977) |
| High-income Asia Pacific | Female | 2008 | 435.8113422(351.8298293 to 532.865313) | 247.1295358(189.4892747 to 280.9932817) | 4212.946095(3499.749871 to 4729.79962) |
| High-income Asia Pacific | Female | 2009 | 418.0023977(337.600521 to 511.382157) | 234.0396101(178.4529396 to 266.7879805) | 4004.44878(3315.456276 to 4508.021886) |
| High-income Asia Pacific | Female | 2010 | 404.8117637(326.7143789 to 494.9704402) | 226.0740949(171.5830777 to 258.2153639) | 3869.610745(3198.711815 to 4362.288408) |
| High-income Asia Pacific | Female | 2011 | 394.4292909(319.2904124 to 481.1034617) | 222.0765153(168.3014127 to 253.6503927) | 3787.665429(3128.020639 to 4273.874163) |
| High-income Asia Pacific | Female | 2012 | 384.5768292(312.0196499 to 467.9346977) | 213.0534977(160.3372403 to 244.3012718) | 3638.157789(2993.145758 to 4113.400149) |
| High-income Asia Pacific | Female | 2013 | 376.0589093(305.4376895 to 457.047059) | 203.5060993(152.2329045 to 234.2318165) | 3489.197929(2856.064219 to 3955.681621) |
| High-income Asia Pacific | Female | 2014 | 370.0819602(300.2290301 to 449.9636822) | 193.7682306(143.6607517 to 223.4348996) | 3346.10688(2725.243414 to 3802.305882) |
| High-income Asia Pacific | Female | 2015 | 367.6838066(297.9313315 to 447.4491496) | 185.3476196(136.2337552 to 214.4757729) | 3227.753525(2617.357646 to 3682.12028) |
| High-income Asia Pacific | Female | 2016 | 369.7959747(299.9274557 to 449.9745338) | 178.8362184(130.895347 to 207.3269687) | 3137.601749(2537.062241 to 3581.253125) |
| High-income Asia Pacific | Female | 2017 | 374.9379895(303.2523909 to 456.8131854) | 172.493143(125.3235115 to 200.3320665) | 3047.004734(2460.26995 to 3482.511328) |
| High-income Asia Pacific | Female | 2018 | 380.0969373(306.4365051 to 463.4239567) | 168.468593(122.1778552 to 195.9486511) | 2987.694174(2407.960523 to 3420.984303) |
| High-income Asia Pacific | Female | 2019 | 382.4715456(307.7898458 to 467.4744074) | 164.1098657(118.7470556 to 191.8873885) | 2923.851519(2347.038795 to 3355.968484) |
| High-income Asia Pacific | Female | 2020 | 373.1888198(298.9112638 to 461.8468546) | 155.4513271(111.406995 to 182.8085223) | 2818.026936(2252.846452 to 3248.789655) |
| High-income Asia Pacific | Female | 2021 | 374.1992443(299.497225 to 462.1545818) | 160.0062649(115.2383854 to 187.7697335) | 2877.623894(2299.805019 to 3310.247309) |
| High-income North America | Female | 1990 | 576.0150749(419.7986946 to 770.5600121) | 291.7338649(245.5806298 to 316.4634163) | 4932.789617(4347.066554 to 5364.120915) |
| High-income North America | Female | 1991 | 555.2775273(408.4435741 to 737.156305) | 282.554054(237.1163296 to 306.428533) | 4811.544777(4233.54739 to 5235.175534) |
| High-income North America | Female | 1992 | 536.181884(397.0358243 to 707.3904056) | 279.0445623(234.1192222 to 302.7074812) | 4763.265227(4187.425959 to 5183.11608) |
| High-income North America | Female | 1993 | 519.5699273(387.6228548 to 682.5572654) | 285.0986852(239.767192 to 309.1260407) | 4841.123299(4261.857186 to 5267.162553) |
| High-income North America | Female | 1994 | 506.3144405(380.4709894 to 664.1067245) | 288.2705519(242.4878318 to 312.6639555) | 4882.369324(4299.084509 to 5305.060633) |
| High-income North America | Female | 1995 | 497.2327805(375.6073216 to 650.9429183) | 291.1692743(244.972125 to 316.0178817) | 4927.949824(4336.573371 to 5355.304087) |
| High-income North America | Female | 1996 | 491.4224714(372.4085705 to 641.1940634) | 290.083875(244.0626036 to 314.9057945) | 4918.979873(4322.12637 to 5346.2662) |
| High-income North America | Female | 1997 | 486.8547054(369.7977396 to 633.2971632) | 288.814968(242.8263811 to 313.4032057) | 4912.554326(4312.97185 to 5339.347129) |
| High-income North America | Female | 1998 | 482.761705(367.1355882 to 626.8114203) | 288.7767888(242.6491511 to 313.1588586) | 4915.190628(4312.988152 to 5344.775767) |
| High-income North America | Female | 1999 | 478.3919196(364.1558191 to 620.2475374) | 296.6637718(249.4693935 to 321.4390332) | 5010.482183(4396.766903 to 5451.006134) |
| High-income North America | Female | 2000 | 472.9591274(360.2374897 to 612.2920447) | 294.6039559(246.991317 to 319.5577766) | 4978.738439(4361.550391 to 5426.250289) |
| High-income North America | Female | 2001 | 464.9578756(354.4991736 to 601.6482644) | 289.6730936(242.9009797 to 314.7121032) | 4896.869514(4290.3735 to 5347.730991) |
| High-income North America | Female | 2002 | 454.2350184(346.547581 to 587.5799374) | 284.4758368(238.2526414 to 309.1639186) | 4808.859272(4209.486425 to 5253.198877) |
| High-income North America | Female | 2003 | 442.073625(337.6658574 to 571.1651516) | 273.1425652(228.5057715 to 297.1473098) | 4639.7938(4051.747714 to 5070.648593) |
| High-income North America | Female | 2004 | 429.8132605(328.7142027 to 554.3429356) | 258.1453094(214.9488734 to 281.5110764) | 4428.591638(3862.12817 to 4851.751903) |
| High-income North America | Female | 2005 | 418.7299798(320.6026332 to 539.2313944) | 246.7716157(204.9577329 to 268.9957578) | 4269.819205(3719.215111 to 4682.604799) |
| High-income North America | Female | 2006 | 407.5437271(312.9331741 to 523.8238163) | 236.4274406(195.8596885 to 257.9144519) | 4120.919475(3576.395932 to 4525.175075) |
| High-income North America | Female | 2007 | 395.271748(304.1054217 to 506.7884572) | 229.8574011(189.7544241 to 251.1755422) | 4001.373796(3467.559997 to 4400.555534) |
| High-income North America | Female | 2008 | 383.6952944(296.4049025 to 490.1059959) | 224.3795861(184.5812691 to 245.2710043) | 3899.796652(3372.068976 to 4287.321218) |
| High-income North America | Female | 2009 | 374.6646339(290.6643079 to 477.2459466) | 216.9761176(177.7894514 to 237.5278768) | 3779.168288(3264.906326 to 4160.517278) |
| High-income North America | Female | 2010 | 369.9405434(287.6349578 to 470.8171907) | 211.8458783(172.7489563 to 232.4716234) | 3692.257457(3185.163589 to 4070.856898) |
| High-income North America | Female | 2011 | 368.072836(286.3075127 to 467.2638265) | 210.0275367(171.1762919 to 230.4474091) | 3653.820827(3151.21279 to 4025.649295) |
| High-income North America | Female | 2012 | 366.3323759(285.2972196 to 464.2469124) | 206.5926699(167.8014952 to 226.8942477) | 3592.11384(3093.324802 to 3959.113132) |
| High-income North America | Female | 2013 | 364.8031429(284.5180596 to 463.16969) | 203.6484588(164.9135734 to 223.6962251) | 3544.999194(3053.482729 to 3908.484149) |
| High-income North America | Female | 2014 | 363.543966(283.9096999 to 461.5349018) | 205.5883498(166.341598 to 226.0721856) | 3560.939223(3066.497547 to 3930.499001) |
| High-income North America | Female | 2015 | 362.572667(282.8747128 to 460.0258205) | 207.2158234(167.2434975 to 227.9566736) | 3578.489306(3080.840282 to 3947.617451) |
| High-income North America | Female | 2016 | 362.8158393(282.9575546 to 461.3340306) | 208.6679699(168.3481533 to 229.6281438) | 3594.456652(3094.214133 to 3965.917341) |
| High-income North America | Female | 2017 | 364.3833606(283.4589082 to 464.4156682) | 208.1027709(167.6398685 to 229.0562791) | 3582.739479(3085.71743 to 3952.208325) |
| High-income North America | Female | 2018 | 366.0584668(283.5047714 to 467.792106) | 204.011683(163.9577336 to 224.947551) | 3524.044176(3027.405078 to 3888.822654) |
| High-income North America | Female | 2019 | 366.6085862(282.9961622 to 469.657824) | 200.7254391(161.0047536 to 221.4754934) | 3473.949081(2982.24733 to 3836.560063) |
| High-income North America | Female | 2020 | 367.4629511(281.6593849 to 474.7565844) | 197.9936936(158.7453498 to 218.8335142) | 3419.514536(2931.735097 to 3779.064226) |
| High-income North America | Female | 2021 | 366.6225012(280.7901268 to 473.4765769) | 199.0058753(159.5313998 to 220.1138936) | 3450.250459(2955.670739 to 3814.119392) |
| North Africa and Middle East | Female | 1990 | 845.1585659(676.7875945 to 1043.264717) | 1186.391726(965.4067037 to 1390.517841) | 19189.75343(15816.48865 to 22366.21614) |
| North Africa and Middle East | Female | 1991 | 845.6703061(680.7153749 to 1041.553096) | 1132.684958(924.6852036 to 1322.662235) | 18590.82061(15397.25847 to 21566.973) |
| North Africa and Middle East | Female | 1992 | 845.3075209(682.589175 to 1036.645199) | 1099.755427(897.0627871 to 1277.431035) | 18183.7863(15096.94153 to 20999.36494) |
| North Africa and Middle East | Female | 1993 | 844.2146673(683.6312533 to 1033.315215) | 1084.902889(889.1209522 to 1256.88212) | 17997.76861(14998.14512 to 20685.02195) |
| North Africa and Middle East | Female | 1994 | 842.2632795(683.2216156 to 1029.973566) | 1068.293235(876.3566692 to 1233.335289) | 17759.88781(14822.20981 to 20340.18303) |
| North Africa and Middle East | Female | 1995 | 839.6820353(682.0467959 to 1026.471953) | 1050.187343(864.6538872 to 1210.907978) | 17454.97092(14595.18945 to 19964.87064) |
| North Africa and Middle East | Female | 1996 | 835.1995029(679.0428739 to 1020.54622) | 1035.497936(855.2990241 to 1193.19476) | 17232.49557(14468.38829 to 19702.08333) |
| North Africa and Middle East | Female | 1997 | 828.357502(673.2501754 to 1013.184913) | 1021.437988(844.0580456 to 1177.221794) | 17025.74296(14314.97949 to 19448.76931) |
| North Africa and Middle East | Female | 1998 | 820.2286759(666.4249245 to 1003.077609) | 1002.288343(829.1813751 to 1155.535589) | 16742.57355(14078.7061 to 19116.73643) |
| North Africa and Middle East | Female | 1999 | 812.3352422(658.5052199 to 994.6252149) | 978.1679641(811.0296444 to 1129.19004) | 16358.05594(13778.33129 to 18702.04002) |
| North Africa and Middle East | Female | 2000 | 805.5930075(650.99313 to 988.5762921) | 952.9583898(790.7610834 to 1097.219981) | 15849.77594(13377.97726 to 18100.9951) |
| North Africa and Middle East | Female | 2001 | 798.5814753(643.9277812 to 980.4874886) | 944.7374067(786.419501 to 1084.578398) | 15684.59494(13298.12835 to 17850.55027) |
| North Africa and Middle East | Female | 2002 | 790.0109351(634.9804564 to 971.1546278) | 923.1928048(774.5057116 to 1058.34283) | 15320.40083(13074.28849 to 17405.71002) |
| North Africa and Middle East | Female | 2003 | 780.6835885(626.7116124 to 961.468046) | 900.6595362(755.3878611 to 1030.195915) | 14916.85064(12734.02575 to 16938.11289) |
| North Africa and Middle East | Female | 2004 | 771.5744766(618.2876458 to 951.6309869) | 870.1128476(729.6870743 to 997.0371959) | 14441.2954(12327.06207 to 16402.46364) |
| North Africa and Middle East | Female | 2005 | 763.44147(610.1258718 to 942.0379967) | 847.5941131(709.7884664 to 972.4157599) | 14093.34419(11995.1295 to 16038.54127) |
| North Africa and Middle East | Female | 2006 | 754.6762272(603.6749616 to 929.9834265) | 831.7350938(694.4473908 to 953.0705713) | 13806.15272(11740.58867 to 15716.49228) |
| North Africa and Middle East | Female | 2007 | 743.9924673(595.955929 to 913.5414494) | 821.6148864(684.5288337 to 942.0923178) | 13587.67801(11562.99927 to 15490.2885) |
| North Africa and Middle East | Female | 2008 | 733.039038(587.3492612 to 898.3742202) | 822.2080332(686.5411765 to 942.8335167) | 13534.11406(11520.59762 to 15417.82658) |
| North Africa and Middle East | Female | 2009 | 723.3033449(578.8202858 to 886.527637) | 824.1299884(690.0114599 to 943.4617266) | 13511.06205(11537.34544 to 15394.59072) |
| North Africa and Middle East | Female | 2010 | 716.2190639(572.7003389 to 878.9895078) | 811.8123947(680.4526356 to 930.8337584) | 13308.58538(11379.51847 to 15198.44829) |
| North Africa and Middle East | Female | 2011 | 711.9499274(570.5493218 to 871.9512424) | 798.7969488(668.799702 to 917.1051028) | 13056.27722(11136.722 to 14918.05849) |
| North Africa and Middle East | Female | 2012 | 708.9986067(568.9939479 to 865.8454854) | 792.7723674(662.21725 to 912.7506779) | 12936.98464(11007.83352 to 14811.71815) |
| North Africa and Middle East | Female | 2013 | 707.0588402(567.856309 to 862.1915144) | 786.2853618(659.2782286 to 905.0100682) | 12747.95216(10870.186 to 14600.7362) |
| North Africa and Middle East | Female | 2014 | 705.9646808(565.4904164 to 859.7021774) | 802.0637249(674.0414643 to 919.7619951) | 12892.17034(11011.05243 to 14720.44588) |
| North Africa and Middle East | Female | 2015 | 705.4808044(563.9906862 to 858.3912019) | 808.2960135(680.6077454 to 927.6037503) | 12896.03062(11047.47042 to 14689.53979) |
| North Africa and Middle East | Female | 2016 | 706.2357789(565.9260126 to 858.3925628) | 803.9394664(678.167938 to 922.2914513) | 12738.37772(10903.53537 to 14537.71713) |
| North Africa and Middle East | Female | 2017 | 707.787376(567.9175956 to 859.2595497) | 787.2239413(662.7164871 to 906.0456658) | 12409.3652(10604.13761 to 14199.70781) |
| North Africa and Middle East | Female | 2018 | 708.734812(568.7160707 to 862.9137154) | 771.3246088(645.4789897 to 893.0053755) | 12179.18634(10349.17589 to 13962.06228) |
| North Africa and Middle East | Female | 2019 | 707.9138357(567.3803577 to 865.0341892) | 767.841029(642.0176008 to 888.2216195) | 12074.85527(10250.76107 to 13877.88842) |
| North Africa and Middle East | Female | 2020 | 682.0396989(543.9962944 to 840.9950183) | 764.1430813(635.2304311 to 887.2835237) | 11982.73541(10106.06991 to 13858.43456) |
| North Africa and Middle East | Female | 2021 | 685.8292941(545.51577 to 850.078367) | 745.9838889(617.3664146 to 868.8437118) | 11748.83618(9867.283496 to 13653.78915) |
| Oceania | Female | 1990 | 933.9904028(757.5672126 to 1140.325397) | 1334.314137(1034.746366 to 1674.358856) | 24067.25929(18818.06832 to 30226.60111) |
| Oceania | Female | 1991 | 932.5241658(761.698173 to 1131.778937) | 1339.651004(1042.589569 to 1683.048183) | 24131.88402(18915.7006 to 30313.90065) |
| Oceania | Female | 1992 | 930.0297127(763.2347675 to 1123.839451) | 1336.928541(1042.183917 to 1670.764972) | 24050.48356(18824.78044 to 30062.01331) |
| Oceania | Female | 1993 | 927.3232952(763.4835792 to 1114.400834) | 1331.304012(1037.996363 to 1656.879666) | 23927.07853(18802.5294 to 29770.0791) |
| Oceania | Female | 1994 | 924.5201619(761.3359192 to 1109.043588) | 1322.490237(1035.084399 to 1637.351875) | 23738.23598(18707.89838 to 29320.52966) |
| Oceania | Female | 1995 | 921.7876994(758.6607011 to 1105.920802) | 1321.594014(1039.50306 to 1634.133846) | 23660.37794(18755.81899 to 29253.5831) |
| Oceania | Female | 1996 | 919.2097552(759.7466707 to 1103.070247) | 1324.636125(1048.519118 to 1637.270827) | 23669.6895(18915.99966 to 29210.19031) |
| Oceania | Female | 1997 | 915.0504276(756.2673492 to 1098.509757) | 1322.479327(1052.689848 to 1630.560849) | 23574.20439(18922.25063 to 28989.51634) |
| Oceania | Female | 1998 | 910.2331842(750.9123219 to 1093.672511) | 1316.361391(1047.186035 to 1625.145719) | 23422.33211(18794.31083 to 28831.11475) |
| Oceania | Female | 1999 | 905.1392005(744.1138707 to 1088.33409) | 1312.653928(1048.861417 to 1621.101568) | 23339.35352(18812.39186 to 28693.55976) |
| Oceania | Female | 2000 | 900.1727307(738.4334068 to 1083.870586) | 1307.104244(1048.768473 to 1605.432691) | 23245.89219(18787.26808 to 28395.79777) |
| Oceania | Female | 2001 | 896.3595562(735.9232564 to 1079.209168) | 1298.020477(1039.012281 to 1589.339235) | 23042.11865(18687.00753 to 28029.60255) |
| Oceania | Female | 2002 | 891.1786313(732.1658244 to 1073.28354) | 1287.013367(1029.396334 to 1568.934653) | 22857.04276(18464.57511 to 27685.50894) |
| Oceania | Female | 2003 | 885.500093(728.0405788 to 1066.17666) | 1277.155156(1021.053252 to 1554.460284) | 22673.51927(18326.03247 to 27434.74606) |
| Oceania | Female | 2004 | 879.5925252(722.6089326 to 1059.015748) | 1261.191198(1010.839114 to 1532.932642) | 22389.24873(18117.43102 to 27037.84243) |
| Oceania | Female | 2005 | 873.7471178(716.8919714 to 1051.65942) | 1256.60352(1004.207198 to 1526.935924) | 22314.44841(18055.03861 to 26953.0646) |
| Oceania | Female | 2006 | 867.8819796(713.764092 to 1042.182414) | 1263.277234(1011.967969 to 1530.21571) | 22363.01325(18130.44121 to 26964.54438) |
| Oceania | Female | 2007 | 859.1414872(708.6649066 to 1031.590562) | 1245.59416(997.7786076 to 1508.007977) | 22073.72473(17891.93605 to 26606.70342) |
| Oceania | Female | 2008 | 850.0437433(702.1297552 to 1021.277119) | 1232.021137(989.1045158 to 1491.210187) | 21833.82898(17718.12761 to 26282.8384) |
| Oceania | Female | 2009 | 841.5397011(694.805669 to 1010.599799) | 1211.085155(972.3886062 to 1470.854138) | 21463.38893(17412.95834 to 25959.79318) |
| Oceania | Female | 2010 | 834.4877406(688.409428 to 1003.503192) | 1197.869182(963.2984616 to 1459.260898) | 21235.17315(17234.23304 to 25713.48881) |
| Oceania | Female | 2011 | 826.5654747(682.7320564 to 991.3506892) | 1194.324076(957.5207972 to 1455.424199) | 21115.90839(17086.21958 to 25597.97371) |
| Oceania | Female | 2012 | 815.9450639(674.6305874 to 976.6280232) | 1176.215929(941.1749716 to 1443.069292) | 20835.08651(16867.72244 to 25473.41814) |
| Oceania | Female | 2013 | 805.6151702(666.6480618 to 964.0878333) | 1157.81189(927.8824904 to 1420.236689) | 20522.68781(16584.88643 to 25071.72881) |
| Oceania | Female | 2014 | 797.5082309(659.2898307 to 956.5638559) | 1144.644123(913.8938972 to 1404.345517) | 20321.28854(16411.26858 to 24907.59488) |
| Oceania | Female | 2015 | 793.5730268(653.5807646 to 954.8106706) | 1133.686182(903.660239 to 1396.368184) | 20169.20662(16220.52598 to 24790.50469) |
| Oceania | Female | 2016 | 793.0345267(654.6486991 to 949.852623) | 1121.015439(889.0530768 to 1377.46399) | 19883.62434(15923.19861 to 24355.21645) |
| Oceania | Female | 2017 | 792.3965231(655.7045325 to 947.2773153) | 1109.454598(875.3871318 to 1363.659553) | 19710.99862(15670.84444 to 24187.74965) |
| Oceania | Female | 2018 | 792.0648472(655.3213455 to 947.5088795) | 1100.73748(870.1503012 to 1358.552127) | 19573.58047(15614.45636 to 24091.40975) |
| Oceania | Female | 2019 | 791.3465211(653.8722316 to 951.4317486) | 1095.92654(869.417447 to 1358.979815) | 19525.32302(15645.65749 to 24118.42846) |
| Oceania | Female | 2020 | 781.0595099(639.6422019 to 942.6445062) | 1086.094935(858.4455219 to 1352.359729) | 19394.56079(15460.51807 to 23985.89712) |
| Oceania | Female | 2021 | 777.7595057(636.2313525 to 938.4266995) | 1076.995472(846.8335885 to 1349.258964) | 19216.50714(15177.05216 to 23864.87929) |
| South Asia | Female | 1990 | 784.0638691(607.5612112 to 992.6866816) | 674.930491(548.9278584 to 787.0847859) | 12347.00232(10190.25935 to 14241.3649) |
| South Asia | Female | 1991 | 782.0250173(608.8421886 to 986.7035763) | 666.1002798(538.9084074 to 776.7062473) | 12201.4649(10048.28564 to 14081.24531) |
| South Asia | Female | 1992 | 779.2591286(609.6333906 to 979.5955697) | 682.7946848(555.1270981 to 790.8425621) | 12398.59074(10219.39274 to 14191.58313) |
| South Asia | Female | 1993 | 775.7063067(609.3978064 to 972.6954993) | 671.7575582(544.1153842 to 780.4431922) | 12192.42368(10043.83485 to 13988.4208) |
| South Asia | Female | 1994 | 771.3446193(607.0826043 to 965.1019162) | 676.8624421(550.2847245 to 783.9225949) | 12246.33431(10122.24239 to 14059.09525) |
| South Asia | Female | 1995 | 766.4520606(603.6665413 to 956.921332) | 707.4425201(577.4778212 to 819.6999126) | 12564.27669(10412.09799 to 14398.94273) |
| South Asia | Female | 1996 | 759.5815939(599.0550561 to 947.5330653) | 688.1417516(564.3766186 to 793.2638779) | 12292.70123(10244.1746 to 14005.07019) |
| South Asia | Female | 1997 | 749.7420595(592.0688831 to 934.4811075) | 657.5879181(539.4874509 to 756.9512189) | 11939.37018(9938.832028 to 13564.52359) |
| South Asia | Female | 1998 | 738.0804951(582.6860124 to 919.7588797) | 648.2604432(532.8650291 to 745.0362839) | 11849.00041(9920.320267 to 13478.91225) |
| South Asia | Female | 1999 | 725.5266364(572.4324668 to 904.7525269) | 622.9212321(515.8915966 to 713.8110861) | 11360.66264(9543.052561 to 12893.19561) |
| South Asia | Female | 2000 | 713.0853917(562.0821519 to 889.4312146) | 600.8103759(497.3462629 to 681.9378649) | 10968.05332(9222.412159 to 12322.38607) |
| South Asia | Female | 2001 | 700.4034586(552.377347 to 874.4623013) | 593.9641743(494.242754 to 675.6240034) | 10855.53438(9185.433585 to 12198.80992) |
| South Asia | Female | 2002 | 686.3593253(541.3696731 to 858.0010812) | 599.1600705(504.3469336 to 681.427686) | 10931.90272(9340.565618 to 12313.6433) |
| South Asia | Female | 2003 | 671.8333729(529.8541158 to 839.8561109) | 593.9316528(499.9752863 to 673.0733459) | 10803.21304(9250.657788 to 12108.9445) |
| South Asia | Female | 2004 | 658.1445979(518.8915368 to 822.445222) | 568.6030543(481.608514 to 646.5874303) | 10299.50913(8867.306044 to 11588.01544) |
| South Asia | Female | 2005 | 645.9826805(509.4269914 to 807.6123232) | 565.5726598(477.6213038 to 642.5880779) | 10256.58088(8803.651165 to 11544.01141) |
| South Asia | Female | 2006 | 633.5503367(501.1514697 to 790.9988392) | 579.4090013(494.284932 to 654.6464299) | 10456.68312(9081.72262 to 11681.39817) |
| South Asia | Female | 2007 | 619.6990346(491.0335215 to 772.5105584) | 588.9020496(505.0599303 to 664.6895278) | 10550.50055(9188.416024 to 11807.60052) |
| South Asia | Female | 2008 | 605.7079044(480.4486592 to 754.3709458) | 582.2852314(499.1788475 to 658.2329851) | 10416.43792(9076.985877 to 11684.39386) |
| South Asia | Female | 2009 | 592.8519925(470.4280286 to 737.5074789) | 556.9428563(477.936912 to 628.6669464) | 10038.34104(8756.135747 to 11213.82478) |
| South Asia | Female | 2010 | 582.4733322(462.0506481 to 723.937589) | 541.6159062(466.4482461 to 611.4058453) | 9823.657926(8589.902986 to 10998.85506) |
| South Asia | Female | 2011 | 572.9998763(454.7790106 to 710.9339109) | 535.4357542(460.9561088 to 604.538533) | 9790.181715(8551.309539 to 10933.66819) |
| South Asia | Female | 2012 | 563.3686352(447.2502724 to 698.0702007) | 535.8734028(460.4277499 to 607.4633541) | 9834.946339(8580.695384 to 11022.58634) |
| South Asia | Female | 2013 | 555.2954519(441.3417934 to 686.5129851) | 549.7743183(477.7660234 to 620.7184669) | 9786.862339(8617.9451 to 10946.06814) |
| South Asia | Female | 2014 | 550.2322647(437.7119246 to 679.2092981) | 548.0828727(476.5114591 to 620.667458) | 9541.999142(8415.547608 to 10721.94726) |
| South Asia | Female | 2015 | 549.3076773(437.6470058 to 677.9569705) | 538.3979103(467.2580114 to 607.6057785) | 9501.846368(8354.524258 to 10604.1219) |
| South Asia | Female | 2016 | 553.1072307(441.1730299 to 682.8460588) | 534.4560988(463.2965819 to 606.7979488) | 9503.363071(8352.242705 to 10698.12502) |
| South Asia | Female | 2017 | 559.7509864(446.7630185 to 691.1130859) | 543.3475496(469.8203063 to 612.7249731) | 9662.875336(8490.001073 to 10815.70566) |
| South Asia | Female | 2018 | 566.7242233(452.1412902 to 700.4744404) | 538.6877063(468.1934256 to 612.4535062) | 9669.822105(8524.026226 to 10881.37412) |
| South Asia | Female | 2019 | 571.6831438(455.3052924 to 707.891451) | 531.1689686(459.0920242 to 602.4893488) | 9539.923754(8387.751146 to 10745.18971) |
| South Asia | Female | 2020 | 564.574118(446.7952935 to 704.1224552) | 518.3242991(445.9967387 to 589.4616443) | 9284.646035(8138.263505 to 10489.15107) |
| South Asia | Female | 2021 | 567.3764927(450.3540697 to 706.9026089) | 506.2862262(432.326757 to 580.5493865) | 9057.946304(7843.66343 to 10320.03636) |
| Southeast Asia | Female | 1990 | 1058.56291(844.7895967 to 1305.24644) | 1241.20675(1051.973794 to 1446.856716) | 21771.61452(18818.26224 to 25019.39686) |
| Southeast Asia | Female | 1991 | 1059.873572(850.5322127 to 1302.61359) | 1237.118478(1059.289019 to 1428.46578) | 21672.29647(18889.69945 to 24659.02756) |
| Southeast Asia | Female | 1992 | 1061.208827(855.3118309 to 1302.279026) | 1237.708302(1068.767925 to 1420.25869) | 21663.33424(19051.63534 to 24541.82898) |
| Southeast Asia | Female | 1993 | 1062.150963(858.1456523 to 1300.792831) | 1238.275065(1075.693297 to 1410.744535) | 21664.56517(19168.11275 to 24379.21862) |
| Southeast Asia | Female | 1994 | 1062.693274(859.4591932 to 1299.545986) | 1238.996769(1079.067574 to 1412.797518) | 21646.78965(19157.05379 to 24311.44818) |
| Southeast Asia | Female | 1995 | 1062.81748(859.7945296 to 1298.72889) | 1238.447926(1085.447998 to 1401.61248) | 21607.17757(19271.94945 to 24161.43507) |
| Southeast Asia | Female | 1996 | 1062.592518(860.2372442 to 1298.579948) | 1239.316654(1084.588841 to 1392.919119) | 21600.94952(19226.46146 to 23990.49265) |
| Southeast Asia | Female | 1997 | 1061.832553(860.0972594 to 1297.060466) | 1231.102399(1079.969664 to 1381.283821) | 21465.27786(19159.51262 to 23849.55659) |
| Southeast Asia | Female | 1998 | 1060.456444(859.4807085 to 1295.735995) | 1225.645848(1083.490899 to 1373.932486) | 21343.59671(19161.12616 to 23696.63685) |
| Southeast Asia | Female | 1999 | 1058.4322(858.5436177 to 1293.647124) | 1230.488339(1086.31602 to 1371.343416) | 21390.89183(19167.00547 to 23604.30551) |
| Southeast Asia | Female | 2000 | 1055.837215(855.2520258 to 1292.16841) | 1228.386203(1084.855339 to 1366.51904) | 21318.91486(19095.52467 to 23531.14919) |
| Southeast Asia | Female | 2001 | 1052.020154(853.0007451 to 1286.256545) | 1227.317797(1079.404131 to 1364.935814) | 21239.8076(18951.4661 to 23433.98502) |
| Southeast Asia | Female | 2002 | 1046.689694(849.0217146 to 1278.585814) | 1236.290226(1086.520486 to 1376.600876) | 21315.81511(19025.95203 to 23604.1273) |
| Southeast Asia | Female | 2003 | 1040.230045(844.6338187 to 1269.347774) | 1235.702309(1090.475982 to 1372.88106) | 21256.68501(19040.57854 to 23426.97284) |
| Southeast Asia | Female | 2004 | 1033.076792(839.5632948 to 1261.776241) | 1236.257218(1089.868067 to 1376.282269) | 21205.87132(18948.14732 to 23455.22256) |
| Southeast Asia | Female | 2005 | 1025.834078(833.8952986 to 1253.36688) | 1232.371548(1086.439668 to 1369.910956) | 21060.83232(18829.75583 to 23259.1889) |
| Southeast Asia | Female | 2006 | 1016.939781(827.5152104 to 1240.787182) | 1222.386842(1079.700283 to 1355.362849) | 20829.32591(18677.90112 to 22965.57681) |
| Southeast Asia | Female | 2007 | 1005.305103(818.4849974 to 1225.10834) | 1205.091402(1071.380199 to 1338.609281) | 20498.82969(18458.07088 to 22636.01404) |
| Southeast Asia | Female | 2008 | 992.0880764(807.8996054 to 1208.038273) | 1188.110312(1051.272872 to 1316.93752) | 20178.69446(18100.22943 to 22291.09761) |
| Southeast Asia | Female | 2009 | 978.7807086(797.4872952 to 1192.807129) | 1169.379746(1025.330587 to 1297.74887) | 19834.39265(17644.89455 to 21902.55357) |
| Southeast Asia | Female | 2010 | 967.1290481(788.3487761 to 1178.389583) | 1147.27989(1006.115187 to 1275.967364) | 19442.40099(17262.14445 to 21622.6588) |
| Southeast Asia | Female | 2011 | 954.1700045(778.4966895 to 1161.72627) | 1120.131137(979.1841453 to 1255.46382) | 18966.68478(16763.95564 to 21098.38534) |
| Southeast Asia | Female | 2012 | 939.483502(767.3955669 to 1143.630359) | 1090.831739(954.6282026 to 1217.023712) | 18480.57902(16392.46243 to 20527.95685) |
| Southeast Asia | Female | 2013 | 925.4988336(755.4210436 to 1126.778214) | 1067.72104(922.5318877 to 1198.144049) | 18093.51986(15868.40555 to 20249.53459) |
| Southeast Asia | Female | 2014 | 914.7515545(745.9592602 to 1112.861097) | 1046.146326(912.6975715 to 1172.061425) | 17741.36185(15660.65153 to 19784.4454) |
| Southeast Asia | Female | 2015 | 909.9124995(741.658668 to 1107.235453) | 1029.921557(883.6306268 to 1158.72121) | 17482.39488(15277.05482 to 19650.59273) |
| Southeast Asia | Female | 2016 | 910.2377136(742.9543357 to 1107.365656) | 1011.373071(879.6945089 to 1144.472701) | 17160.54586(15102.76895 to 19329.41859) |
| Southeast Asia | Female | 2017 | 912.6777228(745.3555673 to 1108.472478) | 992.5590133(852.9639641 to 1118.351478) | 16855.0046(14735.8583 to 18905.95938) |
| Southeast Asia | Female | 2018 | 915.221685(746.2884365 to 1112.081815) | 976.2260425(838.3904435 to 1103.061993) | 16587.82076(14453.59726 to 18612.8861) |
| Southeast Asia | Female | 2019 | 916.0575327(746.1076624 to 1113.12537) | 963.6311266(827.7971638 to 1093.896145) | 16380.47737(14250.3808 to 18571.44708) |
| Southeast Asia | Female | 2020 | 893.4225434(723.0926088 to 1098.667991) | 944.8047554(806.6830376 to 1074.649853) | 16086.04772(13909.46727 to 18241.38615) |
| Southeast Asia | Female | 2021 | 898.639619(726.7065998 to 1104.013252) | 938.0581065(798.2967759 to 1071.146691) | 15984.41282(13779.5874 to 18191.66497) |
| Southern Latin America | Female | 1990 | 746.11508(604.816686 to 913.1434405) | 656.2843179(581.6254863 to 711.666765) | 10791.75876(9762.476218 to 11662.85394) |
| Southern Latin America | Female | 1991 | 735.7457672(598.2756849 to 893.0098419) | 631.3078778(559.5419786 to 684.5345038) | 10399.63779(9413.707113 to 11249.06169) |
| Southern Latin America | Female | 1992 | 724.6915977(591.6906183 to 877.6829302) | 609.9120817(540.5179342 to 662.2516114) | 10074.47746(9120.701981 to 10886.65977) |
| Southern Latin America | Female | 1993 | 713.1692469(582.1937975 to 864.4717794) | 576.6407877(509.2764192 to 627.7263698) | 9595.59549(8668.948653 to 10374.43476) |
| Southern Latin America | Female | 1994 | 701.3909575(570.1589778 to 852.2887511) | 536.8308735(473.0480153 to 586.0373497) | 8993.094482(8099.212852 to 9733.46064) |
| Southern Latin America | Female | 1995 | 689.5769135(558.5304042 to 843.1839215) | 513.443628(451.9564851 to 559.9411036) | 8612.36023(7745.510035 to 9331.955419) |
| Southern Latin America | Female | 1996 | 677.3418682(549.4383619 to 822.6081267) | 490.1212953(430.4948777 to 535.2089218) | 8245.985332(7412.596597 to 8948.915027) |
| Southern Latin America | Female | 1997 | 664.2113064(538.6432612 to 805.4290137) | 473.0596312(414.4221102 to 517.779442) | 7915.911922(7107.334911 to 8608.835071) |
| Southern Latin America | Female | 1998 | 650.2587934(528.9863608 to 790.747611) | 472.8788433(415.3475345 to 517.2606201) | 7875.369082(7071.371381 to 8560.57628) |
| Southern Latin America | Female | 1999 | 635.543542(516.1087639 to 773.5685654) | 464.4300629(407.7546136 to 508.266125) | 7713.391284(6913.00447 to 8401.66624) |
| Southern Latin America | Female | 2000 | 620.1112489(501.1470363 to 759.9069387) | 435.1213138(380.7589188 to 476.9176943) | 7252.293471(6478.936735 to 7909.281335) |
| Southern Latin America | Female | 2001 | 602.0149435(489.6694023 to 732.3401173) | 422.9722183(370.3155045 to 463.1037221) | 7071.612435(6331.485206 to 7707.496028) |
| Southern Latin America | Female | 2002 | 580.8347894(474.5883835 to 703.9184919) | 414.6535817(363.7162279 to 454.2162669) | 6949.454333(6229.96809 to 7580.384126) |
| Southern Latin America | Female | 2003 | 558.9775838(458.035711 to 673.2661347) | 415.3604768(364.2215644 to 454.3428569) | 6917.302982(6195.499892 to 7538.686845) |
| Southern Latin America | Female | 2004 | 538.8018155(442.2570409 to 648.070073) | 400.6136411(350.8758163 to 438.0995786) | 6669.430077(5965.83832 to 7261.111524) |
| Southern Latin America | Female | 2005 | 522.6157898(427.4439916 to 631.0639123) | 376.4797803(328.7679708 to 412.4008142) | 6291.279043(5620.833122 to 6856.881938) |
| Southern Latin America | Female | 2006 | 509.4860585(418.5465848 to 613.5719662) | 359.9278792(313.7404058 to 395.232423) | 6022.70618(5379.776182 to 6585.981405) |
| Southern Latin America | Female | 2007 | 497.0820533(408.7991511 to 598.0526672) | 355.1031963(309.4403373 to 390.0348202) | 5911.631746(5282.98981 to 6481.388997) |
| Southern Latin America | Female | 2008 | 485.6900813(398.9276797 to 584.8452462) | 336.3303127(292.2033671 to 369.8324426) | 5636.594474(5027.237343 to 6187.871225) |
| Southern Latin America | Female | 2009 | 475.6172453(389.7890688 to 574.3069082) | 327.9831722(284.019498 to 360.9414581) | 5490.432592(4891.179196 to 6024.296439) |
| Southern Latin America | Female | 2010 | 467.1558208(381.9697938 to 565.7676434) | 326.3764467(282.6640072 to 359.8110097) | 5429.915487(4837.978612 to 5962.297162) |
| Southern Latin America | Female | 2011 | 459.2480072(376.6764153 to 555.4787174) | 318.59837(275.4605476 to 351.8695517) | 5292.290203(4704.549829 to 5813.388892) |
| Southern Latin America | Female | 2012 | 451.21171(369.6227796 to 546.3901678) | 309.6368564(267.8131231 to 343.1121891) | 5141.620884(4565.011343 to 5660.505054) |
| Southern Latin America | Female | 2013 | 444.0355777(362.9787762 to 538.4344083) | 303.1670814(262.109725 to 335.6266604) | 5028.191037(4463.848149 to 5532.998597) |
| Southern Latin America | Female | 2014 | 438.6533677(357.6202525 to 532.9188127) | 298.7313862(258.1222727 to 331.1084309) | 4961.661097(4401.726132 to 5461.247807) |
| Southern Latin America | Female | 2015 | 435.9995748(353.9271925 to 531.8967428) | 296.5679004(256.1662474 to 328.725957) | 4918.934651(4361.817374 to 5412.29748) |
| Southern Latin America | Female | 2016 | 435.9884803(355.7756049 to 529.2571985) | 294.4608143(253.7368124 to 326.7000333) | 4883.845241(4324.238502 to 5367.759815) |
| Southern Latin America | Female | 2017 | 437.1462015(357.3676678 to 529.4288473) | 278.9413575(240.0342794 to 310.0502909) | 4642.484646(4106.335351 to 5117.54646) |
| Southern Latin America | Female | 2018 | 438.3561994(357.4669105 to 531.5615201) | 269.7570705(231.3347661 to 299.682986) | 4521.939736(3977.755712 to 4995.282502) |
| Southern Latin America | Female | 2019 | 438.4701878(354.9877667 to 534.1217812) | 265.3519462(227.342378 to 295.3002524) | 4450.090136(3908.119394 to 4929.727319) |
| Southern Latin America | Female | 2020 | 426.5543462(344.534226 to 522.109249) | 249.6980972(211.9462705 to 278.6518485) | 4248.58341(3711.474021 to 4713.295211) |
| Southern Latin America | Female | 2021 | 425.4095828(344.5977097 to 517.5195111) | 240.048249(202.1661992 to 268.6492589) | 4093.367612(3571.1209 to 4539.116684) |
| Southern Sub-Saharan Africa | Female | 1990 | 1023.526159(762.2199246 to 1343.00605) | 630.4612329(499.9548592 to 742.9006623) | 11330.32974(9268.011782 to 13138.44034) |
| Southern Sub-Saharan Africa | Female | 1991 | 1036.139306(778.4221961 to 1353.003808) | 616.6209348(487.8710313 to 730.2057987) | 11077.00765(9041.356414 to 12917.63433) |
| Southern Sub-Saharan Africa | Female | 1992 | 1049.983486(793.6727733 to 1366.756907) | 652.2183215(525.6877035 to 760.5259498) | 11582.8022(9590.646773 to 13354.60114) |
| Southern Sub-Saharan Africa | Female | 1993 | 1064.836528(808.2187356 to 1383.197973) | 635.9920045(511.013593 to 747.5064646) | 11291.59726(9324.757155 to 13059.20231) |
| Southern Sub-Saharan Africa | Female | 1994 | 1080.456248(822.0414922 to 1401.917968) | 672.86389(549.7373519 to 781.435201) | 11873.61602(9952.283514 to 13619.06667) |
| Southern Sub-Saharan Africa | Female | 1995 | 1096.550038(835.6662706 to 1424.503472) | 655.7899272(537.6758541 to 764.3272084) | 11583.25673(9767.455121 to 13321.05596) |
| Southern Sub-Saharan Africa | Female | 1996 | 1115.964735(851.0390097 to 1446.427749) | 717.2496115(606.6017053 to 819.5969004) | 12604.24621(10898.55921 to 14213.27417) |
| Southern Sub-Saharan Africa | Female | 1997 | 1139.407907(868.4583481 to 1475.851651) | 804.4652214(702.6333628 to 903.3173389) | 14076.82772(12495.95642 to 15662.52618) |
| Southern Sub-Saharan Africa | Female | 1998 | 1164.033807(886.8258843 to 1506.16168) | 836.8114003(735.2167189 to 929.9981595) | 14592.01011(13036.85655 to 16064.09431) |
| Southern Sub-Saharan Africa | Female | 1999 | 1186.967004(903.1400003 to 1536.272036) | 851.3533146(751.3465151 to 943.3821957) | 14796.91934(13245.12163 to 16275.20898) |
| Southern Sub-Saharan Africa | Female | 2000 | 1204.993161(915.9752478 to 1559.632942) | 907.2390832(801.6006912 to 998.5326098) | 15705.49111(14113.06878 to 17196.37042) |
| Southern Sub-Saharan Africa | Female | 2001 | 1221.335422(928.3794729 to 1582.009482) | 925.7548062(817.7912306 to 1020.067545) | 15958.75578(14366.00231 to 17463.98489) |
| Southern Sub-Saharan Africa | Female | 2002 | 1239.388587(941.8619428 to 1607.557114) | 960.714428(849.0041046 to 1056.718217) | 16496.79146(14826.41737 to 18056.22525) |
| Southern Sub-Saharan Africa | Female | 2003 | 1256.325047(953.2863937 to 1631.263585) | 988.8465399(872.7287736 to 1086.183778) | 16936.01937(15234.00379 to 18550.90737) |
| Southern Sub-Saharan Africa | Female | 2004 | 1268.986085(960.0381805 to 1649.848115) | 982.7038972(869.0897064 to 1078.230008) | 16840.6602(15133.21236 to 18414.55027) |
| Southern Sub-Saharan Africa | Female | 2005 | 1274.442964(961.4463126 to 1661.014109) | 984.4151161(870.8055069 to 1080.731476) | 16895.09469(15176.24126 to 18496.93127) |
| Southern Sub-Saharan Africa | Female | 2006 | 1268.305161(960.9524391 to 1648.803122) | 997.8800198(881.6785036 to 1097.416609) | 17097.30351(15351.82206 to 18719.19914) |
| Southern Sub-Saharan Africa | Female | 2007 | 1250.827902(949.9905294 to 1621.550495) | 999.2887752(884.8253801 to 1098.305596) | 17053.05033(15320.9861 to 18720.34272) |
| Southern Sub-Saharan Africa | Female | 2008 | 1226.629652(935.9995687 to 1585.941501) | 997.7945415(881.5959376 to 1097.306725) | 17042.24328(15290.9841 to 18678.1417) |
| Southern Sub-Saharan Africa | Female | 2009 | 1200.136653(920.3850332 to 1547.679374) | 1000.549956(884.4665676 to 1101.10604) | 17081.29858(15307.72448 to 18771.01053) |
| Southern Sub-Saharan Africa | Female | 2010 | 1176.103643(900.8144042 to 1515.362787) | 986.8169196(871.4730207 to 1086.95105) | 16847.83616(15117.07638 to 18481.74239) |
| Southern Sub-Saharan Africa | Female | 2011 | 1150.656437(883.1049066 to 1480.762653) | 960.233449(846.1713344 to 1055.264739) | 16382.60363(14681.38059 to 17932.84051) |
| Southern Sub-Saharan Africa | Female | 2012 | 1120.605569(859.3514112 to 1442.520785) | 931.5832956(821.4290651 to 1021.937464) | 15849.04618(14212.86565 to 17344.03806) |
| Southern Sub-Saharan Africa | Female | 2013 | 1091.789699(837.1771567 to 1406.015327) | 909.0413405(800.4050436 to 1000.966212) | 15446.90937(13819.94971 to 16900.30378) |
| Southern Sub-Saharan Africa | Female | 2014 | 1069.845982(820.9949025 to 1376.550823) | 907.1178889(799.1185227 to 999.045142) | 15411.48897(13791.06979 to 16887.00824) |
| Southern Sub-Saharan Africa | Female | 2015 | 1060.40593(812.7551063 to 1363.034709) | 906.6923642(798.2189299 to 998.6993714) | 15396.05194(13764.60556 to 16885.3459) |
| Southern Sub-Saharan Africa | Female | 2016 | 1060.508942(814.9234477 to 1362.350394) | 886.9200723(779.8738792 to 977.9738398) | 15095.64763(13477.25181 to 16562.09764) |
| Southern Sub-Saharan Africa | Female | 2017 | 1062.540569(818.2985911 to 1365.210426) | 852.8868373(748.4095062 to 943.5427361) | 14529.28796(12915.43808 to 16001.6528) |
| Southern Sub-Saharan Africa | Female | 2018 | 1064.62016(820.7954582 to 1368.399606) | 830.8912639(726.3185238 to 922.0535406) | 14151.55725(12568.37209 to 15596.66354) |
| Southern Sub-Saharan Africa | Female | 2019 | 1064.750342(820.1787129 to 1370.475041) | 799.2017547(695.0473561 to 892.3242137) | 13580.54302(12006.16154 to 15040.40571) |
| Southern Sub-Saharan Africa | Female | 2020 | 1020.954779(779.6571948 to 1322.154487) | 812.4267932(708.9926305 to 907.4873759) | 13798.3353(12228.47283 to 15325.23083) |
| Southern Sub-Saharan Africa | Female | 2021 | 1025.222073(780.407712 to 1334.946628) | 784.6516337(679.3642017 to 879.8201888) | 13429.64376(11838.4581 to 14947.96123) |
| Tropical Latin America | Female | 1990 | 783.4605643(580.9935894 to 1036.389916) | 792.5338035(700.2154386 to 844.2444325) | 12946.11434(11743.0096 to 13686.8849) |
| Tropical Latin America | Female | 1991 | 776.7194485(578.4082593 to 1024.212035) | 743.5055793(654.0896919 to 792.6334452) | 12204.11424(11036.20538 to 12910.19905) |
| Tropical Latin America | Female | 1992 | 768.1802093(573.6911263 to 1010.365163) | 729.1062399(642.4061549 to 777.8699544) | 12007.48717(10864.03017 to 12693.58015) |
| Tropical Latin America | Female | 1993 | 758.229441(568.2788984 to 994.5205992) | 728.5046287(641.9090933 to 777.6868573) | 11990.88891(10845.82019 to 12686.66966) |
| Tropical Latin America | Female | 1994 | 747.2085605(562.1368019 to 977.9183237) | 700.5085773(615.9957332 to 748.4629986) | 11548.09199(10432.75847 to 12243.33658) |
| Tropical Latin America | Female | 1995 | 735.4478852(555.8437688 to 958.9182038) | 670.643881(588.6432585 to 716.7107756) | 11090.83087(10008.45281 to 11749.78452) |
| Tropical Latin America | Female | 1996 | 719.6260552(544.4395335 to 937.50558) | 642.4672106(562.2118949 to 687.9507491) | 10646.56014(9594.677927 to 11302.93305) |
| Tropical Latin America | Female | 1997 | 698.2000849(528.6201312 to 909.2156456) | 613.324175(535.6240965 to 657.7594332) | 10182.01775(9169.070078 to 10820.2682) |
| Tropical Latin America | Female | 1998 | 674.148434(510.7795289 to 876.9092106) | 592.0348219(516.4562716 to 635.0376123) | 9860.118123(8879.80279 to 10487.60492) |
| Tropical Latin America | Female | 1999 | 650.4740104(492.3799197 to 846.1775545) | 568.0034151(495.1203264 to 609.5056999) | 9499.83184(8543.327967 to 10103.31735) |
| Tropical Latin America | Female | 2000 | 630.2405415(476.4814352 to 819.5859283) | 543.5834358(471.9608297 to 584.431128) | 9099.764309(8159.703924 to 9686.17545) |
| Tropical Latin America | Female | 2001 | 613.1901578(463.0922446 to 798.5576669) | 530.6750118(459.2712883 to 570.8052108) | 8858.679298(7926.26747 to 9433.569168) |
| Tropical Latin America | Female | 2002 | 596.9468853(450.601057 to 778.3161529) | 521.1532195(451.0453496 to 560.6406289) | 8678.053343(7764.643781 to 9244.768938) |
| Tropical Latin America | Female | 2003 | 581.4508394(438.6941764 to 757.803239) | 509.0308053(440.2487837 to 547.155375) | 8475.748386(7593.3069 to 9017.939414) |
| Tropical Latin America | Female | 2004 | 566.6500702(427.3017907 to 738.4462362) | 496.2526288(427.533798 to 534.3227512) | 8271.282696(7390.231004 to 8824.25022) |
| Tropical Latin America | Female | 2005 | 552.4781277(416.7911739 to 720.1801974) | 475.6159708(407.808706 to 513.1141168) | 7903.831477(7035.733396 to 8443.796159) |
| Tropical Latin America | Female | 2006 | 538.2456866(407.0447081 to 699.3417079) | 469.7480661(402.1897267 to 507.1049121) | 7782.087464(6918.045249 to 8315.947855) |
| Tropical Latin America | Female | 2007 | 523.8471(397.8099766 to 679.4918741) | 454.337451(388.9199518 to 490.7677452) | 7534.58469(6692.249772 to 8054.634399) |
| Tropical Latin America | Female | 2008 | 509.9773219(388.6260468 to 659.9684173) | 442.155351(377.6070262 to 478.5005494) | 7324.1019(6497.487014 to 7838.972449) |
| Tropical Latin America | Female | 2009 | 497.365385(379.4327367 to 642.4286352) | 433.3020126(368.9505928 to 468.4219787) | 7149.770431(6330.389023 to 7646.122739) |
| Tropical Latin America | Female | 2010 | 486.7362074(372.6029986 to 627.0173357) | 418.7152812(357.9339475 to 452.7235075) | 6890.625274(6115.992484 to 7370.037921) |
| Tropical Latin America | Female | 2011 | 476.807357(366.1338577 to 613.0075137) | 406.4970032(347.5353223 to 439.5842707) | 6687.503054(5934.829021 to 7156.11168) |
| Tropical Latin America | Female | 2012 | 466.6751285(359.0426587 to 599.9665292) | 390.1869212(332.9999153 to 422.1068507) | 6426.791894(5688.8956 to 6875.717162) |
| Tropical Latin America | Female | 2013 | 457.5534367(352.7664412 to 587.9561084) | 376.4146519(321.1432158 to 407.7468263) | 6193.255604(5472.337798 to 6638.441496) |
| Tropical Latin America | Female | 2014 | 450.65835(347.970407 to 578.5741098) | 363.7803969(309.4772685 to 393.902397) | 5966.709987(5259.126851 to 6390.323698) |
| Tropical Latin America | Female | 2015 | 447.1795901(344.9068929 to 573.9416928) | 356.3917802(303.6274011 to 386.2563691) | 5845.766719(5164.039395 to 6273.214198) |
| Tropical Latin America | Female | 2016 | 446.4579708(344.7637284 to 573.044865) | 351.5996662(299.2440997 to 381.2384989) | 5781.635808(5109.00712 to 6197.281117) |
| Tropical Latin America | Female | 2017 | 446.5331195(344.5894445 to 573.8519636) | 331.8245374(282.270607 to 359.915754) | 5474.818332(4831.087844 to 5870.346025) |
| Tropical Latin America | Female | 2018 | 446.6047981(344.1439517 to 574.5656993) | 318.4249934(269.8421677 to 345.9094939) | 5275.211632(4648.458845 to 5670.996787) |
| Tropical Latin America | Female | 2019 | 445.87087(343.0787473 to 574.4213149) | 312.007598(264.2490804 to 339.0602604) | 5169.382302(4552.630496 to 5550.073086) |
| Tropical Latin America | Female | 2020 | 436.7575062(333.379773 to 566.4285544) | 305.5835415(257.9299021 to 333.6108015) | 5107.035238(4481.982123 to 5505.773841) |
| Tropical Latin America | Female | 2021 | 424.6584237(324.3478436 to 546.3690438) | 294.8091266(247.0379876 to 323.1518041) | 4975.182875(4349.899661 to 5382.655977) |
| Western Europe | Female | 1990 | 723.1830746(580.2168119 to 886.5978088) | 582.3552449(505.1977401 to 623.4289806) | 8553.074431(7649.517426 to 9102.411129) |
| Western Europe | Female | 1991 | 709.9782535(572.1477678 to 862.91922) | 562.9284411(487.0509853 to 603.3740858) | 8270.081574(7390.315505 to 8810.674031) |
| Western Europe | Female | 1992 | 696.1315321(563.8416714 to 841.6961137) | 538.3810088(463.5786248 to 577.9934086) | 7918.268595(7049.360158 to 8451.646969) |
| Western Europe | Female | 1993 | 682.1525321(555.8635481 to 821.7361618) | 518.5718746(446.1322145 to 556.5774805) | 7635.350269(6787.576778 to 8151.25719) |
| Western Europe | Female | 1994 | 668.4939544(546.8719886 to 802.9160355) | 496.6014245(424.8987795 to 533.8236974) | 7321.244617(6481.461147 to 7832.251692) |
| Western Europe | Female | 1995 | 655.1853695(537.4498122 to 784.0306962) | 480.249066(409.9055476 to 516.8848794) | 7078.665924(6262.965089 to 7584.322919) |
| Western Europe | Female | 1996 | 640.2527005(528.0393235 to 763.0775056) | 464.5290132(396.1089622 to 500.1245419) | 6834.915332(6042.882847 to 7332.901025) |
| Western Europe | Female | 1997 | 622.7826486(515.5977862 to 739.8399794) | 447.5137784(381.2341018 to 482.5653794) | 6584.299798(5803.100363 to 7071.499883) |
| Western Europe | Female | 1998 | 604.3187721(500.9676565 to 715.1378298) | 434.4117996(369.5974159 to 468.4207908) | 6398.831223(5625.959899 to 6868.02509) |
| Western Europe | Female | 1999 | 586.2399311(486.3955867 to 692.7936942) | 418.9687326(355.3293936 to 452.0691838) | 6182.013403(5433.887228 to 6644.447971) |
| Western Europe | Female | 2000 | 569.8670437(473.7178516 to 673.1980879) | 395.9959198(333.6348102 to 428.103049) | 5854.213285(5122.032298 to 6305.669134) |
| Western Europe | Female | 2001 | 554.2354134(461.7009815 to 653.5487246) | 381.882514(320.3774157 to 413.4587833) | 5631.556849(4915.102539 to 6074.156864) |
| Western Europe | Female | 2002 | 537.9765843(448.9203593 to 632.2028192) | 374.7394795(313.7836454 to 405.9146513) | 5495.780048(4781.686214 to 5932.026716) |
| Western Europe | Female | 2003 | 521.9083916(436.2591009 to 612.1957988) | 364.1733635(304.0581627 to 394.8114443) | 5326.644232(4625.718376 to 5753.705258) |
| Western Europe | Female | 2004 | 506.4836091(423.6706038 to 593.5159034) | 334.6697504(277.5657917 to 363.623887) | 4934.681381(4261.994887 to 5350.400603) |
| Western Europe | Female | 2005 | 492.0853606(411.7524955 to 576.123941) | 315.2999877(260.5306526 to 343.5252377) | 4675.070473(4031.227792 to 5078.911104) |
| Western Europe | Female | 2006 | 477.6528381(400.4818818 to 559.1995507) | 298.5104208(245.707938 to 326.0072425) | 4438.730906(3816.745147 to 4833.967674) |
| Western Europe | Female | 2007 | 462.2679511(388.4631783 to 540.7205748) | 286.9555034(235.9907393 to 314.1326702) | 4259.952429(3657.646134 to 4645.008085) |
| Western Europe | Female | 2008 | 447.4756368(376.4855526 to 522.8024258) | 281.2390049(230.8117272 to 308.1180251) | 4155.651919(3557.353066 to 4534.682127) |
| Western Europe | Female | 2009 | 434.706579(365.9900307 to 508.1493902) | 268.8322509(219.9084383 to 294.9249474) | 3982.31181(3398.016518 to 4354.518366) |
| Western Europe | Female | 2010 | 425.2319364(357.9263449 to 497.7429524) | 254.4735181(207.2106607 to 279.5364734) | 3789.780107(3232.022905 to 4150.351496) |
| Western Europe | Female | 2011 | 417.7911263(351.8123167 to 488.3037253) | 242.6728124(196.9999994 to 267.0361005) | 3635.213526(3093.045814 to 3992.097607) |
| Western Europe | Female | 2012 | 410.6152926(346.0538941 to 479.6559076) | 237.4612(192.472921 to 261.4230593) | 3554.937861(3024.509409 to 3905.395712) |
| Western Europe | Female | 2013 | 404.2322955(340.4801609 to 472.5147646) | 229.2652814(185.5109072 to 252.6761804) | 3443.095538(2921.125497 to 3785.234144) |
| Western Europe | Female | 2014 | 399.2251483(335.7711667 to 467.2528017) | 221.6298455(178.8359667 to 244.8548559) | 3338.107834(2821.853703 to 3672.2018) |
| Western Europe | Female | 2015 | 396.0206733(332.4475548 to 463.6557603) | 219.4461629(177.4782096 to 242.0622571) | 3304.778494(2795.758175 to 3633.008677) |
| Western Europe | Female | 2016 | 393.6125141(330.4175437 to 460.6007888) | 212.8157001(171.6326889 to 235.1916034) | 3220.948641(2721.877306 to 3546.85647) |
| Western Europe | Female | 2017 | 390.943744(328.3809863 to 457.4539771) | 205.145295(165.0608358 to 226.9609628) | 3123.003405(2633.195617 to 3441.816077) |
| Western Europe | Female | 2018 | 388.3353778(325.5334666 to 454.6767317) | 198.7922144(159.4634155 to 220.3439005) | 3042.511881(2564.927445 to 3353.319272) |
| Western Europe | Female | 2019 | 386.185559(322.7306534 to 453.2881364) | 191.4305369(152.8466019 to 212.6711362) | 2950.488292(2485.902806 to 3260.978847) |
| Western Europe | Female | 2020 | 378.7051415(314.8000354 to 446.4085924) | 189.2328594(150.3814257 to 210.6731933) | 2906.111025(2444.8587 to 3214.794339) |
| Western Europe | Female | 2021 | 377.3198895(314.4670829 to 447.4695621) | 182.507385(144.1991713 to 203.6824475) | 2830.840334(2373.625649 to 3138.497928) |
| Western Sub-Saharan Africa | Female | 1990 | 1002.763911(790.8385621 to 1253.554132) | 1107.594182(897.4508985 to 1335.446047) | 19310.97332(15839.06435 to 23161.56536) |
| Western Sub-Saharan Africa | Female | 1991 | 993.9943352(787.8580769 to 1238.076071) | 1091.989184(888.0836554 to 1304.028964) | 19068.01038(15679.50073 to 22717.14393) |
| Western Sub-Saharan Africa | Female | 1992 | 985.7256106(785.4553394 to 1224.562073) | 1084.57953(885.037971 to 1301.191714) | 18936.36097(15623.05649 to 22628.75257) |
| Western Sub-Saharan Africa | Female | 1993 | 978.4712793(782.9116201 to 1213.4767) | 1072.930215(870.3653167 to 1277.111496) | 18728.16712(15367.12131 to 22217.23853) |
| Western Sub-Saharan Africa | Female | 1994 | 972.7041519(780.2684178 to 1204.548263) | 1064.858264(856.9957808 to 1279.86126) | 18585.37726(15133.11452 to 22237.96958) |
| Western Sub-Saharan Africa | Female | 1995 | 968.7341798(778.5463017 to 1198.126532) | 1057.343733(851.3199734 to 1265.844085) | 18454.83149(15005.19915 to 22070.95482) |
| Western Sub-Saharan Africa | Female | 1996 | 966.4461596(777.0280249 to 1194.504202) | 1049.424505(850.3254367 to 1251.528204) | 18309.35453(14955.92938 to 21728.07532) |
| Western Sub-Saharan Africa | Female | 1997 | 964.5625842(775.5850021 to 1192.714341) | 1042.243492(839.4119379 to 1262.371353) | 18172.26423(14806.36208 to 22037.32124) |
| Western Sub-Saharan Africa | Female | 1998 | 962.7867553(773.1219032 to 1190.559468) | 1032.570636(824.9156007 to 1257.876892) | 17992.90672(14574.31908 to 22009.79367) |
[truncated: 725,538 more chars]
